# Decarboxylative Polyfluoroarylation of Alkylcarboxylic Acids

**DOI:** 10.1002/anie.202015596

**Published:** 2021-03-30

**Authors:** Xiang Sun, Tobias Ritter

**Affiliations:** ^1^ Max-Planck-Institut für Kohlenforschung Kaiser-Wilhelm-Platz 1 45470 Mülheim an der Ruhr Germany

**Keywords:** decarboxylation polyfluoroarylation, defluorinative alkylation, late-stage functionalization, photoredox catalysis, radical reaction

## Abstract

Polyfluoroarenes are useful building blocks in several areas such as drug discovery, materials, and crop protection. Herein, we report the first polyfluoroarylation of aliphatic carboxylic acids via photoredox decarboxylation. The method proceeds with broad substrate scope and high functional group tolerance. Moreover, small complex molecules such as natural products and drugs can be modified by late‐stage modification.

## Graphical abstract

A photocatalyzed decarboxylative polyfluoroarylation of aliphatic carboxylic acids is reported. The defluorinative C−C bond formation through a radical process enables expedient access to polyfluoroaromatics from abundant carboxylic acids.

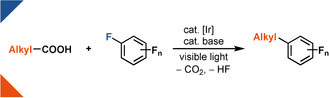

In 2019, two additional polyfluoroaryl‐containing drugs were approved by the FDA,^[1]^ which further expanded the documented utility of polyfluoroarenes in drug discovery.^[2]^ In addition to their use in pharmaceuticals, polyfluoroaromatics also play an important role in materials^[3]^ and pesticides,^[4]^ likely as a consequence of their specific properties such as low oxidation potential and metabolic stability. While mono‐fluorinated arenes are typically made by introduction of a single fluorine substituent by C−X[^5^, ^6^] and C−H bond fluorination,[^5^, ^7^] polyfluoroarene synthesis can be approached by the defluorinative functionalization of readily accessible, simple polyfluoroarenes.[^8^, ^9^] Nucleophilic aromatic substitution (S_N_Ar) of fluoride and transition‐metal‐catalyzed C−F bond functionalization represent two main routes.^[8]^ In these methods, preformed organometallic reagents such as alkyl‐ or aryl lithium, ‐Grignard, or ‐zinc reagents are normally required as carbon nucleophiles that come with their common limitation in substrate scope due to deleterious reactions. Here we show the first decarboxylative polyfluoroarylation of aliphatic carboxylic acids. The transformation is enabled by radical addition of alkyl radicals to polyfluoroarenes via photoredox decarboxylation of readily available aliphatic carboxylic acids and conceptually differs from previously published radical addition to polyfluoroarenes: The expedient synthesis of otherwise challenging‐to‐access, substituted fluoroarenes is enabled by an approach distinct from prior art through mild generation of alkyl radicals combined with single electron reduction of the resulting fluoroaryl radical, which results in broad substrate scope and high functional group tolerance of the new carbon–carbon bond‐forming reaction.

Simple polyfluorinated arenes are produced as inexpensive feedstocks by the Halex process.^[8b]^ Derivatization by defluorinative alkylation with organometallic reagents is challenging for complex small molecules because their conversion to organometallics can be difficult as a consequence of the presence of diverse functional groups. To avoid the use of stoichiometric alkylmetallic reagents, catalytic in situ generation of alkyl‐cuprates from alkenes was reported^[10]^ but despite great advances, only styrenes and activated alkenes were demonstrated to successfully participate in this reaction. The Weaver group reported the addition of perfluorinated (het)arene‐derived radicals to π systems, including alkenes, alkynes, and arenes,^[11]^ with radicals being generated through photocatalytic single‐electron reductive C−F fragmentation.[^11d^, ^12^] Although large in scope, to suppress competitive hydrogen abstraction transfer,^[13]^ alkenes were required in an excess, which is not desirable for our goal of late‐stage functionalization. Hashmi and co‐workers reported an elegant polyfluoroarylation of tertiary anilines by a mechanistically distinct radical–radical coupling of α‐amino radicals derived from tertiary anilines and [Ir]‐stabilized polyfluoroaryl radical anions.^[14]^ A general defluorinative alkylation of oligofluoroarenes that can also be used in late‐stage functionalization is still challenging. Here, we attempt to fill this gap: Irradiation of a reaction mixture containing a carboxylic acid and a polyfluoroarene in the presence of a base and a photoredox catalyst, results in defluorinative alkylation on a gram scale. While the aforementioned methods generally require perfluoro(het)arenes, our alkylation also proceeds successfully to afford trifluoro and tetrafluoroarene‐substituted molecules that are of large synthetic value for example for drug development (Scheme 1). Addition to arenes with smaller fluorine content, such as trifluoroarenes to produce difluoroaryl‐substituted compounds, did not proceed in synthetically useful yields.

**Scheme 1:**
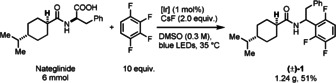
Gram‐scale decarboxylative polyfluoroarylation of nateglinide. [Ir] represents Ir(dFFppy)_2_(dtbpy)PF_6_.

The reactivity of polyfluoroarenes as radical acceptors dates back to independent reports by Williams and Kobrina from the 1960s.^[15]^ Addition of carbon radicals that were generated from peroxides formed alkylated perfluorobenzenes but also resulted in dimerization of the radical adduct to produce compound mixtures of little synthetic value (Scheme 2 a).[^8b^, ^15^] Similarly, UV‐irradiation of a solution of hexafluorobenzene in cyclohexane or in methanol in the presence of benzophenone led to simple alkylated pentafluorophenyl derivatives in product mixtures.^[16]^ Radical addition to perfluoroarenes results in neutral radicals that would need to formally lose a fluorine radical for productive alkylation. This process is inefficient due to the large C−F bond dissociation energy (BDE=145 kcal mol^−1^ in C_6_F_6_ ^[17]^), and results in competing dimerization and hydroalkylation, which is the reason that defluorinative alkylation through radical addition to fluoroarenes is of little synthetic value so far. Perfluoroarenes and related electron‐deficient arenes can be reduced to the corresponding radical anions by excited photoredox catalysts from which the carbon fluoride bond can be more readily cleaved heterolytically by elimination of fluoride to generate a synthetically useful aryl radical, as shown by Weaver for addition of the aryl radical to π systems.[^11^, ^13^] Our reaction design is conceptually different in that we hypothesized to add a radical to the perfluoroarene, and subsequently reduce the resulting radical to an anion, from which fluoride could be readily eliminated. Based on this hypothesis, we designed a process, in which a photoredox catalyst, reduced in its excited state by a carboxylate,[^18^, ^19^] has appropriate redox potential to reduce the initially formed radical adduct to an anion (Scheme 2 b). Consequently, we have employed a cationic photoredox catalyst that is less reducing in its excited state than the catalysts used for fluoroarene reduction chemistry^[11a]^ (*E*_ox_(Ir^IV^/*Ir^III^)=−1.17 V vs. SCE in MeCN^[20]^). The ability of our system to also furnish fluoroarenes with smaller fluorine content such as trifluoroarenes is likely a consequence of the different reduction potentials compared to catalysts employed in other, related transformations that function through arene reduction. On the other hand, pentafluoropyridine that works well in reactions that reduce the arene cannot be used as reagent in our reaction possibly because it is too oxidizing.

**Scheme 2:**
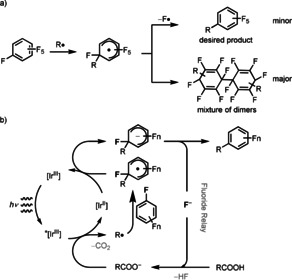
a) Perfluorobenzene as radical acceptor. b) Our reaction design.

Ir(dFFppy)_2_(dtbpy)PF_6_ was identified as the most suitable photoredox catalyst, while neither Mes‐Acr‐Me⋅ClO_4_ nor 4CzlPN (2,4,5,6‐tetra(9*H*‐carbazol‐9‐yl)isophthalonitrile) can promote this reaction (see the Supporting Information, Table S1). Examination of the base showed that Li_2_CO_3_ provided higher yields than other carbonate salts. Likewise, a catalytic amount of CsF (20 mol %) could also promote the reaction in high yields, possibly explained through the fluoride relay shown in Scheme 2 (see Supporting Information, Table S2). Evaluation of solvents revealed DMSO as best solvent. A lower yield (from 93 % to 70 %) was observed when the reaction was set up in wet DMSO with 10 equiv of H_2_O (see the Supporting Information, Table S3). Control experiments proved that light irradiation, photocatalyst, and base are each essential to this transformation (see the Supporting Information, Table S4).

Two equivalents of perfluorobenzene is enough for reactions with sufficiently nucleophilic radicals such as that derived from *N*‐Boc glycine but higher loading of perfluoroarenes are required with substrates that produce carbon radicals with lower nucleophilicity^[21]^ (see the Supporting Information, Table S5 and S6). Due to its low cost, we employed perfluorobenzene in access (5–10 equiv) for evaluation of the substrate scope with respect to the carboxylic acid, which resulted in higher product yield and shorter reaction time (Table 1). A variety of structurally and functionally complex carboxylic acids are compatible with the mild reaction conditions. α‐Amino acids (**7**), α‐oxy‐substituted carboxylic acids (**11** and **12**), and aliphatic carboxylic acids that result in the formation of secondary radicals (**2**, **3**, **4**, and **6**) are tolerated. Di‐perfluoroarylation is achievable with di‐carboxylic acids (**16**). The reaction tolerates hydroxyl groups (**8** and **10**), carbonyl groups (**20**), thiazoles (**9**), amides (**4**), and indoles (**5**). Late‐stage functionalization of natural products (**21**) and drug molecules (**13** and **17**) is possible as well. Carboxylic acids that result in the formation of less nucleophilic, primary radicals (**15**) afford lower yield^[21]^ when compared to the transformations through secondary and tertiary carbon radicals (**14**), respectively.

**Table 1:** Substrate scope of carboxylic acids.

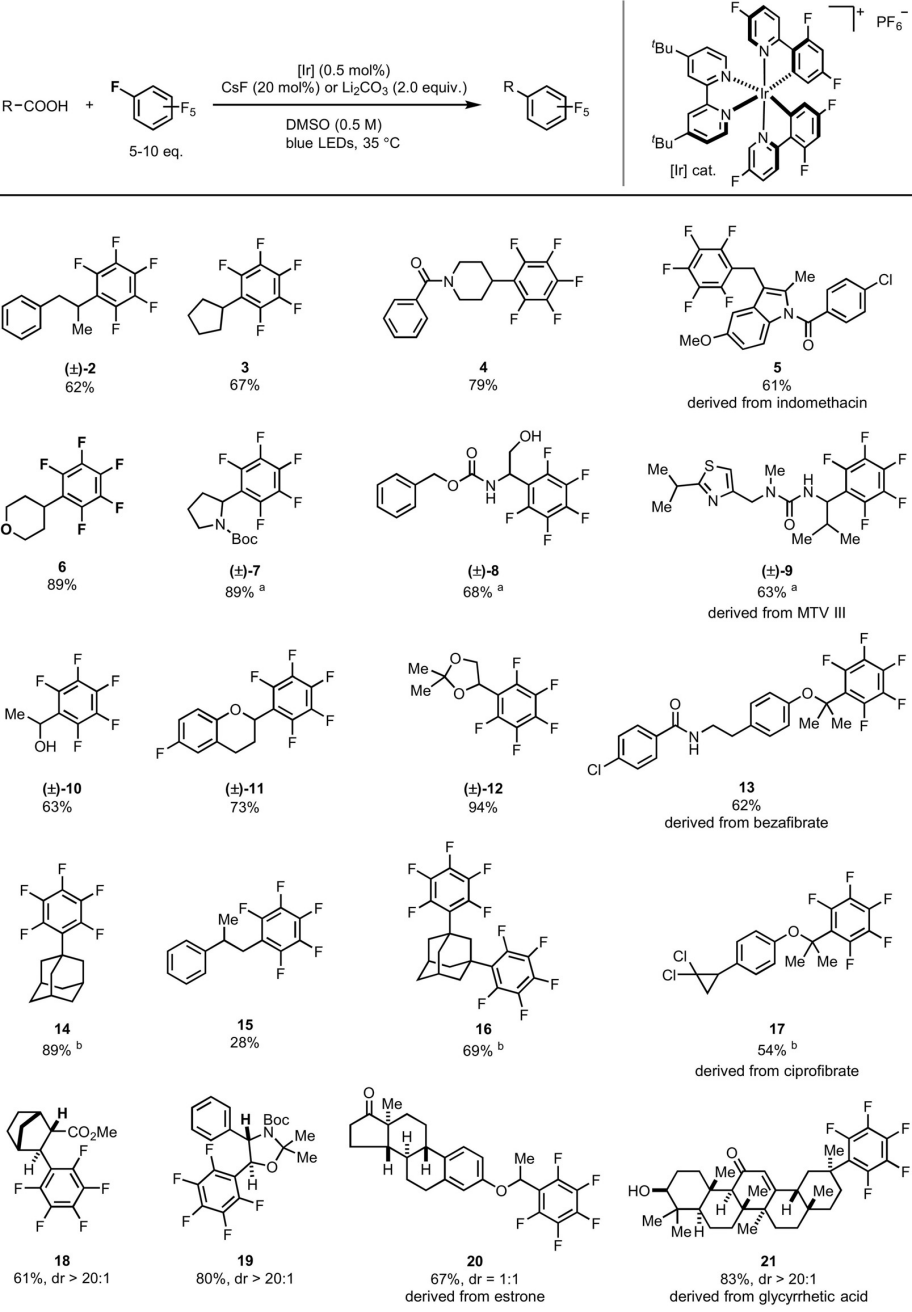

General conditions except where otherwise noted: carboxylic acid (0.3 mmol), perfluorobenzene (3.0 mmol, 10 equiv), Ir(dFFppy)_2_(dtbpy)PF_6_ (0.5 mol %), Li_2_CO_3_ (2.0 equiv), DMSO (0.5 M), blue LEDs, 35 °C. [a] Perfluorobenzene (1.5 mmol, 5.0 equiv), CsF (20 mol %). [b] Perfluorobenzene (1.5 mmol, 5.0 equiv).

Several simple polyfluoroarenes are suitable for the decarboxylative polyfluoroarylation (Table 2). While higher yields are observed with higher fluorine substitution, alkylated arenes with as few as three fluorine atoms, in the absence of other substituents, are achievable as demonstrated by reaction with *N*‐Boc glycine (**27**). Constitutional isomers that resulted from addition at different positions for unsymmetric fluoroarenes could all be purified chromatographically (**23**, **24**, **25**, **26**, **31**, **32**, and **33**). The observed selectivity is reminiscent to that observed in other radical addition reactions to (het)arenes.^[22]^ The reaction is chemoselective: chloro‐fluoro arenes reacted exclusively with fluorine substitution (**23**, **28**, and **30**), which is in contrast to the reactivity observed by the Weaver group.[^11^, ^13^]

**Table 2:** Substrate scope of polyfluoroarenes.

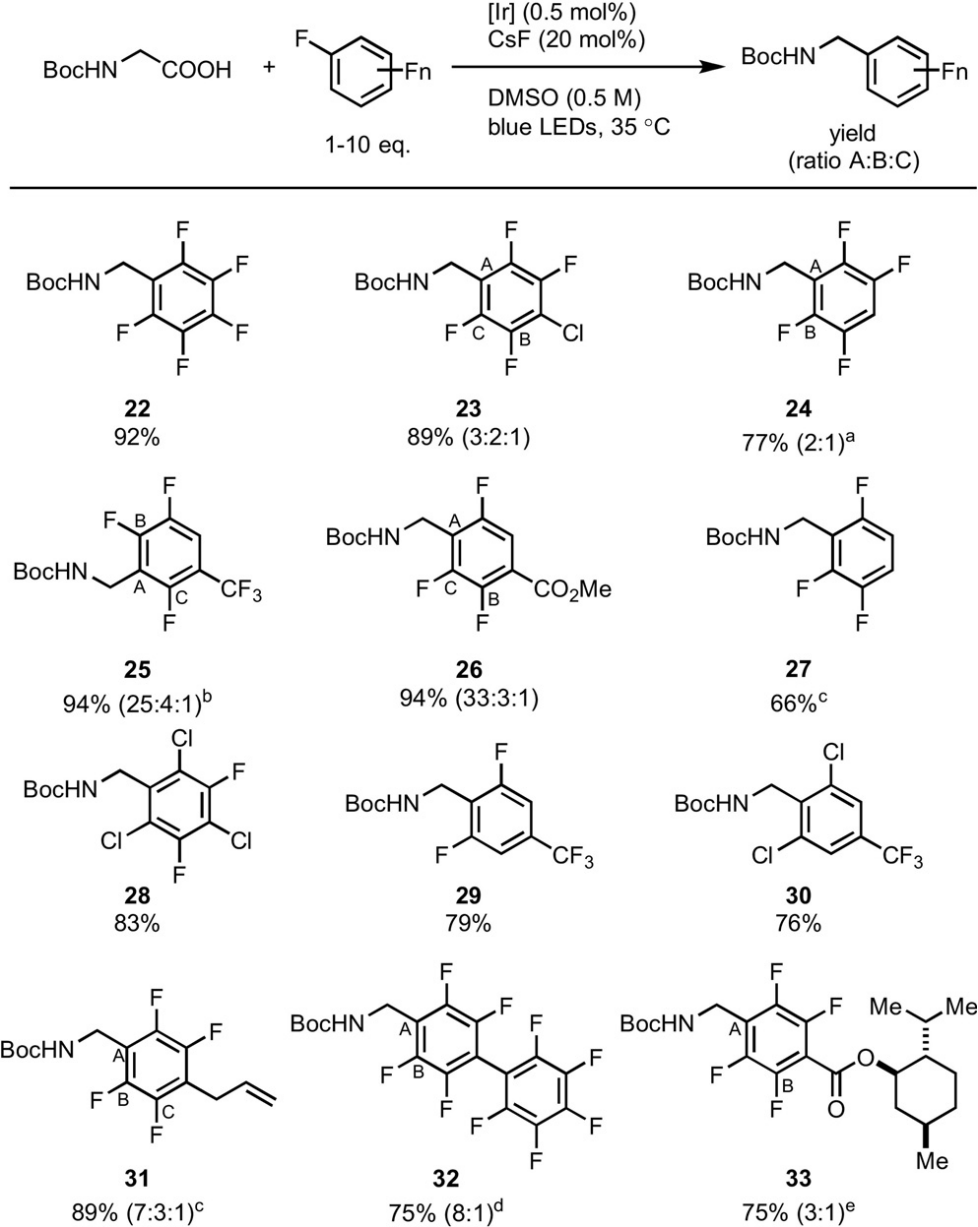

General conditions except where otherwise noted: *N*‐Boc glycine (0.3 mmol), polyfluoroarene (0.6 mmol, 2.0 equiv), Ir(dFFppy)_2_(dtbpy)PF_6_ (0.5 mol %), CsF (20 mol %), DMSO (0.5 M), blue LEDs, 35 °C. All products have been isolated and characterized as analytically pure samples. ^A,B,C^ denote sites of substitution of the substrate; the ratio of the constitutional isomers is reported based on ^19^F NMR spectroscopy of the reaction mixture. [a] Polyfluoroarene (1.5 mmol, 5.0 equiv). [b] Li_2_CO_3_ (2.0 equiv) instead of CsF (20 mol %). [c] Polyfluoroarene (3.0 mmol, 10 equiv). [d] DMSO (0.15 M). [e] Polyfluoroarene (0.3 mmol, 1.0 equiv).

Preliminary experiments to elucidate the mechanism of the transformation are consistent with our original reaction design (Scheme 2 b). A Stern–Volmer analysis revealed that photoexited [Ir^III^] was quenched by carboxylate rather than perfluorobenzene (see the Supporting Information, Scheme S3). Reductive quenching of photoexcited [Ir^III^] (*E*_red_(*Ir^III^/Ir^II^)=1.48 V vs. SCE in MeCN^[20]^) by carboxylates (*E*_ox_(carboxylate) ranges from 0.95 V to 1.25 V vs. SCE in MeCN[^19a^, ^23^]) results in [Ir^II^] and, after decarboxylation, a carbon radical. Carbon radical generation is supported by formation of product **34 a** (Scheme 3 a) that is expected to form upon fast radical cyclization of the 5‐hexenyl radical prior to addition to the fluoroarene. Regeneration of the [Ir^III^] catalyst by reduction to the anion followed by fluoride elimination would conclude the cycle. While Hashmi proposed a radical–radical coupling pathway for the C−C formation in their work,[^14^, ^19a^] that pathway is not favored in our reaction. First, the single‐electron reduction of perfluoroarene by the iridium photocatalyst is not likely to happen in consideration of their redox potentials (*E*_red_(C_6_F_6_)=−2.85 V vs. SCE in THF,^[24]^ *E*_ox_(Ir^III^/Ir^II^)=−1.32 V vs. SCE in MeCN^[20]^). Plus, by adding ^*t*^Bu‐acetylene, norbornene, and 1,3,5‐trimethoxybenzene as scavengers, no perfluorophenyl radical adduct was observed. (Scheme 3 b). Additionally, in the reaction of chloro‐fluoro arenes, the observed exclusive fluorine substitution also renders the polyfluoroaryl radical anion generation in the reaction process unlikely.^[12]^

**Scheme 3:**
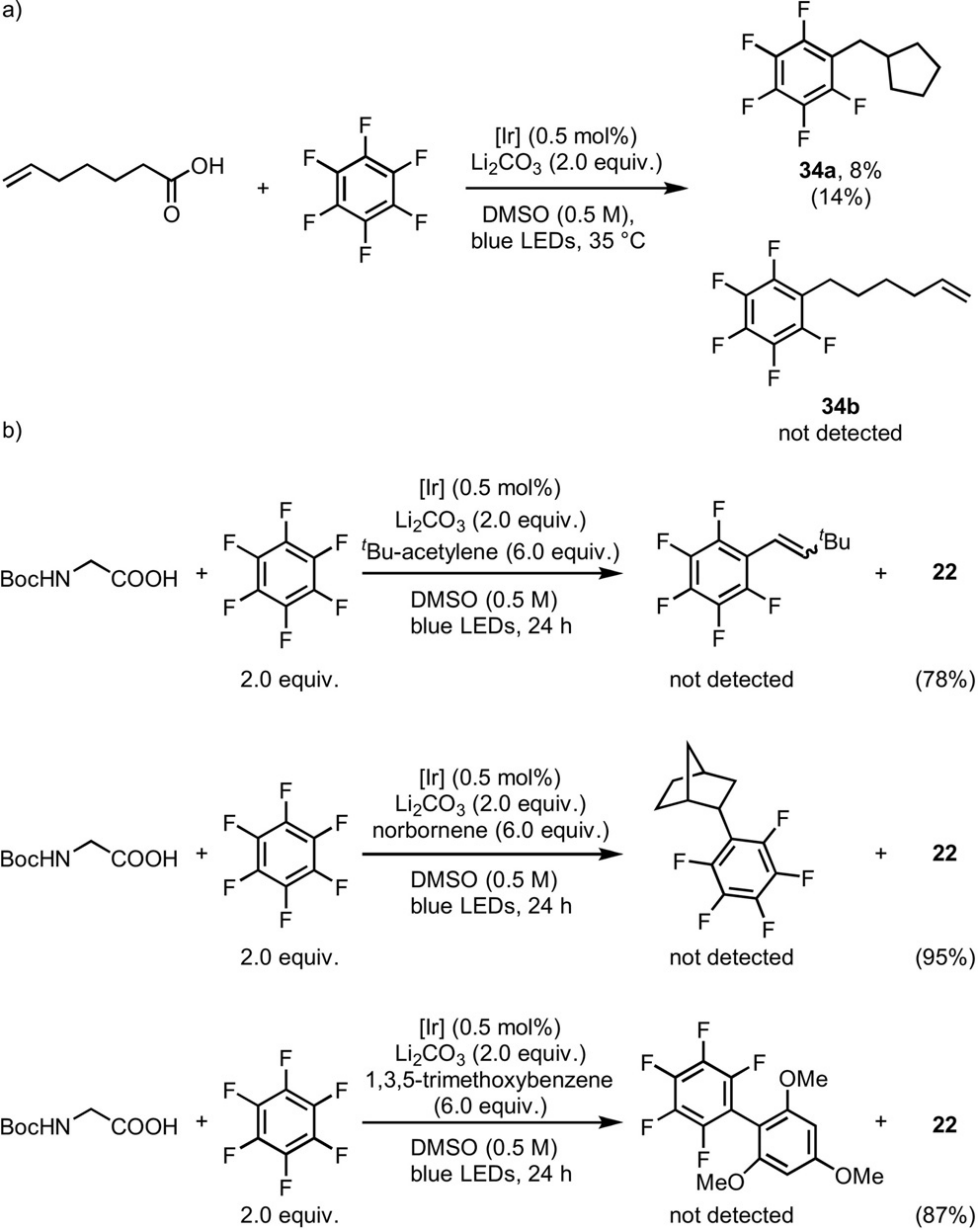
Mechanism study. a) Radical cyclization trapped by perfluorobenzene. b) Trials to trap perfluorophenyl radical. Yields in bracket are based on ^19^F NMR.

In conclusion, we have reported a defluorinative alkylation with aliphatic carboxylic acids via photodecarboxylation. Polyfluoroaryl moieties can be easily introduced to molecules with structural complexity. The radical pathway of carbon radical addition to polyfluoroarenes coupled with photocatalysis can provide a platform for polyfluoroaromatics synthesis with various carbon radical precursors.

## Conflict of interest

The authors declare no conflict of interest.

## Supporting information

As a service to our authors and readers, this journal provides supporting information supplied by the authors. Such materials are peer reviewed and may be re‐organized for online delivery, but are not copy‐edited or typeset. Technical support issues arising from supporting information (other than missing files) should be addressed to the authors.

Supplementary
