# Supplementary material for: Decarboxylative Polyfluoroarylation of Alkylcarboxylic Acids
Source: Angew Chem Int Ed Engl. 2021 Mar 30;60(19):10557–62. doi: 10.1002/anie.202015596 (PMC8252513; doi:10.1002/anie.202015596)
Supplement: Supplementary file 1 — Supplementary [file ANIE-60-10557-s001.pdf]

Angewandte Chemie
Eine Zeitschrift der Gesellschaft Deutscher Chemiker

Supporting Information

Decarboxylative Polyfluoroarylation of Alkylcarboxylic Acids

*Xiang Sun and Tobias Ritter**

anie_202015596_sm_miscellaneous_information.pdf

TABLE OF CONTENTS

TABLE OF CONTENTS 1

MATERIALS AND METHODS 8

EXPERIMENTAL DATA 9

Representative procedure for decarboxylative polyfluoroarlyation 9

Reaction optimization for decarboxylative polyfluoroaryltion 10

Table S1. Photocatalyst optimization 10
Table S2. Base optimization 10
Table S3. Solvent optimization 11
Table S4. Control experiments 11
Table S5. Amount of perfluorobenzene optimization with 2-methyl-3-phenylpropanoic acid 11
Table S6. Amount of perfluorobenzene optimization with 6-fluorochromane-2-carboxylic acid 12
Table S7. List of unsuccessful substrates 12

Preparation of substrates 13

(1R,2S,5R)-2-Isopropyl-5-methylcyclohexylpentafluorobenzoate (**S1**) 13
2-((1,3,5[10]-Estratrien-17-one-3-yl)oxy)propanoic acid (**S2**) 13

Decarboxylative polyfluoroarylation of carboxylic acids 15

Nateglinide derivative **1** 15
Pentafluoro-(1-phenylpropan-2-yl)benzene (**2**) 15
Cyclopentylpentafluorobenzene (**3**) 16
N-Benzoyl-4-perfluorophenylpiperidine (**4**) 17
Indomethacin derivative **5** 18
4-(Perfluorophenyl)tetrahydropyran (**6**) 18
N-Boc-2-perfluoropehnylpyrrolidine (**7**) 19
N-Cbz-1-hydroxyl-2-perfluoropehnylethan-2-amine (**8**) 20
MTV III derivative **9** 21
1-(Perfluorophenyl)ethanol (**10**) 21
6-Fluorochromane-2-pentafluorobenzene (**11**) 22
2,2-Dimethyl-4-(perfluorophenyl)-1,3-dioxolane (**12**) 23
Bezafibrate derivative **13** 23
1-(Perfluorophenyl)adamantane (**14**) 24
Pentafluoro-(2-phenylpropyl)benzene (**15**) 25
1,3-Bis(perfluorophenyl)adamantane (**16**) 26

Ciprofibrate derivative **17** ... 26
(2S,3R)-2-(Methylcarboxyl)-3-pentafluorophenylnorbornan (**18**) ... 27
(4S,5S)-*N*-Boc-2,2-dimethyl-4-phenyl-5-pentafluorophenyloxazolidine (**19**) ... 28
Estrone derivative **20** ... 29
Glycyrrhetic acid derivative **21** ... 30
N-Boc-pentafluorobenzylamine (**22**) ... 30
N-Boc-chlorotetrafluorobenzylamines **23a**, **23b**, **23c** ... 31
N-Boc-tetrafluorobenzylamines **24a**, **24b** ... 33
N-Boc-trifluoro-(trifluoromethyl)benzylamines **25a**, **25b**, **25c** ... 34
N-Boc-trifluoro-methylcarboxylbenzylamines **26a**, **26b**, **26c** ... 36
N-Boc-2,3,6-trifluorobenzylamine (**27**) ... 38
N-Boc-2,4,6-trichloro-3,5-difluorobenzylamine (**28**) ... 38
N-Boc-4-trifluoromethyl-2,6-difluorobenzylamine (**29**) ... 39
N-Boc-4-trifluoromethyl-2,6-dichlorobenzylamine (**30**) ... 40
N-Boc-allyl-tetrafluorobenzylamines **31a**, **31b**, **31c** ... 40
N-Boc-tetrafluoro-(pentafluorophenyl)benzylamines **32a**, **32b** ... 42
N-Boc-tetrafluoro-((1'R,2'S,5'R)-2'-isopropyl-5'-methylcyclohexylcarboxyl)benzylamines **33a**, **33b** .. 43

Mechanism study ... 45

Proposed possible mechanism ... 45
Evidence for carbon radical ... 46
Experiments to trap perfluorophenyl radical ... 47
Quantum yield measurement ... 47
Stern-Volmer quenching experiment ... 49
Cyclic voltammograms of $Ir(dFFppy)_2(dtbpy)PF_6$ ... 50
Investigation of the low yield of primary carboxylic acid ... 52

SPECTROSCOPIC DATA ... 53

^{1}H NMR of (1R,2S,5R)-2-isopropyl-5-methylcyclohexylpentafluorobenzoate (**S1**) ... 53

^{13}C NMR of (1R,2S,5R)-2-isopropyl-5-methylcyclohexylpentafluorobenzoate (**S1**) ... 54

^{19}F NMR of (1R,2S,5R)-2-isopropyl-5-methylcyclohexylpentafluorobenzoate (**S1**) ... 55

^{1}H NMR of 2-((1,3,5[10]-estratrien-17-one-3-yl)oxy)propanoic acid (**S2**) ... 56

^{13}C NMR of 2-((1,3,5[10]-estratrien-17-one-3-yl)oxy)propanoic acid (**S2**) ... 57

^{1}H NMR of Nateglinide derivative **1** ... 58

^{13}C NMR of Nateglinide derivative **1** ... 59

^{19}F NMR of Nateglinide derivative **1** ... 60

^{1}H NMR of Pentafluoro-(1-phenylpropan-2-yl)benzene (**2**) 61

^{13}C NMR of Pentafluoro-(1-phenylpropan-2-yl)benzene (**2**) 62

^{19}F NMR of Pentafluoro-(1-phenylpropan-2-yl)benzene (**2**) 63

^{1}H NMR of Cyclopentylpentafluorobenzene (**3**) 64

^{13}C NMR of Cyclopentylpentafluorobenzene (**3**) 65

^{19}F NMR of Cyclopentylpentafluorobenzene (**3**) 66

^{1}H NMR of *N*-Benzoyl-4-perfluorophenylpiperidine (**4**) 67

^{13}C NMR of *N*-Benzoyl-4-perfluorophenylpiperidine (**4**) 68

^{19}F NMR of *N*-Benzoyl-4-perfluorophenylpiperidine (**4**) 69

^{1}H NMR of Indomethacin derivative **5** 70

^{13}C NMR of Indomethacin derivative **5** 71

^{19}F NMR of Indomethacin derivative **5** 72

^{1}H NMR of 4-(Perfluorophenyl)tetrahydropyran (**6**) 73

^{13}C NMR of 4-(Perfluorophenyl)tetrahydropyran (**6**) 74

^{19}F NMR of 4-(Perfluorophenyl)tetrahydropyran (**6**) 75

^{1}H NMR of *N*-Boc-2-perfluoropehnylpyrrolidine (**7**) 76

^{13}C NMR of *N*-Boc-2-perfluoropehnylpyrrolidine (**7**) 77

^{19}F NMR of *N*-Boc-2-perfluoropehnylpyrrolidine (**7**) 78

^{1}H NMR of *N*-Cbz-1-hydroxyl-2-perfluoropehnylethan-2-amine (**8**) 79

^{13}C NMR of *N*-Cbz-1-hydroxyl-2-perfluoropehnylethan-2-amine (**8**) 80

^{19}F NMR of *N*-Cbz-1-hydroxyl-2-perfluoropehnylethan-2-amine (**8**) 81

^{1}H NMR of MTV III derivative **9** 82

^{13}C NMR of MTV III derivative **9** 83

^{19}F NMR of MTV III derivative **9** 84

^{1}H NMR of 1-(Perfluorophenyl)ethanol (**10**) 85

^{13}C NMR of 1-(Perfluorophenyl)ethanol (**10**) 86

^{19}F NMR of 1-(Perfluorophenyl)ethanol (**10**) 87

^{1}H NMR of 6-Fluorochromane-2-pentafluorobenzene (**11**) 88

^{13}C NMR of 6-Fluorochromane-2-pentafluorobenzene (**11**) 89

^{19}F NMR of 6-Fluorochromane-2-pentafluorobenzene (**11**) 90
^{1}H NMR of 2,2-Dimethyl-4-(perfluorophenyl)-1,3-dioxolane (**12**) 91
^{13}C NMR of 2,2-Dimethyl-4-(perfluorophenyl)-1,3-dioxolane (**12**) 92
^{19}F NMR of 2,2-Dimethyl-4-(perfluorophenyl)-1,3-dioxolane (**12**) 93
^{1}H NMR of Bezafibrate derivative **13** 94
^{13}C NMR of Bezafibrate derivative **13** 95
^{19}F NMR of Bezafibrate derivative **13** 96
^{1}H NMR of 1-(Perfluorophenyl)adamantane (**14**) 97
^{13}C NMR of 1-(Perfluorophenyl)adamantane (**14**) 98
^{19}F NMR of 1-(Perfluorophenyl)adamantane (**14**) 99
^{1}H NMR of Pentafluoro-(2-phenylpropyl)benzene (**15**) 100
^{13}C NMR of Pentafluoro-(2-phenylpropyl)benzene (**15**) 101
^{19}F NMR of Pentafluoro-(2-phenylpropyl)benzene (**15**) 102
^{1}H NMR of 1,3-Bis(perfluorophenyl)adamantane (**16**) 103
^{13}C NMR of 1,3-Bis(perfluorophenyl)adamantane (**16**) 104
^{19}F NMR of 1,3-Bis(perfluorophenyl)adamantane (**16**) 105
^{1}H NMR of Ciprofibrate derivative **17** 106
^{13}C NMR of Ciprofibrate derivative **17** 107
^{19}F NMR of Ciprofibrate derivative **17** 108
^{1}H NMR of (2S,3R)-2-(Methylcarboxyl)-3-pentafluorophenylnorbornan (**18**) 109
^{13}C NMR of (2S,3R)-2-(Methylcarboxyl)-3-pentafluorophenylnorbornan (**18**) 110
^{19}F NMR of (2S,3R)-2-(Methylcarboxyl)-3-pentafluorophenylnorbornan (**18**) 111
H-H-NOESY spectrum of (2S,3R)-2-(Methylcarboxyl)-3-pentafluorophenylnorbornan (**18**) 112
H-F-NOESY spectrum of (2S,3R)-2-(Methylcarboxyl)-3-pentafluorophenylnorbornan (**18**) 113
^{1}H NMR of (4S,5S)-*N*-Boc-2,2-dimethyl-4-phenyl-5-pentafluorophenyloxazolidine (**19**) 114
^{13}C NMR of (4S,5S)-*N*-Boc-2,2-dimethyl-4-phenyl-5-pentafluorophenyloxazolidine (**19**) 115
^{19}F NMR of (4S,5S)-*N*-Boc-2,2-dimethyl-4-phenyl-5-pentafluorophenyloxazolidine (**19**) 116
H-H-NOESY spectrum of (4S,5S)-*N*-Boc-2,2-dimethyl-4-phenyl-5-pentafluorophenyloxazolidine (**19**) ... 117
^{1}H NMR of Estrone derivative **20** 118

^{13}C NMR of Estrone derivative **20** 119

^{19}F NMR of Estrone derivative **20** 120

^{1}H NMR of Glycyrrhetic acid derivative **21** 121

^{13}C NMR of Glycyrrhetic acid derivative **21** 122

^{19}F NMR of Glycyrrhetic acid derivative **21** 123

H-H-NOESY spectrum of Glycyrrhetic acid derivative **21** 124

^{1}H NMR of *N*-Boc-pentafluorobenzylamine (**22**) 125

^{13}C NMR of *N*-Boc-pentafluorobenzylamine (**22**) 126

^{19}F NMR of *N*-Boc-pentafluorobenzylamine (**22**) 127

^{1}H NMR of *N*-Boc-4-chlorotetrafluorobenzylamine (**23a**) 128

^{13}C NMR of *N*-Boc-4-chlorotetrafluorobenzylamine (**23a**) 129

^{19}F NMR of *N*-Boc-4-chlorotetrafluorobenzylamine (**23a**) 130

^{1}H NMR of *N*-Boc-2-chlorotetrafluorobenzylamine (**23b**) 131

^{13}C NMR of *N*-Boc-2-chlorotetrafluorobenzylamine (**23b**) 132

^{19}F NMR of *N*-Boc-2-chlorotetrafluorobenzylamine (**23b**) 133

^{1}H NMR of *N*-Boc-3-chlorotetrafluorobenzylamine (**23c**) 134

^{13}C NMR of *N*-Boc-3-chlorotetrafluorobenzylamine (**23c**) 135

^{19}F NMR of *N*-Boc-3-chlorotetrafluorobenzylamine (**23c**) 136

^{1}H NMR of *N*-Boc-2,3,5,6-tetrafluorobenzylamine (**24a**) 137

^{13}C NMR of *N*-Boc-2,3,5,6-tetrafluorobenzylamine (**24a**) 138

^{19}F NMR of *N*-Boc-2,3,5,6-tetrafluorobenzylamine (**24a**) 139

^{1}H NMR of *N*-Boc-2,3,4,6-tetrafluorobenzylamine (**24b**) 140

^{13}C NMR of *N*-Boc-2,3,4,6-tetrafluorobenzylamine (**24b**) 141

^{19}F NMR of *N*-Boc-2,3,4,6-tetrafluorobenzylamine (**24b**) 142

^{1}H NMR of *N*-Boc-2,3,6-trifluoro-5-(trifluoromethyl)benzylamine (**25a**) 143

^{13}C NMR of *N*-Boc-2,3,6-trifluoro-5-(trifluoromethyl)benzylamine (**25a**) 144

^{19}F NMR of *N*-Boc-2,3,6-trifluoro-5-(trifluoromethyl)benzylamine (**25a**) 145

^{1}H NMR of *N*-Boc-2,3,6-trifluoro-4-(trifluoromethyl)benzylamine (**25b**) 146

^{13}C NMR of *N*-Boc-2,3,6-trifluoro-4-(trifluoromethyl)benzylamine (**25b**) 147

^{19}F NMR of *N*-Boc-2,3,6-trifluoro-4-(trifluoromethyl)benzylamine (**25b**) 148

^{1}H NMR of *N*-Boc-2,3,4-trifluoro-6-(trifluoromethyl)benzylamine (**25c**) 149

^{13}C NMR of *N*-Boc-2,3,4-trifluoro-6-(trifluoromethyl)benzylamine (**25c**) 150

^{19}F NMR of *N*-Boc-2,3,4-trifluoro-6-(trifluoromethyl)benzylamine (**25c**) 151

^{1}H NMR of *N*-Boc-2,3,6-trifluoro-4-(methyl)carboxylbenzylamine (**26a**) 152

^{13}C NMR of *N*-Boc-2,3,6-trifluoro-4-(methyl)carboxylbenzylamine (**26a**) 153

^{19}F NMR of *N*-Boc-2,3,6-trifluoro-4-(methyl)carboxylbenzylamine (**26a**) 154

^{1}H NMR of *N*-Boc-2,3,4-trifluoro-6-(methyl)carboxylbenzylamine (**26b**) 155

^{13}C NMR of *N*-Boc-2,3,4-trifluoro-6-(methyl)carboxylbenzylamine (**26b**) 156

^{19}F NMR of *N*-Boc-2,3,4-trifluoro-6-(methyl)carboxylbenzylamine (**26b**) 157

^{1}H NMR of *N*-Boc-2,3,6-trifluoro-5-(methyl)carboxylbenzylamine (**26c**) 158

^{13}C NMR of *N*-Boc-2,3,6-trifluoro-5-(methyl)carboxylbenzylamine (**26c**) 159

^{19}F NMR of *N*-Boc-2,3,6-trifluoro-5-(methyl)carboxylbenzylamine (**26c**) 160

^{1}H NMR of *N*-Boc-2,3,6-trifluorobenzylamine (**27**) 161

^{13}C NMR of *N*-Boc-2,3,6-trifluorobenzylamine (**27**) 162

^{19}F NMR of *N*-Boc-2,3,6-trifluorobenzylamine (**27**) 163

^{1}H NMR of *N*-Boc-2,4,6-trichloro-3,5-difluorobenzylamine (**28**) 164

^{13}C NMR of *N*-Boc-2,4,6-trichloro-3,5-difluorobenzylamine (**28**) 165

^{19}F NMR of *N*-Boc-2,4,6-trichloro-3,5-difluorobenzylamine (**28**) 166

^{1}H NMR of *N*-Boc-4-trifluoromethyl-2,6-difluorobenzylamine (**29**) 167

^{13}C NMR of *N*-Boc-4-trifluoromethyl-2,6-difluorobenzylamine (**29**) 168

^{19}F NMR of *N*-Boc-4-trifluoromethyl-2,6-difluorobenzylamine (**29**) 169

^{1}H NMR of *N*-Boc-4-trifluoromethyl-2,6-dichlorobenzylamine (**30**) 170

^{13}C NMR of *N*-Boc-4-trifluoromethyl-2,6-dichlorobenzylamine (**30**) 171

^{19}F NMR of *N*-Boc-4-trifluoromethyl-2,6-dichlorobenzylamine (**30**) 172

^{1}H NMR of *N*-Boc-4-allyl-2,3,5,6-tetrafluorobenzylamine (**31a**) 173

^{13}C NMR of *N*-Boc-4-allyl-2,3,5,6-tetrafluorobenzylamine (**31a**) 174

^{19}F NMR of *N*-Boc-4-allyl-2,3,5,6-tetrafluorobenzylamine (**31a**) 175

^{1}H NMR of *N*-Boc-5-allyl-2,3,4,6-tetrafluorobenzylamine (**31b**) 176

^{13}C NMR of *N*-Boc-5-allyl-2,3,4,6-tetrafluorobenzylamine (**31b**)....177

^{19}F NMR of *N*-Boc-5-allyl-2,3,4,6-tetrafluorobenzylamine (**31b**)....178

^{1}H NMR of *N*-Boc-6-allyl-2,3,4,5-tetrafluorobenzylamine (**31c**)....179

^{13}C NMR of *N*-Boc-6-allyl-2,3,4,5-tetrafluorobenzylamine (**31c**)....180

^{19}F NMR of *N*-Boc-6-allyl-2,3,4,5-tetrafluorobenzylamine (**31c**)....181

^{1}H NMR of *N*-Boc-2,3,5,6-tetrafluoro-4-(pentafluorophenyl)benzylamine (**32a**)....182

^{13}C NMR of *N*-Boc-2,3,5,6-tetrafluoro-4-(pentafluorophenyl)benzylamine (**32a**)....183

^{19}F NMR of *N*-Boc-2,3,5,6-tetrafluoro-4-(pentafluorophenyl)benzylamine (**32a**)....184

^{1}H NMR of *N*-Boc-2,3,4,6-tetrafluoro-5-(pentafluorophenyl)benzylamine (**32b**)....185

^{13}C NMR of *N*-Boc-2,3,4,6-tetrafluoro-5-(pentafluorophenyl)benzylamine (**32b**)....186

^{19}F NMR of *N*-Boc-2,3,4,6-tetrafluoro-5-(pentafluorophenyl)benzylamine (**32b**)....187

^{1}H NMR of *N*-Boc-2,3,5,6-tetrafluoro-4-((1’R,2’S,5’R)-2’-isopropyl-5’-methylcyclohexylcarboxyl)benzylamine (**33a**)....188

^{13}C NMR of *N*-Boc-2,3,5,6-tetrafluoro-4-((1’R,2’S,5’R)-2’-isopropyl-5’-methylcyclohexylcarboxyl)benzylamine (**33a**)....189

^{19}F NMR of *N*-Boc-2,3,5,6-tetrafluoro-4-((1’R,2’S,5’R)-2’-isopropyl-5’-methylcyclohexylcarboxyl)benzylamine (**33a**)....190

^{1}H NMR of *N*-Boc-2,3,4,5-tetrafluoro-6-((1’R,2’S,5’R)-2’-isopropyl-5’-methylcyclohexylcarboxyl)benzylamine (**33b**)....191

^{13}C NMR of *N*-Boc-2,3,4,5-tetrafluoro-6-((1’R,2’S,5’R)-2’-isopropyl-5’-methylcyclohexylcarboxyl)benzylamine (**33b**)....192

^{19}F NMR of *N*-Boc-2,3,4,5-tetrafluoro-6-((1’R,2’S,5’R)-2’-isopropyl-5’-methylcyclohexylcarboxyl)benzylamine (**33b**)....193

^{1}H NMR of Cyclopentylmethyl-pentafluorobenzene (**34a**)....194

^{13}C NMR of Cyclopentylmethyl-pentafluorobenzene (**34a**)....195

^{19}F NMR of Cyclopentylmethyl-pentafluorobenzene (**34a**)....196

REFERENCES....197

MATERIALS AND METHODS

All reactions were carried out under an ambient atmosphere unless otherwise stated and monitored by thin-layer chromatography (TLC). Concentration under reduced pressure was performed by rotary evaporation at 25–40 °C at an appropriate pressure. Purified compounds were further dried under high vacuum (0.008–0.5 Torr). Yields refer to purified and spectroscopically pure compounds. A Kessil® A160WE LED was used as the light source. High-resolution mass spectra were obtained using *Q Exactive Plus* from *Thermo*.

Solvents

DMSO was purchased from Sigma-Aldrich dried with 3Å molecular sieves. Anhydrous solvents were obtained from Phoenix Solvent Drying Systems. All deuterated solvents were purchased from Euriso-Top®.

Chromatography

Thin layer chromatography (TLC) was performed using EMD TLC plates pre-coated with 250 μm thickness silica gel 60 F_{254} plates and visualized by fluorescence quenching under UV light. Flash chromatography was performed using silica gel (40–63 μm particle size) purchased from Geduran®. Preparatory high-performance liquid chromatographic separation was executed on a Shimadzu Prominence Preparative HPLC system.

Spectroscopy and Instruments

NMR spectra were recorded on a Bruker Ascend™ 500 spectrometer operating at 500 MHz, 125 MHz, and 470 MHz for ^{1}H, ^{13}C, and ^{19}F acquisitions, respectively; or on a Varian Unity/Inova 600 spectrometer operating at 600 MHz, 150 MHz, and 565 MHz for ^{1}H, ^{13}C, and ^{19}F acquisitions, respectively. Chemical shifts are reported in ppm with the solvent resonance as the internal standard. For ^{1}H NMR: $CDCl_3$, δ 7.26; $(CD_3)_2SO$, δ 2.50. For ^{13}C NMR: $CDCl_3$, δ 77.16; $(CD_3)_2SO$, δ 39.52. ^{19}F NMR spectra were referenced using a unified chemical shift scale based on the ^{1}H resonance of tetramethylsilane (1% (v/v) solution in the respective solvent). Data is reported as follows: s = singlet, d = doublet, t = triplet, q = quartet, m = multiplet, br = broad; coupling constants in Hz; integration.

Starting materials

All substrates and reagents were used as received from commercial suppliers, unless otherwise stated. Polyfluoroarenes were purchased from *Fluorochem Ltd.*

EXPERIMENTAL DATA

Representative procedure for decarboxylative polyfluoroarlyation

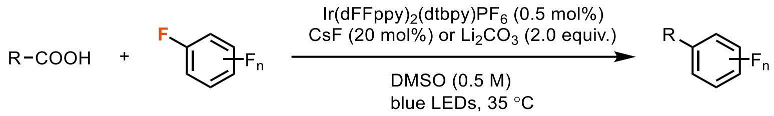

Under nitrogen atmosphere, to a 4-mL borosilicate vial equipped with a stir bar was added Ir(dFFppy)$_2$(dtbpy)PF$_6$ (1.5 mg, 1.5 µmol, 0.50 mol%), CsF (9.2 mg, 60 µmol, 20 mol%), or Li_2CO_3 (44.3 mg, 0.600 mmol, 2.00 equiv.), carboxylic acid (0.300 mmol, 1.00 equiv.), dry DMSO (0.6 mL, c = 0.5 M), and polyfluorobenzene (0.300 – 3.00 mmol, 1.00 – 10.0 equiv.). The vial was sealed with a septum-cap and placed 2 cm away from two 34 W blue LEDs. The temperature was kept at approximately 35 °C through cooling with a fan. After being stirred for 24 hours, the reaction mixture was poured into 20 mL of water, and the resulting mixture was extracted with EtOAc (3 × 20 mL). The combined organic phase was dried over $MgSO_4$, filtered through celite, and concentrated under vacuum. The residue was further purified by flash column chromatography on silica gel. Where necessary, further purification was accomplished by HPLC.

Reaction optimization for decarboxylative polyfluoroaryltion

Table S1. Photocatalyst optimization[a]

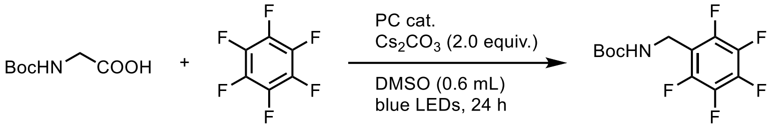

22

PC	yield
Ir[dF(CF_3)ppy]$_2$(dtbpy)PF_6 (0.5 mol%)	45%
Ir(dFFppy)$_2$(dtbpy)PF_6 (0.5 mol%)	52%
fac-Ir(ppy)$_3$ (0.5 mol%)	0%
Ru(bpz)$_2$(PF_6)$_2$ (1 mol%)	0%
Mes-Acr-Me•ClO_4 (5 mol%)	0%
4CzIPN (5 mol%)	0%
Riboflavin tetraacetate (RFT) (5 mol%)	trace

[a] Reaction conditions: *N*-Boc glycine (0.3 mmol), perfluorobenzene (0.6 mmol), Cs_2CO_3 (2.0 equiv.), DMSO (0.6 mL), 35 °C, blue LEDs, 24 h. Isolated yield.

Table S2. Base optimization[a]

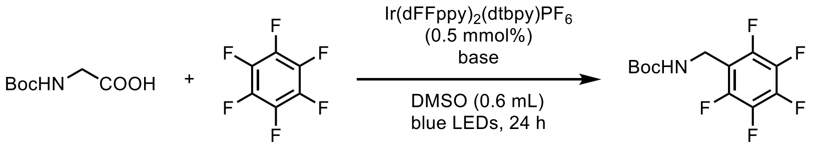

22

base	yield
Cs_2CO_3 (2.0 equiv.)	52%
K_2CO_3 (2.0 equiv.)	85%
Li_2CO_3 (2.0 equiv.)	93%
CsF (2.0 equiv.)	82%
CsF (20 mol%)	92%
Barton's base (20 mol%)	83%

[a] Reaction conditions: *N*-Boc glycine (0.3 mmol), perfluorobenzene (0.6 mmol), Ir(dFFppy)$_2$(dtbpy)PF_6 (0.5 mol%), DMSO (0.6 mL), 35 °C, blue LEDs, 24 h. Isolated yield.

Table S3. Solvent optimization[a]

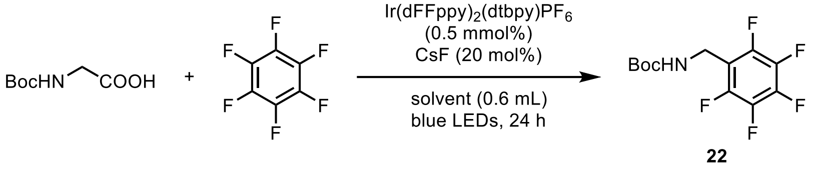

solvent	yield
DMSO	92%
DMF	72%
Acetone	9%[b]
MeCN	0%
Toluene	9%[b]
DCE	0%
MeOH	0%
DMSO[c]	70%

[a] Reaction conditions: *N*-Boc glycine (0.3 mmol), perfluorobenzene (0.6 mmol), 35 °C, Ir(dFFppy)$_2$(dtbpy)PF$_6$ (0.5 mol%), CsF (20 mol%), blue LEDs, 24 h. Isolated yield. [b] ^{19}F NMR yield with 4-fluoroanisole as internal standard. [c] H_2O (10 equiv.) was added, Li_2CO_3 (2.0 equiv.) instead of CsF (20 mol%).

Table S4. Control experiments[a]

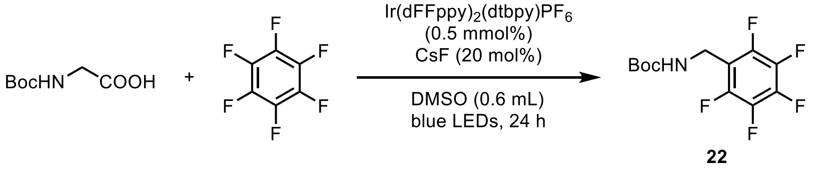

reaction conditions	yield
no photocatalyst	0%
no base	0%
no irradiation	0%

[a] Reaction conditions: *N*-Boc glycine (0.3 mmol), perfluorobenzene (0.6 mmol), Ir(dFFppy)$_2$(dtbpy)PF$_6$ (0.5 mol%), CsF (20 mol%), DMSO (0.6 mL), 35 °C, blue LEDs, 24 h.

Table S5. Amount of perfluorobenzene optimization with 2-methyl-3-phenylpropanoic acid[a]

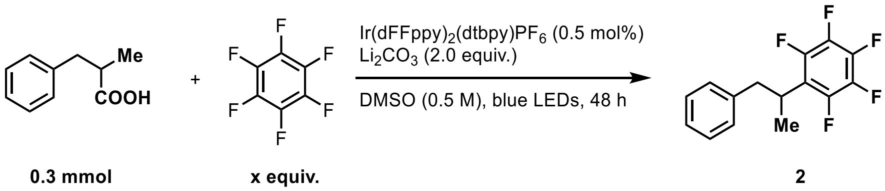

amount of perfluorobenzene	yield
2 equiv.	20%
5 equiv.	45%
10 equiv.	62%

[a] Reaction conditions: 2-methyl-3-phenylpropanoic acid (0.3 mmol), $Ir(dFFppy)_2(dtbpy)PF_6$ (0.5 mol%), Li_2CO_3 (2.0 equiv.), DMSO (0.6 mL), 35 °C, blue LEDs, 48 h. Isolated yield.

Table S6. Amount of perfluorobenzene optimization with 6-fluorochromane-2-carboxylic acid[a]

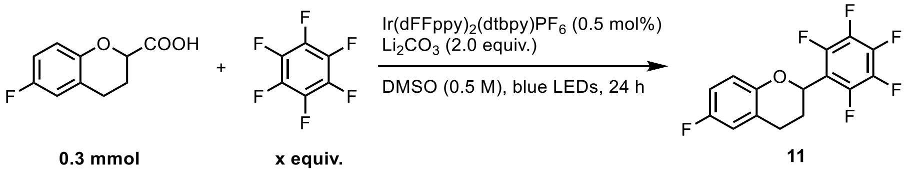

amount of perfluorobenzene	yield
2 equiv.	28%
5 equiv.	56%
10 equiv.	73%

[a] Reaction conditions: 6-fluorochromane-2-carboxylic acid (0.3 mmol), $Ir(dFFppy)_2(dtbpy)PF_6$ (0.5 mol%), Li_2CO_3 (2.0 equiv.), DMSO (0.6 mL), 35 °C, blue LEDs, 24 h. Isolated yield.

Table S7. List of unsuccessful substrates

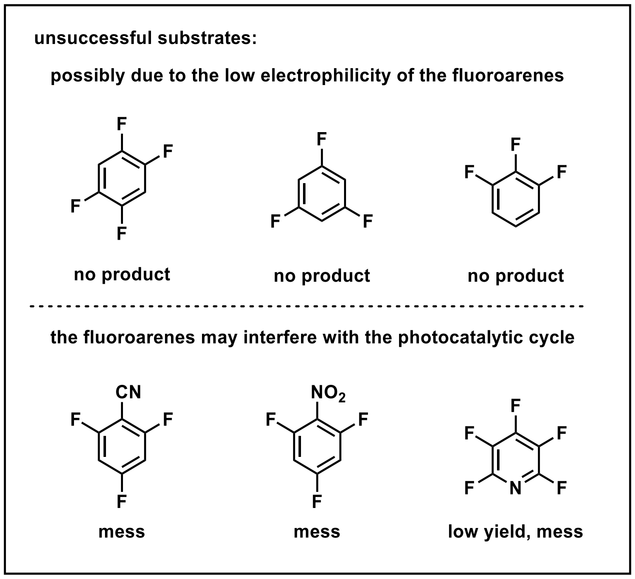

Preparation of substrates

(1R,2S,5R)-2-Isopropyl-5-methylcyclohexylpentafluorobenzoate (S1)

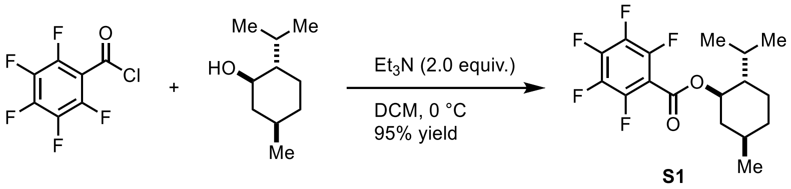

To a 50-mL round-bottom flask charged with (+)-menthol (2.34 g, 15.0 mmol, 1.00 equiv.) was added Et_3N (4.18 mL, 3.00 g, 30.0 mmol, 2.00 equiv.) and DCM (30 mL, c = 0.50 M). The resulting mixture was stirred at 0 °C in an ice-water bath for 5 min. Then, pentafluorobenzoyl chloride (3.46 g, 2.16 mL, 15.0 mmol, 1.00 equiv.) was added dropwise. After stirring at 0 °C for 6 hours, the reaction mixture was filtered through celite, and the filtrate was concentrated under vacuum. The residue was purified by flash column chromatography on silica gel, eluting with EtOAc/hexanes 1:40 (v/v) to afford 5 g (95%) of the title compound as a colorless liquid.

R_f = 0.30 (EtOAc/hexanes 1:40 (v/v)).

NMR Spectroscopy:

^{1}H NMR (500 MHz, $CDCl_3$, 23 ºC, δ): 4.98 (td, *J* = 10.9, 4.4 Hz, 1H), 2.19 – 2.11 (m, 1H), 2.01 – 1.91 (m, 1H), 1.78 – 1.68 (m, 2H), 1.59 – 1.44 (m, 2H), 1.18 – 1.04 (m, 2H), 0.95 (d, *J* = 6.6 Hz, 3H), 0.92 (d, *J* = 7.1 Hz, 3H), 0.91 – 0.89 (m, 1H), 0.80 (d, *J* = 7.0 Hz, 3H).

^{13}C NMR (125 MHz, $CDCl_3$, 23 ºC, δ): 158.8, 146.3 – 144.1 (m), 144.1 – 141.8 (m), 139.0 – 136.6 (m), 109.4 – 109.1 (m), 77.9, 47.0, 40.8, 34.2, 31.7, 26.1, 23.2, 22.1, 20.9, 16.0.

^{19}F NMR (470 MHz, $CDCl_3$, 23 ºC, δ): -138.9 – -139.1 (m, 2F), -149.7 – -149.9 (m, 1F), -160.5 – -160.7 (m, 2F).

HRMS-ESI(m/z) calc'd for $C_{17}H_{19}O_2F_5Na^+$ $[M+Na]^+$, 373.1197; found, 373.1195; deviation 0.78 ppm.

2-((1,3,5[10]-Estratrien-17-one-3-yl)oxy)propanoic acid (S2)

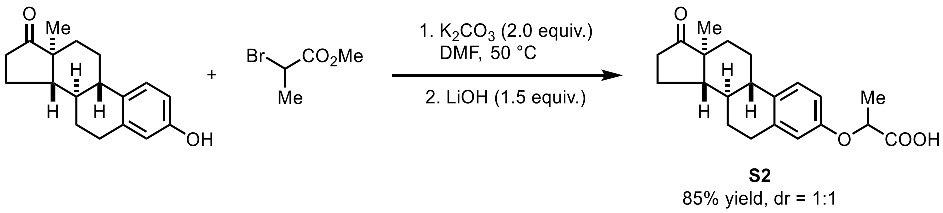

To a 50-mL round-bottom flask were added estrone (1.08 g, 4.00 mmol, 1.00 equiv.), methyl 2-bromopropanoate (835 mg, 557 µL, 5.00 mmol, 1.25 equiv.), K_2CO_3 (1.11 g, 8.00 mmol, 2.00 equiv.), and DMF (20.0 mL). The resulting mixture was stirred at 50 °C in a pre-heated oil bath for 15 hours. The resulting mixture was poured into water (100 mL), and the resultant precipitate was collected by filtration. The solid was purified by flash column chromatography on silica gel, eluting with EtOAc/hexanes 1:3 (v/v) to afford the corresponding methylpropanoate as a colorless solid. Then, the methylpropanoate was dissolved in dioxane (80 mL) in a 250-

mL round-bottom flask, followed by addition of aqueous 1M LiOH solution (6 mL). After stirring at room temperature for 8 hours, the reaction mixture was acidified to pH~3 with aqueous 10% HCl solution. Additional water (40 mL) was added, and the resulting organic phase was separated. The aqueous phase was extracted with EtOAc (3 × 100 mL). The combined organic phase was washed with brine (50 mL) and dried over $MgSO_4$. Removal of the solvent afforded 1.16 g (85% yield) of the title compound (a mixture of two diastereomers, dr = 1:1) as a colorless solid.

NMR Spectroscopy:

^{1}H NMR (500 MHz, $CDCl_3$, 23 ºC, δ): 7.19 (dd, *J* = 8.6, 1.0 Hz, 1H), 6.69 (dd, *J* = 8.6, 2.8 Hz, 1H), 6.65 (t, *J* = 3.3 Hz, 1H), 4.77 (q, *J* = 6.8 Hz, 1H), (4.76 (q, *J* = 6.8 Hz, 1H)), 2.91 – 2.84 (m, 2H), 2.55 – 2.46 (m, 1H), 2.41 – 2.33 (m, 1H), 2.27 – 2.19 (m, 1H), 2.20 – 2.10 (m, 1H), 2.09 – 1.95 (m, 2H), 1.97 – 1.90 (m, 1H), 1.64 (d, *J* = 6.8 Hz, 3H), 1.62 – 1.39 (m, 6H), 0.90 (s, 3H), (0.89 (s, 3H)).

^{13}C NMR (125 MHz, $CDCl_3$, 23 ºC, δ): 221.69, (221.67), 177.30, (177.27), 155.3, 138.20, (138.19), 133.44, (133.41), 126.6, 115.5, (115.4), 112.53, (112.48), 72.1, 50.5, 48.2, 44.1, 38.3, 36.0, 31.6, 29.71, (29.69), 26.6, 26.0, 21.7, 18.6, 13.9.

HRMS-ESI(m/z) calc'd for $C_{21}H_{25}O_4^-$ [M-H]$^-$, 341.1758; found, 341.1762; deviation 1.13 ppm.

Decarboxylative polyfluoroarylation of carboxylic acids

Nateglinide derivative 1

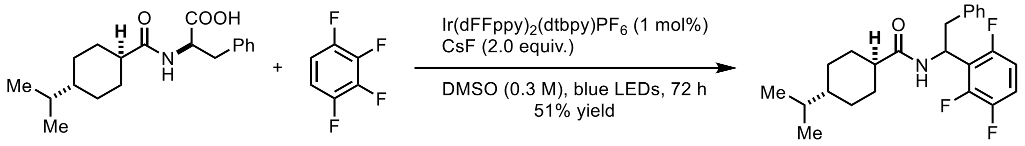

(±)-1

Under nitrogen atmosphere, to a 100-mL Schlenk tube equipped with a stir bar was added Ir(dFFppy)$_2$(dtbpy)PF$_6$ (60.0 mg, 60.0 µmol, 1.00 mol%), CsF (1.82 g, 12.0 mmol, 2.00 equiv.), nateglinide (1.91 g, 6.00 mmol, 1.00 equiv.), dry DMSO (20 mL, c = 0.30 M), and 1,2,3,4-tetrafluorobenzene (5.46 mL, 9.00 g, 60.0 mmol, 10.0 equiv.). The tube was sealed with a rubber plug and placed 2 cm away from two 34 W blue LEDs. The temperature was kept at approximately 35 °C through cooling with a fan. After being stirred for 72 hours, the reaction mixture was poured into 100 mL of water, and the resulting mixture was extracted with EtOAc (3 × 100 mL). The combined organic phase was dried over MgSO$_4$, filtered through celite, and concentrated under vacuum. The residue was further purified by flash column chromatography on silica gel, eluting with EtOAc/hexanes 1:10 (v/v) to afford 1.24 g (51% yield) of the title compound as a colorless solid.

R*f* = 0.20 (EtOAc/hexanes 1:10 (v/v)).

NMR Spectroscopy:

^{1}H NMR (500 MHz, CDCl$_3$, 23 °C, δ): 7.25 – 7.16 (m, 3H), 7.12 – 7.07 (m, 2H), 7.04 – 6.94 (m, 1H), 6.79 – 6.70 (m, 1H), 6.13 (d, *J* = 8.9 Hz, 1H), 5.80 – 5.71 (m, 1H), 3.15 (dd, *J* = 13.6, 7.8 Hz, 1H), 3.08 (dd, *J* = 13.6, 8.0 Hz, 1H), 1.96 (tt, *J* = 12.2, 3.5 Hz, 1H), 1.86 – 1.71 (m, 4H), 1.46 – 1.24 (m, 3H), 1.08 – 0.91 (m, 3H), 0.85 (s, 3H), 0.83 (s, 3H).

^{13}C NMR (125 MHz, CDCl$_3$, 23 °C, δ): 175.4, 157.3 – 155.1 (m), 148.7 (ddd, *J* = 249.2, 14.4, 8.9 Hz), 148.4 – 146.0 (m), 136.6, 129.1, 128.5, 127.0, 119.5 (dd, *J* = 19.1, 13.8 Hz), 115.8 (dd, *J* = 19.3, 10.6 Hz), 111.0 (ddd, *J* = 25.2, 6.5, 4.0 Hz), 45.7, 45.6, 43.3, 41.0, 32.8, 29.7, 29.6, 29.0, 29.0, 19.8, 19.8.

^{19}F NMR (470 MHz, CDCl$_3$, 23 °C, δ): -120.0 – -120.1 (m, 1F), -138.1 (dd, *J* = 20.7, 8.8 Hz, 1F), -141.7 – -142.1 (m, 1F).

HRMS-ESI(m/z) calc'd for $C_{24}H_{28}NOF_3Na^+$ $[M+Na]^+$, 426.2015; found, 426.2013; deviation 0.63 ppm.

Pentafluoro-(1-phenylpropan-2-yl)benzene (2)

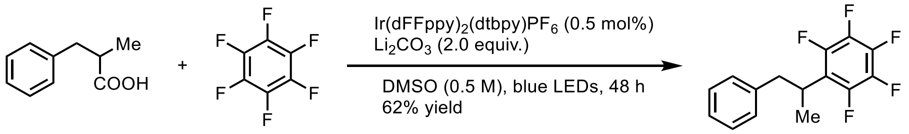

(±)-2

Under nitrogen atmosphere, to a 4-mL borosilicate vial equipped with a stir bar was added Ir(dFFppy)$_2$(dtbpy)PF$_6$ (1.5 mg, 1.5 µmol, 0.50 mol%), Li$_2$CO$_3$ (44.3 mg, 0.600 mmol, 2.00 equiv.), 2-methyl-3-

phenylpropanoic acid (49.3 mg, 0.300 mmol, 1.00 equiv.), dry DMSO (0.6 mL, c = 0.5 M), and perfluorobenzene (347 µL, 558 mg, 3.00 mmol, 10.0 equiv.). The vial was sealed with a septum-cap and placed 2 cm away from two 34 W blue LEDs. The temperature was kept at approximately 35 °C through cooling with a fan. After being stirred for 48 hours, the reaction mixture was poured into 20 mL of water, and the resulting mixture was extracted with EtOAc (3 × 20 mL). The combined organic phase was dried over $MgSO_4$, filtered through celite, and concentrated under vacuum. The residue was further purified by flash column chromatography on silica gel, eluting with hexanes to afford 53.4 mg (62% yield) of the title compound as a colorless liquid.

R*f* = 0.60 (hexanes).

NMR Spectroscopy:

^{1}H NMR (500 MHz, $CDCl_3$, 23 ºC, δ): 7.27 – 7.23 (m, 2H), 7.21 – 7.15 (m, 1H), 7.14 – 7.09 (m, 2H), 3.60 – 3.48 (m, 1H), 3.07 – 2.96 (m, 2H), 1.41 (d, *J* = 7.1 Hz, 3H).

^{13}C NMR (151 MHz, $CDCl_3$, 23 ºC, δ): 146.2 – 144.3 (m), 139.6, 140.5 – 138.5 (m), 138.6 – 136.6 (m), 128.7, 128.6, 126.6, 119.1 – 118.7 (m), 41.5 (t, *J* = 2.3 Hz), 32.7 – 32.6 (m), 19.2 (t, *J* = 2.3 Hz).

^{19}F NMR (470 MHz, $CDCl_3$, 23 ºC, δ): -142.8 (dd, *J* = 22.5, 8.3 Hz, 2F), -157.7 – -157.8 (m, 1F), -162.8 – -163.0 (m, 2F).

HRMS-EI(m/z) calc'd for $C_{15}H_{11}F_5$ $[M]^+$, 286.0775; found, 286.0775; deviation 0.01 ppm.

Cyclopentylpentafluorobenzene (3)

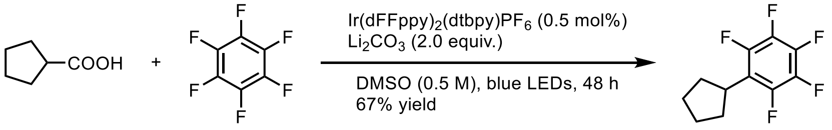

3

Under nitrogen atmosphere, to a 4-mL borosilicate vial equipped with a stir bar was added $Ir(dFFppy)_2(dtbpy)PF_6$ (1.5 mg, 1.5 µmol, 0.50 mol%), Li_2CO_3 (44.3 mg, 0.600 mmol, 2.00 equiv.), cyclopentanecarboxylic acid (34.3 mg, 0.300 mmol, 1.00 equiv.), dry DMSO (0.6 mL, c = 0.5 M), and perfluorobenzene (347 µL, 558 mg, 3.00 mmol, 10.0 equiv.). The vial was sealed with a septum-cap and placed 2 cm away from two 34 W blue LEDs. The temperature was kept at approximately 35 °C through cooling with a fan. After being stirred for 48 hours, the reaction mixture was directly purified by flash column chromatography on silica gel, eluting with pentane to afford 47.8 mg (67% yield) of the title compound as a colorless liquid.

R*f* = 0.80 (hexanes).

NMR Spectroscopy:

^{1}H NMR (500 MHz, $CDCl_3$, 23 ºC, δ): 3.34 (p, *J* = 9.0 Hz, 1H), 2.06 – 1.94 (m, 2H), 1.93 – 1.83 (m, 2H), 1.84 – 1.74 (m, 2H), 1.73 – 1.62 (m, 2H).

^{13}C NMR (150 MHz, $CDCl_3$, 23 ºC, δ): 146.3 – 144.4 (m), 140.3 – 138.5 (m), 138.5 – 136.7 (m), 119.4 – 119.1 (m), 34.5 – 34.5 (m), 32.3, 26.4.

^{19}F NMR (470 MHz, $CDCl_3$, 23 ºC, δ): -143.2 (dd, *J* = 22.1, 7.8 Hz, 2F), -158.9 (t, *J* = 21.2 Hz, 1F), -163.3 – -163.4 (m, 2F).

HRMS-EI(m/z) calc'd for $C_{11}H_9F_5$ $[M]^+$, 236.0619; found, 236.0622; deviation 1.13 ppm.

N-Benzoyl-4-perfluorophenylpiperidine (4)

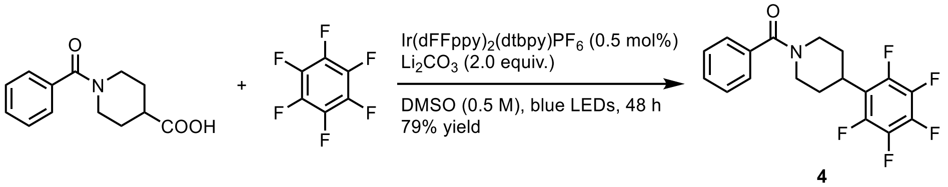

4

Under nitrogen atmosphere, to a 4-mL borosilicate vial equipped with a stir bar was added Ir(dFFppy)$_2$(dtbpy)PF_6 (1.5 mg, 1.5 µmol, 0.50 mol%), Li_2CO_3 (44.3 mg, 0.600 mmol, 2.00 equiv.), *N*-benzoylpiperidine-4-carboxylic acid (70.0 mg, 0.300 mmol, 1.00 equiv.), dry DMSO (0.6 mL, c = 0.5 M), and perfluorobenzene (347 µL, 558 mg, 3.00 mmol, 10.0 equiv.). The vial was sealed with a septum-cap and placed 2 cm away from two 34 W blue LEDs. The temperature was kept at approximately 35 °C through cooling with a fan. After being stirred for 48 hours, the reaction mixture was poured into 20 mL of water, and the resulting mixture was extracted with EtOAc (3 × 20 mL). The combined organic phase was dried over $MgSO_4$, filtered through celite, and concentrated under vacuum. The residue was further purified by flash column chromatography on silica gel, eluting with EtOAc/hexanes 1:3 (v/v) to afford 84.2 mg (79% yield) of the title compound as a colorless solid.

R_f = 0.20 (EtOAc/hexanes 1:3 (v/v)).

NMR Spectroscopy:

^{1}H NMR (500 MHz, DMSO-d_6, 100 ºC, δ): 7.46 – 7.43 (m, 3H), 7.41 – 7.39 (m, 2H), 4.20 (brs, 2H), 3.33 (tt, *J* = 12.1, 4.0 Hz, 1H), 3.11 – 3.00 (m, 2H), 1.96 – 1.86 (m, 2H), 1.85 – 1.80 (m, 2H).

^{13}C NMR (125 MHz, DMSO-d_6, 100 ºC, δ): 168.8, 145.6 – 143.2 (m), 139.9 – 137.7 (m), 137.7 – 135.5 (m), 135.9, 128.7, 127.8, 126.0, 117.5 – 117.2 (m), 44.3, 32.7, 29.4.

^{19}F NMR (470 MHz, DMSO-d_6, 100 ºC, δ): -142.7 – -142.8 (m, 2F), -158.2 (t, *J* = 21.4 Hz, 1F), -163.2 (td, *J* = 22.2, 7.3 Hz, 2F).

HRMS-CI(m/z) calc'd for $C_{18}H_{13}F_5NO^+$ $[M-H]^+$, 354.0912; found, 354.0919; deviation 2.00 ppm.

Indomethacin derivative 5

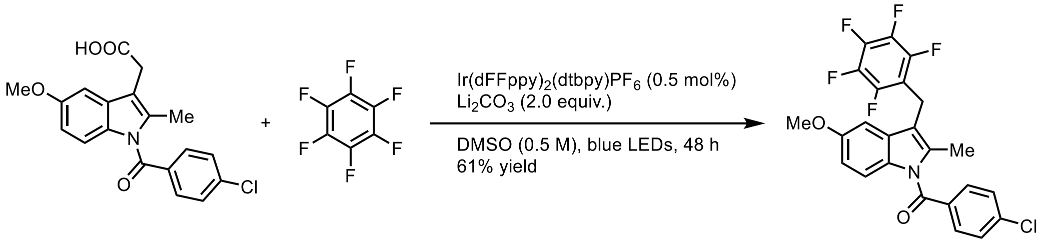

5

Under nitrogen atmosphere, to a 4-mL borosilicate vial equipped with a stir bar was added Ir(dFFppy)$_2$(dtbpy)PF$_6$ (1.5 mg, 1.5 µmol, 0.50 mol%), Li_2CO_3 (44.3 mg, 0.600 mmol, 2.00 equiv.), indomethacin (108 mg, 0.300 mmol, 1.00 equiv.), dry DMSO (0.6 mL, c = 0.5 M), and perfluorobenzene (347 µL, 558 mg, 3.00 mmol, 10.0 equiv.). The vial was sealed with a septum-cap and placed 2 cm away from two 34 W blue LEDs. The temperature was kept at approximately 35 °C through cooling with a fan. After being stirred for 48 hours, the reaction mixture was poured into 20 mL of water, and the resulting mixture was extracted with EtOAc (3 × 20 mL). The combined organic phase was dried over $MgSO_4$, filtered through celite, and concentrated under vacuum. The residue was further purified by flash column chromatography on silica gel, eluting with EtOAc/hexanes 1:10 (v/v) to afford 87.8 mg (61% yield) of the title compound as a colorless solid.

R*f* = 0.40 (EtOAc/hexanes 1:10 (v/v)).

NMR Spectroscopy:

^{1}H NMR (500 MHz, $CDCl_3$, 23 ºC, δ): 7.69 – 7.62 (m, 2H), 7.50 – 7.44 (m, 2H), 6.97 (d, *J* = 2.5 Hz, 1H), 6.86 (d, *J* = 9.0 Hz, 1H), 6.66 (dd, *J* = 9.0, 2.5 Hz, 1H), 4.05 (d, *J* = 1.6 Hz, 2H), 3.83 (s, 3H), 2.47 (s, 3H).

^{13}C NMR (125 MHz, $CDCl_3$, 23 ºC, δ): 168.4, 156.1, 146.5 – 144.2 (m), 139.5, 141.3 – 138.9 (m), 138.9 – 136.5 (m), 135.7, 134.0, 131.3, 130.9, 130.1, 129.3, 115.0, 114.8, 113.5 – 113.1 (m), 111.7, 100.8 (t, *J* = 3.4 Hz), 55.6, 17.6, 13.4 – 13.2 (m).

^{19}F NMR (470 MHz, $CDCl_3$, 23 ºC, δ): -142.1 (dd, *J* = 22.5, 7.9 Hz, 2F), -156.8 (t, *J* = 20.8 Hz, 1F), -162.2 (td, *J* = 21.9, 7.9 Hz, 2F).

HRMS-EI(m/z) calc'd for $C_{24}H_{15}ClF_5NO_2^+$ [M]$^+$, 479.0706; found, 479.0708; deviation 0.48 ppm.

4-(Perfluorophenyl)tetrahydropyran (6)

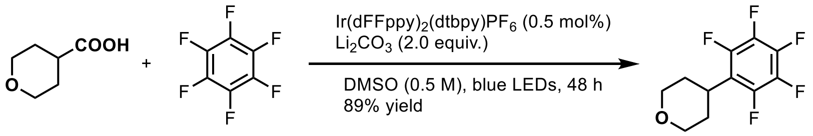

6

Under nitrogen atmosphere, to a 4-mL borosilicate vial equipped with a stir bar was added Ir(dFFppy)$_2$(dtbpy)PF$_6$ (1.5 mg, 1.5 µmol, 0.50 mol%), Li_2CO_3 (44.3 mg, 0.600 mmol, 2.00 equiv.),

tetrahydropyran-4-carboxylic acid (39.1 mg, 0.300 mmol, 1.00 equiv.), dry DMSO (0.6 mL, c = 0.5 M), and perfluorobenzene (347 µL, 558 mg, 3.00 mmol, 10.0 equiv.). The vial was sealed with a septum-cap and placed 2 cm away from two 34 W blue LEDs. The temperature was kept at approximately 35 °C through cooling with a fan. After being stirred for 48 hours, the reaction mixture was poured into 20 mL of water, and the resulting mixture was extracted with EtOAc (3 × 20 mL). The combined organic phase was dried over $MgSO_4$, filtered through celite, and concentrated under vacuum. The residue was further purified by flash column chromatography on silica gel, eluting with EtOAc/hexanes 1:40 (v/v) to afford 67.1 mg (89% yield) of the title compound as a colorless liquid.

R*f* = 0.20 (EtOAc/hexanes 1:40 (v/v)).

NMR Spectroscopy:

¹H NMR (500 MHz, $CDCl_3$, 23 ºC, δ): 4.11 – 4.04 (m, 2H), 3.54 – 3.46 (m, 2H), 3.24 (tt, *J* = 12.5, 3.7 Hz, 1H), 2.24 – 2.11 (m, 2H), 1.67 – 1.59 (m, 2H).

¹³C NMR (150 MHz, $CDCl_3$, 23 ºC, δ): 146.3 – 144.3 (m), 139.8 (dtt, *J* = 252.3, 13.5, 5.3 Hz), 138.8 – 136.8 (m), 117.9 (tdt, *J* = 16.1, 3.6, 1.7 Hz), 68.4, 32.6 – 32.5 (m), 30.8 (t, *J* = 3.0 Hz)

¹⁹F NMR (470 MHz, $CDCl_3$, 23 ºC, δ): -143.0 (dd, *J* = 22.5, 8.2 Hz, 2F), -157.3 – -157.5 (m, 1F), -162.4 (td, *J* = 21.0, 7.6 Hz, 2F).

HRMS-EI(m/z) calc'd for $C_{11}H_9F_5O$ $[M]^+$, 252.0568; found, 252.0569; deviation 0.17 ppm.

N-Boc-2-perfluoropehnylpyrrolidine (7)

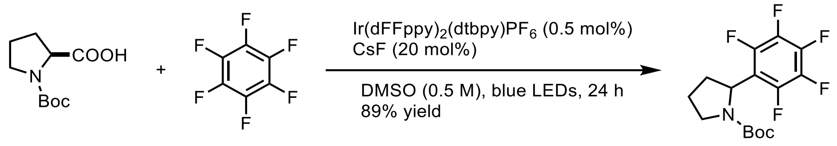

(±)-7

Under nitrogen atmosphere, to a 4-mL borosilicate vial equipped with a stir bar was added Ir(dFFppy)$_2$(dtbpy)PF_6 (1.5 mg, 1.5 µmol, 0.50 mol%), CsF (9.2 mg, 60 µmol, 20 mol%), *N*-Boc-*L*-proline (64.6 mg, 0.300 mmol, 1.00 equiv.), dry DMSO (0.6 mL, c = 0.5 M), and perfluorobenzene (174 µL, 279 mg, 1.50 mmol, 5.00 equiv.). The vial was sealed with a septum-cap and placed 2 cm away from two 34 W blue LEDs. The temperature was kept at approximately 35 °C through cooling with a fan. After being stirred for 24 hours, the reaction mixture was poured into 20 mL of water, and the resulting mixture was extracted with EtOAc (3 × 20 mL). The combined organic phase was dried over $MgSO_4$, filtered through celite, and concentrated under vacuum. The residue was further purified by flash column chromatography on silica gel, eluting with EtOAc/hexanes 1:10 (v/v) to afford 90.0 mg (89% yield) of the title compound as a colorless liquid.

R*f* = 0.20 (EtOAc/hexanes 1:10 (v/v)).

NMR Spectroscopy:

¹H NMR (500 MHz, DMSO-*d*$_6$, 80 ºC, δ): 5.08 – 5.00 (m, 1H), 3.61 – 3.53 (m, 1H), 3.42 – 3.33 (m, 1H),

2.46 – 2.39 (m, 1H), 2.03 – 1.86 (m, 3H), 1.26 (s, 9H).

^{13}C NMR (125 MHz, DMSO-*d*$_6$, 80 ºC, δ): 152.5, 145.0 – 142.8 (m), 140.0 – 137.7 (m), 137.7 – 135.4 (m), 117.8 – 117.4 (m), 78.5, 51.2, 46.1, 32.3, 27.4.

^{19}F NMR (470 MHz, DMSO-*d*$_6$, 80 ºC, δ): -144.7 – -146.1 (m, 2F), -157.7 – -158.3 (m, 1F), -163.9 (td, *J* = 22.1, 7.4 Hz, 2F).

HRMS-ESI(m/z) calc'd for $C_{15}H_{16}NO_2F_5Na^+$ [M+Na]$^+$, 360.0993; found, 360.0994; deviation 0.08 ppm.

N-Cbz-1-hydroxyl-2-perfluoropehnylethan-2-amine (8)

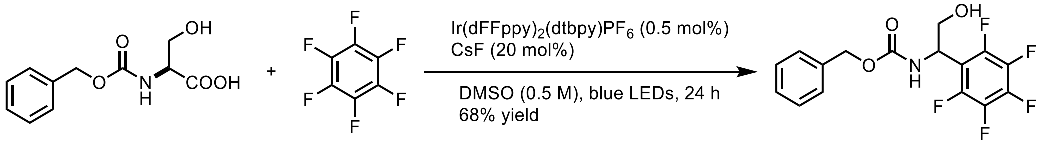

Under nitrogen atmosphere, to a 4-mL borosilicate vial equipped with a stir bar was added Ir(dFFppy)$_2$(dtbpy)PF$_6$ (1.5 mg, 1.5 µmol, 0.50 mol%), CsF (9.2 mg, 60 µmol, 20 mol%), *N*-Cbz-*L*-serine (71.8 mg, 0.300 mmol, 1.00 equiv.), dry DMSO (0.6 mL, c = 0.5 M), and perfluorobenzene (174 µL, 279 mg, 1.50 mmol, 5.00 equiv.). The vial was sealed with a septum-cap and placed 2 cm away from two 34 W blue LEDs. The temperature was kept at approximately 35 °C through cooling with a fan. After being stirred for 24 hours, the reaction mixture was poured into 20 mL of water, and the resulting mixture was extracted with EtOAc (3 × 20 mL). The combined organic phase was dried over $MgSO_4$, filtered through celite, and concentrated under vacuum. The residue was further purified by flash column chromatography on silica gel, eluting with EtOAc/hexanes 1:2 (v/v) to afford 73.7 mg (68% yield) of the title compound as a colorless solid.

R*f* = 0.30 (EtOAc/hexanes 1:2 (v/v)).

NMR Spectroscopy:

^{1}H NMR (500 MHz, CDCl$_3$, 23 ºC, δ): 7.39 – 7.29 (m, 5H), 5.59 (d, *J* = 9.2 Hz, 1H), 5.39 – 5.31 (m, 1H), 5.16 – 5.04 (m, 2H), 3.94 – 3.81 (m, 2H), 2.08 (brs, 1H).

^{13}C NMR (125 MHz, CDCl$_3$, 23 ºC, δ): 156.3, 146.5 – 143.8 (m), 142.3 – 139.6 (m), 139.2 – 136.5 (m), 135.9, 128.7, 128.5, 128.3, 113.1 – 112.7 (m), 67.6, 64.1, 49.0.

^{19}F NMR (470 MHz, CDCl$_3$, 23 ºC, δ): -142.8 (dd, *J* = 21.8, 7.7 Hz, 2F), -154.2 (t, *J* = 21.0 Hz, 1F), -161.1 – -161.3 (m, 2F).

HRMS-ESI(m/z) calc'd for $C_{16}H_{12}NO_3F_5Na^+$ [M+Na]$^+$, 384.0630; found, 384.0631; deviation 0.35 ppm.

MTV III derivative 9

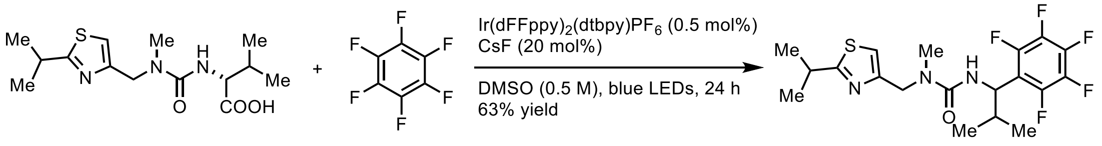

(±)-9

Under nitrogen atmosphere, to a 4-mL borosilicate vial equipped with a stir bar was added Ir(dFFppy)$_2$(dtbpy)PF$_6$ (1.5 mg, 1.5 µmol, 0.50 mol%), CsF (9.2 mg, 60 µmol, 20 mol%), MTV III (94.0 mg, 0.300 mmol, 1.00 equiv.), dry DMSO (0.6 mL, c = 0.5 M), and perfluorobenzene (174 µL, 279 mg, 1.50 mmol, 5.00 equiv.). The vial was sealed with a septum-cap and placed 2 cm away from two 34 W blue LEDs. The temperature was kept at approximately 35 °C through cooling with a fan. After being stirred for 24 hours, the reaction mixture was poured into 20 mL of water, and the resulting mixture was extracted with EtOAc (3 × 20 mL). The combined organic phase was dried over MgSO$_4$, filtered through celite, and concentrated under vacuum. The residue was further purified by flash column chromatography on silica gel, eluting with EtOAc/hexanes 1:2 (v/v) to afford 82.3 mg (63% yield) of the title compound as a colorless solid.

R*f* = 0.23 (EtOAc/hexanes 1:2 (v/v)).

NMR Spectroscopy:

^{1}H NMR (500 MHz, CDCl$_3$, 23 ºC, δ): 6.95 (s, 1H), 6.54 (d, *J* = 7.2 Hz, 1H), 5.00 – 4.93 (m, 1H), 4.42 – 4.31 (m, 2H), 3.30 (hept, *J* = 6.9 Hz, 1H), 2.94 (s, 3H), 2.21 – 2.09 (m, 1H), 1.39 (d, *J* = 2.3 Hz, 3H), 1.38 (d, *J* = 2.3 Hz, 3H), 1.10 (d, *J* = 6.6 Hz, 3H), 0.84 (d, *J* = 6.8 Hz, 3H).

^{13}C NMR (125 MHz, CDCl$_3$, 23 ºC, δ): 179.4, 158.4, 152.1, 145.9 – 144.0 (m), 141.1 – 139.1 (m), 138.5 – 136.5 (m), 117.3 – 116.9 (m), 114.4, 52.8, 49.3, 35.0, 33.4, 32.5, 23.3, 23.2, 20.1, 19.8.

^{19}F NMR (470 MHz, CDCl$_3$, 23 ºC, δ): -142.8 (dd, *J* = 22.7, 7.8 Hz, 2F), -156.9 (t, *J* = 20.8 Hz, 1F), -162.6 (td, *J* = 22.2, 8.1 Hz, 2F).

HRMS-ESI(m/z) calc'd for $C_{19}H_{22}N_3OSF_5Na^+$ [M+Na]$^+$, 458.1296; found, 458.1296; deviation 0.01 ppm.

1-(Perfluorophenyl)ethanol (10)

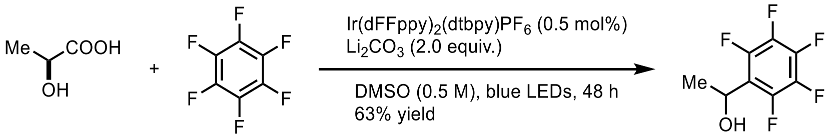

(±)-10

Under nitrogen atmosphere, to a 4-mL borosilicate vial equipped with a stir bar was added Ir(dFFppy)$_2$(dtbpy)PF$_6$ (1.5 mg, 1.5 µmol, 0.50 mol%), Li_2CO_3 (44.3 mg, 0.600 mmol, 2.00 equiv.), *L*-lactic acid (27.0 mg, 0.300 mmol, 1.00 equiv.), dry DMSO (0.6 mL, c = 0.5 M), and perfluorobenzene (347 µL, 558 mg, 3.00 mmol, 10.0 equiv.). The vial was sealed with a septum-cap and placed 2 cm away from two 34 W blue LEDs. The temperature was kept at approximately 35 °C through cooling with a fan. After being stirred for 48 hours, the reaction mixture was directly purified by flash column chromatography on silica gel, eluting with

EtOAc/hexanes 1:10 (v/v) to afford 40.0 mg (63% yield) of the title compound as a colorless liquid.

R*f* = 0.30 (EtOAc/hexanes 1:10 (v/v)).

NMR Spectroscopy:

^{1}H NMR (500 MHz, $CDCl_3$, 23 ºC, δ): 5.26 (q, *J* = 6.8 Hz, 1H), 2.24 (brs, 1H), 1.65 (d, *J* = 6.7 Hz, 3H).

^{13}C NMR (150 MHz, $CDCl_3$, 23 ºC, δ): 145.8 – 143.8 (m), 141.7 – 139.6 (m), 138.7 – 136.7 (m), 118.3 – 117.9 (m), 62.5, 23.4.

^{19}F NMR (470 MHz, $CDCl_3$, 23 ºC, δ): -144.7 (dd, *J* = 22.2, 8.2 Hz, 2H), -155.6 (t, *J* = 20.8 Hz, 1H), -161.9 (td, *J* = 21.9, 8.1 Hz, 2H).

HRMS-EI(m/z) calc'd for $C_8H_5OF_5$ $[M]^+$, 212.0255; found, 212.0255; deviation 0.11 ppm.

These NMR spectroscopic data correspond to those of authentic sample reported in the literature.[1]

6-Fluorochromane-2-pentafluorobenzene (11)

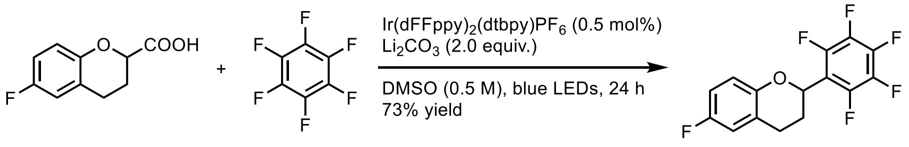

(±)-11

Under nitrogen atmosphere, to a 4-mL borosilicate vial equipped with a stir bar was added $Ir(dFFppy)_2(dtbpy)PF_6$ (1.5 mg, 1.5 µmol, 0.50 mol%), Li_2CO_3 (44.3 mg, 0.600 mmol, 2.00 equiv.), 6-fluorochromane-2-carboxylic acid (58.9 mg, 0.300 mmol, 1.00 equiv.), dry DMSO (0.6 mL, c = 0.5 M), and perfluorobenzene (347 µL, 558 mg, 3.00 mmol, 10.0 equiv.). The vial was sealed with a septum-cap and placed 2 cm away from two 34 W blue LEDs. The temperature was kept at approximately 35 °C through cooling with a fan. After being stirred for 24 hours, the reaction mixture was poured into 20 mL of water, and the resulting mixture was extracted with EtOAc (3 × 20 mL). The combined organic phase was dried over $MgSO_4$, filtered through celite, and concentrated under vacuum. The residue was further purified by flash column chromatography on silica gel, eluting with hexanes to afford 69.7 mg (73% yield) of the title compound as a colorless solid.

R*f* = 0.20 (hexanes).

NMR Spectroscopy:

^{1}H NMR (500 MHz, $CDCl_3$, 23 ºC, δ): 6.87 – 6.79 (m, 2H), 6.79 – 6.73 (m, 1H), 5.38 (dd, *J* = 11.8, 2.3 Hz, 1H), 3.09 – 2.99 (m, 1H), 2.91 – 2.83 (m, 1H), 2.55 – 2.43 (m, 1H), 2.19 – 2.09 (m, 1H).

^{13}C NMR (125 MHz, $CDCl_3$, 23 ºC, δ): 157.2 (d, *J* = 238.9 Hz), 150.5 (d, *J* = 2.3 Hz), 146.5 – 144.3 (m), 142.5 – 140.2 (m), 139.1 – 136.7 (m), 122.5 (d, *J* = 7.7 Hz), 117.9 (d, *J* = 8.0 Hz), 115.5 (d, *J* = 22.7 Hz), 114.4 (d, *J* = 23.1 Hz), 69.7, 26.5, 25.7. (One carbon signal was missing because of overlap.)

^{19}F NMR (470 MHz, $CDCl_3$, 23 ºC, δ): -123.5 (td, *J* = 8.2, 4.8 Hz, 1F), -142.1 – -142.2 (m, 2F), -153.9 (t,

J = 20.8 Hz, 1F), -161.2 – -162.0 (m, 2F).

HRMS-EI(m/z) calc'd for $C_{15}H_8F_6O^+$ $[M]^+$, 318.0474; found, 318.0478; deviation 1.30 ppm.

2,2-Dimethyl-4-(perfluorophenyl)-1,3-dioxolane (12)

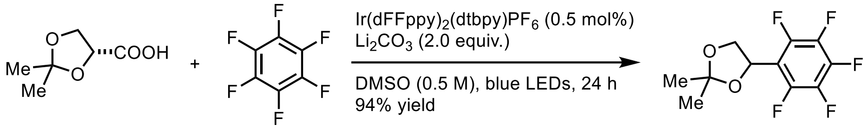

Under nitrogen atmosphere, to a 4-mL borosilicate vial equipped with a stir bar was added Ir(dFFppy)$_2$(dtbpy)PF$_6$ (1.5 mg, 1.5 µmol, 0.50 mol%), Li_2CO_3 (44.3 mg, 0.600 mmol, 2.00 equiv.), *α,β*-isopropylidene-*D*-glyceric acid (43.8 mg, 0.300 mmol, 1.00 equiv.), dry DMSO (0.6 mL, c = 0.5 M), and perfluorobenzene (347 µL, 558 mg, 3.00 mmol, 10.0 equiv.). The vial was sealed with a septum-cap and placed 2 cm away from two 34 W blue LEDs. The temperature was kept at approximately 35 °C through cooling with a fan. After being stirred for 24 hours, the reaction mixture was poured into 20 mL of water, and the resulting mixture was extracted with EtOAc (3 × 20 mL). The combined organic phase was dried over $MgSO_4$, filtered through celite, and concentrated under vacuum. The residue was further purified by flash column chromatography on silica gel, eluting with EtOAc/hexanes 1:40 (v/v) to afford 75.6 mg (94% yield) of the title compound as a colorless liquid.

R_f = 0.20 (EtOAc/hexanes 1:40 (v/v)).

NMR Spectroscopy:

^{1}H NMR (500 MHz, $CDCl_3$, 23 ºC, δ): 5.38 (t, J = 7.2 Hz, 1H), 4.28 (dd, J = 8.2, 6.7 Hz, 1H), 4.09 – 4.02 (m, 1H), 1.53 (s, 3H), 1.45 (s, 3H).

^{13}C NMR (125 MHz, $CDCl_3$, 23 ºC, δ): 146.8 – 144.6 (m), 142.6 – 140.1 (m), 139.0 – 136.6 (m), 112.3 – 112.0 (m), 111.0, 68.6 – 68.5 (m), 68.5 – 68.4 (m), 26.1, 25.5.

^{19}F NMR (470 MHz, $CDCl_3$, 23 ºC, δ): -142.1 – -142.2 (m, 2F), -153.5 – -153.7 (m, 1F), -161.7 – -161.9 (m, 2F).

HRMS-CI(m/z) calc'd for $C_{11}H_{10}F_5O_2^+$ $[M+H]^+$, 269.0595; found, 269.0597; deviation 0.53 ppm.

Bezafibrate derivative 13

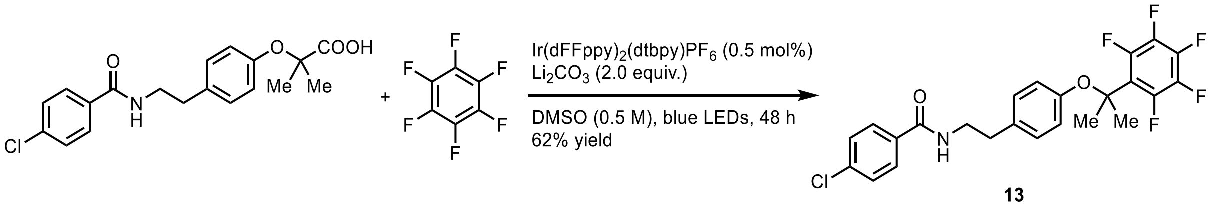

Under nitrogen atmosphere, to a 4-mL borosilicate vial equipped with a stir bar was added Ir(dFFppy)$_2$(dtbpy)PF$_6$ (1.5 mg, 1.5 µmol, 0.50 mol%), Li_2CO_3 (44.3 mg, 0.600 mmol, 2.00 equiv.), bezafibrate

(109 mg, 0.300 mmol, 1.00 equiv.), dry DMSO (0.6 mL, c = 0.5 M), and perfluorobenzene (347 µL, 558 mg, 3.00 mmol, 10.0 equiv.). The vial was sealed with a septum-cap and placed 2 cm away from two 34 W blue LEDs. The temperature was kept at approximately 35 °C through cooling with a fan. After being stirred for 48 hours, the reaction mixture was poured into 20 mL of water, and the resulting mixture was extracted with EtOAc (3 × 20 mL). The combined organic phase was dried over $MgSO_4$, filtered through celite, and concentrated under vacuum. The residue was further purified by flash column chromatography on silica gel, eluting with EtOAc/hexanes 1:5 (v/v) to afford 90.0 mg (62% yield) of the title compound as a colorless solid.

R*f* = 0.20 (EtOAc/hexanes 1:5 (v/v)).

NMR Spectroscopy:

^{1}H NMR (500 MHz, $CDCl_3$, 23 ºC, δ): 7.63 – 7.57 (m, 2H), 7.40 – 7.31 (m, 2H), 7.05 – 6.98 (m, 2H), 6.74 – 6.64 (m, 2H), 6.24 (t, *J* = 5.8 Hz, 1H), 3.62 (td, *J* = 7.0, 5.7 Hz, 2H), 2.82 (t, *J* = 7.0 Hz, 2H), 1.85 (t, *J* = 2.3 Hz, 6H).

^{13}C NMR (125 MHz, $CDCl_3$, 23 ºC, δ): 166.5, 154.0, 146.3 – 144.0 (m), 141.6 – 139.3 (m), 137.8, 139.2 – 136.8 (m), 133.1, 132.7, 129.6, 128.9, 128.4, 119.9, 118.8 – 118.6 (m), 79.3, 41.3, 34.8, 29.5 (t, *J* = 4.2 Hz).

^{19}F NMR (470 MHz, $CDCl_3$, 23 ºC, δ): -138.3 – -138.4 (m, 2F), -154.8 (t, *J* = 21.1 Hz, 1F), -161.5 (td, *J* = 21.7, 6.8 Hz, 2F).

HRMS-ESI(m/z) calc'd for $C_{24}H_{19}ClF_5NO_2Na^+$ $[M+Na]^+$, 506.0917; found, 506.0914; deviation 0.57 ppm.

1-(Perfluorophenyl)adamantane (14)

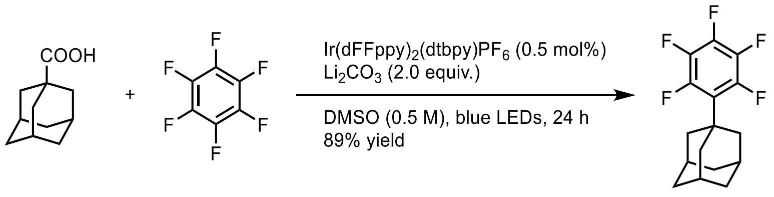

14

Under nitrogen atmosphere, to a 4-mL borosilicate vial equipped with a stir bar was added $Ir(dFFppy)_2(dtbpy)PF_6$ (1.5 mg, 1.5 µmol, 0.50 mol%), Li_2CO_3 (44.3 mg, 0.600 mmol, 2.00 equiv.), adamantane-1-carboxylic acid (54.1 mg, 0.300 mmol, 1.00 equiv.), dry DMSO (0.6 mL, c = 0.5 M), and perfluorobenzene (174 µL, 279 mg, 1.50 mmol, 5.00 equiv.). The vial was sealed with a septum-cap and placed 2 cm away from two 34 W blue LEDs. The temperature was kept at approximately 35 °C through cooling with a fan. After being stirred for 24 hours, the reaction mixture was poured into 20 mL of water, and the resulting mixture was extracted with EtOAc (3 × 20 mL). The combined organic phase was dried over $MgSO_4$, filtered through celite, and concentrated under vacuum. The residue was further purified by flash column chromatography on silica gel, eluting with hexanes to afford 80.4 mg (89% yield) of the title compound as a colorless solid.

R*f* = 0.86 (hexanes).

NMR Spectroscopy:

^{1}H NMR (500 MHz, $CDCl_3$, 23 ºC, δ): 2.25 – 2.19 (m, 6H), 2.10 – 2.02 (m, 3H), 1.88 – 1.72 (m, 6H).

^{13}C NMR (150 MHz, $CDCl_3$, 23 ºC, δ): 147.3 – 145.5 (m), 140.2 – 138.1 (m), 139.0 – 137.1 (m), 122.7 – 122.4 (m), 41.3 (t, *J* = 5.3 Hz), 40.7 – 40.5 (m), 36.6, 28.9.

^{19}F NMR (565 MHz, $CDCl_3$, 23 ºC, δ): -138.1 (dddd, *J* = 19.6, 8.8, 4.5, 2.2 Hz, 2F), -158.5 (tt, *J* = 21.3, 2.1 Hz, 1F), -163.0 – -163.1 (m, 2F).

HRMS-EI(m/z) calc'd for $C_{16}H_{15}F_5^+$ [M]$^+$, 302.1088; found, 302.1092; deviation 1.05 ppm.

These NMR spectroscopic data correspond to those of authentic sample reported in the literature.[2]

Pentafluoro-(2-phenylpropyl)benzene (15)

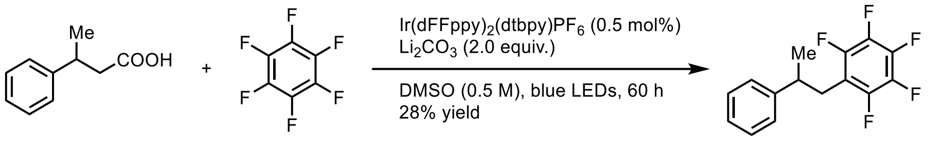

Under nitrogen atmosphere, to a 4-mL borosilicate vial equipped with a stir bar was added $Ir(dFFppy)_2(dtbpy)PF_6$ (1.5 mg, 1.5 µmol, 0.50 mol%), Li_2CO_3 (44.3 mg, 0.600 mmol, 2.00 equiv.), 3-phenylbutanoic acid (49.3 mg, 0.300 mmol, 1.00 equiv.), dry DMSO (0.6 mL, c = 0.5 M), and perfluorobenzene (347 µL, 558 mg, 3.00 mmol, 10.0 equiv.). The vial was sealed with a septum-cap and placed 2 cm away from two 34 W blue LEDs. The temperature was kept at approximately 35 °C through cooling with a fan. After being stirred for 60 hours, the reaction mixture was poured into 20 mL of water, and the resulting mixture was extracted with EtOAc (3 × 20 mL). The combined organic phase was dried over $MgSO_4$, filtered through celite, and concentrated under vacuum. The residue was further purified by flash column chromatography on silica gel, eluting with hexanes to afford 24.0 mg (28% yield) of the title compound as a colorless liquid.

R*f* = 0.60 (hexanes).

NMR Spectroscopy:

^{1}H NMR (600 MHz, $CDCl_3$, 23 ºC, δ): 7.30 – 7.27 (m, 2H), 7.22 – 7.19 (m, 1H), 7.19 – 7.17 (m, 2H), 3.09 – 3.02 (m, 1H), 2.98 – 2.88 (m, 2H), 1.30 (d, *J* = 7.0 Hz, 3H).

^{13}C NMR (150 MHz, $CDCl_3$, 23 ºC, δ): 146.2 – 144.3 (m), 145.3, 140.7 – 138.7 (m), 138.4 – 136.4 (m), 128.6, 126.9, 126.8, 114.2 – 113.9 (m), 39.9, 31.5 – 31.4 (m), 21.0.

^{19}F NMR (565 MHz, $CDCl_3$, 23 ºC, δ): -143.1 (dd, *J* = 22.7, 8.4 Hz, 2F), -157.4 (t, *J* = 20.9 Hz, 1F), -162.9 – -163.1 (m, 2F).

HRMS-EI(m/z) calc'd for $C_{15}H_{11}F_5^+$ [M]$^+$, 286.0775; found, 286.0777; deviation 0.55 ppm.

These NMR spectroscopic data correspond to those of authentic sample reported in the literature.[3]

1,3-Bis(perfluorophenyl)adamantane (16)

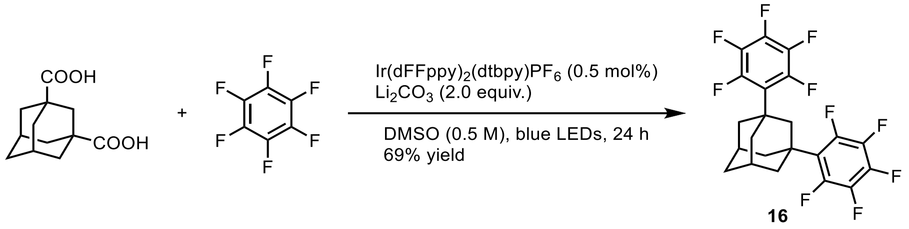

Under nitrogen atmosphere, to a 4-mL borosilicate vial equipped with a stir bar was added Ir(dFFppy)$_2$(dtbpy)PF$_6$ (1.5 mg, 1.5 µmol, 0.50 mol%), Li_2CO_3 (44.3 mg, 0.600 mmol, 2.00 equiv.), adamantane-1,3-dicarboxylic acid (67.3 mg, 0.300 mmol, 1.00 equiv.), dry DMSO (0.6 mL, c = 0.5 M), and perfluorobenzene (174 µL, 279 mg, 1.50 mmol, 5.00 equiv.). The vial was sealed with a septum-cap and placed 2 cm away from two 34 W blue LEDs. The temperature was kept at approximately 35 °C through cooling with a fan. After being stirred for 24 hours, the reaction mixture was poured into 20 mL of water, and the resulting mixture was extracted with EtOAc (3 × 20 mL). The combined organic phase was dried over $MgSO_4$, filtered through celite, and concentrated under vacuum. The residue was further purified by flash column chromatography on silica gel, eluting with hexanes to afford 97.0 mg (69% yield) of the title compound as a colorless solid.

R*f* = 0.60 (hexanes).

NMR Spectroscopy:

^{1}H NMR (500 MHz, $CDCl_3$, 23 °C, δ): 2.64 (s, 2H), 2.36 – 2.30 (m, 4H), 2.29 – 2.26 (m, 2H), 2.26 – 2.21 (m, 4H), 1.85 – 1.80 (m, 2H).

^{13}C NMR (150 MHz, $CDCl_3$, 23 °C, δ): 147.3 – 145.4 (m), 140.5 – 138.4 (m), 139.1 – 137.2 (m), 128.9 – 118.2 (m), 43.9 (p, *J* = 5.4 Hz), 41.2 – 41.1 (m), 40.2 (t, *J* = 5.5 Hz), 35.6, 29.4.

^{19}F NMR (470 MHz, $CDCl_3$, 23 °C, δ): -137.8 – -138.0 (m, 2F), -157.5 (t, *J* = 21.2 Hz, 1F), -162.4 – -162.5 (m, 2F).

HRMS-EI(m/z) calc'd for $C_{22}H_{14}F_{10}^+$ [M]$^+$, 468.0930; found, 468.0929; deviation 0.29 ppm.

Ciprofibrate derivative 17

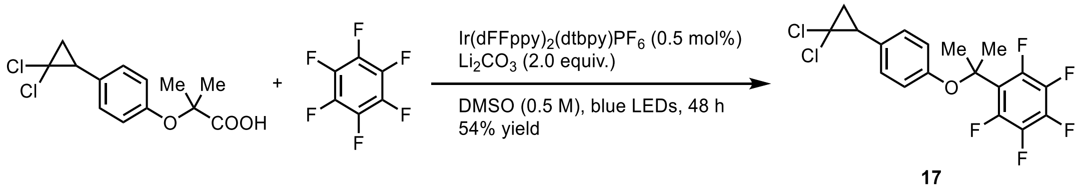

Under nitrogen atmosphere, to a 4-mL borosilicate vial equipped with a stir bar was added Ir(dFFppy)$_2$(dtbpy)PF$_6$ (1.5 mg, 1.5 µmol, 0.50 mol%), Li_2CO_3 (44.3 mg, 0.600 mmol, 2.00 equiv.), ciprofibrate (86.7 mg, 0.300 mmol, 1.00 equiv.), dry DMSO (0.6 mL, c = 0.5 M), and perfluorobenzene (174 µL, 279 mg, 1.50 mmol, 5.00 equiv.). The vial was sealed with a septum-cap and placed 2 cm away from two 34 W blue LEDs. The temperature was kept at approximately 35 °C through cooling with a fan. After being stirred for 48

hours, the reaction mixture was poured into 20 mL of water, and the resulting mixture was extracted with EtOAc (3 × 20 mL). The combined organic phase was dried over $MgSO_4$, filtered through celite, and concentrated under vacuum. The residue was further purified by flash column chromatography on silica gel, eluting with EtOAc/hexanes 1:100 (v/v) to afford 66.6 mg (54% yield) of the title compound as a colorless oil.

R*f* = 0.20 (EtOAc/hexanes 1:100 (v/v)).

NMR Spectroscopy:

^{1}H NMR (600 MHz, $CDCl_3$, 23 ºC, δ): 7.07 – 7.05 (m, 2H), 6.73 – 6.69 (m, 2H), 2.80 (dd, *J* = 10.7, 8.3 Hz, 1H), 1.92 (dd, *J* = 10.7, 7.4 Hz, 1H), 1.86 (t, *J* = 2.3 Hz, 6H), 1.75 (dd, *J* = 8.3, 7.4 Hz, 1H).

^{13}C NMR (150 MHz, $CDCl_3$, 23 ºC, δ): 154.8, 146.1 – 144.2 (m), 141.5 – 139.5 (m), 139.1 – 137.1 (m), 129.8, 128.5, 119.2, 118.6 (t, *J* = 13.0 Hz), 79.3, 60.9, 34.9, 29.6 – 29.5 (m), 26.0.

^{19}F NMR (470 MHz, $CDCl_3$, 23 ºC, δ): -138.3 – -138.4 (m, 2F), -154.7 – -154.9 (m, 1F), -161.4 – -161.6 (m, 2F).

HRMS-EI(m/z) calc'd for $C_9H_8Cl_2O^+$ $[A+H]^+$, 201.9947; found, 201.9949; deviation 1.14 ppm.

HRMS-EI(m/z) calc'd for $C_9H_6F_5^+$ $[B]^+$, 209.0384; found, 209.0387; deviation 1.35 ppm.

(The molecular ion peak can't be found with several methods including EI, ESI, CI, and APPI. The HRMS of the two fragments A and B were offered to support the structure of **17**.)

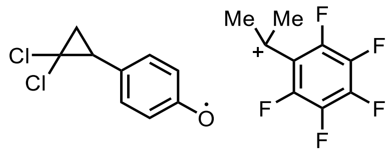

fragment A fragment B

(2S,3R)-2-(Methylcarboxyl)-3-pentafluorophenylnorbornan (18)

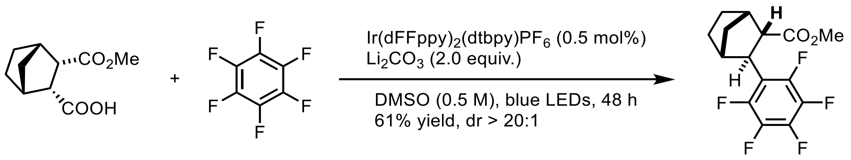

18

Under nitrogen atmosphere, to a 4-mL borosilicate vial equipped with a stir bar was added $Ir(dFFppy)_2(dtbpy)PF_6$ (1.5 mg, 1.5 µmol, 0.50 mol%), Li_2CO_3 (44.3 mg, 0.600 mmol, 2.00 equiv.), (2R,3S)-3-(methylcarboxyl)norbornan-2-carboxylic acid (59.5 mg, 0.300 mmol, 1.00 equiv.), dry DMSO (0.6 mL, c = 0.5 M), and perfluorobenzene (347 µL, 558 mg, 3.00 mmol, 10.0 equiv.). The vial was sealed with a septum-cap and placed 2 cm away from two 34 W blue LEDs. The temperature was kept at approximately 35 °C through cooling with a fan. After being stirred for 48 hours, the reaction mixture was poured into 20 mL of water, and the resulting mixture was extracted with EtOAc (3 × 20 mL). The combined organic phase was dried over $MgSO_4$, filtered through celite, and concentrated under vacuum. The residue was further purified by flash

column chromatography on silica gel, eluting with EtOAc/hexanes 1:50 (v/v) to afford 58.6 mg (61% yield) of the title compound as a colorless liquid.

R*f* = 0.40 (EtOAc/hexanes 1:50 (v/v)).

NMR Spectroscopy:

^{1}H NMR (500 MHz, $CDCl_3$, 23 ºC, δ): 3.69 (s, 3H), 3.48 (dd, *J* = 6.6, 1.5 Hz, 1H), 3.18 – 3.12 (m, 1H), 2.74 – 2.68 (m, 1H), 2.44 – 2.39 (m, 1H), 1.99 – 1.92 (m, 1H), 1.70 – 1.60 (m, 1H), 1.57 – 1.47 (m, 2H), 1.46 – 1.38 (m, 2H).

^{13}C NMR (125 MHz, $CDCl_3$, 23 ºC, δ): 173.9, 145.4 (dm, *J* = 246.5 Hz), 139.6 (dm, *J* = 252.2 Hz), 137.8 (dm, *J* = 248.0 Hz), 117.9 – 117.5 (m), 53.1, 52.0, 43.5, 41.1, 41.0, 39.1 (t, *J* = 4.0 Hz), 30.9, 23.9.

^{19}F NMR (470 MHz, $CDCl_3$, 23 ºC, δ): -140.3 – -140.5 (m, 2F), -157.5 (t, *J* = 21.1 Hz, 1F), -162.6 – -162.8 (m, 2F).

HRMS-EI(m/z) calc'd for $C_{15}H_{13}F_5O_2^+$ $[M]^+$, 320.0830; found, 320.0830; deviation 0.06 ppm.

(4S,5S)-*N*-Boc-2,2-dimethyl-4-phenyl-5-pentafluorophenyloxazolidine (19)

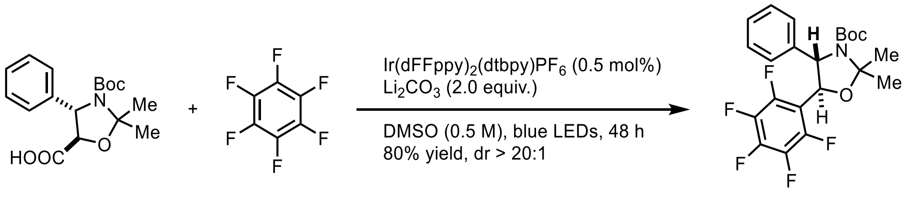

19

Under nitrogen atmosphere, to a 4-mL borosilicate vial equipped with a stir bar was added $Ir(dFFppy)_2(dtbpy)PF_6$ (1.5 mg, 1.5 µmol, 0.50 mol%), Li_2CO_3 (44.3 mg, 0.600 mmol, 2.00 equiv.), (4S,5R)-*N*-Boc-2,2-dimethyl-4-phenyloxazolidine-5-carboxylic acid (96.4 mg, 0.300 mmol, 1.00 equiv.), dry DMSO (0.6 mL, c = 0.5 M), and perfluorobenzene (347 µL, 558 mg, 3.00 mmol, 10.0 equiv.). The vial was sealed with a septum-cap and placed 2 cm away from two 34 W blue LEDs. The temperature was kept at approximately 35 °C through cooling with a fan. After being stirred for 48 hours, the reaction mixture was poured into 20 mL of water, and the resulting mixture was extracted with EtOAc (3 × 20 mL). The combined organic phase was dried over $MgSO_4$, filtered through celite, and concentrated under vacuum. The residue was further purified by flash column chromatography on silica gel, eluting with EtOAc/hexanes 1:40 (v/v) to afford 105.6 mg (80% yield) of the title compound as a colorless solid.

R*f* = 0.20 (EtOAc/hexanes 1:40 (v/v)).

NMR Spectroscopy:

^{1}H NMR (500 MHz, DMSO-*d*$_6$, 80 ºC, δ): 7.36 – 7.32 (m, 2H), 7.31 – 7.27 (m, 1H), 7.23 – 7.18 (m, 2H), 5.14 (d, *J* = 8.8 Hz, 1H), 4.90 (d, *J* = 8.8 Hz, 1H), 1.77 (s, 3H), 1.71 (s, 3H), 1.13 (s, 9H).

^{13}C NMR (125 MHz, DMSO-*d*$_6$, 80 ºC, δ): 150.6, 144.9 (dm, *J* = 250.3 Hz), 140.6 (dm, *J* = 252.8 Hz), 138.4,

136.9 (dm, J = 250.2 Hz), 128.1, 127.2, 125.5, 110.1 (t, J = 12.5 Hz), 94.9, 79.2, 74.9, 65.2, 27.2, 25.8, 25.3.

^{19}F NMR (470 MHz, DMSO-d_6, 80 ºC, δ): -142.4 – -142.5 (m, 2F), -154.0 (t, J = 21.7 Hz, 1F), -162.6 – -162.8 (m, 2F).

HRMS-ESI(m/z) calc'd for $C_{22}H_{22}F_5NO_3Na^+$ [M+Na]$^+$, 466.1412; found, 466.1409; deviation 0.63 ppm.

Estrone derivative 20

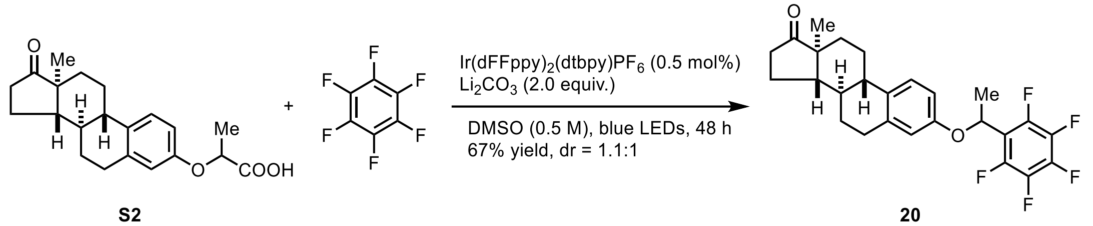

S2 **20**

Under nitrogen atmosphere, to a 4-mL borosilicate vial equipped with a stir bar was added Ir(dFFppy)$_2$(dtbpy)PF$_6$ (1.5 mg, 1.5 µmol, 0.50 mol%), Li_2CO_3 (44.3 mg, 0.600 mmol, 2.00 equiv.), **S2** (103 mg, 0.300 mmol, 1.00 equiv.), dry DMSO (0.6 mL, c = 0.5 M), and perfluorobenzene (347 µL, 558 mg, 3.00 mmol, 10.0 equiv.). The vial was sealed with a septum-cap and placed 2 cm away from two 34 W blue LEDs. The temperature was kept at approximately 35 °C through cooling with a fan. After being stirred for 48 hours, the reaction mixture was poured into 20 mL of water, and the resulting mixture was extracted with EtOAc (3 × 20 mL). The combined organic phase was dried over $MgSO_4$, filtered through celite, and concentrated under vacuum. The residue was further purified by flash column chromatography on silica gel, eluting with EtOAc/hexanes 1:10 (v/v) to afford 93.4 mg (67% yield) of the title compound as a colorless solid.

R_f = 0.25 (EtOAc/hexanes 1:10 (v/v)).

NMR Spectroscopy:

^{1}H NMR (600 MHz, $CDCl_3$, 23 ºC, δ): 7.14 (d, J = 8.6 Hz, 1H), 6.68 (dt, J = 8.6, 2.6 Hz, 1H), 6.64 (d, J = 2.0 Hz, 1H), 5.69 (q, J = 6.6 Hz, 1H), 2.90 – 2.80 (m, 2H), 2.53 – 2.46 (m, 1H), 2.38 – 2.31 (m, 1H), 2.25 – 2.18 (m, 1H), 2.17 – 2.09 (m, 1H), 2.07 – 2.02 (m, 1H), 2.01 – 1.96 (m, 1H), 1.95 – 1.91 (m, 1H), 1.76 (d, J = 6.6 Hz, 3H), 1.65 – 1.37 (m, 6H), 0.89 (s, 3H).

^{13}C NMR (150 MHz, $CDCl_3$, 23 ºC, δ): 221.0, 155.1, 145.8 – 143.9 (m), 141.9 – 139.9 (m), 138.7 – 136.7 (m), 138.19 (138.18), 133.41 (133.38), 126.62 (126.59), 115.99 (115.92), 115.8 – 115.5 (m), 112.7, 66.94 (66.92), 50.5, 48.1, 44.10 (44.09), 38.36 (38.35), 36.0, 31.7, 29.70 (29.67), 26.6, 25.95 (25.91), 21.7, 21.1, 14.0.

^{19}F NMR (470 MHz, $CDCl_3$, 23 ºC, δ): -142.9 (dd, J = 22.5, 8.0 Hz, 2F), -154.6 (t, J = 20.8 Hz, 1F), -161.62 – -161.8 (m, 2F).

HRMS-ESI(m/z) calc'd for $C_{26}H_{25}F_5O_2Na^+$ [M+Na]$^+$, 487.1667; found, 487.1665; deviation 0.33 ppm.

Glycyrrhetic acid derivative 21

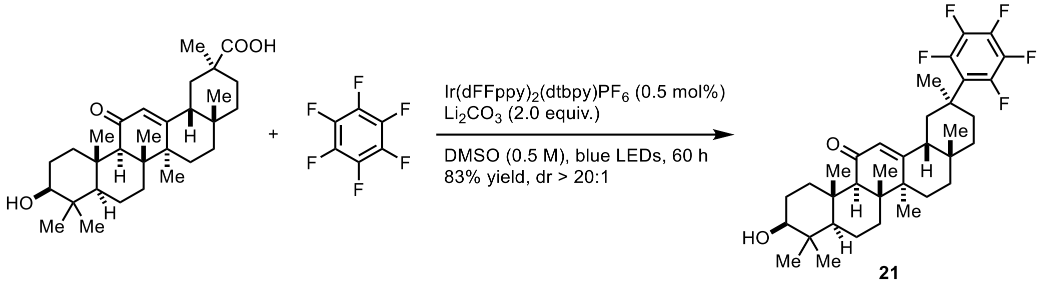

21

Under nitrogen atmosphere, to a 4-mL borosilicate vial equipped with a stir bar was added Ir(dFFppy)$_2$(dtbpy)PF$_6$ (1.5 mg, 1.5 µmol, 0.50 mol%), Li_2CO_3 (44.3 mg, 0.600 mmol, 2.00 equiv.), glycyrrhetic acid (142 mg, 0.300 mmol, 1.00 equiv.), dry DMSO (0.6 mL, c = 0.5 M), and perfluorobenzene (347 µL, 558 mg, 3.00 mmol, 10.0 equiv.). The vial was sealed with a septum-cap and placed 2 cm away from two 34 W blue LEDs. The temperature was kept at approximately 35 °C through cooling with a fan. After being stirred for 60 hours, the reaction mixture was poured into 20 mL of water, and the resulting mixture was extracted with EtOAc (3 × 20 mL). The combined organic phase was dried over $MgSO_4$, filtered through celite, and concentrated under vacuum. The residue was further purified by flash column chromatography on silica gel, eluting with EtOAc/hexanes 1:5 (v/v) to afford 147.6 mg (83% yield) of the title compound as a colorless solid.

R*f* = 0.20 (EtOAc/hexanes 1:5 (v/v)).

NMR Spectroscopy:

^{1}H NMR (500 MHz, $CDCl_3$, 23 ºC, δ): 5.64 (s, 1H), 3.22 (dd, *J* = 11.0, 5.2 Hz, 1H), 2.77 (dt, *J* = 13.4, 3.6 Hz, 1H), 2.38 – 2.25 (m, 3H), 2.18 – 2.05 (m, 2H), 1.98 – 1.90 (m, 1H), 1.89 – 1.79 (m, 2H), 1.70 – 1.55 (m, 5H), 1.47 – 1.45 (m, 3H), 1.50 – 1.38 (m, 5H), 1.35 (s, 3H), 1.27 – 1.16 (m, 1H), 1.14 (s, 3H), 1.13 (s, 3H), 1.07 – 1.00 (m, 1H), 1.00 (s, 3H), 0.99 – 0.92 (m, 1H), 0.91 (s, 3H), 0.80 (s, 3H).

^{13}C NMR (150 MHz, $CDCl_3$, 23 ºC, δ): 200.3, 169.0, 147.1 – 145.2 (m), 140.4 – 138.3 (m), 139.1 – 137.2 (m), 128.9, 123.3 – 123.0 (m), 78.9, 62.0, 55.1, 47.0, 45.6, 43.5, 41.8 – 41.3 (m), 41.2 (t, *J* = 5.9 Hz), 39.3, 39.2, 37.2, 35.8, 32.9, 32.0, 31.4 (t, *J* = 6.2 Hz), 28.6, 28.2, 27.4, 26.6, 26.4, 23.5, 21.5 (t, *J* = 4.2 Hz), 18.9, 17.6, 16.5, 15.7.

^{19}F NMR (470 MHz, $CDCl_3$, 23 ºC, δ): -136.9 (dd, *J* = 21.2, 6.0 Hz, 2F), -157.6 (t, *J* = 21.5 Hz, 1F), -161.8 – -162.5 (m, 2F).

HRMS-ESI(m/z) calc'd for $C_{33}H_{45}F_5O_2Na^+$ [M+Na]$^+$, 615.3232; found, 615.3233; deviation 0.10 ppm.

N-Boc-pentafluorobenzylamine (22)

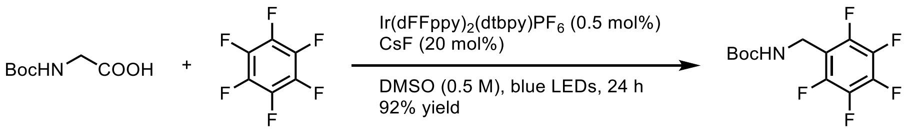

22

Under nitrogen atmosphere, to a 4-mL borosilicate vial equipped with a stir bar was added Ir(dFFppy)$_2$(dtbpy)PF$_6$ (1.5 mg, 1.5 µmol, 0.50 mol%), CsF (9.2 mg, 60 µmol, 20 mol%), *N*-Boc-glycine (52.6 mg, 0.300 mmol, 1.00 equiv.), dry DMSO (0.6 mL, c = 0.5 M), and perfluorobenzene (70.0 µL, 112 mg, 0.600 mmol, 2.00 equiv.). The vial was sealed with a septum-cap and placed 2 cm away from two 34 W blue LEDs. The temperature was kept at approximately 35 °C through cooling with a fan. After being stirred for 24 hours, the reaction mixture was poured into 20 mL of water, and the resulting mixture was extracted with EtOAc (3 × 20 mL). The combined organic phase was dried over MgSO$_4$, filtered through celite, and concentrated under vacuum. The residue was further purified by flash column chromatography on silica gel, eluting with EtOAc/hexanes 1:10 (v/v) to afford 82.0 mg (92% yield) of the title compound as a colorless solid.

R*f* = 0.24 (EtOAc/hexanes 1:10 (v/v)).

NMR Spectroscopy:

^{1}H NMR (500 MHz, CDCl$_3$, 23 ºC, δ): 5.05 (t, *J* = 6.4 Hz, 1H), 4.48 – 4.28 (m, 2H), 1.41 (s, 9H).

^{13}C NMR (125 MHz, CDCl$_3$, 23 ºC, δ): 155.3, 146.5 – 144.2 (m), 142.1 – 139.7 (m), 138.8 – 136.4 (m), 112.7 – 112.2 (m), 80.3, 32.6, 28.4.

^{19}F NMR (470 MHz, CDCl$_3$, 23 ºC, δ): -143.3 (dd, *J* = 22.7, 9.0 Hz, 2F), -155.2 (t, *J* = 20.5 Hz, 1F), -162.1 (td, *J* = 22.4, 8.8 Hz, 2F).

HRMS-ESI(m/z) calc'd for $C_{11}H_{12}F_5NO_2Na^+$ [M+Na]$^+$, 320.0680; found, 320.0683; deviation 0.81 ppm.

N-Boc-chlorotetrafluorobenzylamines 23a, 23b, 23c

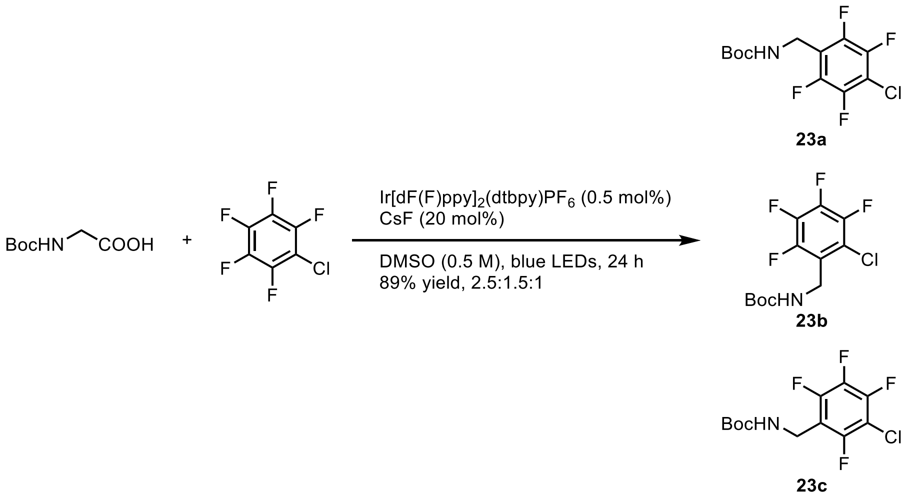

Under nitrogen atmosphere, to a 4-mL borosilicate vial equipped with a stir bar was added Ir(dFFppy)$_2$(dtbpy)PF$_6$ (1.5 mg, 1.5 µmol, 0.50 mol%), CsF (9.2 mg, 60 µmol, 20 mol%), *N*-Boc-glycine (52.6 mg, 0.300 mmol, 1.00 equiv.), dry DMSO (0.6 mL, c = 0.5 M), and chloropentafluorobenzene (77.4 µL, 122 mg, 0.600 mmol, 2.00 equiv.). The vial was sealed with a septum-cap and placed 2 cm away from two 34 W blue LEDs. The temperature was kept at approximately 35 °C through cooling with a fan. After being stirred for 24 hours, the reaction mixture was poured into 20 mL of water, and the resulting mixture was extracted with EtOAc

(3 × 20 mL). The combined organic phase was dried over $MgSO_4$, filtered through celite, and concentrated under vacuum. The residue was further purified by flash column chromatography on silica gel, eluting with EtOAc/hexanes 1:10 (v/v) to afford 83.8 mg (89% yield) of the mixture of **23a**, **23b**, and **23c**. The ratio of them is 2.5:1.5:1 determined by the ^{19}F NMR of the reaction. Further purification by HPLC (ChiralART Cellulose SJ (250 × 20.0 mm: 5 μm) connected with Chiralcel OJ-H (250 × 20.0 mm: 5 μm), Methanol/Water = 75:25, flow rate = 20 mL/min, 27 °C) provided pure **23a** as a colorless solid, **23b** as a colorless solid and **23c** as a colorless solid.

The following data were obtained for the pure isomers:

***N*-Boc-4-chlorotetrafluorobenzylamine (23a):**

R*f* = 0.24 (EtOAc/hexanes 1:10 (v/v)).

NMR Spectroscopy:

^{1}H NMR (500 MHz, $CDCl_3$, 23 ºC, δ): 5.11 (brs, 1H), 4.42 (d, *J* = 6.3 Hz, 2H), 1.40 (d, *J* = 1.2 Hz, 9H).

^{13}C NMR (125 MHz, $CDCl_3$, 23 ºC, δ): 155.3, 145.3 (dm, *J* = 248.2 Hz), 144.0 (ddd, *J* = 250.3, 15.6, 4.3 Hz), 116.3 (t, *J* = 17.6 Hz), 112.1 – 111.6 (m), 80.3, 32.8, 28.4.

^{19}F NMR (470 MHz, $CDCl_3$, 23 ºC, δ): -141.1 (dd, *J* = 22.3, 10.6 Hz, 2F), -142.7 (dd, *J* = 23.4, 10.3 Hz, 2F).

HRMS-ESI(m/z) calc'd for $C_{12}H_{12}NO_2F_4ClNa^+$ $[M+Na]^+$, 336.0385; found, 336.0386; deviation 0.45 ppm.

***N*-Boc-2-chlorotetrafluorobenzylamine (23b):**

R*f* = 0.24 (EtOAc/hexanes 1:10 (v/v)).

NMR Spectroscopy:

^{1}H NMR (500 MHz, $CDCl_3$, 23 ºC, δ): 5.01 (brs, 1H), 4.48 (d, *J* = 6.0 Hz, 2H), 1.42 (s, 9H).

^{13}C NMR (125 MHz, $CDCl_3$, 23 ºC, δ): 155.2, 146.3 (dm, *J* = 250.5 Hz), 145.9 – 143.5 (m), 142.0 – 139.3 (m), 140.9 – 138.3 (m), 122.1 – 121.2 (m), 117.9 – 117.6 (m), 80.3, 35.7, 28.4.

^{19}F NMR (470 MHz, $CDCl_3$, 23 ºC, δ): -137.6 (dd, *J* = 21.5, 10.0 Hz, 1F), -140.2 (dd, *J* = 21.9, 10.0 Hz, 1F), -154.4 (t, *J* = 20.7 Hz, 1F), -156.3 (t, *J* = 21.3 Hz, 1F).

HRMS-ESI(m/z) calc'd for $C_{12}H_{12}NO_2F_4ClNa^+$ $[M+Na]^+$, 336.0385; found, 336.0386; deviation 0.45 ppm.

***N*-Boc-3-chlorotetrafluorobenzylamine (23c):**

R*f* = 0.24 (EtOAc/hexanes 1:10 (v/v)).

NMR Spectroscopy:

^{1}H NMR (500 MHz, $CDCl_3$, 23 ºC, δ): 4.99 (brs, 1H), 4.41 (d, *J* = 5.9 Hz, 2HH), 1.42 (s, 9H).

^{13}C NMR (125 MHz, $CDCl_3$, 23 ºC, δ): 155.3, 152.6 (dm, *J* = 248.1 Hz), 148.3 (dm, *J* = 250.4 Hz), 148.9 –

146.6 (m), 139.6 – 136.1 (m), 113.0 – 112.4 (m), 107.5 – 107.0 (m), 80.3, 32.8, 28.4.

^{19}F NMR (470 MHz, $CDCl_3$, 23 ºC, δ): -121.3 (d, J = 11.1 Hz, 1F), -133.8 (d, J = 21.0 Hz, 1F), -137.7 (d, J = 21.7 Hz, 1F), -161.8 (td, J = 21.8, 9.1 Hz, 1F).

HRMS-ESI(m/z) calc'd for $C_{12}H_{12}NO_2F_4ClNa^+$ $[M+Na]^+$, 336.0385; found, 336.0386; deviation 0.45 ppm.

N-Boc-tetrafluorobenzylamines 24a, 24b

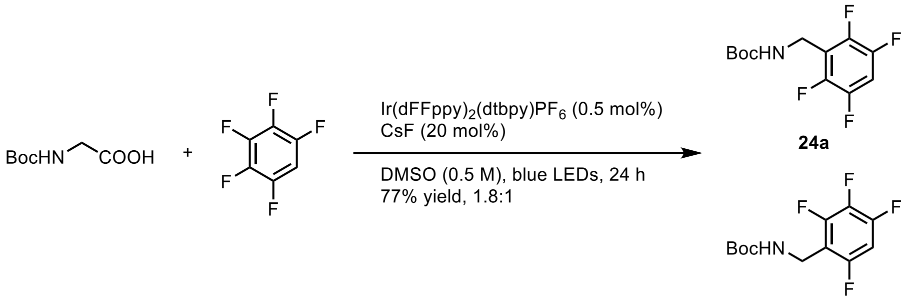

Under nitrogen atmosphere, to a 4-mL borosilicate vial equipped with a stir bar was added $Ir(dFFppy)_2(dtbpy)PF_6$ (1.5 mg, 1.5 µmol, 0.50 mol%), CsF (9.2 mg, 60 µmol, 20 mol%), *N*-Boc-glycine (52.6 mg, 0.300 mmol, 1.00 equiv.), dry DMSO (0.6 mL, c = 0.5 M), and pentafluorobenzene (167 µL, 252 mg, 1.50 mmol, 5.00 equiv.). The vial was sealed with a septum-cap and placed 2 cm away from two 34 W blue LEDs. The temperature was kept at approximately 35 °C through cooling with a fan. After being stirred for 24 hours, the reaction mixture was poured into 20 mL of water, and the resulting mixture was extracted with EtOAc (3 × 20 mL). The combined organic phase was dried over $MgSO_4$, filtered through celite, and concentrated under vacuum. The residue was further purified by flash column chromatography on silica gel, eluting with EtOAc/hexanes 1:10 (v/v) to afford 64.5 mg (77% yield) of the mixture of **24a** and **24b**. The ratio of them is 3.3:1 determined by the ^{19}F NMR of the reaction. Further purification by HPLC (YMC-Actus Triart C18 (150 × 30.0 mm: 5 µm) MeCN/Water = 55:45, flow rate = 42.5 mL/min, 35 °C) provided pure **24a** as a colorless solid and **24b** as a colorless solid.

The following data was obtained for the pure isomers:

N-Boc-2,3,5,6-tetrafluorobenzylamine (24a):

R*f* = 0.25 (EtOAc/hexanes 1:10 (v/v)).

NMR Spectroscopy:

^{1}H NMR (500 MHz, $CDCl_3$, 23 ºC, δ): 7.00 (tt, J = 9.7, 7.4 Hz, 1H), 5.00 (brs, 1H), 4.52 – 4.31 (m, 2H), 1.42 (s, 9H).

^{13}C NMR (125 MHz, $CDCl_3$, 23 ºC, δ): 155.4, 147.3 – 144.7 (m), 146.4 – 143.7 (m), 118.8 – 117.6 (m), 105.5 (t, J = 22.6 Hz), 80.2, 33.0, 28.4.

^{19}F NMR (470 MHz, $CDCl_3$, 23 ºC, δ): -138.9 (dd, J = 21.9, 11.5 Hz, 2F), -143.9 (dd, J = 21.6, 11.3 Hz,

2F).

HRMS-ESI(m/z) calc'd for $C_{12}H_{13}F_4NO_2Na^+$ [M+Na]$^+$, 302.0774; found, 302.0773; deviation 0.54 ppm.

***N*-Boc-2,3,4,6-tetrafluorobenzylamine (24b):**

R*f* = 0.25 (EtOAc/hexanes 1:10 (v/v)).

NMR Spectroscopy:

^{1}H NMR (600 MHz, $CDCl_3$, 23 ºC, δ): 6.80 – 6.73 (m, 1H), 4.93 (brs, 1H), 4.44 – 4.24 (m, 2H), 1.42 (s, 9H).

^{13}C NMR (150 MHz, $CDCl_3$, 23 ºC, δ): 155.7 (dddd, *J* = 247.1, 12.8, 9.1, 3.8 Hz), 155.4, 151.4 – 149.1 (m), 137.3 (dtd, *J* = 248.8, 15.5, 5.2 Hz), 112.8 – 112.2 (m), 101.0 (ddd, *J* = 28.4, 21.5, 3.9 Hz), 80.1, 32.6, 28.4. (One carbon signal was missing because of overlap.)

^{19}F NMR (565 MHz, $CDCl_3$, 23 ºC, δ): -118.6 – -118.8 (m, 1F), -132.6 – -133.2 (m, 1F), -135.4 – -136.1 (m, 1F), -164.5 – -165.1 (m, 1F).

HRMS-ESI(m/z) calc'd for $C_{12}H_{13}F_4NO_2Na^+$ [M+Na]$^+$, 302.0775; found, 302.0773; deviation 0.44 ppm.

N-Boc-trifluoro-(trifluoromethyl)benzylamines 25a, 25b, 25c

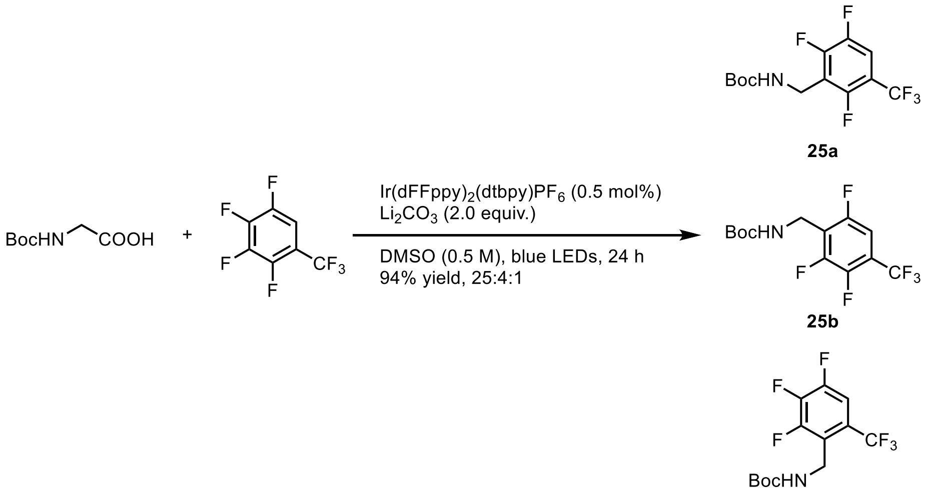

Under nitrogen atmosphere, to a 4-mL borosilicate vial equipped with a stir bar was added Ir(dFFppy)$_2$(dtbpy)PF_6 (1.5 mg, 1.5 µmol, 0.50 mol%), Li_2CO_3 (44.3 mg, 0.600 mmol, 2.00 equiv.), *N*-Boc-glycine (52.6 mg, 0.300 mmol, 1.00 equiv.), dry DMSO (0.6 mL, c = 0.5 M), and 1,2,3,4-tetrafluoro-5-(trifluoromethyl)benzene (131 mg, 0.600 mmol, 2.00 equiv.). The vial was sealed with a septum-cap and placed 2 cm away from two 34 W blue LEDs. The temperature was kept at approximately 35 °C through cooling with a fan. After being stirred for 24 hours, the reaction mixture was poured into 20 mL of water, and the resulting mixture was extracted with EtOAc (3 × 20 mL). The combined organic phase was dried over $MgSO_4$, filtered through celite, and concentrated under vacuum. The residue was further purified by flash column chromatography on silica gel, eluting with EtOAc/hexanes 1:10 (v/v) to afford 92.8 mg (94% yield) of the mixture

of **25a**, **25b**, and **25c**. The ratio of them is 25:4:1 determined by the ^{19}F NMR of the reaction. Further purification by HPLC (YMC-Actus Triart C18 (150 × 30.0 mm: 5 μm) MeCN/Water = 60:40, flow rate = 42.5 mL/min, 35 °C) provided pure **25a** as a colorless solid, **25b** as a colorless solid, and **25c** as a colorless solid.

The following data was obtained for the pure isomers:

***N*-Boc-2,3,6-trifluoro-5-(trifluoromethyl)benzylamine (25a):**

R*f* = 0.25 (EtOAc/hexanes 1:10 (v/v)).

NMR Spectroscopy:

^{1}H NMR (600 MHz, $CDCl_3$, 23 ºC, δ): 7.13 (ddd, *J* = 8.7, 4.9, 2.2 Hz, 1H), 5.03 (brs, 1H), 4.47 – 4.43 (m, 2H), 1.42 (s, 9H).

^{13}C NMR (150 MHz, $CDCl_3$, 23 ºC, δ): 155.9 (ddd, *J* = 248.1, 6.6, 3.5 Hz), 155.4, 149.8 (ddd, *J* = 253.8, 13.6, 8.5 Hz), 146.2 – 143.9 (m), 121.5 (qt, *J* = 272.8, 3.3 Hz), 121.4 – 120.9 (m), 119.5 (qt, *J* = 34.8, 10.3 Hz), 109.0 – 108.7 (m), 80.4, 33.0, 28.4.

^{19}F NMR (565 MHz, $CDCl_3$, 23 ºC, δ): -61.4 (d, *J* = 12.9 Hz, 3F), -117.5 – -117.6 (m, 1F), -134.6 (d, *J* = 20.6 Hz, 1F), -142.9 – -143.0 (m, 1F).

HRMS-ESI(m/z) calc'd for $C_{13}H_{13}NO_2NaF_6^+$ [M+Na]$^+$, 352.0743; found, 352.0741; deviation 0.39 ppm.

***N*-Boc-2,3,6-trifluoro-4-(trifluoromethyl)benzylamine (25b):**

R*f* = 0.25 (EtOAc/hexanes 1:10 (v/v)).

NMR Spectroscopy:

^{1}H NMR (600 MHz, $CDCl_3$, 23 ºC, δ): 7.43 – 7.36 (m, 1H), 4.96 (brs, 1H), 4.49 – 4.45 (m, 2H), 1.44 (s, 9H).

^{13}C NMR (150 MHz, $CDCl_3$, 23 ºC, δ): 155.3, 155.2 – 153.4 (m), 152.7 – 150.2 (m), 147.6 – 145.2 (m), 121.7 (q, *J* = 272.6 Hz), 119.3 – 118.7 (m), 114.9 – 114.7 (m), 114.7 – 114.3 (m), 80.4, 32.9, 28.4.

^{19}F NMR (565 MHz, $CDCl_3$, 23 ºC, δ): -61.1 (d, *J* = 13.2 Hz, 3F), -120.0 – -120.2 (m, 1F), -130.0 – -130.2 (m, 1F), -139.2 – -139.5 (m, 1F).

HRMS-ESI(m/z) calc'd for $C_{13}H_{13}NO_2NaF_6^+$ [M+Na]$^+$, 352.0743; found, 352.0742; deviation 0.11 ppm.

***N*-Boc-2,3,4-trifluoro-6-(trifluoromethyl)benzylamine (25c):**

R*f* = 0.25 (EtOAc/hexanes 1:10 (v/v)).

NMR Spectroscopy:

^{1}H NMR (600 MHz, $CDCl_3$, 23 ºC, δ): 7.35 (ddd, *J* = 9.6, 6.7, 2.2 Hz, 1H), 4.75 (brs, 1H), 4.54 (d, *J* = 5.7 Hz, 2H), 1.44 (s, 9H).

^{13}C NMR (150 MHz, $CDCl_3$, 23 ºC, δ): 155.0, 152.3 – 150.4 (m), 151.0 – 149.0 (m), 142.2 (dt, *J* = 259.4,

15.2 Hz), 125.3 – 124.1 (m), 123.4 – 122.9 (m), 122.9 (q, J = 273.6 Hz), 111.3 – 111.0 (m), 80.2, 34.8, 28.4.

^{19}F NMR (565 MHz, $CDCl_3$, 23 ºC, δ): -58.7 (s, 3F), -131.1 – -131.3 (m, 1F), -131.9 – -132.0 (m, 1F), -152.11 – -152.29 (m, 1F).

HRMS-CI(m/z) calc'd for $C_{13}H_{14}NO_2F_6^+$ $[M+H]^+$, 330.0923; found, 330.0923; deviation 0.05 ppm.

N-Boc-trifluoro-methylcarboxylbenzylamines 26a, 26b, 26c

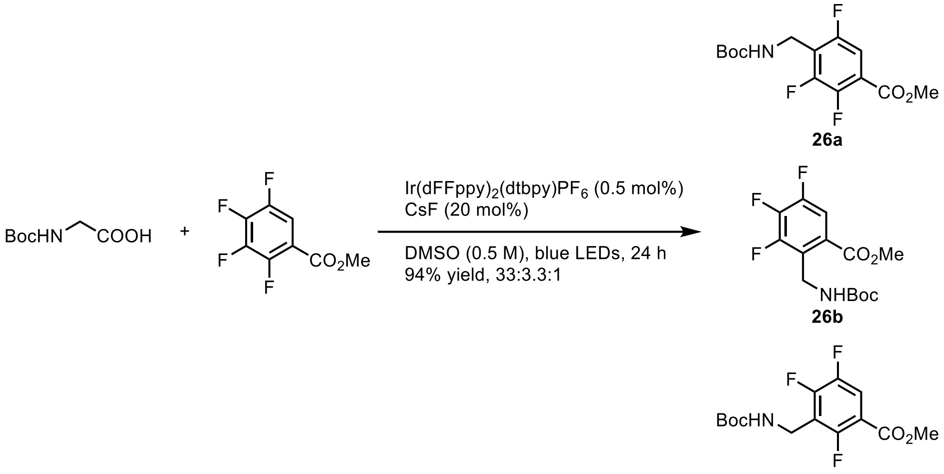

Under nitrogen atmosphere, to a 4-mL borosilicate vial equipped with a stir bar was added $Ir(dFFppy)_2(dtbpy)PF_6$ (1.5 mg, 1.5 µmol, 0.50 mol%), CsF (9.2 mg, 60 µmol, 20 mol%), *N*-Boc-glycine (52.6 mg, 0.300 mmol, 1.00 equiv.), dry DMSO (0.6 mL, c = 0.5 M), and methyl-2,3,4,5-tetrafluorobenzoate (125 mg, 0.600 mmol, 2.00 equiv.). The vial was sealed with a septum-cap and placed 2 cm away from two 34 W blue LEDs. The temperature was kept at approximately 35 °C through cooling with a fan. After being stirred for 24 hours, the reaction mixture was poured into 20 mL of water, and the resulting mixture was extracted with EtOAc (3 × 20 mL). The combined organic phase was dried over $MgSO_4$, filtered through celite, and concentrated under vacuum. The residue was further purified by flash column chromatography on silica gel, eluting with EtOAc/hexanes 1:5 (v/v) to afford 90.0 mg (94% yield) of the mixture of **26a**, **26b**, and **26c**. The ratio of them is 33:3.3:1 determined by the ^{19}F NMR of the reaction. Further purification by HPLC (YMC-Actus Triart C18 (150 × 30.0 mm: 5 µm) MeCN/Water = 55:45, flow rate = 42.5 mL/min, 35 °C) provided pure **26a** as a colorless solid, **26b** as a colorless solid, and **26c** as a colorless solid.

The following data was obtained for the pure isomers:

N-Boc-2,3,6-trifluoro-4-(methyl)carboxylbenzylamine (26a):

R_f = 0.40 (EtOAc/hexanes 1:5 (v/v)).

NMR Spectroscopy:

^{1}H NMR (600 MHz, $CDCl_3$, 23 ºC, δ): 7.36 (ddd, J = 9.5, 5.0, 2.2 Hz, 1H), 5.17 (t, J = 6.4 Hz, 1H), 4.46 –

4.35 (m, 2H), 3.89 (s, 3H), 1.37 (s, 9H).

^{13}C NMR (150 MHz, $CDCl_3$, 23 ºC, δ): 163.0 (q, *J* = 3.3 Hz), 155.6 (ddd, *J* = 246.9, 6.6, 3.4 Hz), 155.4, 149.8 (ddd, *J* = 252.1, 14.8, 8.2 Hz), 147.1 (ddd, *J* = 260.4, 14.6, 3.8 Hz), 122.06 – 120.82 (m), 119.4 (t, *J* = 8.9 Hz), 112.6 (dd, *J* = 26.3, 3.8 Hz), 80.0, 52.9, 33.0, 28.3.

^{19}F NMR (470 MHz, $CDCl_3$, 23 ºC, δ): -119.3 – -119.5 (m, 1F), -135.9 (d, *J* = 20.9 Hz, 1F), -138.7 – -138.9 (m, 1F).

HRMS-ESI(m/z) calc'd for $C_{14}H_{16}NO_4NaF_3^+$ [M+Na]$^+$, 342.0924; found, 342.0923; deviation 0.16 ppm.

***N*-Boc-2,3,4-trifluoro-6-(methyl)carboxylbenzylamine (26b):**

R*f* = 0.40 (EtOAc/hexanes 1:5 (v/v)).

NMR Spectroscopy:

^{1}H NMR (600 MHz, $CDCl_3$, 23 ºC, δ): 7.69 – 7.60 (m, 1H), 5.51 (brs, 1H), 4.59 (d, *J* = 6.5 Hz, 2H), 3.93 (s, 3H), 1.41 (s, 9H).

^{13}C NMR (150 MHz, $CDCl_3$, 23 ºC, δ): 165.5, 155.5, 150.3 (d, *J* = 252.8 Hz), 149.7 (dd, *J* = 249.9, 9.9 Hz), 143.8 – 141.5 (m), 126.7 (d, *J* = 13.9 Hz), 125.3, 115.8 – 114.8 (m), 79.7, 53.0, 34.8, 28.5.

^{19}F NMR (565 MHz, $CDCl_3$, 23 ºC, δ): -133.4 (dd, *J* = 20.7, 7.9 Hz, 1F), -134.1 (ddd, *J* = 20.8, 10.5, 7.8 Hz, 1F), -151.1 – -151.3 (m, 1F).

HRMS-ESI(m/z) calc'd for $C_{14}H_{16}NO_4NaF_3^+$ [M+Na]$^+$, 342.0924; found, 342.0927; deviation 0.90 ppm.

***N*-Boc-2,3,6-trifluoro-5-(methyl)carboxylbenzylamine (26c):**

R*f* = 0.40 (EtOAc/hexanes 1:5 (v/v)).

NMR Spectroscopy:

^{1}H NMR (600 MHz, $CDCl_3$, 23 ºC, δ): 6.96 – 6.91 (m, 1H), 5.17 (brs, 1H), 4.46 (d, *J* = 6.6 Hz, 2H), 3.96 (s, 3H), 1.42 (s, 9H).

^{13}C NMR (150 MHz, $CDCl_3$, 23 ºC, δ): 164.8, 157.0 – 155.2 (m), 155.3, 151.8 (ddd, *J* = 257.2, 15.5, 12.8 Hz), 146.8 – 144.7 (m), 129.4 – 129.1 (m), 117.6 – 117.2 (m), 105.6 (dd, *J* = 28.4, 21.1 Hz), 80.0, 53.2, 35.7, 28.5.

^{19}F NMR (470 MHz, $CDCl_3$, 23 ºC, δ): -112.9 – -113.1 (m, 1F), -127.4 – -127.6 (m, 1F), -143.7 – -143.9 (m, 1F).

HRMS-ESI(m/z) calc'd for $C_{14}H_{16}NO_4NaF_3^+$ [M+Na]$^+$, 342.0924; found, 342.0928; deviation 1.39 ppm.

N-Boc-2,3,6-trifluorobenzylamine (27)

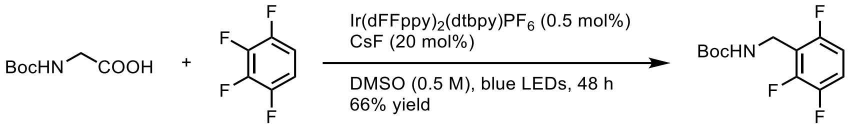

27

Under nitrogen atmosphere, to a 4-mL borosilicate vial equipped with a stir bar was added $Ir(dFFppy)_2(dtbpy)PF_6$ (1.5 mg, 1.5 µmol, 0.50 mol%), CsF (9.2 mg, 60 µmol, 20 mol%), *N*-Boc-glycine (52.6 mg, 0.300 mmol, 1.00 equiv.), dry DMSO (0.6 mL, c = 0.5 M), and 1,2,3,4-tetrafluorobenzene (273 µL, 450 mg, 3.00 mmol, 10.0 equiv.). The vial was sealed with a septum-cap and placed 2 cm away from two 34 W blue LEDs. The temperature was kept at approximately 35 °C through cooling with a fan. After being stirred for 48 hours, the reaction mixture was poured into 20 mL of water, and the resulting mixture was extracted with EtOAc (3 × 20 mL). The combined organic phase was dried over $MgSO_4$, filtered through celite, and concentrated under vacuum. The residue was further purified by flash column chromatography on silica gel, eluting with EtOAc/hexanes 1:10 (v/v) to afford 51.7 mg (66% yield) of the title compound as a colorless solid.

R*f* = 0.23 (EtOAc/hexanes 1:10 (v/v)).

NMR Spectroscopy:

^{1}H NMR (600 MHz, $CDCl_3$, 23 ºC, δ): 7.09 – 7.03 (m, 1H), 6.84 – 6.79 (m, 1H), 4.94 (brs, 1H), 4.42 (m, 2H), 1.42 (s, 9H).

^{13}C NMR (150 MHz, $CDCl_3$, 23 ºC, δ): 156.7 (ddd, *J* = 245.2, 5.9, 2.9 Hz), 155.4, 147.2 (ddd, *J* = 244.9, 13.1, 3.6 Hz), 116.9 – 116.5 (m), 116.4 – 116.1 (m), 111.0 – 110.6 (m), 80.0, 32.9, 28.4.

^{19}F NMR (565 MHz, $CDCl_3$, 23 ºC, δ): -120.3 – -120.4 (m, 1F), -138.0 – -138.1 (m, 1F), -142.0 – -142.4 (m, 1F).

HRMS-ESI(m/z) calc'd for $C_{12}H_{14}F_3NO_2Na^+$ $[M+Na]^+$, 284.0869; found, 284.0872; deviation 0.97 ppm.

N-Boc-2,4,6-trichloro-3,5-difluorobenzylamine (28)

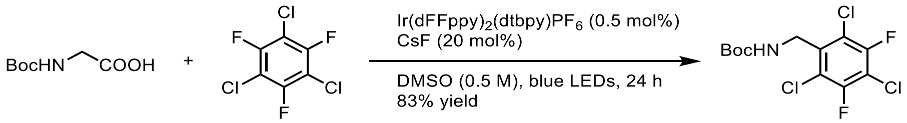

28

Under nitrogen atmosphere, to a 4-mL borosilicate vial equipped with a stir bar was added $Ir(dFFppy)_2(dtbpy)PF_6$ (1.5 mg, 1.5 µmol, 0.50 mol%), CsF (9.2 mg, 60 µmol, 20 mol%), *N*-Boc-glycine (52.6 mg, 0.300 mmol, 1.00 equiv.), dry DMSO (0.6 mL, c = 0.5 M), and 1,3,5-trichloro-2,4,6-trifluorobenzene (141 mg, 0.600 mmol, 2.00 equiv.). The vial was sealed with a septum-cap and placed 2 cm away from two 34 W blue LEDs. The temperature was kept at approximately 35 °C through cooling with a fan. After being stirred for 24 hours, the reaction mixture was poured into 20 mL of water, and the resulting mixture was extracted with EtOAc (3 × 20 mL). The combined organic phase was dried over $MgSO_4$, filtered through celite, and

concentrated under vacuum. The residue was further purified by flash column chromatography on silica gel, eluting with EtOAc/hexanes 1:10 (v/v) to afford 86.3 mg (83% yield) of the title compound as a colorless solid.

R*f* = 0.33 (EtOAc/hexanes 1:10 (v/v)).

NMR Spectroscopy:

^{1}H NMR (500 MHz, $CDCl_3$, 23 ºC, δ): 4.94 (brs, 1H), 4.64 (d, *J* = 6.1 Hz, 2H), 1.43 (s, 9H).

^{13}C NMR (125 MHz, $CDCl_3$, 23 ºC, δ): 155.2, 153.6 (dd, *J* = 251.6, 3.0 Hz), 135.1, 119.3 – 119.0 (m), 111.8 (t, *J* = 22.3 Hz), 80.3, 39.9, 28.5.

^{19}F NMR (470 MHz, $CDCl_3$, 23 ºC, δ): -110.4 (s, 2F).

HRMS-ESI(m/z) calc'd for $C_{12}H_{12}Cl_3F_2NO_2Na^+$ $[M+Na]^+$, 367.9794; found, 367.9800; deviation 1.61 ppm.

N-Boc-4-trifluoromethyl-2,6-difluorobenzylamine (29)

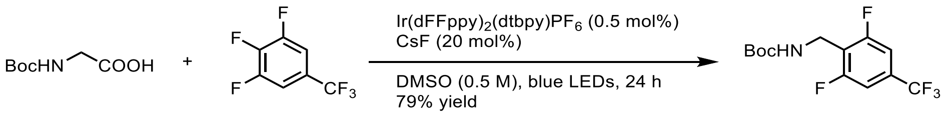

29

Under nitrogen atmosphere, to a 4-mL borosilicate vial equipped with a stir bar was added $Ir(dFFppy)_2(dtbpy)PF_6$ (1.5 mg, 1.5 µmol, 0.50 mol%), CsF (9.2 mg, 60 µmol, 20 mol%), *N*-Boc-glycine (52.6 mg, 0.300 mmol, 1.00 equiv.), dry DMSO (0.6 mL, c = 0.5 M), and 1,2,3-trifluoro-5-(trifluoromethyl)benzene (81.4 µL, 120 mg, 0.600 mmol, 2.00 equiv.). The vial was sealed with a septum-cap and placed 2 cm away from two 34 W blue LEDs. The temperature was kept at approximately 35 °C through cooling with a fan. After being stirred for 24 hours, the reaction mixture was poured into 20 mL of water, and the resulting mixture was extracted with EtOAc (3 × 20 mL). The combined organic phase was dried over $MgSO_4$, filtered through celite, and concentrated under vacuum. The residue was further purified by flash column chromatography on silica gel, eluting with EtOAc/hexanes 1:10 (v/v) to afford 73.7 mg (79% yield) of the title compound as a colorless solid.

R*f* = 0.30 (EtOAc/hexanes 1:10 (v/v)).

NMR Spectroscopy:

^{1}H NMR (500 MHz, $CDCl_3$, 23 ºC, δ): 7.18 – 7.12 (m, 2H), 5.02 (brs, 1H), 4.43 (d, *J* = 6.1 Hz, 2H), 1.41 (s, 9H).

^{13}C NMR (125 MHz, $CDCl_3$, 23 ºC, δ): 161.5 (dd, *J* = 251.6, 8.5 Hz), 155.4, 132.7 – 131.5 (m), 126.6 – 119.3 (m), 118.8 (t, *J* = 19.6 Hz), 109.6 – 108.8 (m), 80.1, 32.6, 28.4.

^{19}F NMR (470 MHz, $CDCl_3$, 23 ºC, δ): -63.1 (s, 3F), -111.5 (s, 2F).

HRMS-ESI(m/z) calc'd for $C_{13}H_{14}NO_2F_5Na^+$ $[M+Na]^+$, 334.0837; found, 334.0840; deviation 0.99 ppm.

N-Boc-4-trifluoromethyl-2,6-dichlorobenzylamine (30)

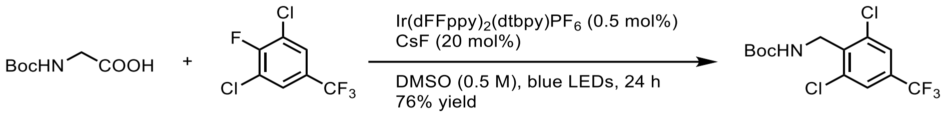

Under nitrogen atmosphere, to a 4-mL borosilicate vial equipped with a stir bar was added Ir(dFFppy)$_2$(dtbpy)PF$_6$ (1.5 mg, 1.5 µmol, 0.50 mol%), CsF (9.2 mg, 60 µmol, 20 mol%), *N*-Boc-glycine (52.6 mg, 0.300 mmol, 1.00 equiv.), dry DMSO (0.6 mL, c = 0.5 M), and 1,3-dichloro-2-fluoro-5-(trifluoromethyl)benzene (90.0 µL, 140 mg, 0.600 mmol, 2.00 equiv.). The vial was sealed with a septum-cap and placed 2 cm away from two 34 W blue LEDs. The temperature was kept at approximately 35 °C through cooling with a fan. After being stirred for 24 hours, the reaction mixture was poured into 20 mL of water, and the resulting mixture was extracted with EtOAc (3 × 20 mL). The combined organic phase was dried over MgSO$_4$, filtered through celite, and concentrated under vacuum. The residue was further purified by flash column chromatography on silica gel, eluting with EtOAc/hexanes 1:10 (v/v) to afford 78.6 mg (76% yield) of the title compound as a colorless solid.

R*f* = 0.30 (EtOAc/hexanes 1:10 (v/v)).

NMR Spectroscopy:

^{1}H NMR (500 MHz, CDCl$_3$, 23 ºC, δ): 7.57 (s, 2H), 4.93 (brs, 1H), 4.67 (d, *J* = 6.1 Hz, 2H), 1.44 (s, 9H).

^{13}C NMR (125 MHz, CDCl$_3$, 23 ºC, δ): 155.2, 138.1 (q, *J* = 1.2 Hz), 136.8, 131.9 (q, *J* = 34.1 Hz), 125.4 (q, *J* = 3.7 Hz), 122.4 (q, *J* = 273.0 Hz), 79.9, 40.0, 28.3.

^{19}F NMR (470 MHz, CDCl$_3$, 23 ºC, δ): -63.1 (s, 3F).

HRMS-ESI(m/z) calc'd for $C_{13}H_{14}NO_2Cl_2F_3Na^+$ [M+Na]$^+$, 366.0246; found, 366.0248; deviation 0.63 ppm.

N-Boc-allyl-tetrafluorobenzylamines 31a, 31b, 31c

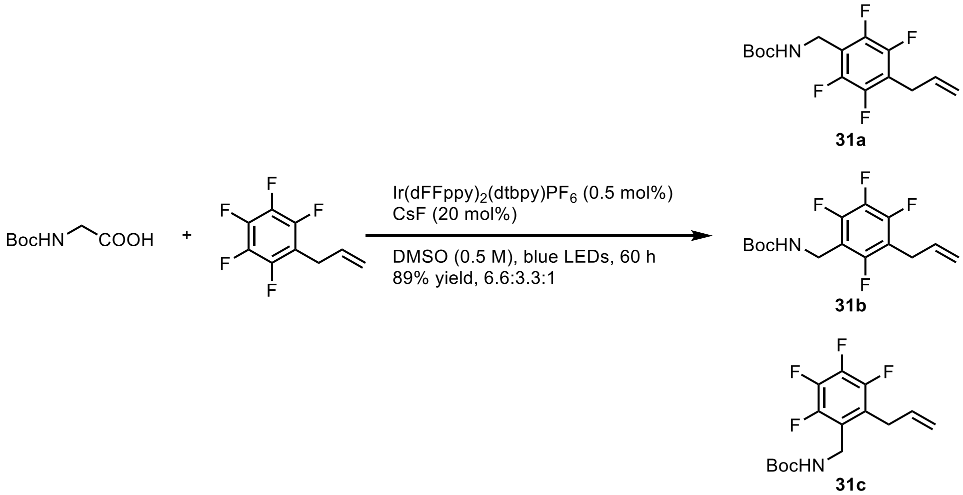

Under nitrogen atmosphere, to a 4-mL borosilicate vial equipped with a stir bar was added

$Ir(dFFppy)_2(dtbpy)PF_6$ (1.5 mg, 1.5 µmol, 0.50 mol%), CsF (9.2 mg, 60 µmol, 20 mol%), *N*-Boc-glycine (52.6 mg, 0.300 mmol, 1.00 equiv.), dry DMSO (0.6 mL, c = 0.5 M), and perfluoro-allylbenzene (626 mg, 3.00 mmol, 10.0 equiv.). The vial was sealed with a septum-cap and placed 2 cm away from two 34 W blue LEDs. The temperature was kept at approximately 35 °C through cooling with a fan. After being stirred for 60 hours, the reaction mixture was poured into 20 mL of water, and the resulting mixture was extracted with EtOAc (3 × 20 mL). The combined organic phase was dried over $MgSO_4$, filtered through celite, and concentrated under vacuum. The residue was further purified by flash column chromatography on silica gel, eluting with EtOAc/hexanes 1:10 (v/v) to afford 85.2 mg (89% yield) of the mixture of **31a**, **31b**, and **31c**. The ratio of them is 6.6:3.3:1 determined by the ^{19}F NMR of the reaction. Further purification by HPLC (YMC-Actus Triart C18 (150 × 30.0 mm: 5 µm) MeCN/Water = 50:50, flow rate = 42.5 mL/min, 35 °C) provided pure **31a** as a colorless solid, **31b** as a colorless solid, and **31c** as a colorless solid.

The following data was obtained for the pure isomers:

***N*-Boc-4-allyl-2,3,5,6-tetrafluorobenzylamine (31a):**

R*f* = 0.35 (EtOAc/hexanes 1:10 (v/v)).

NMR Spectroscopy:

^{1}H NMR (600 MHz, $CDCl_3$, 23 ºC, δ): 5.88 (ddt, *J* = 16.6, 10.1, 6.3 Hz, 1H), 5.11 – 5.05 (m, 2H), 4.93 (brs, 1H), 4.44 (d, *J* = 6.0 Hz, 2H), 3.51 – 3.40 (m, 2H), 1.43 (s, 9H).

^{13}C NMR (150 MHz, $CDCl_3$, 23 ºC, δ): 155.4, 145.9 – 143.8 (m, 2C), 133.2, 117.9 (t, *J* = 18.3 Hz), 117.2, 115.6 – 114.8 (m), 80.2, 32.9, 28.5, 27.0 – 26.9 (m).

^{19}F NMR (470 MHz, $CDCl_3$, 23 ºC, δ): -144.6 (dd, J = 22.3, 12.9 Hz, 2F), -144.8 (dd, J = 21.9, 12.7 Hz, 2F).

HRMS-ESI(m/z) calc'd for $C_{15}H_{17}NO_2F_4Na^+$ $[M+Na]^+$, 342.1088; found, 342.1091; deviation 0.93 ppm.

***N*-Boc-5-allyl-2,3,4,6-tetrafluorobenzylamine (31b):**

R*f* = 0.35 (EtOAc/hexanes 1:10 (v/v)).

NMR Spectroscopy:

^{1}H NMR (500 MHz, $CDCl_3$, 23 ºC, δ): 5.87 (ddt, *J* = 16.6, 10.1, 6.3 Hz, 1H), 5.10 – 5.02 (m, 2H), 4.88 (brs, 1H), 4.45 – 4.34 (m, 2H), 3.39 (dt, *J* = 6.3, 1.6 Hz, 2H), 1.43 (s, 9H).

^{13}C NMR (150 MHz, $CDCl_3$, 23 ºC, δ): 155.4, 155.1 – 152.9 (m), 150.7 – 147.8 (m), 149.1 – 147.1 (m), 139.0 – 135.6 (m), 133.6, 116.8, 113.4 – 112.6 (m), 80.1, 32.7, 28.5, 26.6. (One carbon signal was missing because of overlap.)

^{19}F NMR (470 MHz, $CDCl_3$, 23 ºC, δ): -124.5 (d, *J* = 11.5 Hz, 1F), -137.9 (d, *J* = 21.2 Hz, 1F), -139.7 (d, *J* = 21.6 Hz, 1F), -165.0 (td, *J* = 21.4, 11.4 Hz, 1F).

HRMS-ESI(m/z) calc'd for $C_{15}H_{17}NO_2F_4Na^+$ $[M+Na]^+$, 342.1088; found, 342.1092; deviation 1.16 ppm.

_N_-Boc-6-allyl-2,3,4,5-tetrafluorobenzylamine (31c):

R_f = 0.35 (EtOAc/hexanes 1:10 (v/v)).

NMR Spectroscopy:

^{1}H NMR (600 MHz, $CDCl_3$, 23 ºC, δ): 5.90 (ddt, J = 17.1, 10.1, 5.8 Hz, 1H), 5.11 – 5.04 (m, 1H), 4.97 – 4.87 (m, 1H), 4.78 (brs, 1H), 4.39 – 4.25 (m, 2H), 3.62 – 3.53 (m, 2H), 1.43 (s, 9H).

^{13}C NMR (150 MHz, $CDCl_3$, 23 ºC, δ): 155.4, 147.7 – 145.8 (m), 146.9 – 145.1 (m), 141.3 – 139.0 (m), 140.0 – 138.1 (m), 134.8, 123.3 – 122.4 (m), 121.8 – 120.9 (m), 116.5, 80.1, 34.9, 29.1, 28.5.

^{19}F NMR (470 MHz, $CDCl_3$, 23 ºC, δ): -142.2 (dd, J = 21.3, 12.2 Hz, 1F), -143.5 (dd, J = 21.4, 12.4 Hz, 1F), -156.8 (t, J = 20.8 Hz, 1F), -158.6 (t, J = 20.8 Hz, 1F).

HRMS-ESI(m/z) calc'd for $C_{15}H_{17}NO_2F_4Na^+$ $[M+Na]^+$, 342.1088; found, 342.1091; deviation 0.84 ppm.

_N_-Boc-tetrafluoro-(pentafluorophenyl)benzylamines 32a, 32b

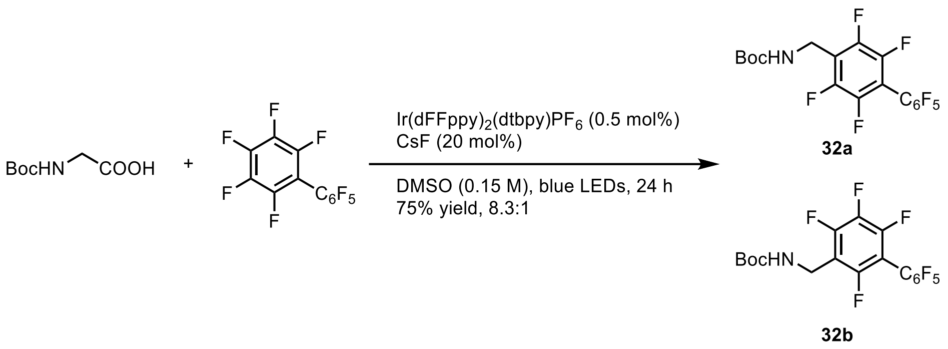

Under nitrogen atmosphere, to a 4-mL borosilicate vial equipped with a stir bar was added $Ir(dFFppy)_2(dtbpy)PF_6$ (1.5 mg, 1.5 µmol, 0.50 mol%), CsF (9.2 mg, 60 µmol, 20 mol%), _N_-Boc-glycine (52.6 mg, 0.300 mmol, 1.00 equiv.), dry DMSO (2.0 mL, c = 0.15 M), and perfluorobiphenyl (201 mg, 0.600 mmol, 2.00 equiv.). The vial was sealed with a septum-cap and placed 2 cm away from two 34 W blue LEDs. The temperature was kept at approximately 35 °C through cooling with a fan. After being stirred for 24 hours, the reaction mixture was poured into 20 mL of water, and the resulting mixture was extracted with EtOAc (3 × 20 mL). The combined organic phase was dried over $MgSO_4$, filtered through celite, and concentrated under vacuum. The residue was further purified by flash column chromatography on silica gel, eluting with EtOAc/hexanes 1:10 (v/v) to afford 100.3 mg (75% yield) of the mixture of **32a** and **32b**. The ratio of them is 8.3:1 determined by the ^{19}F NMR of the reaction. Further purification by HPLC (PVA-Sil NP (250 × 4.6 mm: 5 µm) isohexane/isopropanol = 99:1, flow rate = 1.0 mL/min, 25 °C) provided pure **32a** and **32b** as colorless solids.

The following data was obtained for the pure isomers:

_N_-Boc-2,3,5,6-tetrafluoro-4-(pentafluorophenyl)benzylamine (32a):

R_f = 0.20 (EtOAc/hexanes 1:10 (v/v)).

NMR Spectroscopy:

^{1}H NMR (500 MHz, $CDCl_3$, 23 ºC, δ): 5.08 (brs, 1H), 4.56 – 4.51 (m, 2H), 1.44 (s, 9H).

^{13}C NMR (126 MHz, $CDCl_3$, 23 ºC, δ): 155.4, 145.8 – 143.5 (m), 146.4 – 144.1 (m), 145.3 – 142.8 (m), 143.7 – 141.4 (m), 139.2 – 136.8 (m), 120.1 – 119.7 (m), 105.9 – 105.5 (m), 102.7 – 102.3 (m), 80.5, 33.1, 28.4.

^{19}F NMR (470 MHz, $CDCl_3$, 23 ºC, δ): -137.3 – -137.5 (m, 2F), -138.3 – -138.5 (m, 2F), -142.5 (dd, *J* = 22.1, 12.7 Hz, 2F), -150.4 (t, *J* = 20.9 Hz, 1F), -160.7 (td, *J* = 21.7, 7.7 Hz, 2F).

HRMS-ESI(m/z) calc'd for $C_{18}H_{11}NO_2F_9^-$ [M-H]$^-$, 444.0652; found, 444.0655; deviation 0.72 ppm.

***N*-Boc-2,3,4,6-tetrafluoro-5-(pentafluorophenyl)benzylamine (32b):**

R*f* = 0.20 (EtOAc/hexanes 1:10 (v/v)).

NMR Spectroscopy:

^{1}H NMR (500 MHz, $CDCl_3$, 23 ºC, δ): 4.94 (brs, 1H), 4.47 – 4.44 (m, 2H), 1.44 (s, 9H).

^{13}C NMR (150 MHz, $CDCl_3$, 23 ºC, δ): 155.3, 154.5 – 152.4 (m), 152.1 – 150.1 (m), 149.5 – 147.4 (m), 145.8 – 143.8 (m), 143.4 – 141.3 (m), 138.6 – 136.6 (m), 139.0 – 137.0 (m), 113.2 – 112.8 (m), 103.0 – 102.4 (m), 101.7 – 100.9 (m), 80.4, 32.7, 28.4.

^{19}F NMR (470 MHz, $CDCl_3$, 23 ºC, δ): -118.2 – -118.4 (m, 1F), -131.6 – -131.7 (m, 1F), -131.9 – -132.1 (m, 1F), -137.4 – -137.6 (m, 2F), -150.8 (t, *J* = 20.7 Hz, 1F), -160.7 – -161.0 (m, 2F), -162.5 – -162.7 (m, 1F).

HRMS-ESI(m/z) calc'd for $C_{18}H_{12}NO_2F_9Na^+$ [M+Na]$^+$, 468.0617; found, 468.0619; deviation 0.57 ppm.

N-Boc-tetrafluoro-((1'R,2'S,5'R)-2'-isopropyl-5'-methylcyclohexylcarboxyl)benzylamines 33a, 33b

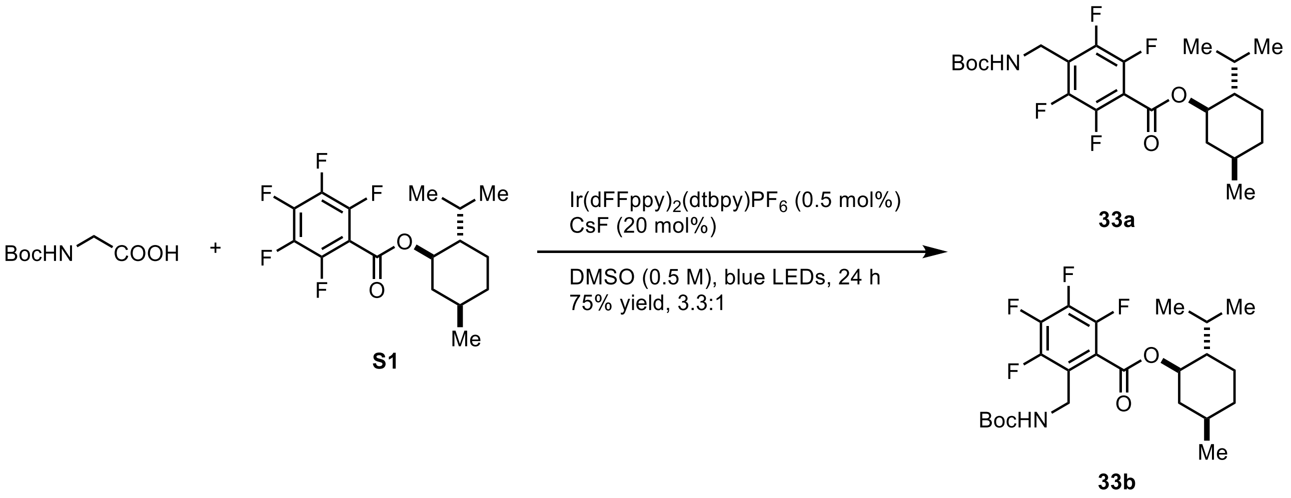

Under nitrogen atmosphere, to a 4-mL borosilicate vial equipped with a stir bar was added $Ir(dFFppy)_2(dtbpy)PF_6$ (1.5 mg, 1.5 µmol, 0.50 mol%), CsF (9.2 mg, 60 µmol, 20 mol%), *N*-Boc-glycine (52.6

mg, 0.300 mmol, 1.00 equiv.), dry DMSO (2.0 mL, c = 0.15 M), and **S1** (105 mg, 0.300 mmol, 1.00 equiv.). The vial was sealed with a septum-cap and placed 2 cm away from two 34 W blue LEDs. The temperature was kept at approximately 35 °C through cooling with a fan. After being stirred for 24 hours, the reaction mixture was poured into 20 mL of water, and the resulting mixture was extracted with EtOAc (3 × 20 mL). The combined organic phase was dried over $MgSO_4$, filtered through celite, and concentrated under vacuum. The residue was further purified by flash column chromatography on silica gel, eluting with EtOAc/hexanes 1:20 (v/v) to afford 24.1 mg of **33b** as a colorless solid and 80.0 mg of **33a** as a colorless solid. (Total yield is 75%. The ratio of them is 3.3:1 determined by the ^{19}F NMR of the reaction.)

The following data was obtained for the pure isomers:

***N*-Boc-2,3,5,6-tetrafluoro-4-((1'R,2'S,5'R)-2'-isopropyl-5'-methylcyclohexylcarboxyl)benzylamine (33a):**

R*f* = 0.20 (EtOAc/hexanes 1:20 (v/v)).

NMR Spectroscopy:

^{1}H NMR (500 MHz, $CDCl_3$, 23 ºC, δ): 5.09 (t, *J* = 6.3 Hz, 1H), 4.96 (td, *J* = 10.9, 4.4 Hz, 1H), 4.44 (d, *J* = 6.3 Hz, 2H), 2.18 – 2.10 (m, 1H), 2.01 – 1.91 (m, 1H), 1.75 – 1.66 (m, 2H), 1.57 – 1.42 (m, 3H), 1.41 (s, 9H), 1.16 – 1.02 (m, 2H), 0.92 (d, *J* = 6.6 Hz, 3H), 0.89 (d, *J* = 7.0 Hz, 3H), 0.78 (d, *J* = 7.0 Hz, 3H).

^{13}C NMR (125 MHz, $CDCl_3$, 23 ºC, δ): 159.3, 155.3, 146.2 – 144.0 (m), 145.3 – 143.0 (m), 120.4 – 119.8 (m), 113.1 (t, *J* = 16.9 Hz), 80.3, 77.6, 47.0, 40.7, 34.2, 33.0, 31.6, 28.4, 26.0, 23.2, 22.0, 20.9, 16.0.

^{19}F NMR (470 MHz, $CDCl_3$, 23 ºC, δ): -140.0 – -140.2 (m, 2F), -142.6 – -142.7 (m, 2F).

HRMS-ESI(m/z) calc'd for $C_{23}H_{31}F_4NO_4Na^+$ $[M+Na]^+$, 484.2081; found, 484.2081; deviation 0.02 ppm.

***N*-Boc-2,3,4,5-tetrafluoro-6-((1'R,2'S,5'R)-2'-isopropyl-5'-methylcyclohexylcarboxyl)benzylamine (33b):**

R*f* = 0.40 (EtOAc/hexanes 1:20 (v/v)).

NMR Spectroscopy:

^{1}H NMR (600 MHz, $CDCl_3$, 23 ºC, δ): 5.22 (brs, 1H), 4.99 (td, *J* = 10.9, 4.4 Hz, 1H), 4.48 – 4.32 (m, 2H), 2.19 – 2.11 (m, 1H), 2.03 – 1.95 (m, 1H), 1.77 – 1.69 (m, 2H), 1.64 – 1.60 (m, 1H), 1.60 – 1.45 (m, 2H), 1.41 (s, 9H), 1.19 – 1.05 (m, 2H), 0.95 (d, *J* = 6.6 Hz, 3H), 0.92 (d, *J* = 7.1 Hz, 3H), 0.81 (d, *J* = 6.9 Hz, 3H).

^{13}C NMR (150 MHz, $CDCl_3$, 23 ºC, δ): 163.4, 155.3, 147.1 – 145.0 (m), 143.1 – 141.1 (m), 141.1 – 139.2 (m), 123.1 – 122.3 (m), 118.2 – 117.5 (m), 80.0, 77.7, 47.0, 40.7, 35.3, 34.2, 31.7, 28.5, 26.0, 23.1, 22.1, 21.0, 16.0. (One carbon signal was missing because of overlap.)

^{19}F NMR (470 MHz, $CDCl_3$, 23 ºC, δ): -137.2 – -137.5 (m, 1F), -139.4 – -139.8 (m, 1F), -150.3 – -150.7 (m, 1F), -154.9 – -155.2 (m, 1F).

HRMS-ESI(m/z) calc'd for $C_{23}H_{31}NO_4F_4Na^+$ $[M+Na]^+$, 484.2081; found, 484.2081; deviation 0.15 ppm.

Mechanism study

Proposed possible mechanism

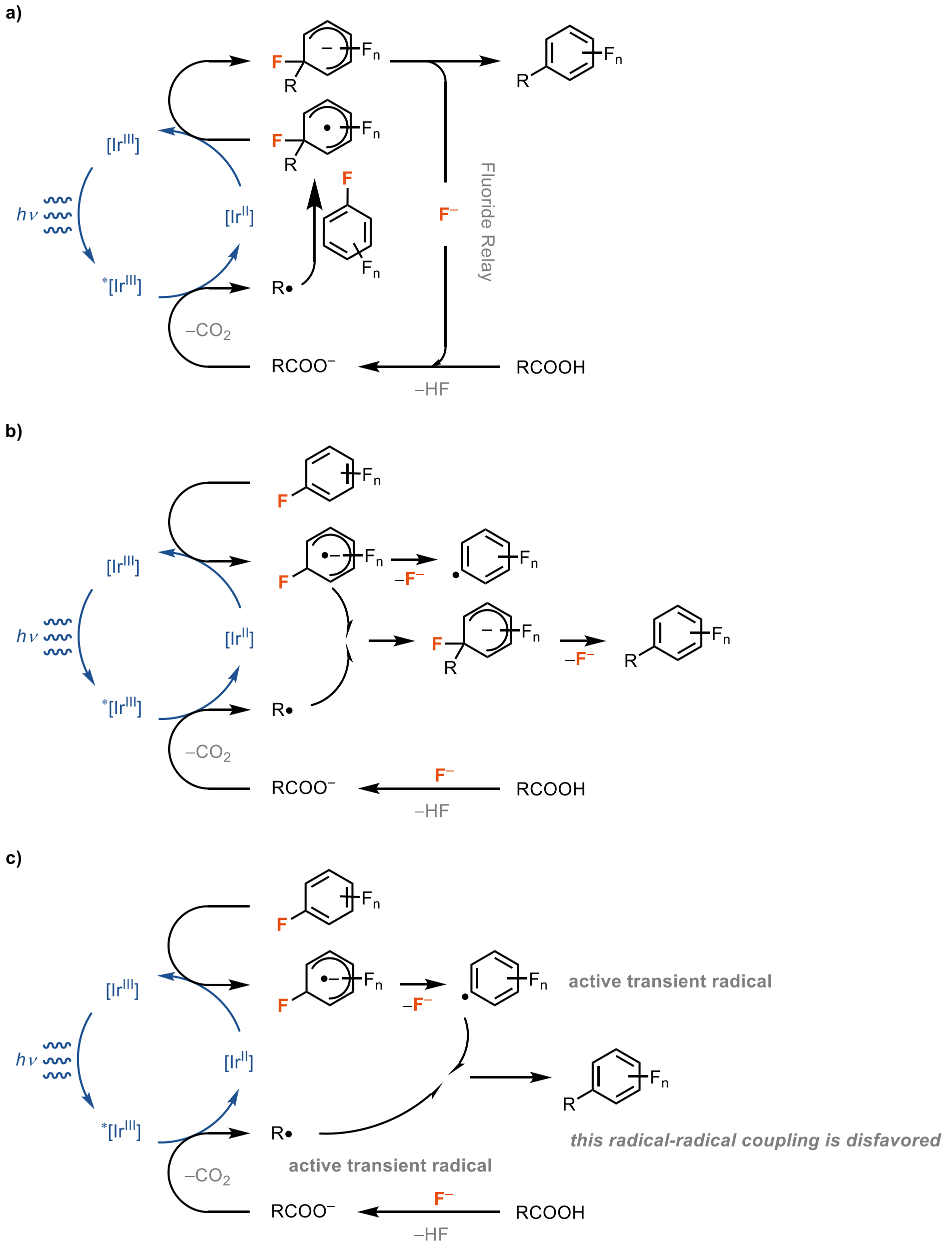

Scheme S1. Three possible mechanisms. a) Direct radical addition to polyfluoroarene. b) Radical coupling between alkyl radical and polyfluoroaryl radical anion. c) Radical coupling between alkyl radical and polyfluoroaryl radical.

Pathway b is disfavored: First, the single electron reduction of perfluoroarene by the iridium photocatalyst is not likely to happen in consideration of their redox potentials ($E_{red}(C_6F_6)$ = -2.85 V *vs.* SCE in THF, $E_{ox}(Ir^{III}/Ir^{II})$ = -1.32 V *vs.* SCE in MeCN). Second, the generated radical anion of pentafluoropyridine should be more stable than the radical anion generated from hexafluorobenzene and should offer a better yield. However, in our reaction, pentafluoropyridine is an unsuccessful substrate with mess outcome. Third, no polyfluoroaryl radical

which can be generated from the polyfluoroaryl radical anion was trapped by ^{t}Bu-acetylene, norbornene, or 1,3,5-trimethoxybenzene.

Pathway c is disfavored: First, the radical-radical coupling between two active transient radicals is disfavored. Second, the single electron reduction of perfluoroarene by the iridium photocatalyst is not likely to happen in consideration of their redox potentials ($E_{red}(C_6F_6)$ = -2.85 V *vs.* SCE in THF, $E_{ox}(Ir^{III}/Ir^{II})$ = -1.32 V *vs.* SCE in MeCN). Third, no polyfluoroaryl radical was trapped by ^{t}Bu-acetylene, norbornene, or 1,3,5-trimethoxybenzene. Last, if polyfluoroaryl radical is generated, in the reaction of chloro-fluoro arenes, the chlorine substitution should be observed. However, in our reaction, the exclusive fluorine substitution is observed.

Evidence for carbon radical

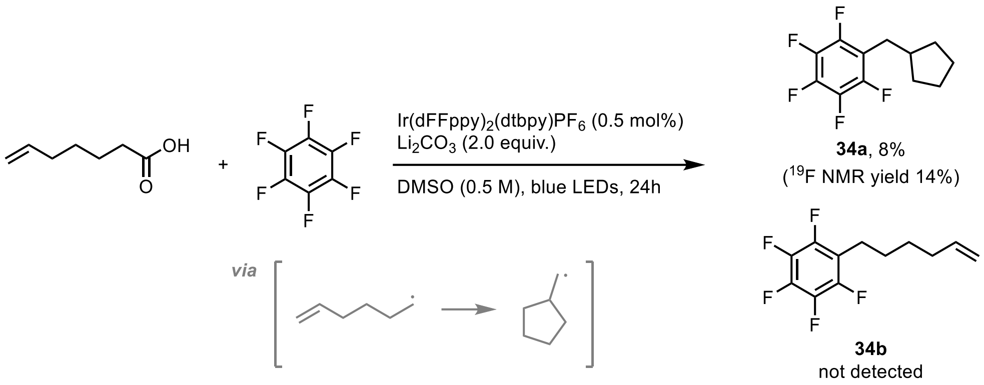

Under nitrogen atmosphere, to a 4-mL borosilicate vial equipped with a stir bar was added Ir(dFFppy)$_2$(dtbpy)PF$_6$ (1.5 mg, 1.5 µmol, 0.50 mol%), Li_2CO_3 (44.3 mg, 0.600 mmol, 2.00 equiv.), 6-heptenoic acid (38.5 mg, 0.300 mmol, 1.00 equiv.), dry DMSO (0.6 mL, c = 0.5 M), and perfluorobenzene (347 µL, 558 mg, 3.00 mmol, 10.0 equiv.). The vial was sealed with a septum-cap and placed 2 cm away from two 34 W blue LEDs. The temperature was kept at approximately 35 °C through cooling with a fan. After being stirred for 24 hours, the reaction mixture was further purified by flash column chromatography on silica gel, eluting with hexanes to afford 6.0 mg (8% yield) of **34a** as a colorless liquid. No **34b** was detected.

Cyclopentylmethyl-pentafluorobenzene (34a):

R*f* = 0.70 (hexanes).

NMR Spectroscopy:

^{1}H NMR (600 MHz, $CDCl_3$, 23 ºC, δ): 2.69 (dt, *J* = 7.6, 1.9 Hz, 2H), 2.13 – 2.04 (m, 1H), 1.73 – 1.64 (m, 4H), 1.60 – 1.50 (m, 2H), 1.24 – 1.17 (m, 2H).

^{13}C NMR (150 MHz, $CDCl_3$, 23 ºC, δ): 146.4 – 144.1 (m), 140.7 – 138.4 (m), 138.5 – 136.3 (m), 115.5 – 115.0 (m), 40.3, 32.3, 27.9 (d, *J* = 1.7 Hz), 24.9.

^{19}F NMR (565 MHz, $CDCl_3$, 23 ºC, δ): -143.5 – -143.6 (m, 2F), -158.4 (t, *J* = 20.7 Hz, 1F), -163.1 – -163.3 (m, 2F).

HRMS-EI(m/z) calc'd for $C_{12}H_{11}F_5^+$ $[M]^+$, 250.0775; found, 250.0777; deviation 0.43 ppm.

Experiments to trap perfluorophenyl radical

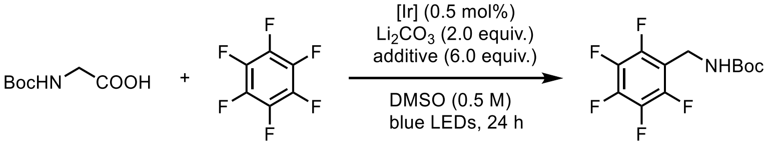

additives	proposed radical adduct		^{19}F NMR yield of **22**
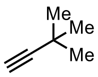	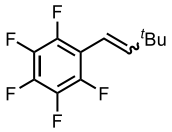	not detected	78%
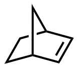	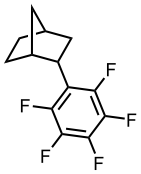	not detected	95%
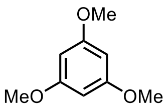	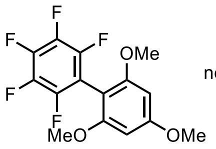	not detected	87%

Scheme S2. Experiments to trap perfluorophenyl radicl. Reaction conditions: *N*-Boc glycine (0.3 mmol), perfluorobenzene (0.6 mmol), Ir(dFFppy)$_2$(dtbpy)PF$_6$ (0.5 mol%), Li_2CO_3 (2.0 equiv.), additives (6.0 equiv.), DMSO (0.6 mL), 35 °C, blue LEDs, 24 h.

Quantum yield measurement

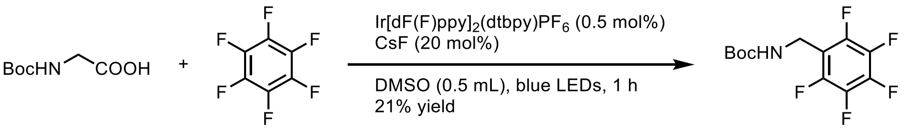

22

Under nitrogen atmosphere, to a 4-mL borosilicate vial equipped with a stir bar was added Ir(dFFppy)$_2$(dtbpy)PF$_6$ (1.5 mg, 1.5 µmol, 0.50 mol%), CsF (9.2 mg, 60 µmol, 20 mol%), *N*-Boc-glycine (52.6 mg, 0.300 mmol, 1.00 equiv.), dry DMSO (0.5 mL), and perfluorobenzene (70.0 µL, 112 mg, 0.600 mmol, 2.00 equiv.). The vial was sealed with a septum-cap and placed 2 cm away from two 34 W blue LEDs. The temperature was kept at approximately 35 °C through cooling with a fan. After irradiation for 1 hour, 4-methoxy-fluorobenzene (34.1 µL, 37.8 mg, 0.300 mmol, 1.00 equiv.) and ethyl acetate (2 mL) were added. The mixture was stirred vigorously for 5 min. The yield of product was determined by ^{19}F NMR without deuterated solvents. Comparison of the integration of the F of 4-methoxy-fluorobenzene (δ = -126, 1F) with the F of the product (δ = -144, 2H) revealed 21% yield of **22** (6.3×10^{-5} mol).

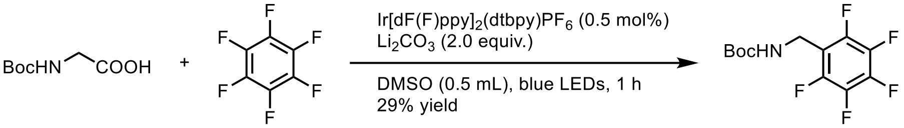

Under nitrogen atmosphere, to a 4-mL borosilicate vial equipped with a stir bar was added Ir(dFFppy)$_2$(dtbpy)PF$_6$ (1.5 mg, 1.5 µmol, 0.50 mol%), Li_2CO_3 (44.3 mg, 0.600 mmol, 2.00 equiv.), *N*-Boc-glycine (52.6 mg, 0.300 mmol, 1.00 equiv.), dry DMSO (0.5 mL), and perfluorobenzene (70.0 µL, 112 mg, 0.600 mmol, 2.00 equiv.). The vial was sealed with a septum-cap and placed 2 cm away from two 34 W blue LEDs. The temperature was kept at approximately 35 °C through cooling with a fan. After irradiation for 1 hour, 4-methoxy-fluorobenzene (34.1 µL, 37.8 mg, 0.300 mmol, 1.00 equiv.) and ethyl acetate (2 mL) were added. The mixture was stirred vigorously for 5 min. The yield of product was determined by ^{19}F NMR without deuterated solvents. Comparison of the integration of the F of 4-methoxy-fluorobenzene (δ = -126, 1F) with the F of the product (δ = -144, 2H) revealed 29% yield of **22** (8.7×10^{-5} mol).

The quantum yield was calculated as follows:

$$\Phi = \frac{\text{mol pruduct}}{\text{flux} \times t \times f}$$

where flux is the photon flux determined by ferrioxalate actinometry[4-6] (1.16×10^{-6} Einstein/s), t is the time (3600 s), and f is the fraction of light absorbed by Ir(dFFppy)$_2$(dtbpy)PF$_6$ at 450 nm. (A 1×10^{-3} M solution of Ir(dFFppy)$_2$(dtbpy)PF$_6$ in DMSO was prepared, and the absorbance of the solution at 450 nm was 0.316. The fraction of light absorbed at 450 nm was calculated: $f = 1.0000 - 10^{-A} = 1.0000 - 10^{-0.316} = 0.517$.)

$$\Phi \text{ (with CsF)} = \frac{6.3 \times 10^{-5}\text{ mol}}{1.16 \times 10^{-6}\text{ Einstein/s} \times 3600\text{ s} \times 0.517} = 0.029$$

$$\Phi \text{ (with } Li_2CO_3\text{)} = \frac{8.7 \times 10^{-5}\text{ mol}}{1.16 \times 10^{-6}\text{ Einstein/s} \times 3600\text{ s} \times 0.517} = 0.040$$

Stern-Volmer quenching experiment

Visible light luminescence intensity was recorded using an Edinburgh Instruments FS5 spectrofluorometer with a screw-top quartz cuvette (Hellma fluorescence quartz cuvette, 10 x 10 mm, 3.5 mL). All solution of Ir(dFFppy)$_2$(dtbpy)PF$_6$, perfluorobenzene, lithium *N*-Boc glycinate, and the mixture of perfluorobenzene and lithium *N*-Boc glycinate was prepared in DMSO in a nitrogen-filled glovebox. The solution was transferred to the screw-top cuvette inside the glovebox, and the cuvette was sealed and brought out of the glovebox for visible light luminescence measurements.

The quenching experiment was conducted by plotting the graph of the I$_0$/I versus different concentration of quencher according to the Stern-Volmer kinetics:

$$\frac{I_0}{I} = k_q \times \tau_0 \times [\text{quencher}] + 1$$

Where I$_0$ is the luminescence intensity without quencher, I is the luminescence intensity with quencher, K$_q$ is the quenching rate constant, τ_0 is the excited state lifetime of the photocatalyst in the absence of quencher, and [quencher] is the concentration of the given quencher.

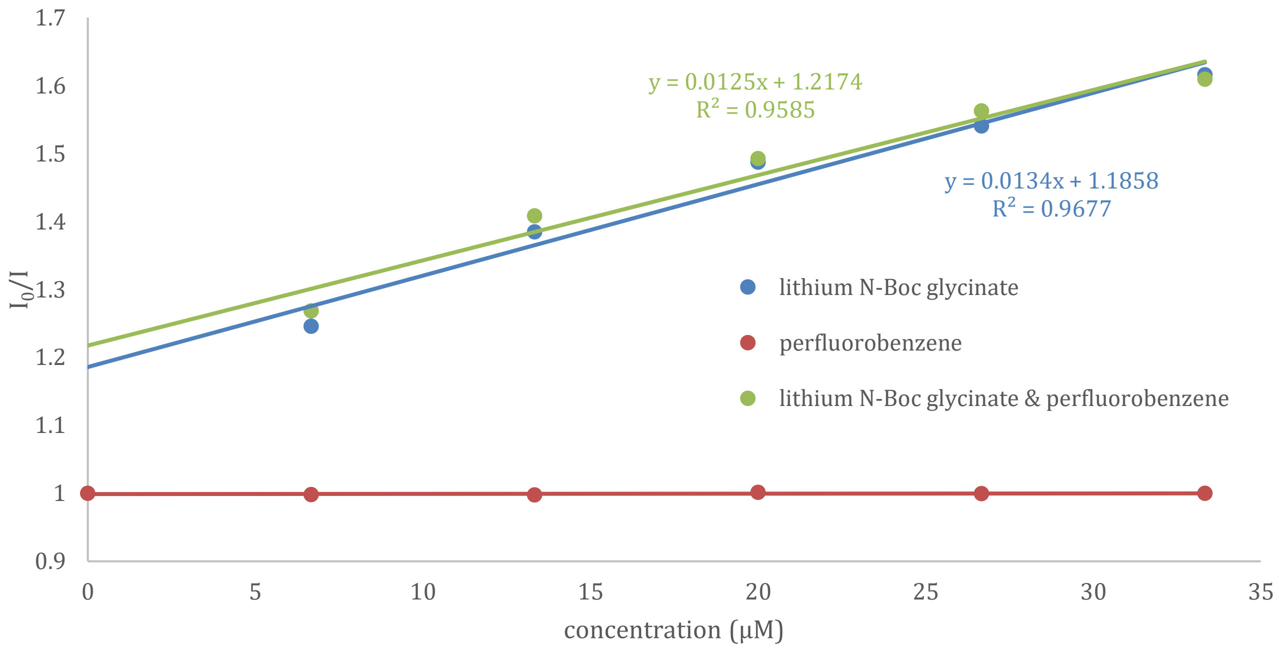

Scheme S3. Stern-Volmer quenching experiment of Ir(dFFppy)$_2$(dtbpy)PF$_6$ with different quenchers.

Cyclic voltammograms of Ir(dFFppy)$_2$(dtbpy)PF_6

Cyclic voltammograms were recorded using an Autolab PGSTAT204 potentiostat, a Pt working electrode, a Ag/AgCl reference electrode, and a Pt sheet auxiliary electrode.

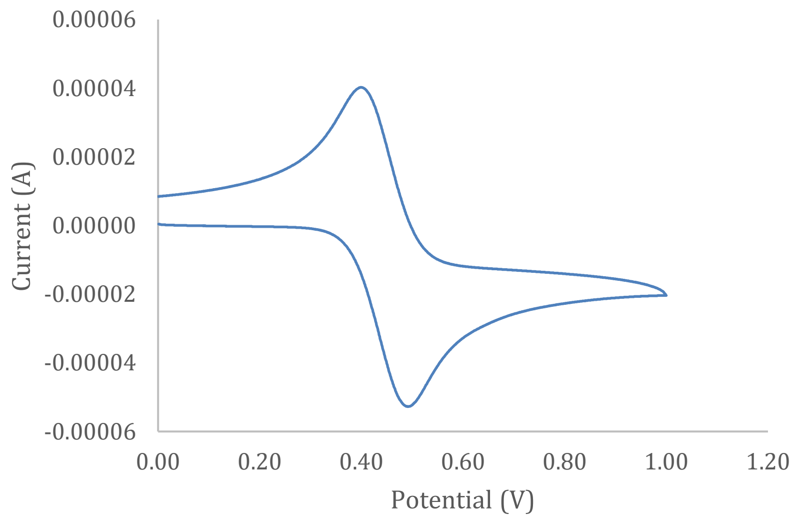

Scheme S4. Cyclic Voltammetry for oxidation of ferrocene.

The voltammograms were recorded at room temperature in 0.1 M tetrabutylammonium hexafluorophosphate in MeCN (3 mL) containing ferrocene (0.6 mg, 0.003 mmol). The scan rate was 100 mV s^{-1}. The potential of ferrocenium/ferrocene couple was found to be 0.45 V vs SCE, in agreement with the value reported in literature (in MeCN).[7]

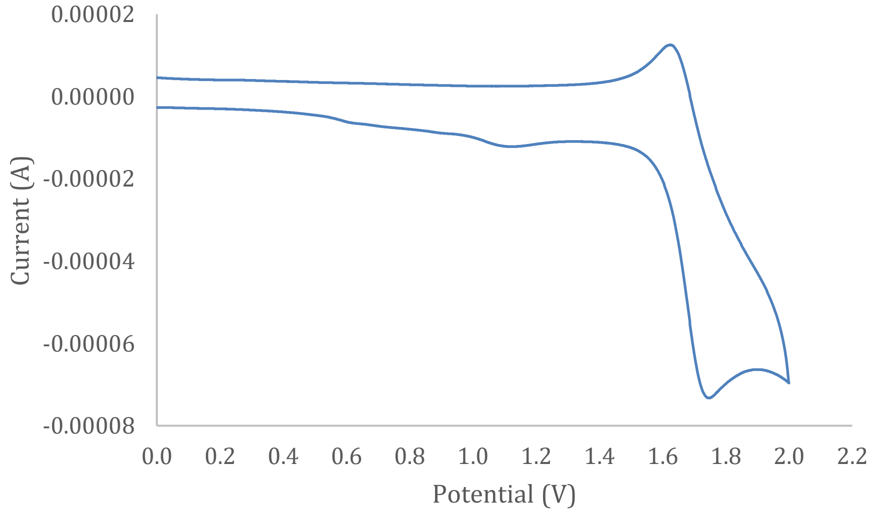

Scheme S5. Anodic Cyclic Voltammetry for Ir(dFFppy)$_2$(dtbpy)PF_6 in MeCN.

The voltammograms were recorded at room temperature in 0.1 M tetrabutylammonium hexafluorophosphate in MeCN (3 mL) containing Ir(dFFppy)$_2$(dtbpy)PF_6 (6 mg, 0.006 mmol). The scan rate was 100 mV s^{-1}. $E_{1/2}$(Ir^{IV}/Ir^{III}) = 1.63 V *vs* SCE.

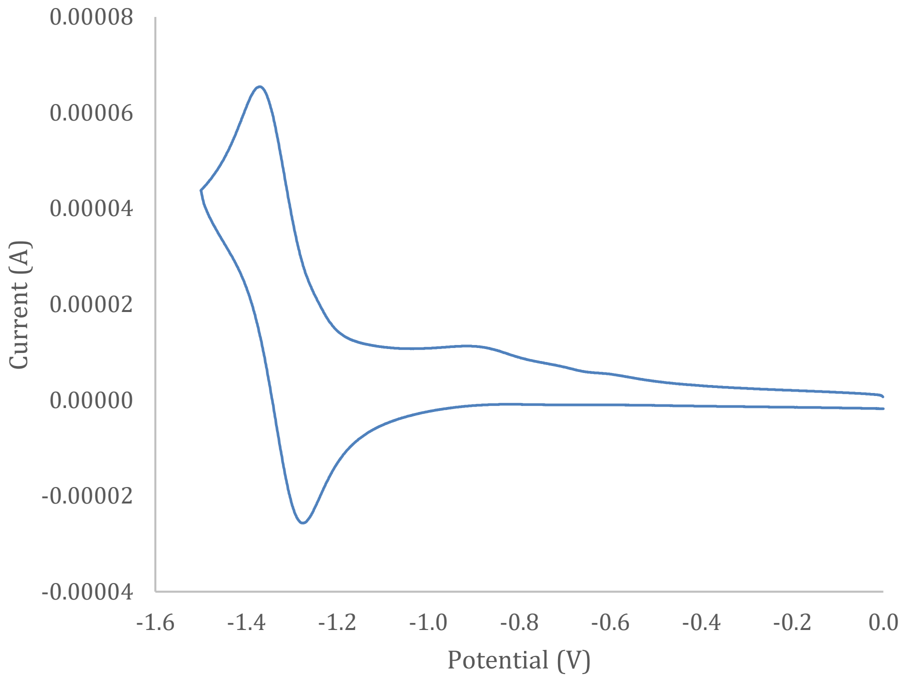

Scheme S6. Cathodic Cyclic Voltammetry of Ir(dFFppy)$_2$(dtbpy)PF$_6$ in MeCN.

The voltammograms were recorded at room temperature in 0.1 M tetrabutylammonium hexafluorophosphate in MeCN (3 mL) containing Ir(dFFppy)$_2$(dtbpy)PF$_6$ (6 mg, 0.006 mmol). The scan rate was 100 mV s^{-1}. $E_{1/2}$(Ir^{III}/Ir^{II}) = -1.32 V *vs* SCE.

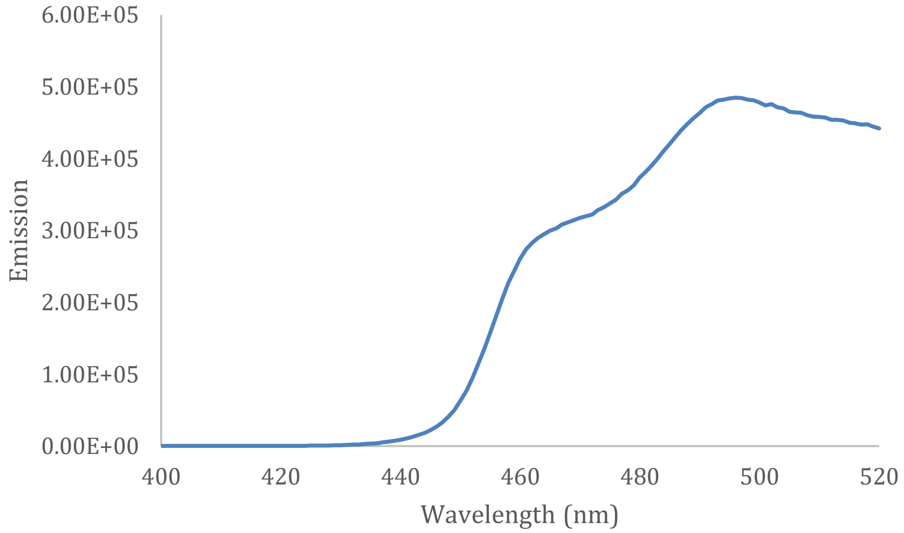

Scheme S7. Emission spectrum of Ir(dFFppy)$_2$(dtbpy)PF$_6$ in MeCN (λ_{ex}=380 nm).

$E_{0,0}$ of Ir(dFFppy)$_2$(dtbpy)PF$_6$ was estimated to be 2.80 eV by selecting the energy at the onset of fluorescence (440 nm). Then, the redox potentials of excited state of Ir(dFFppy)$_2$(dtbpy)PF$_6$ was calculated.[8,9] E_{ox}(Ir^{IV}/*Ir^{III}) = -1.17 V, E_{red}(*Ir^{III}/Ir^{II}) = 1.48 V.

Investigation of the low yield of primary carboxylic acid

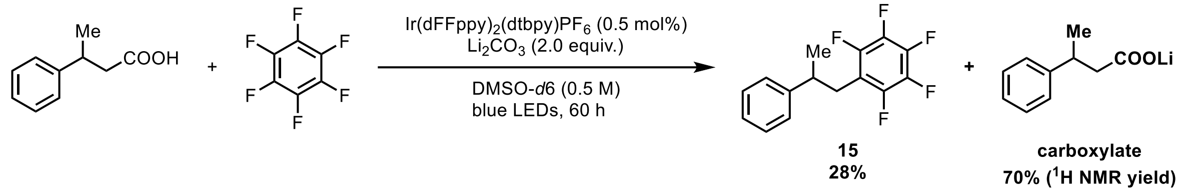

The reaction of 3-phenylbutanoic acid and perfluorobenzene was conducted in DMSO-*d*6. After irradiation for 60 hours, a ^{1}H NMR was measured. From the NMR spectrum, no obvious byproduct was formed. The ratio of the corresponding carboxylate and product **15** is 1: 0.4. The low conversion of the corresponding carboxylate caused the low yield.

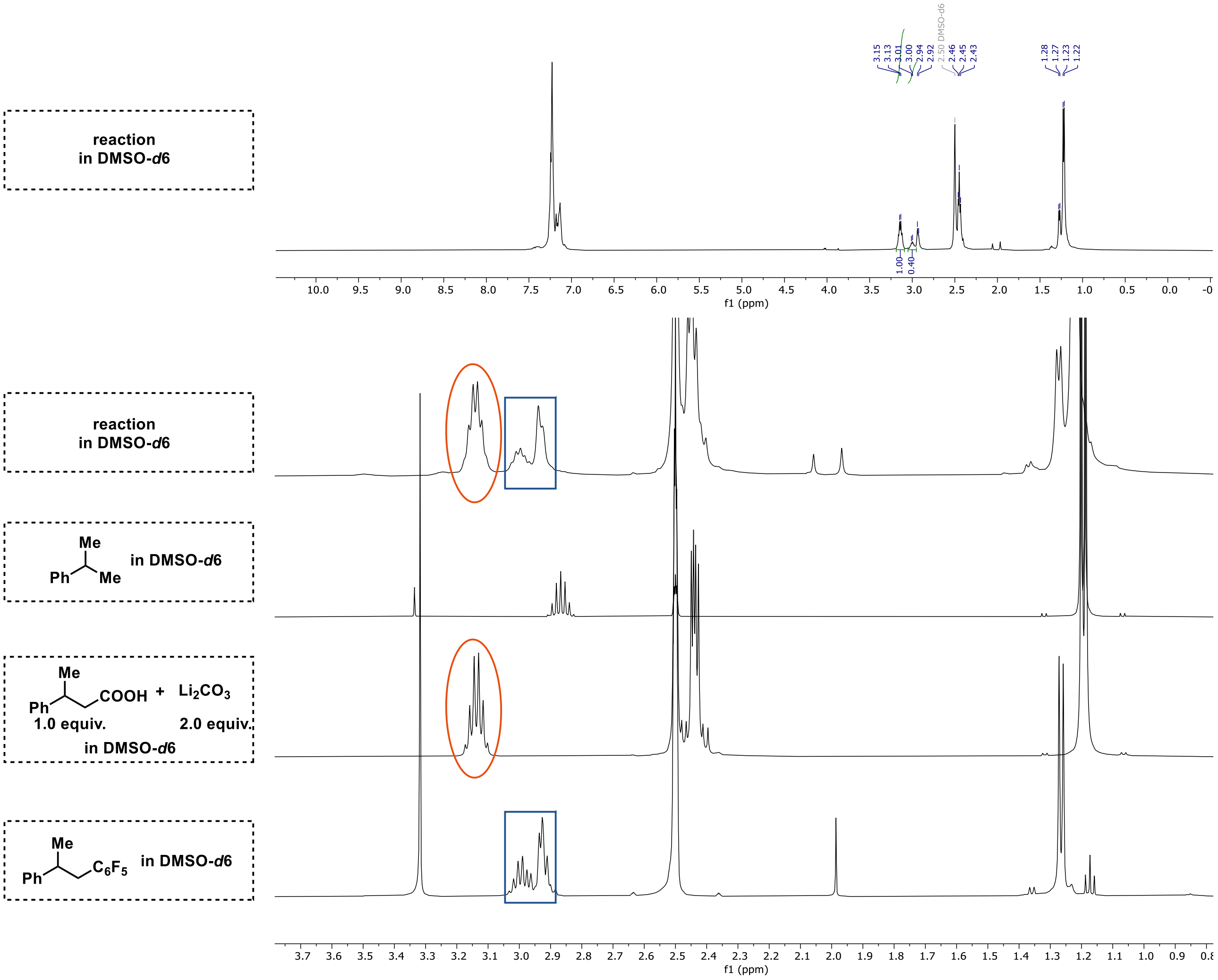

Scheme S8. Comparison of the ^{1}H NMR spectra of reaction, possible byproduct, starting material, and product.

SPECTROSCOPIC DATA

^{1}H NMR of (1R,2S,5R)-2-isopropyl-5-methylcyclohexylpentafluorobenzoate (S1)

$CDCl_3$, 23 ºC

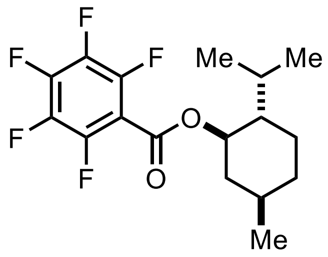

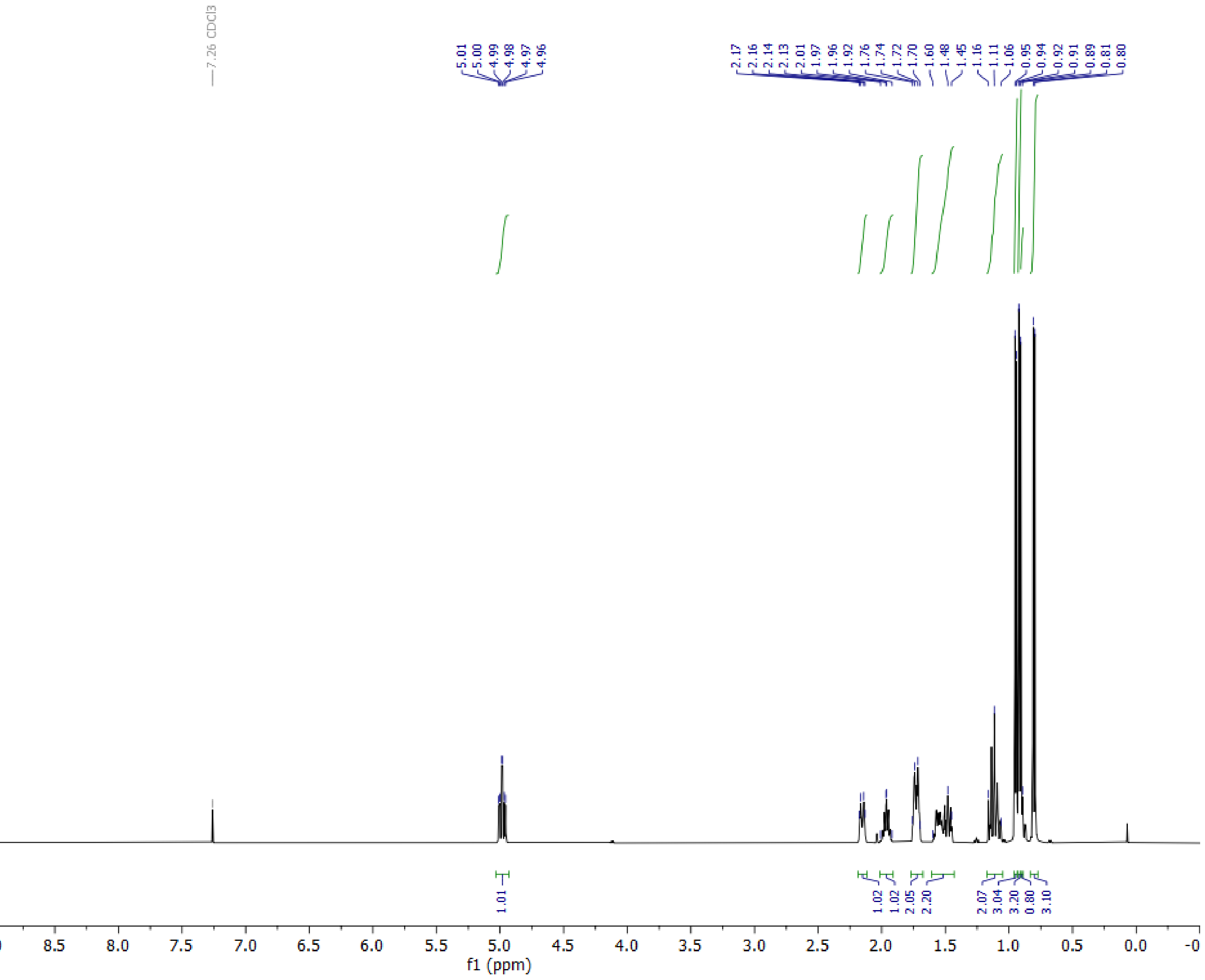

^{13}C NMR of (1R,2S,5R)-2-isopropyl-5-methylcyclohexylpentafluorobenzoate (S1)

CDCl3, 23 ºC

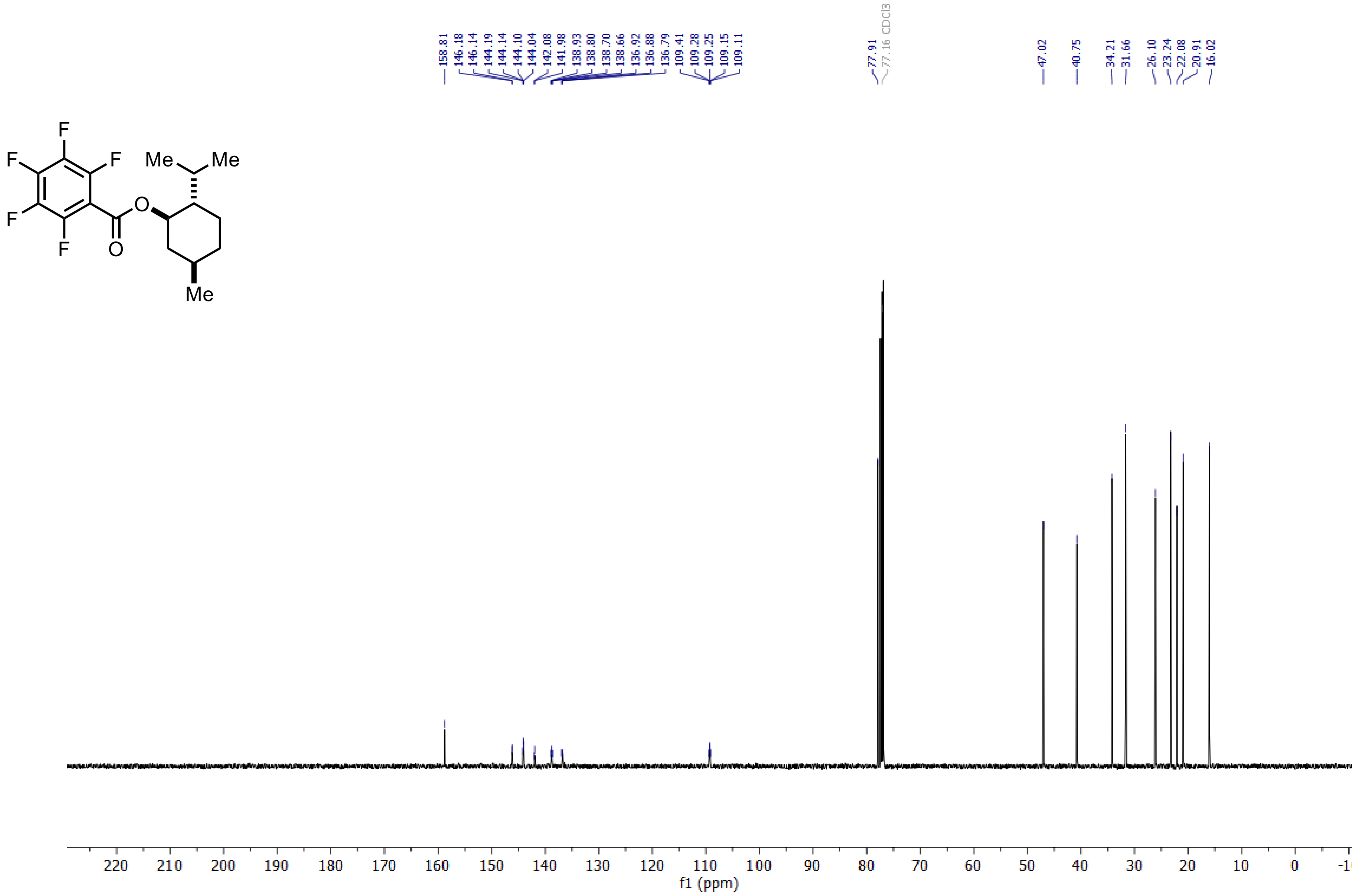

^{19}F NMR of (1R,2S,5R)-2-isopropyl-5-methylcyclohexylpentafluorobenzoate (S1)

$CDCl_3$, 23 ºC

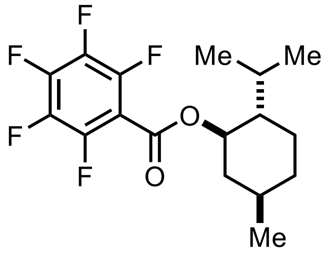

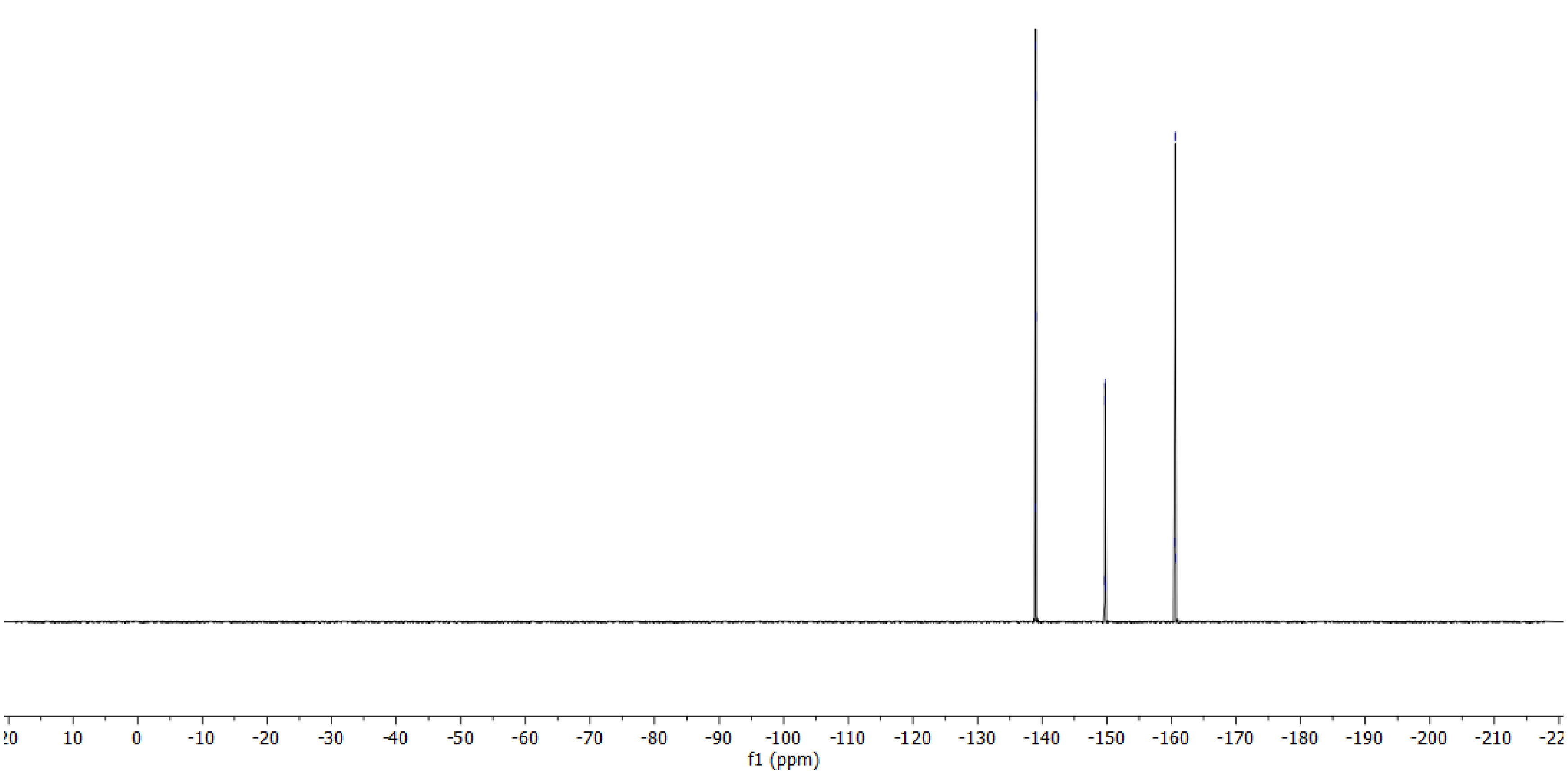

^1H NMR of 2-((1,3,5[10]-estratrien-17-one-3-yl)oxy)propanoic acid (S2)

$CDCl_3$, 23 ºC

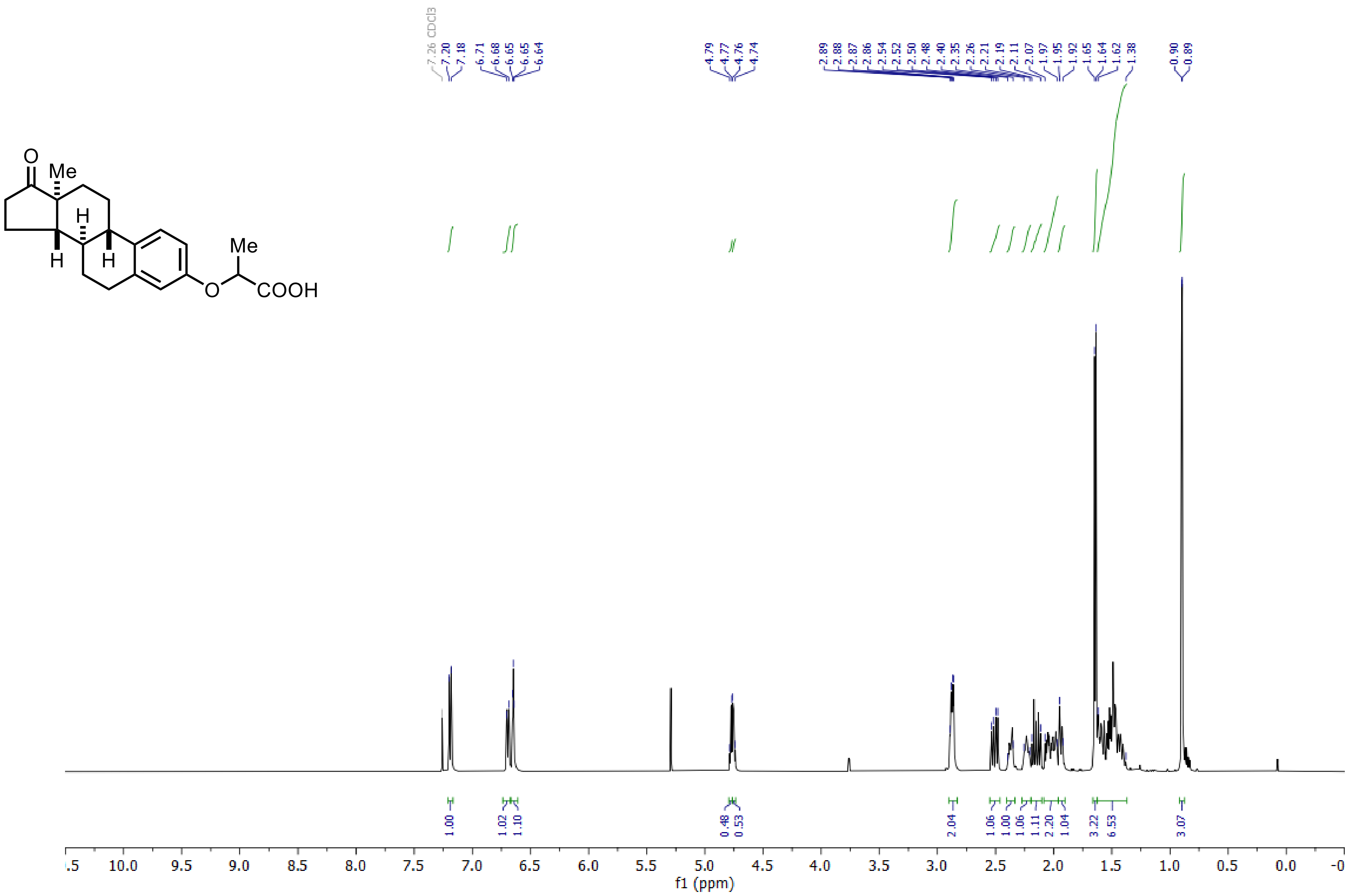

^{13}C NMR of 2-((1,3,5[10]-estratrien-17-one-3-yl)oxy)propanoic acid (S2)

$CDCl_3$, 23 ºC

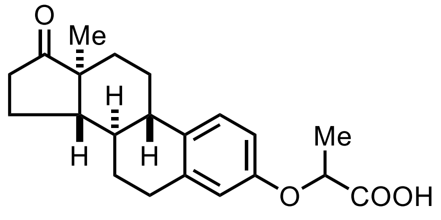

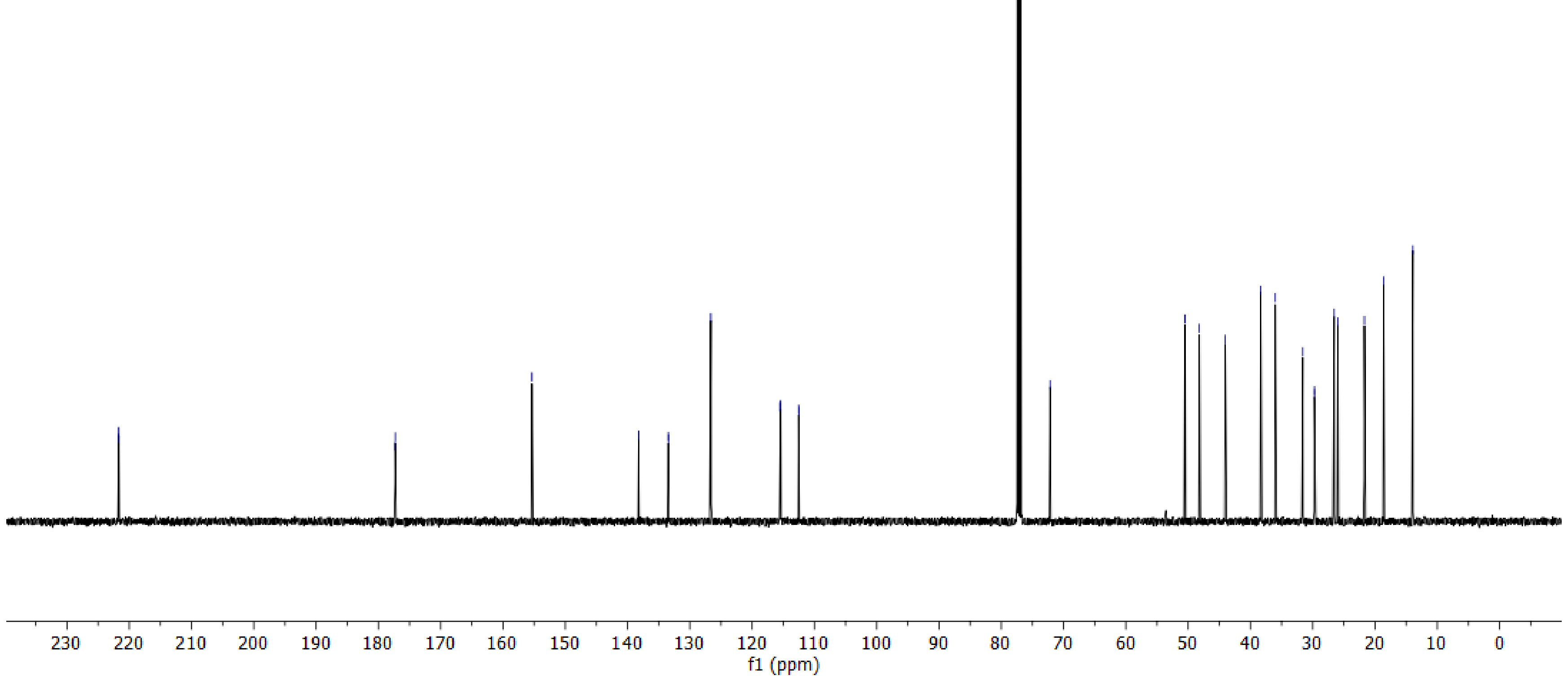

^{1}H NMR of Nateglinide derivative 1

CDCl$_3$, 23 ºC

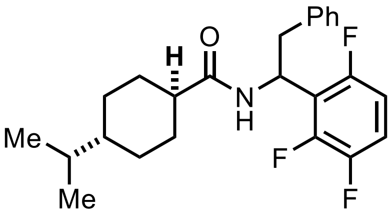

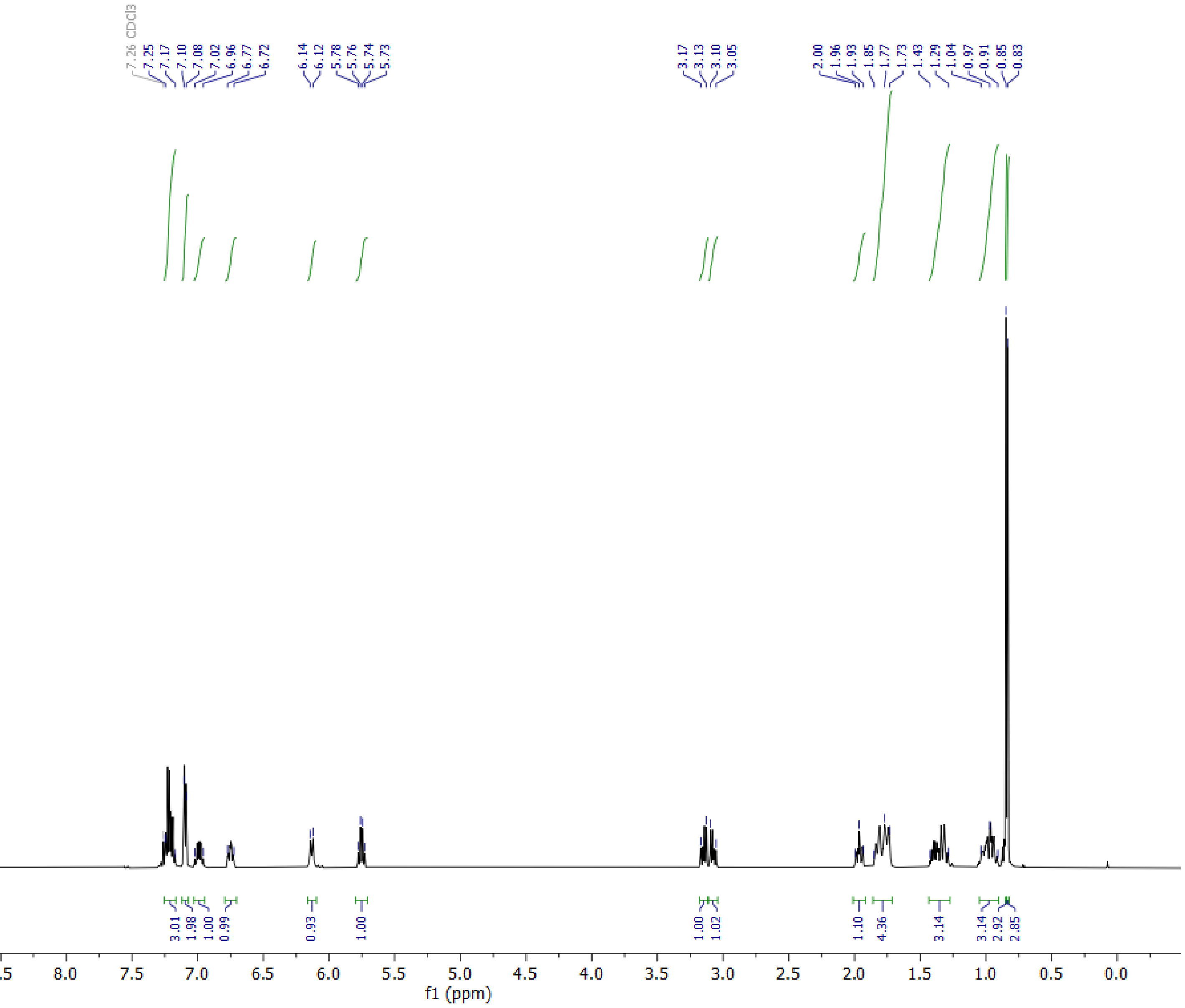

^{13}C NMR of Nateglinide derivative 1

$CDCl_3$, 23 ºC

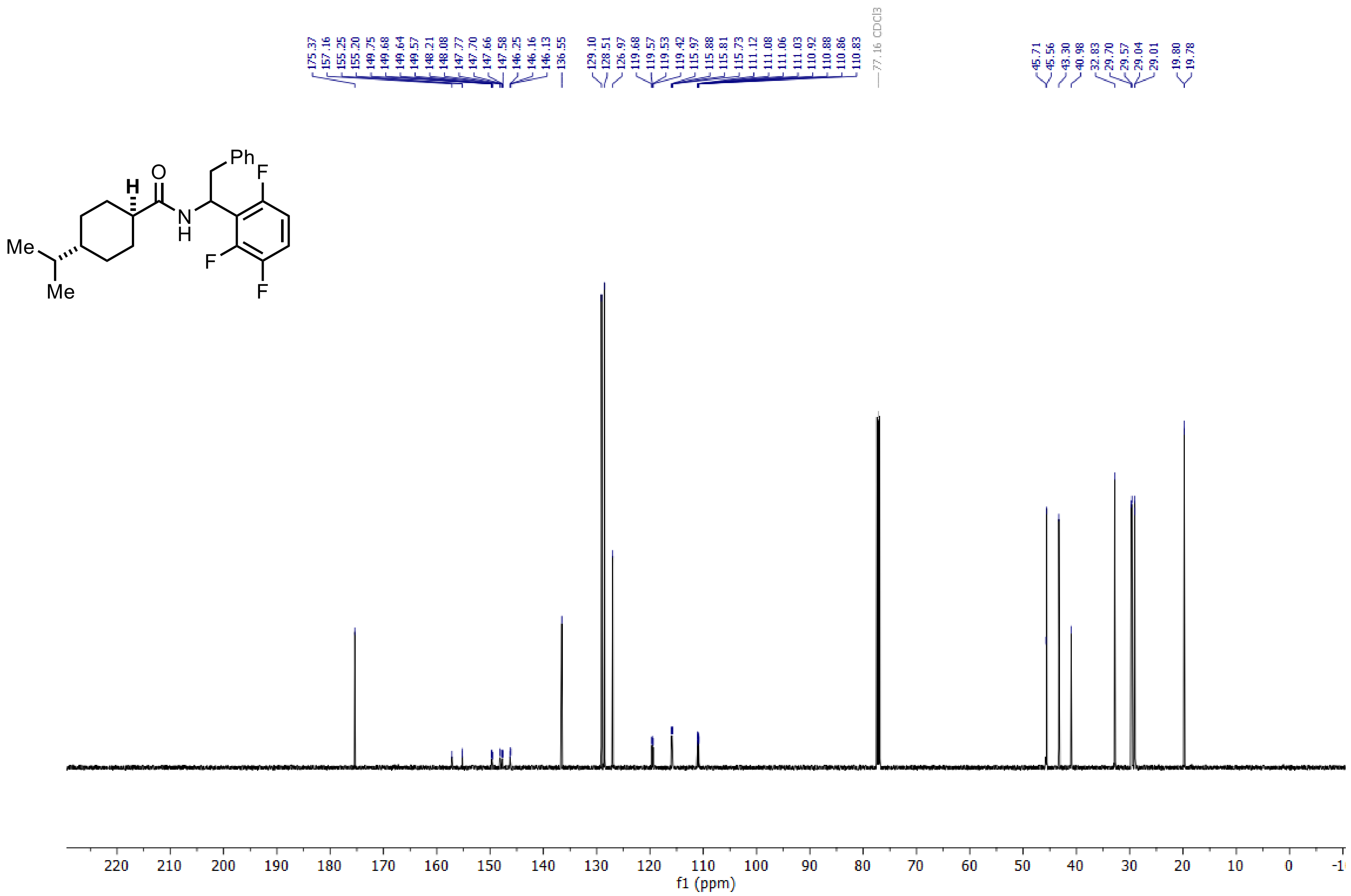

^{19}F NMR of Nateglinide derivative 1

$CDCl_3$, 23 ºC

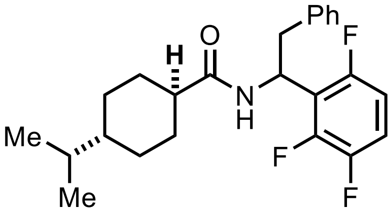

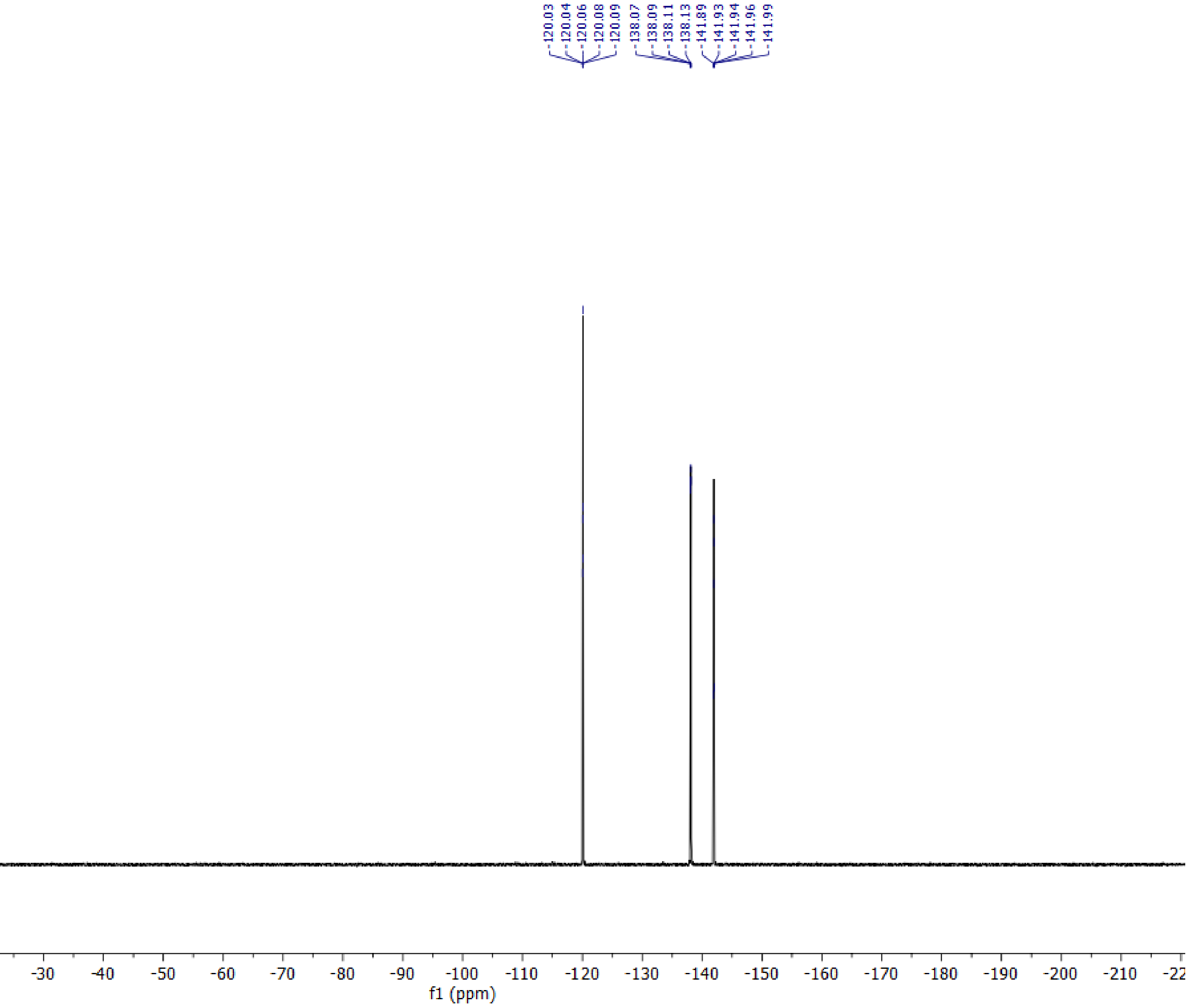

^{1}H NMR of Pentafluoro-(1-phenylpropan-2-yl)benzene (2)

$CDCl_3$, 23 ºC

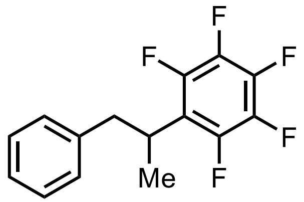

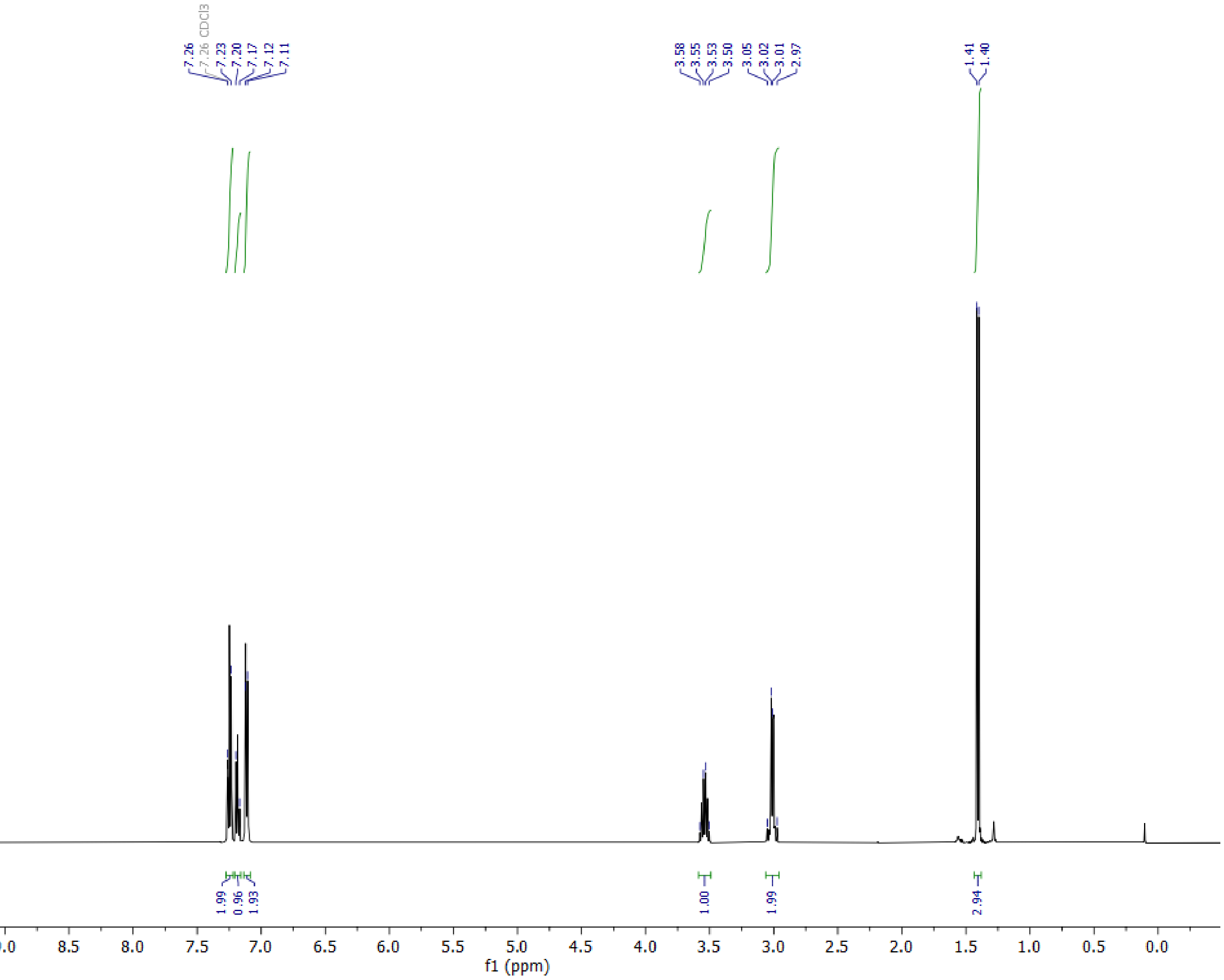

^{13}C NMR of Pentafluoro-(1-phenylpropan-2-yl)benzene (2)

$CDCl_3$, 23 ºC

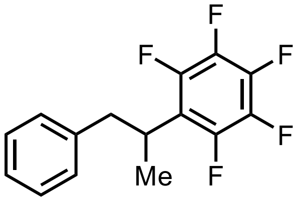

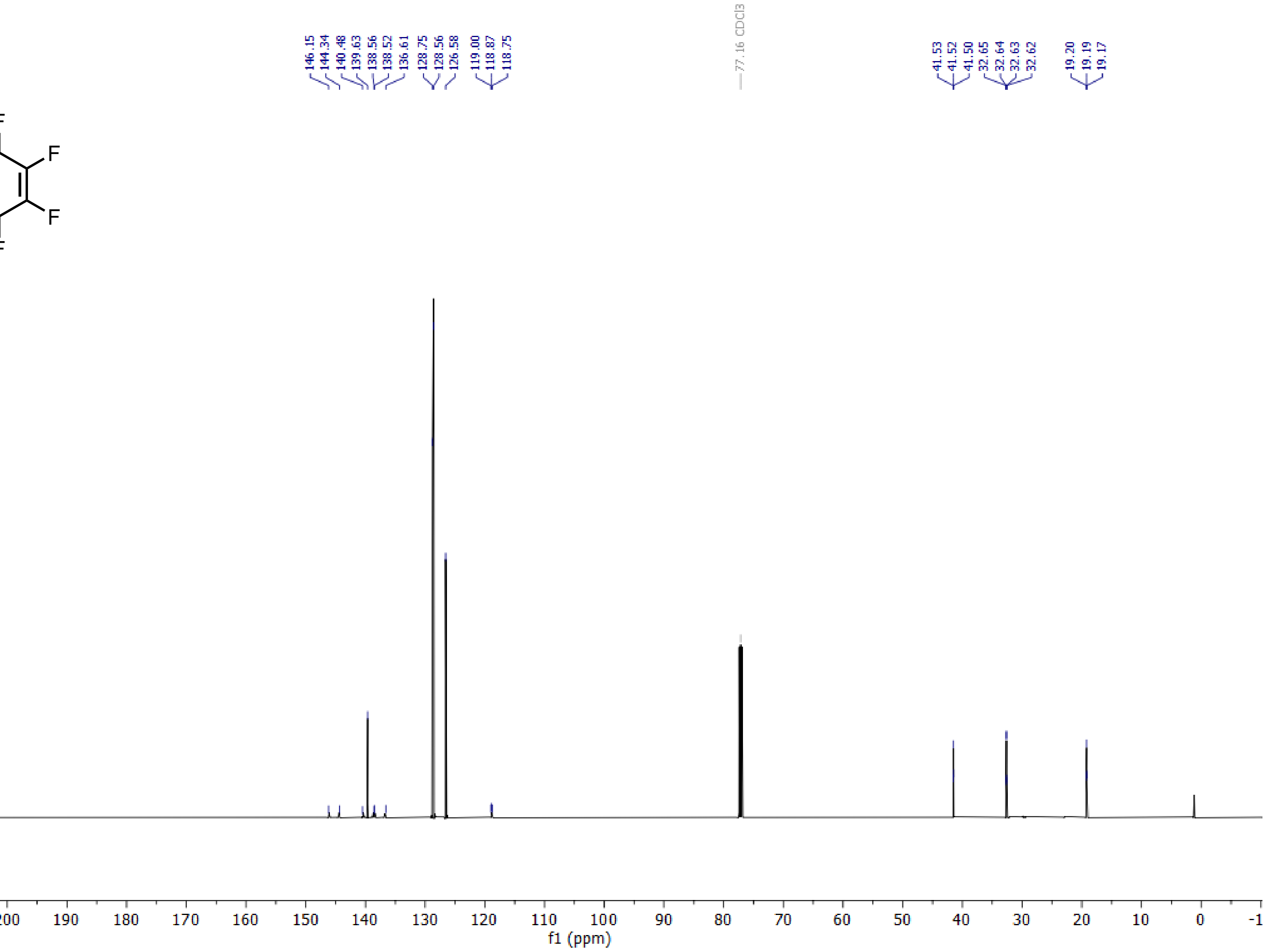

^{19}F NMR of Pentafluoro-(1-phenylpropan-2-yl)benzene (2)

$CDCl_3$, 23 ºC

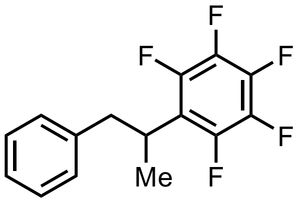

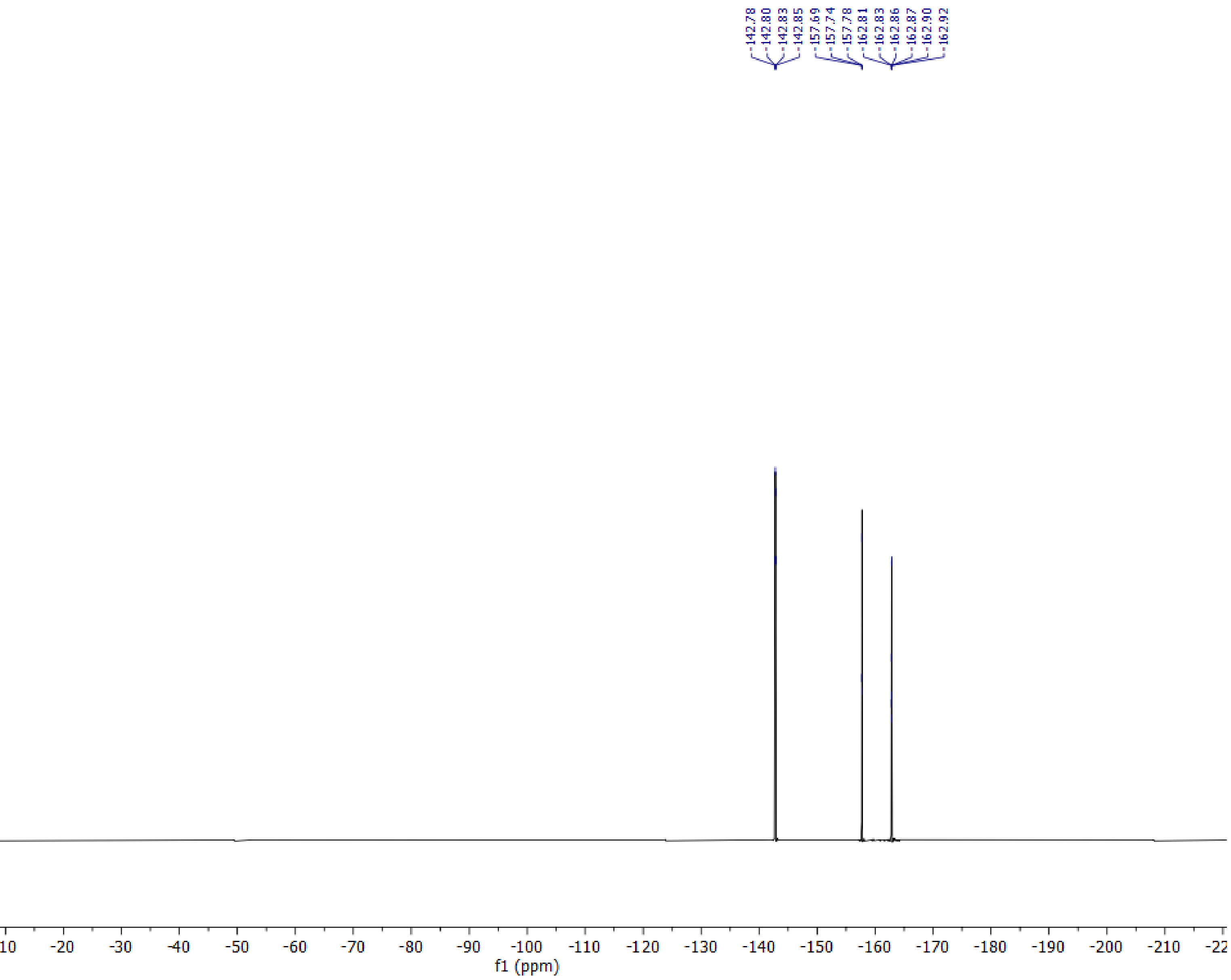

^{1}H NMR of Cyclopentylpentafluorobenzene (3)

$CDCl_3$, 23 ºC

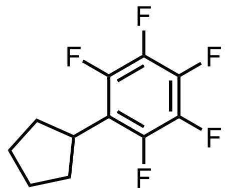

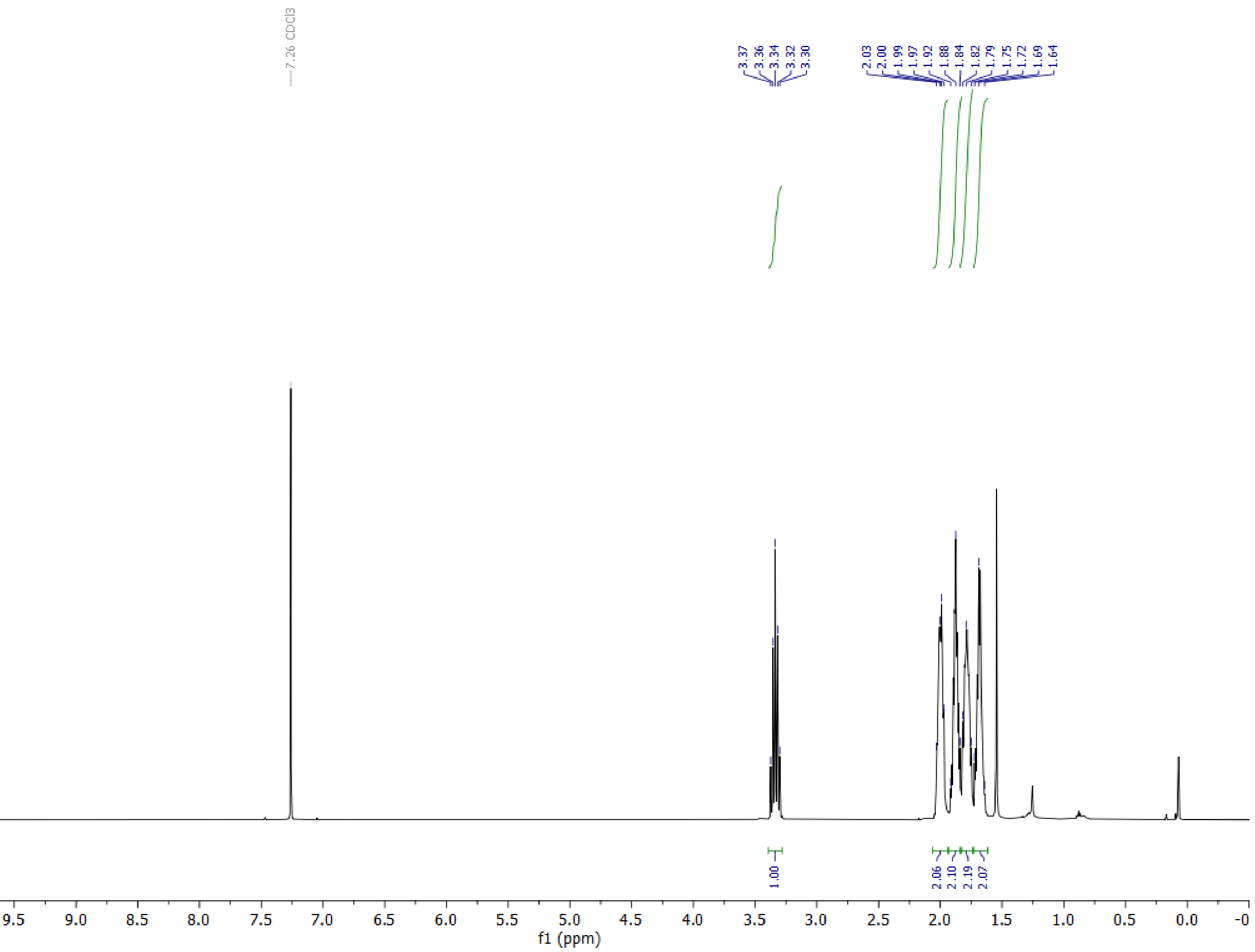

^{13}C NMR of Cyclopentylpentafluorobenzene (3)

$CDCl_3$, 23 ºC

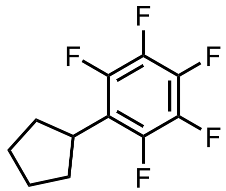

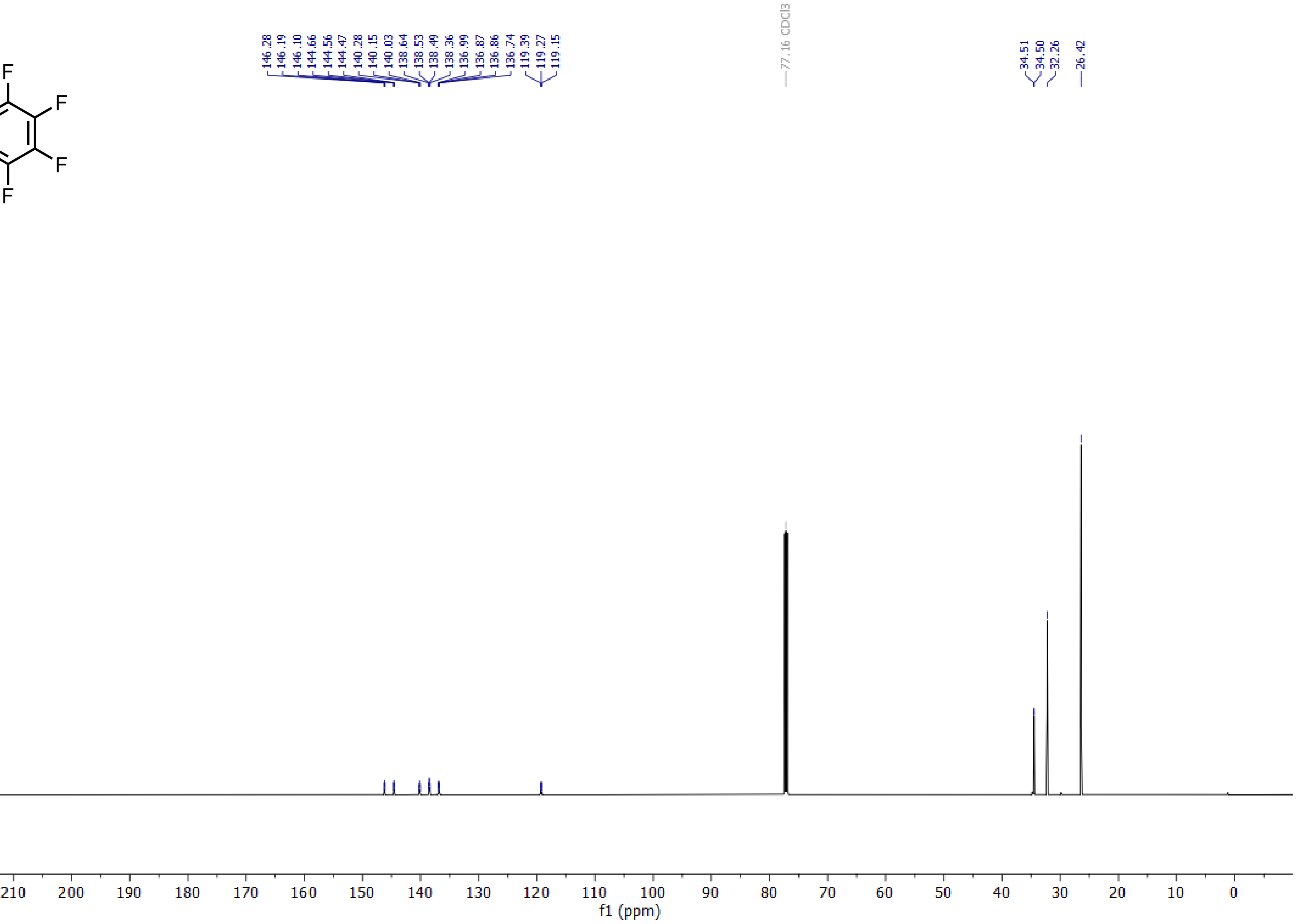

^{19}F NMR of Cyclopentylpentafluorobenzene (3)

$CDCl_3$, 23 ºC

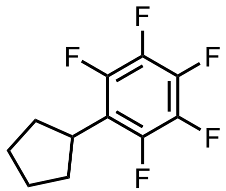

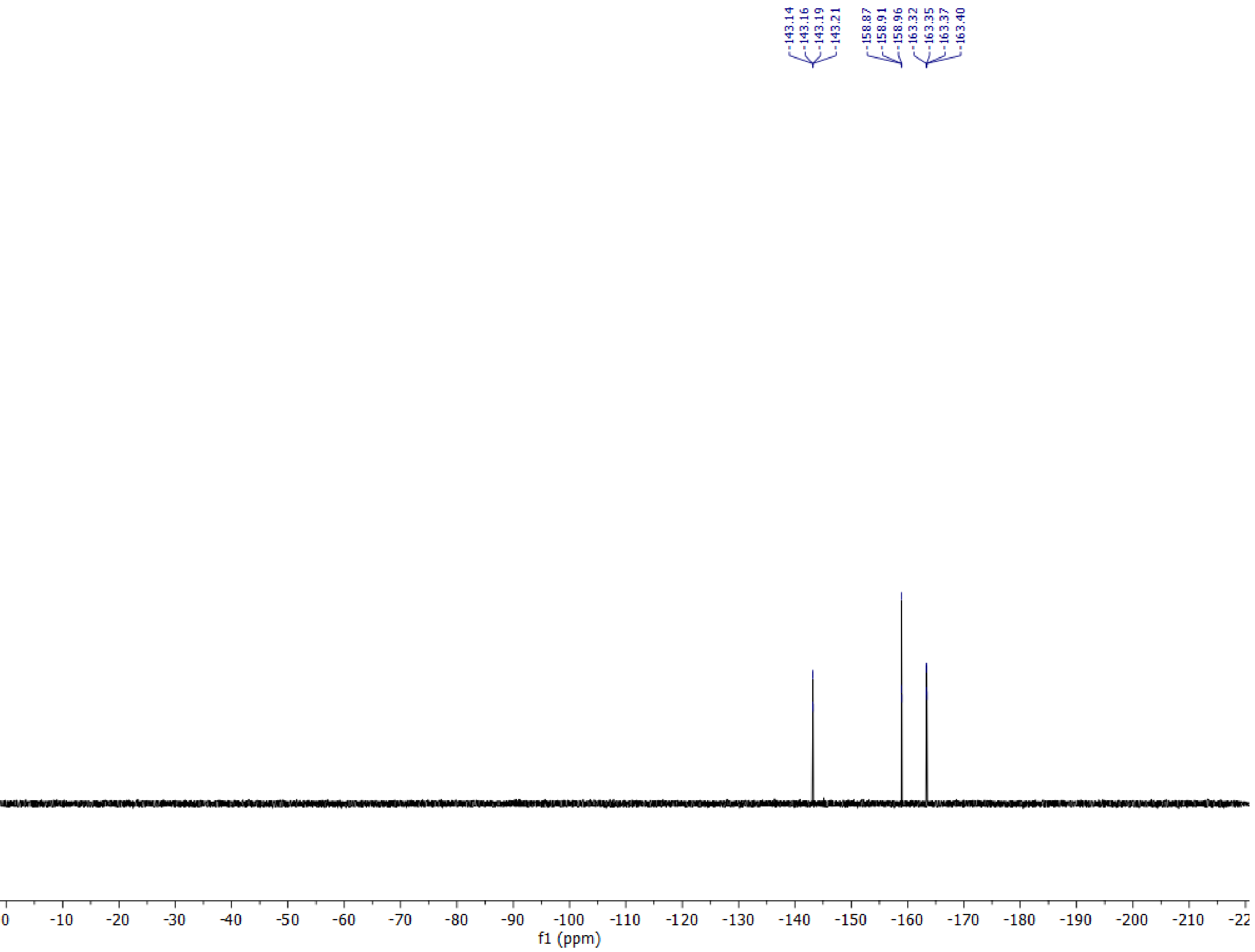

^{1}H NMR of *N*-Benzoyl-4-perfluorophenylpiperidine (4)

DMSO-d_6, 100 ºC

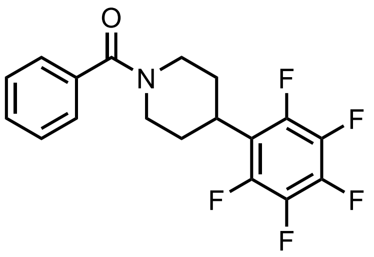

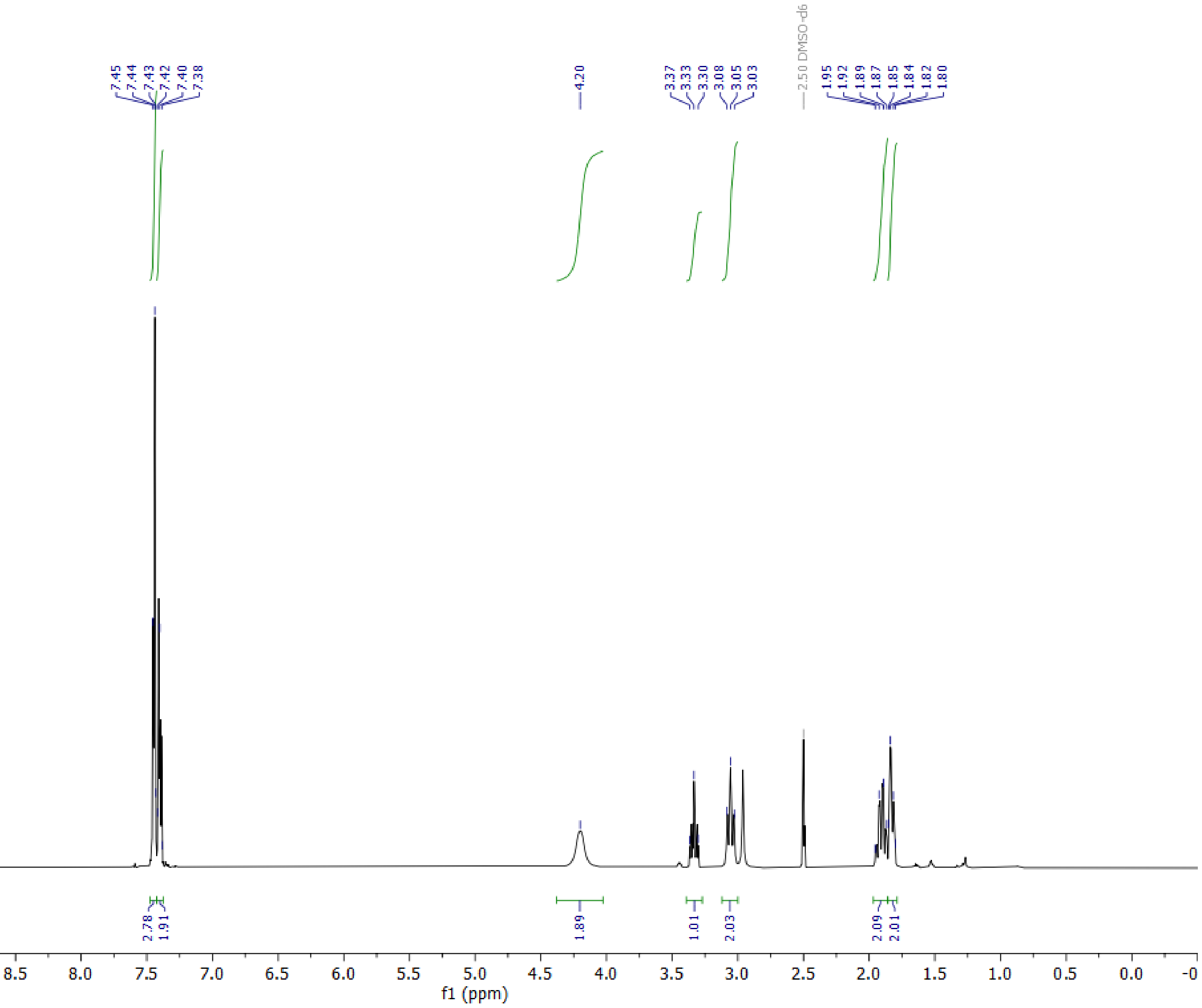

^{13}C NMR of *N*-Benzoyl-4-perfluorophenylpiperidine (4)

DMSO-d_6, 100 ºC

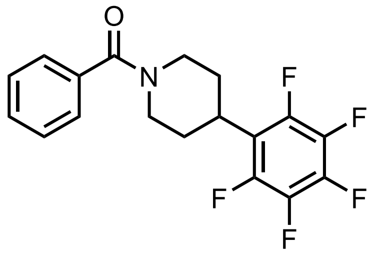

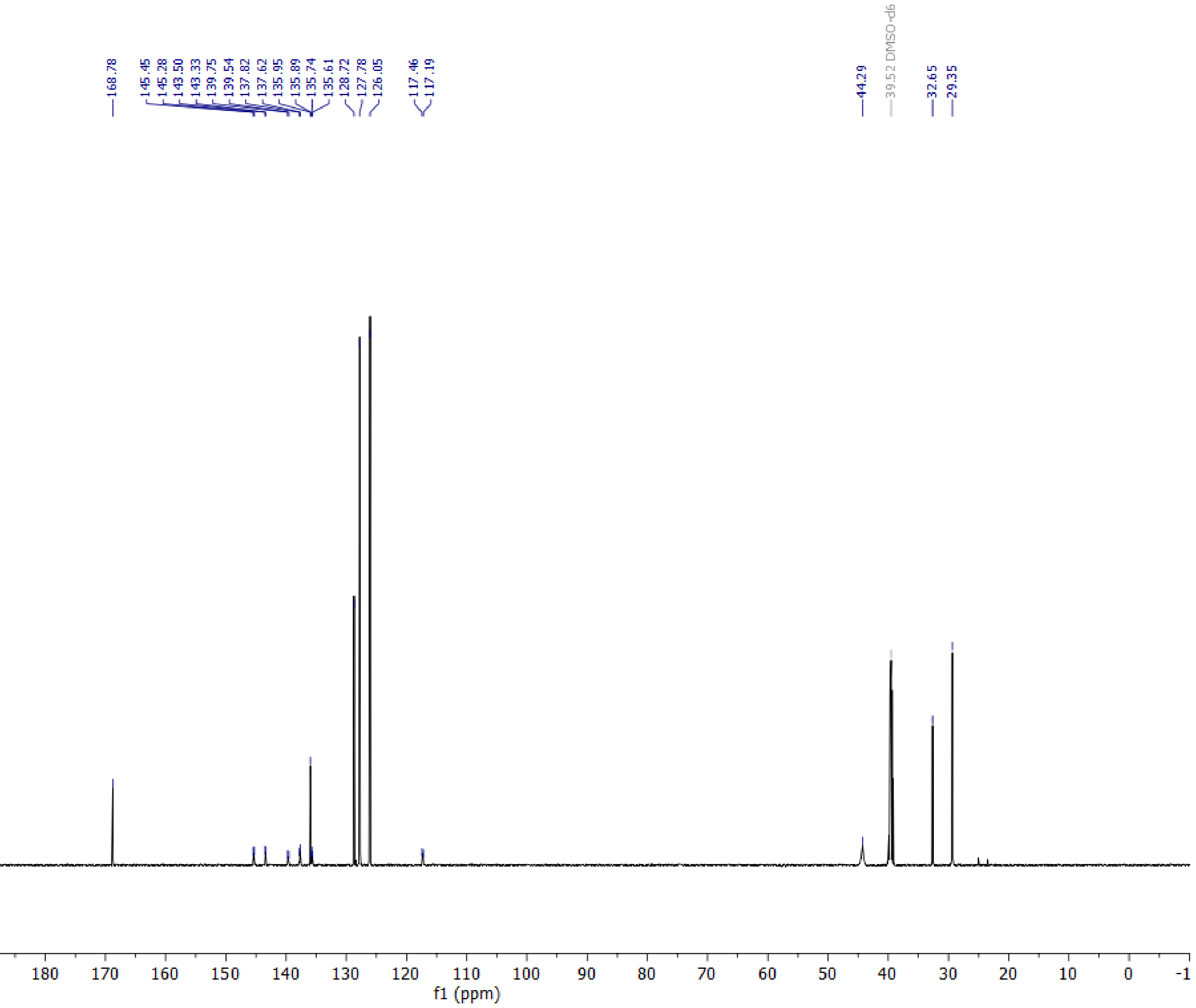

^{19}F NMR of *N*-Benzoyl-4-perfluorophenylpiperidine (4)

DMSO-d_6, 100 ºC

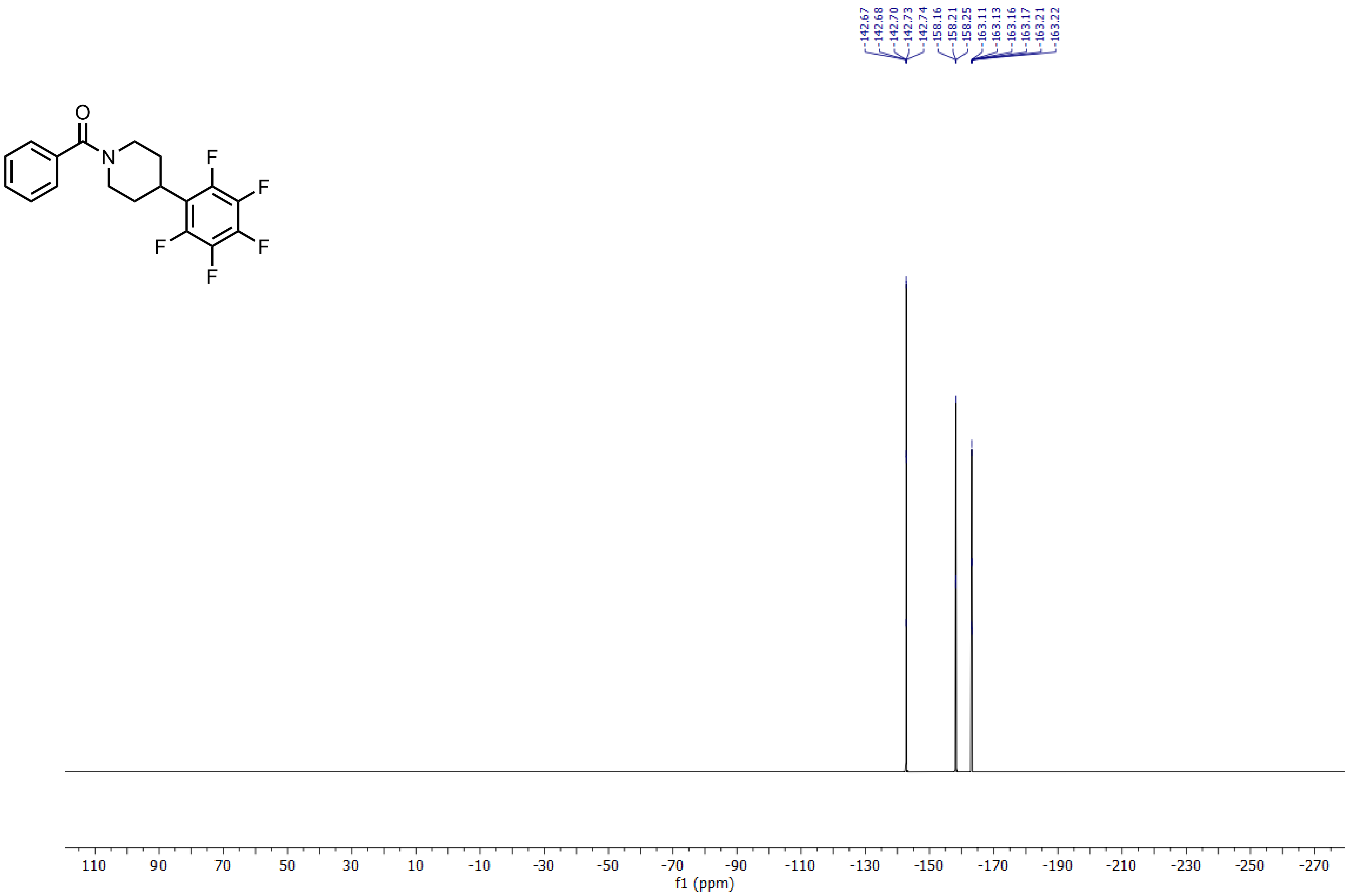

^{1}H NMR of Indomethacin derivative 5

CDCl$_3$, 23 ºC

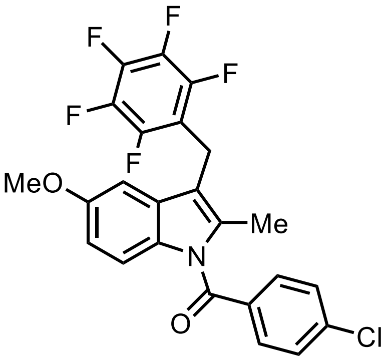

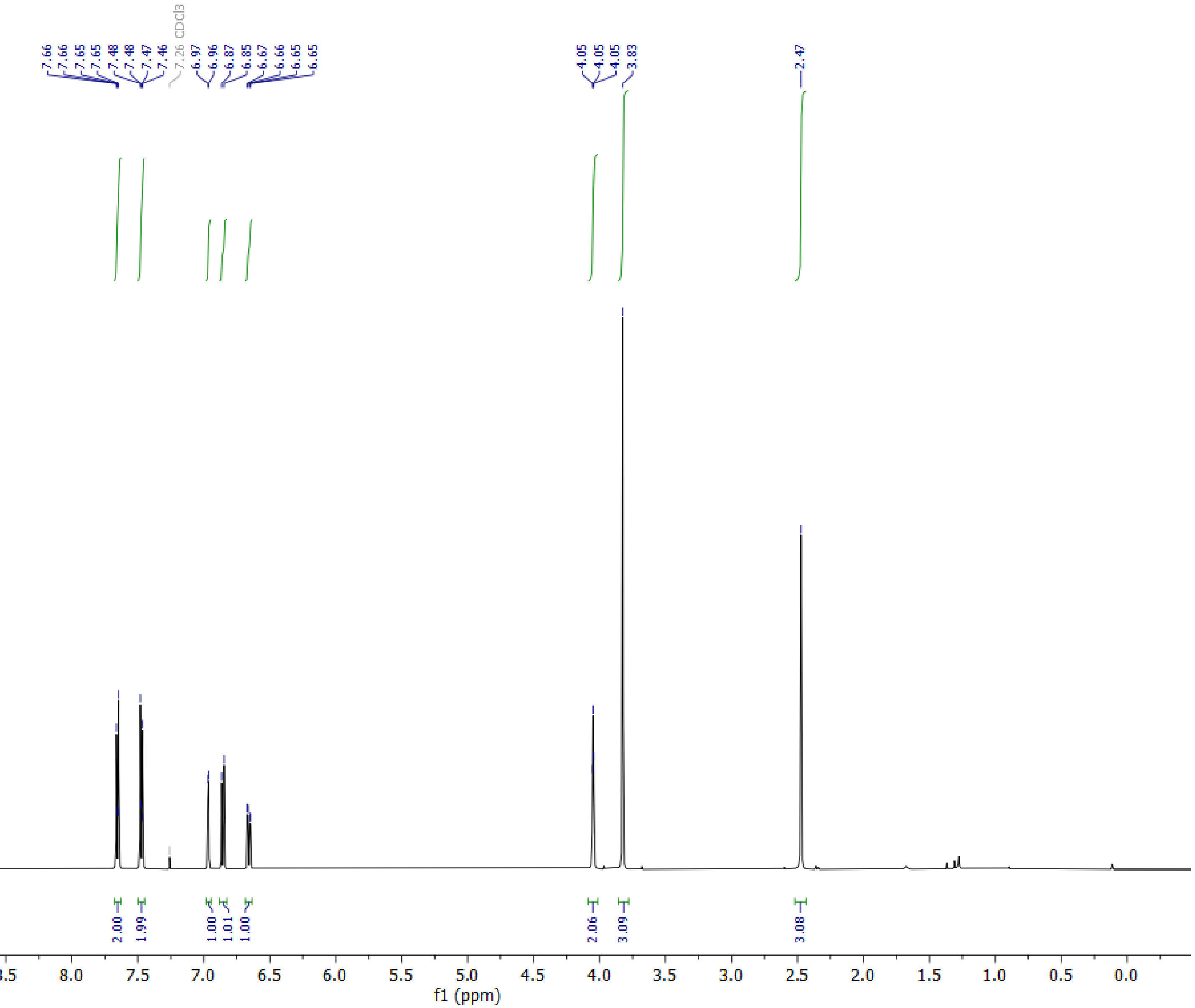

^{13}C NMR of Indomethacin derivative 5

$CDCl_3$, 23 ºC

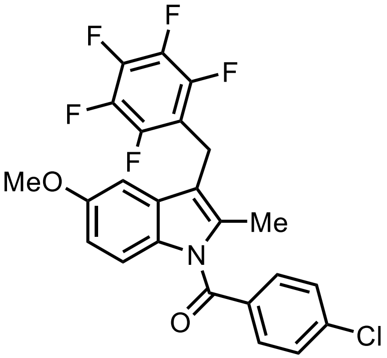

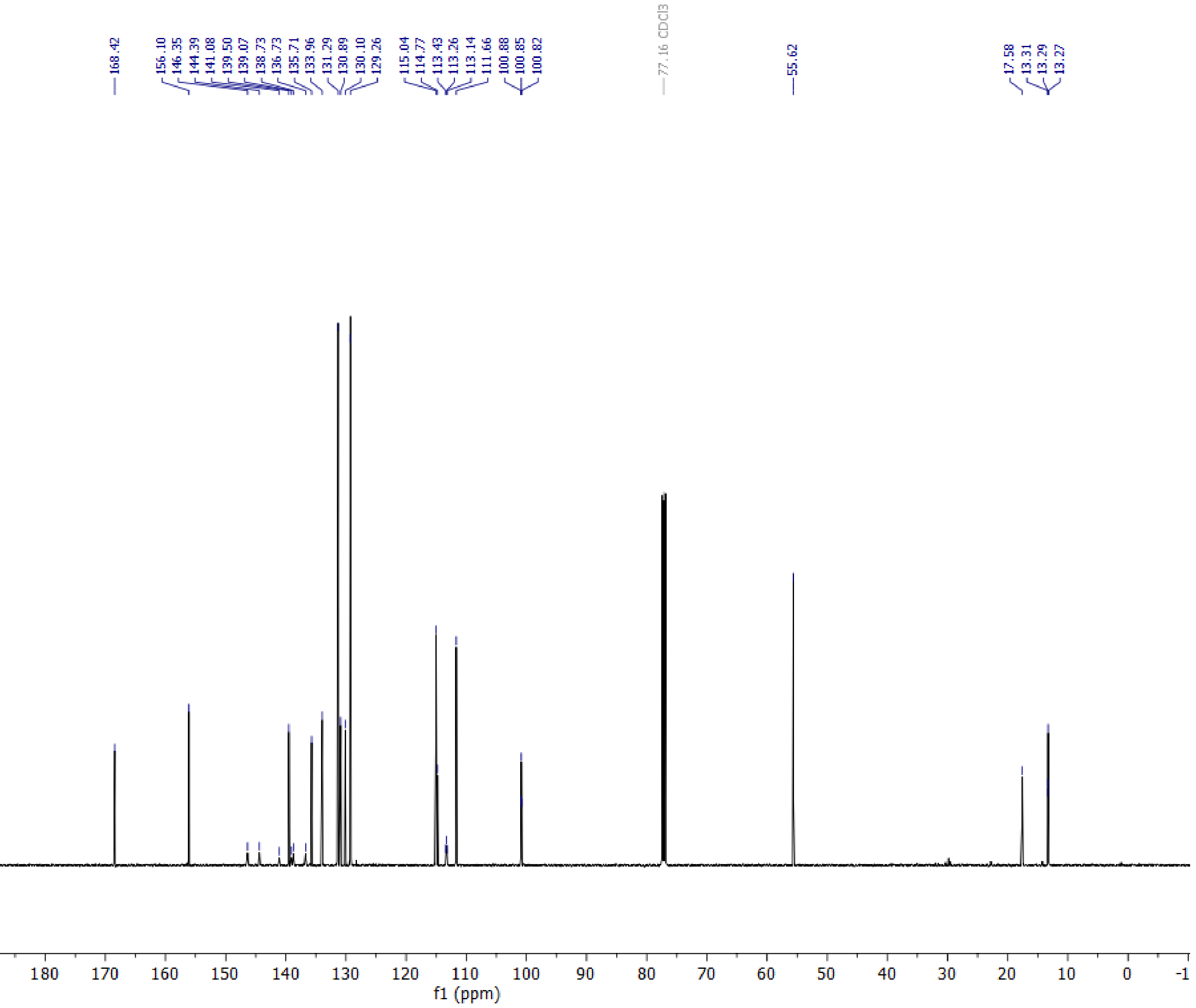

^{19}F NMR of Indomethacin derivative 5

$CDCl_3$, 23 ºC

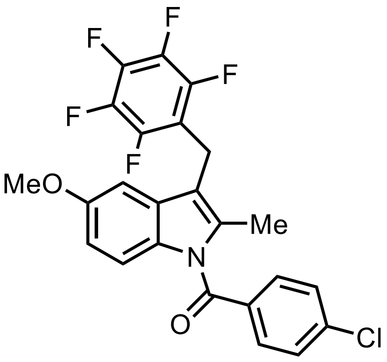

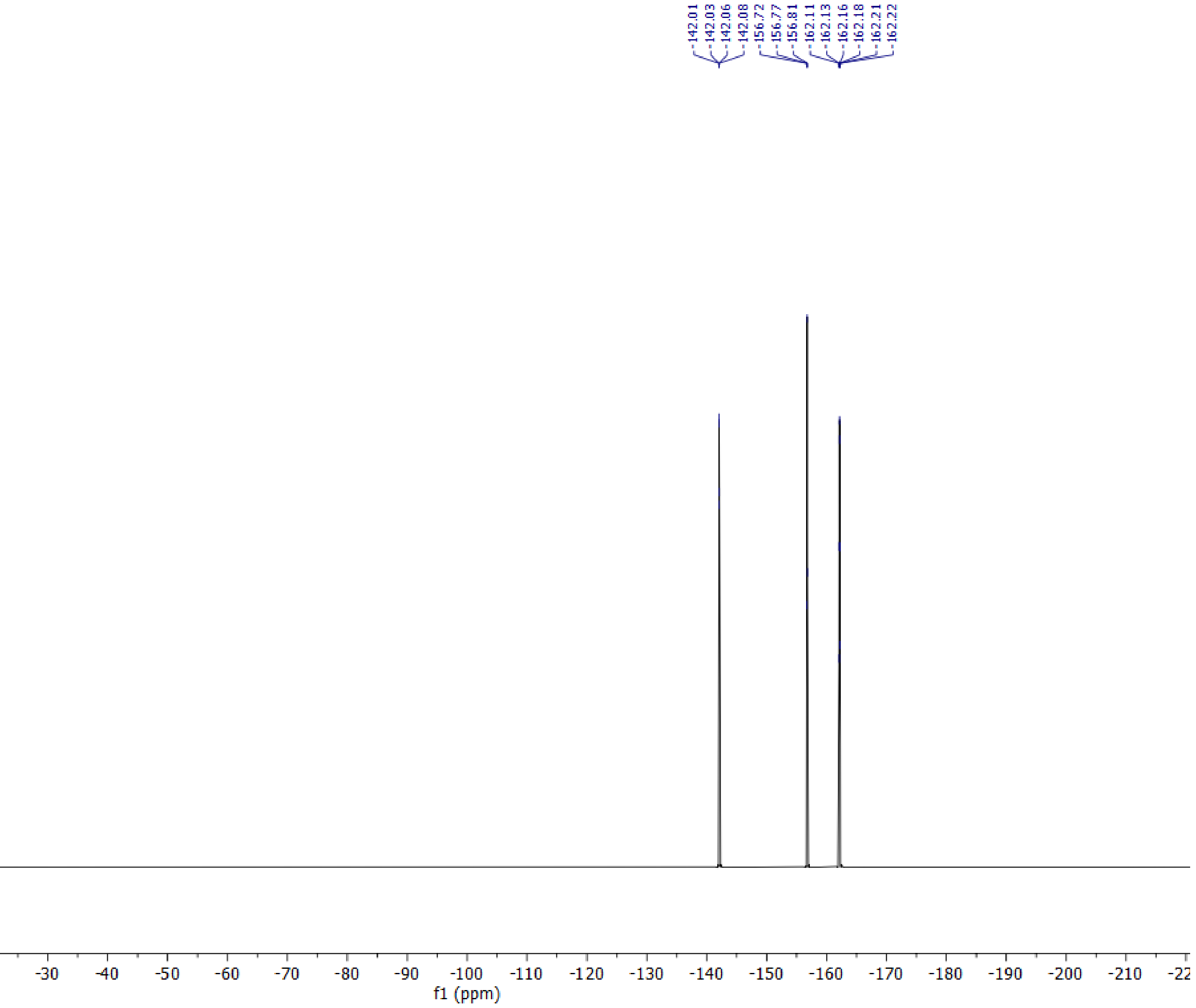

^{1}H NMR of 4-(Perfluorophenyl)tetrahydropyran (6)

$CDCl_3$, 23 ºC

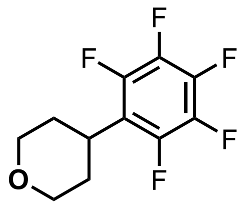

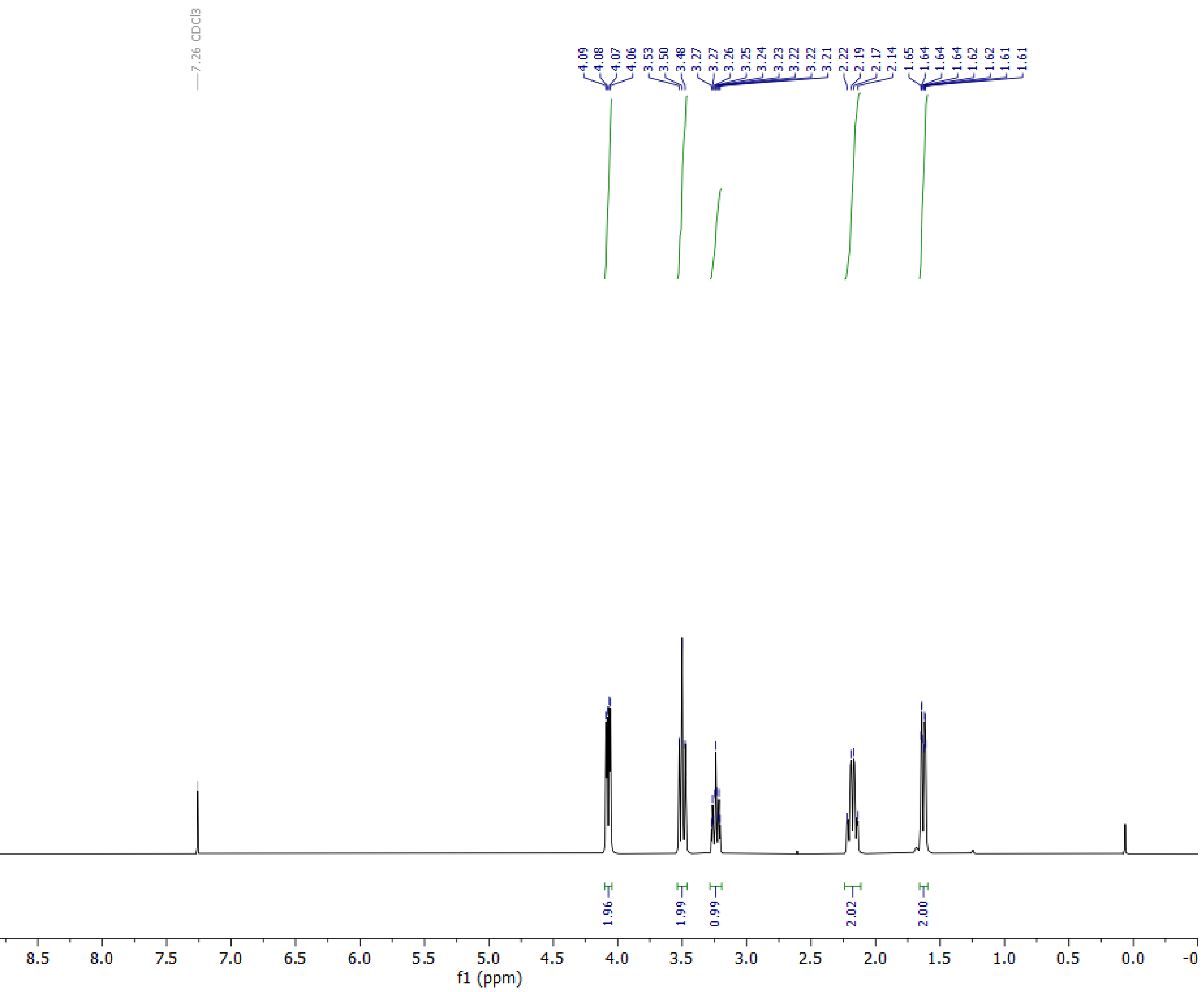

^{13}C NMR of 4-(Perfluorophenyl)tetrahydropyran (6)

$CDCl_3$, 23 ºC

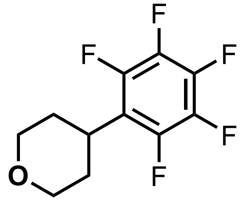

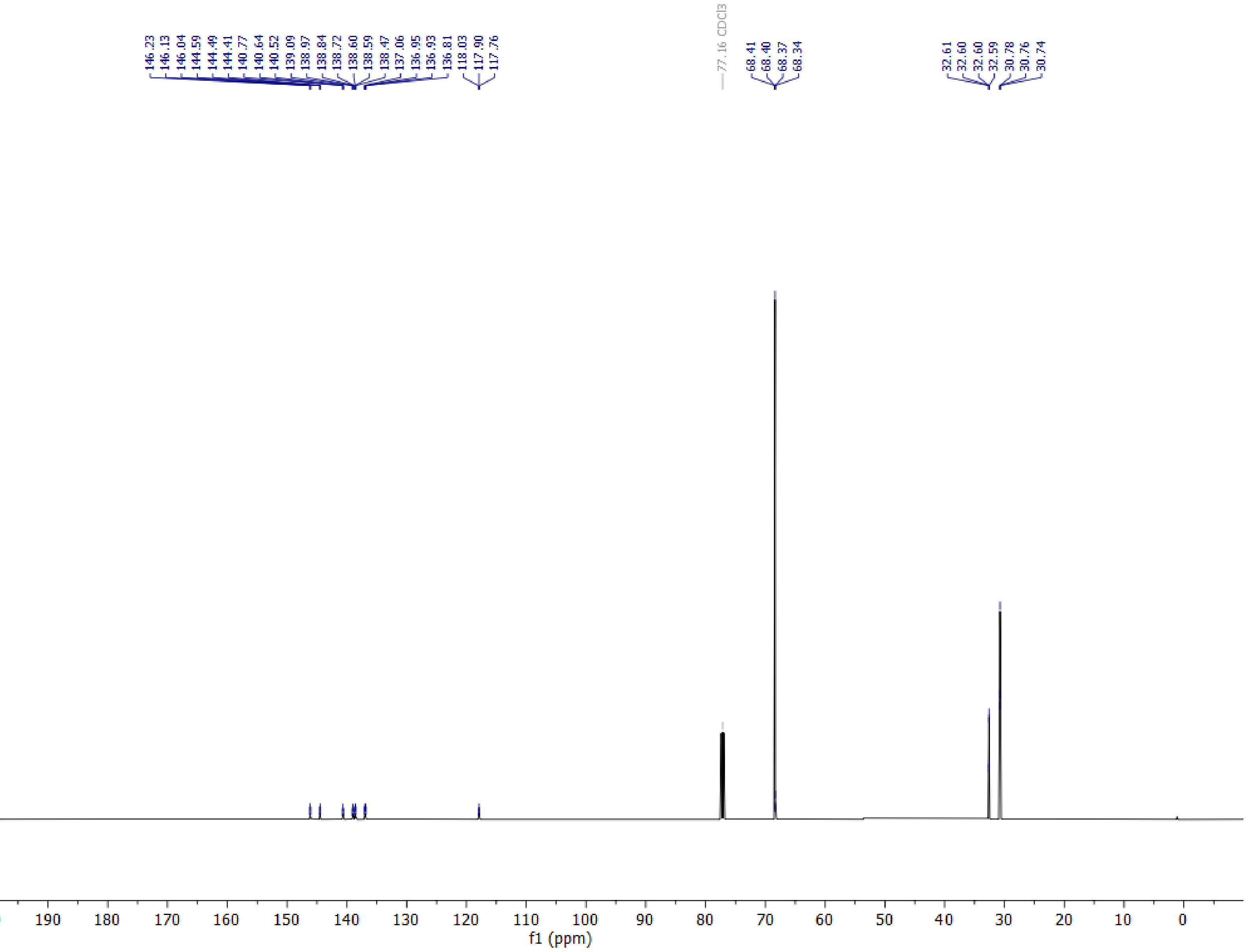

^{19}F NMR of 4-(Perfluorophenyl)tetrahydropyran (6)

$CDCl_3$, 23 ºC

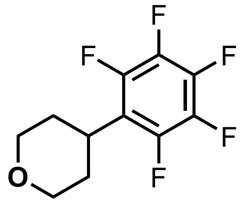

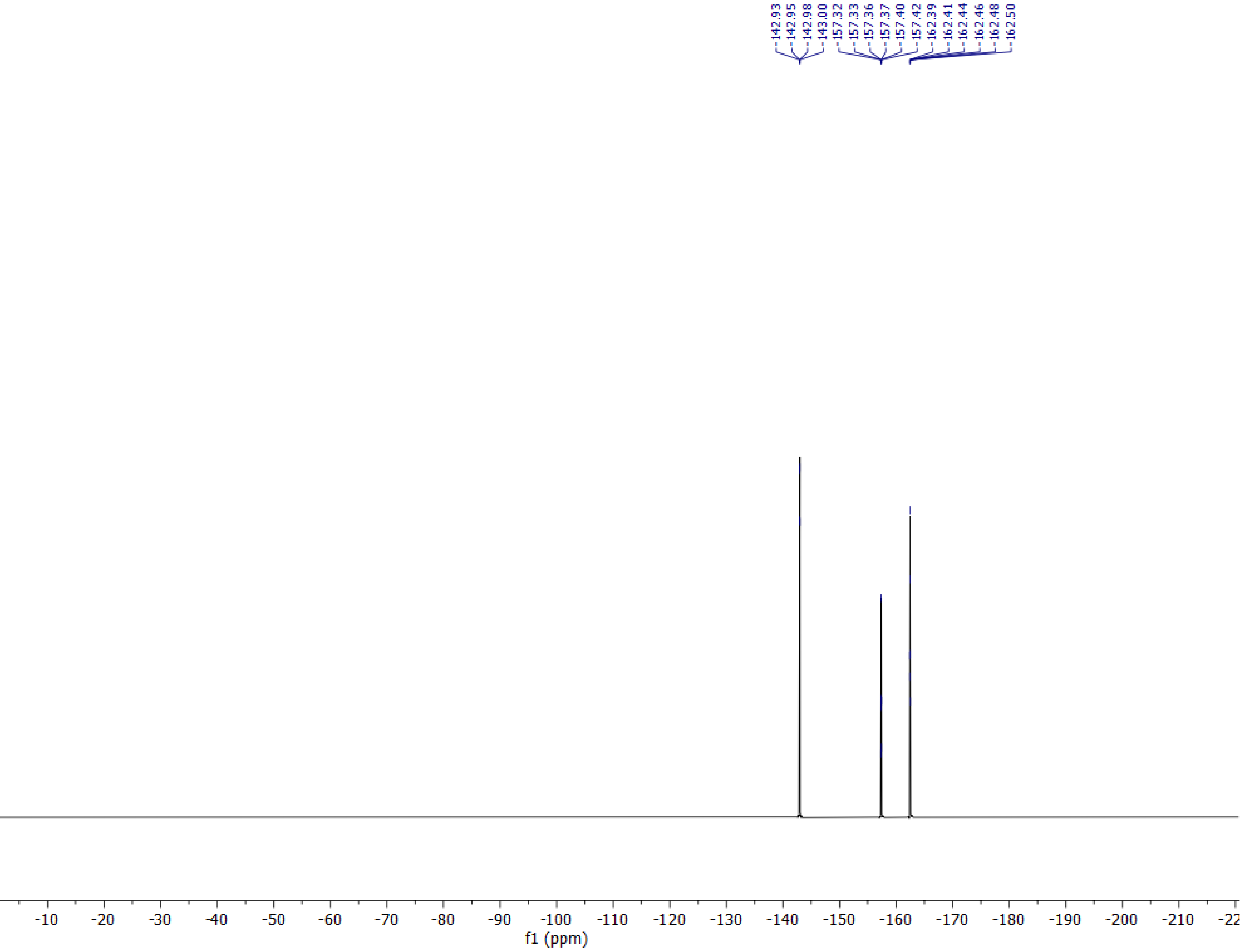

^{1}H NMR of *N*-Boc-2-perfluoropehnylpyrrolidine (7)

DMSO-d_6, 80 ºC

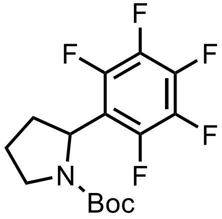

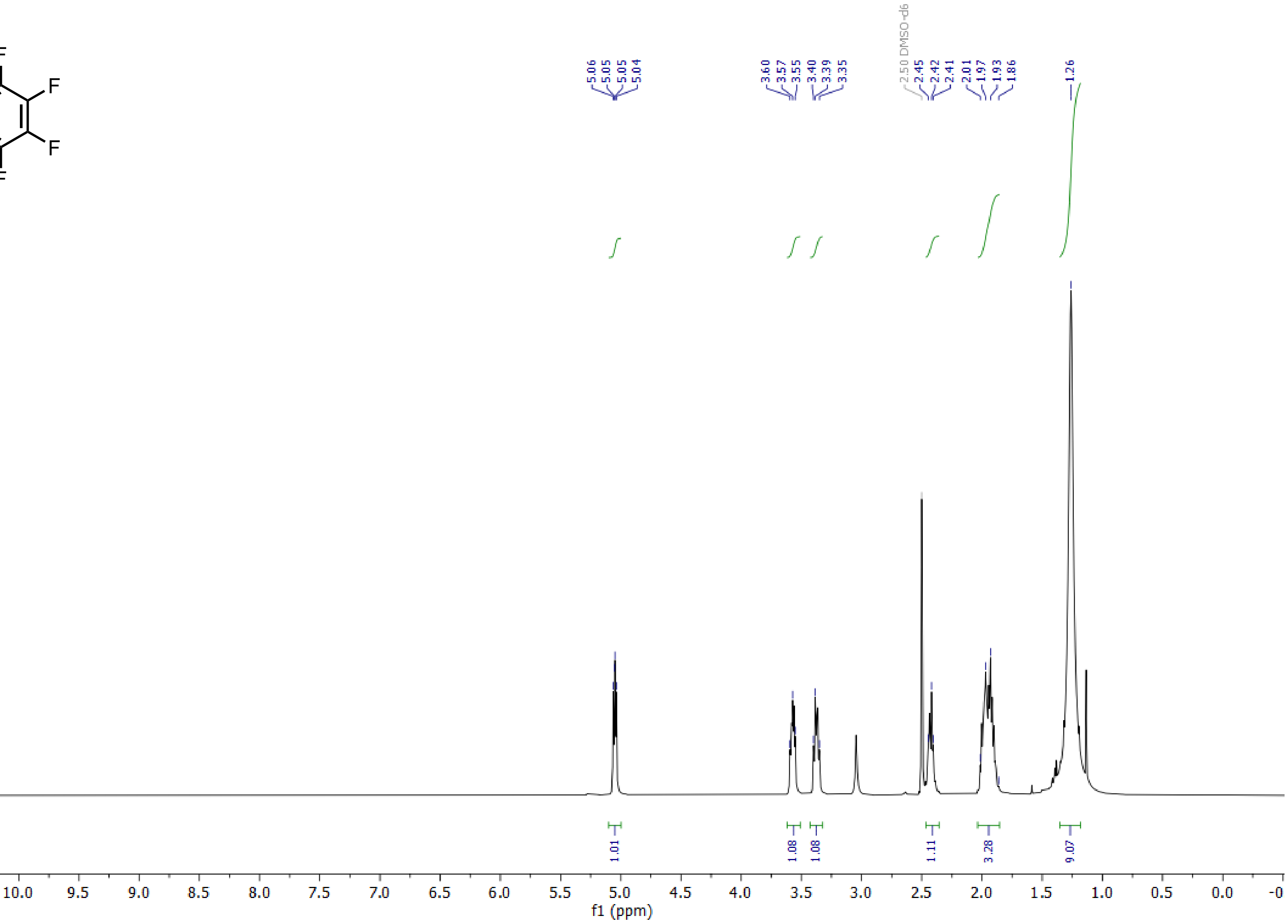

^{13}C NMR of *N*-Boc-2-perfluoropehnylpyrrolidine (7)

DMSO-d_6, 80 ºC

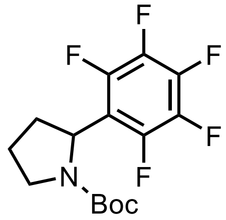

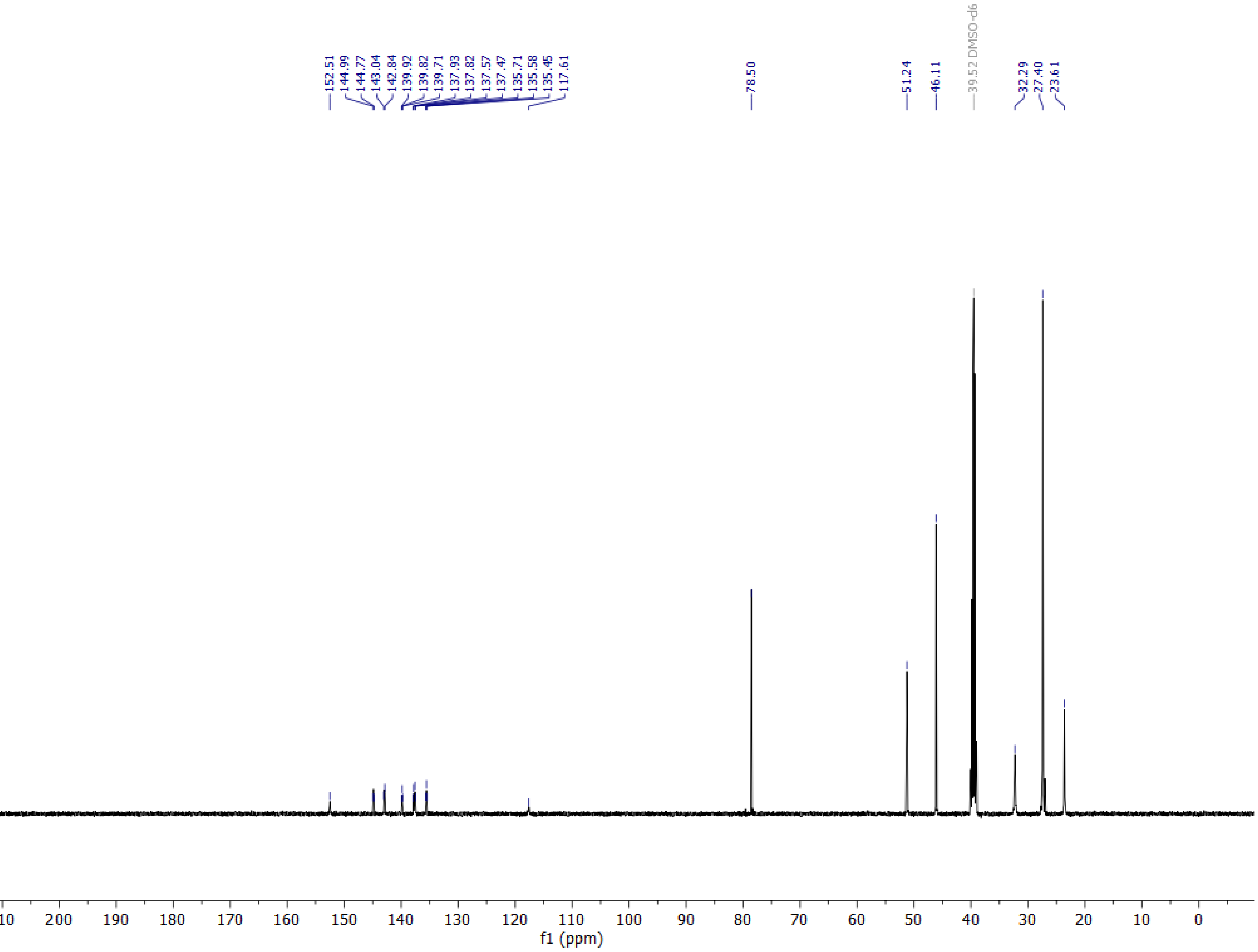

^{19}F NMR of *N*-Boc-2-perfluoropehnylpyrrolidine (7)

DMSO-d_6, 80 ºC

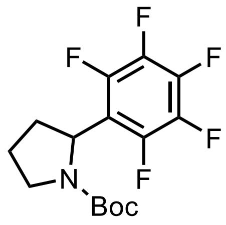

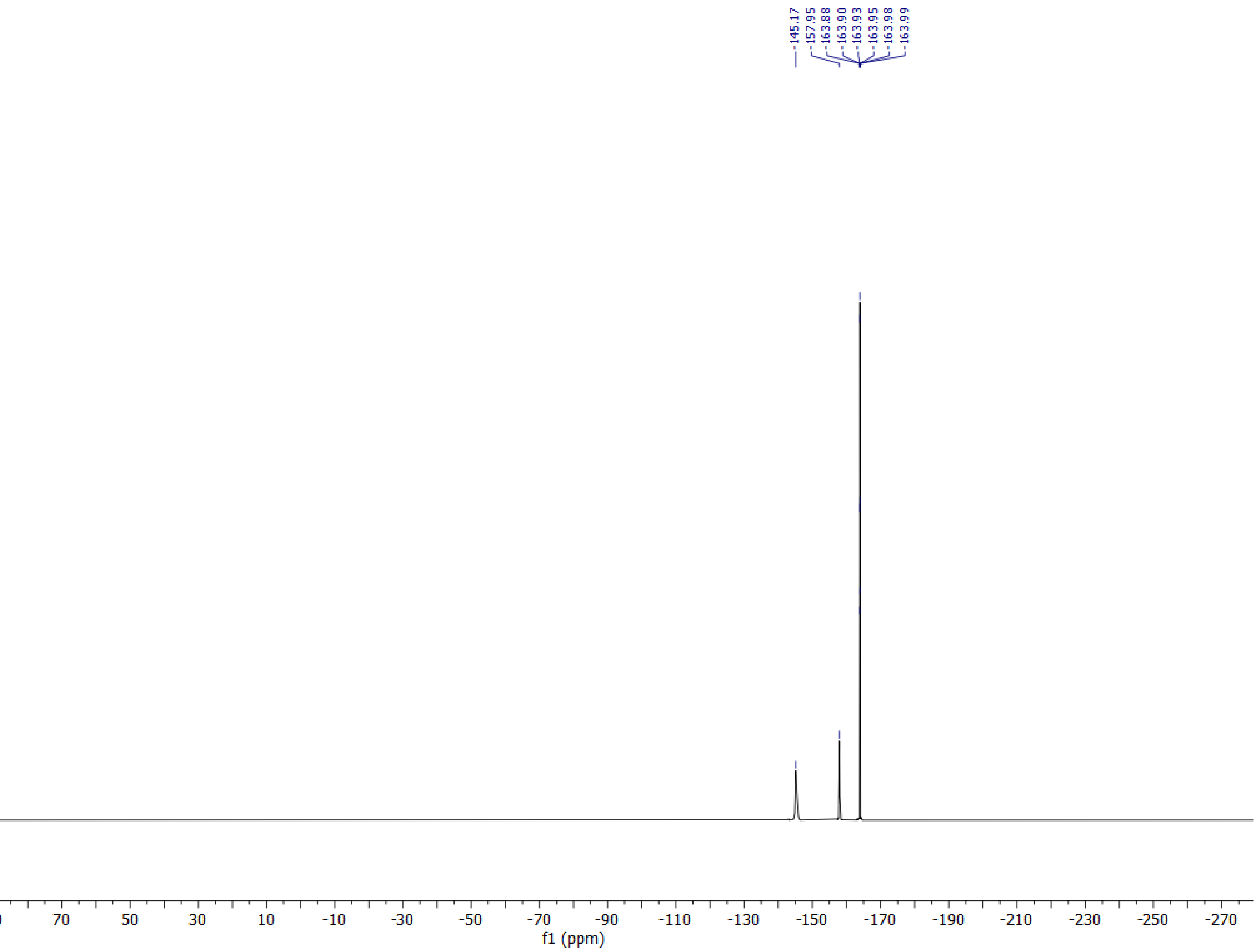

^{1}H NMR of *N*-Cbz-1-hydroxyl-2-perfluoropehnylethan-2-amine (8)

$CDCl_3$, 23 ºC

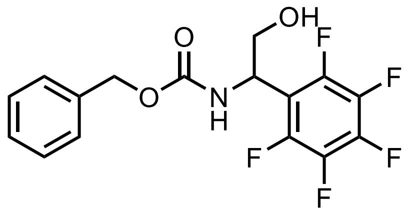

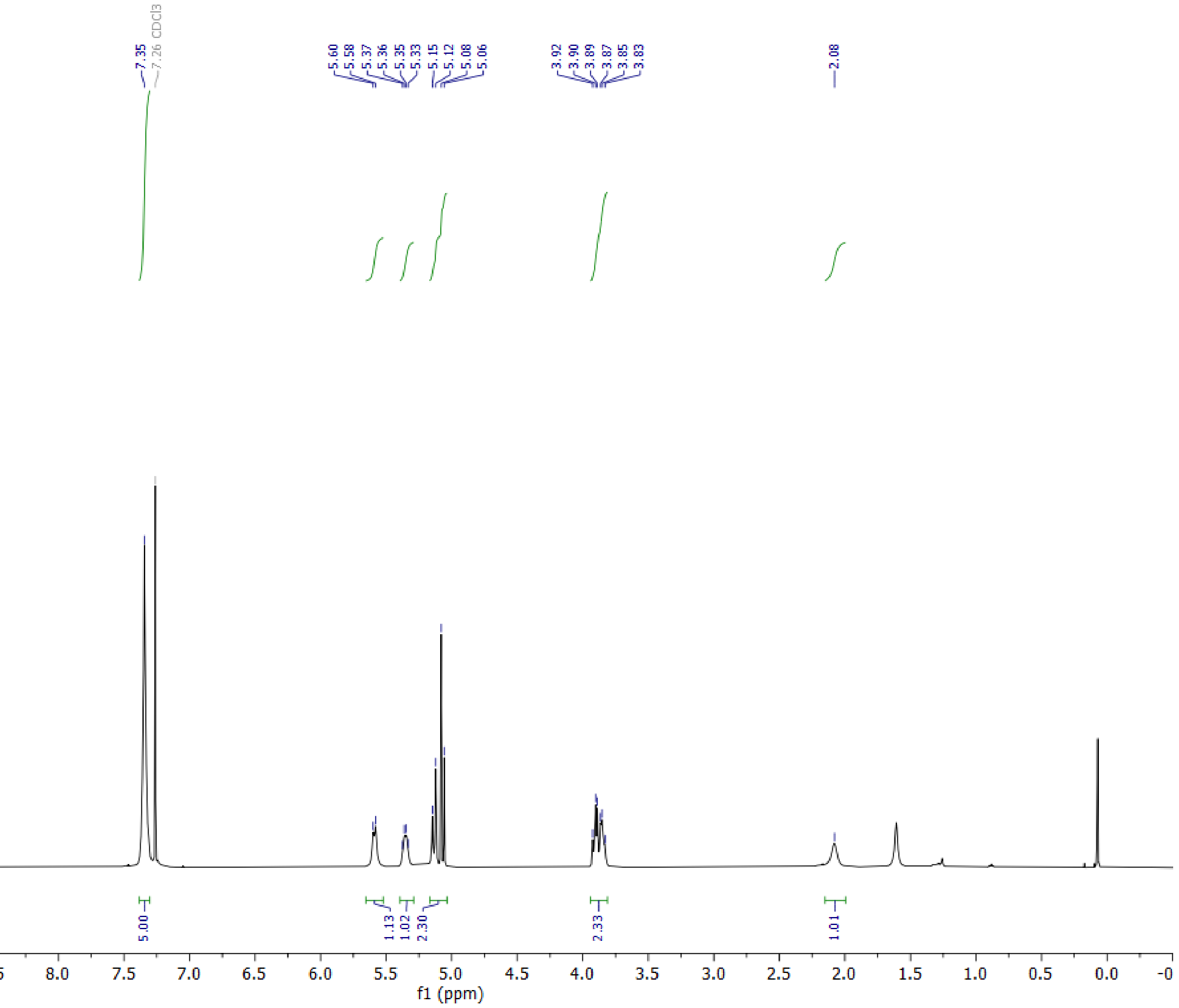

^{13}C NMR of *N*-Cbz-1-hydroxyl-2-perfluoropehnylethan-2-amine (8)

$CDCl_3$, 23 ºC

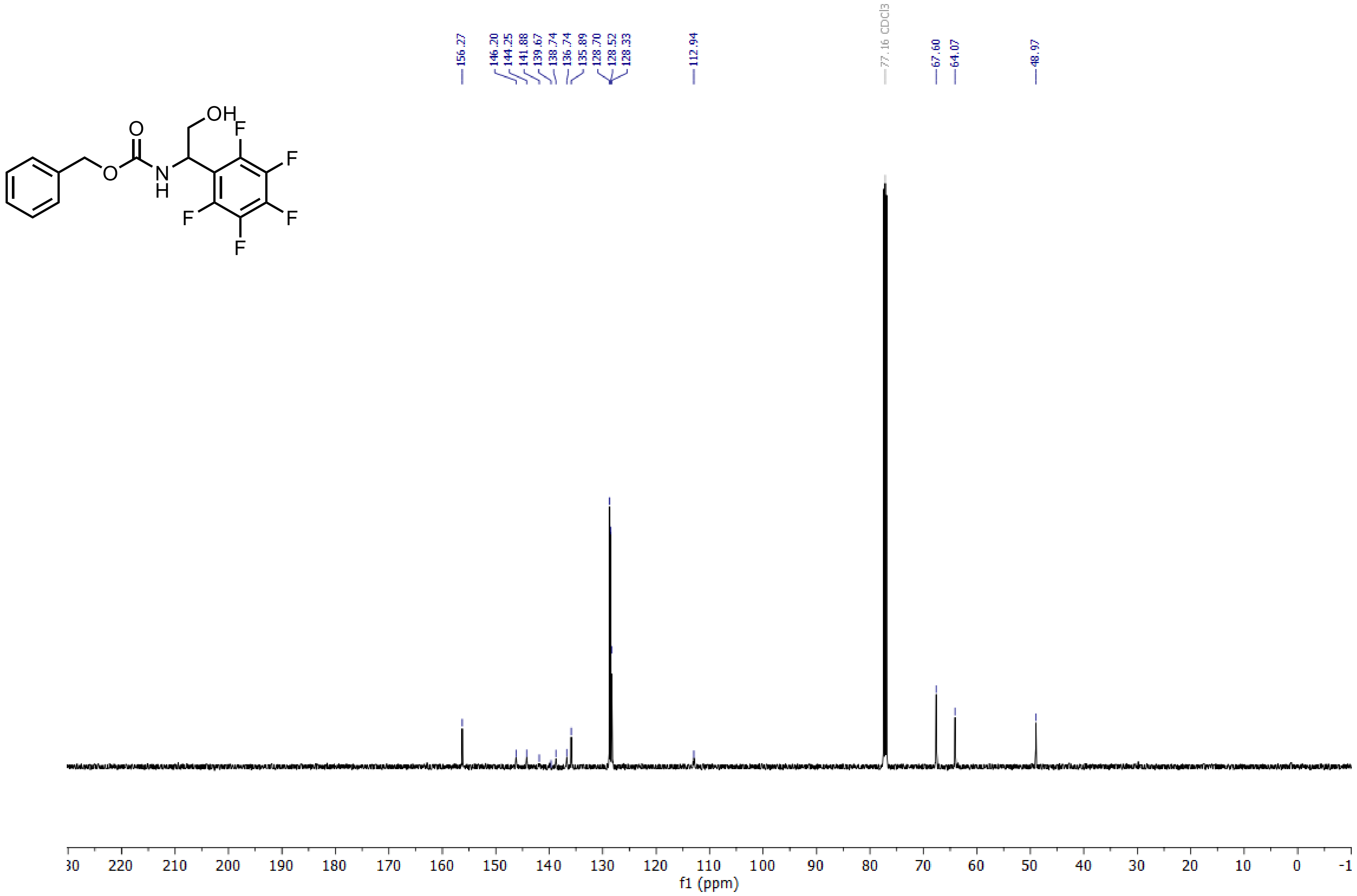

^{19}F NMR of *N*-Cbz-1-hydroxyl-2-perfluoropehnylethan-2-amine (8)

$CDCl_3$, 23 ºC

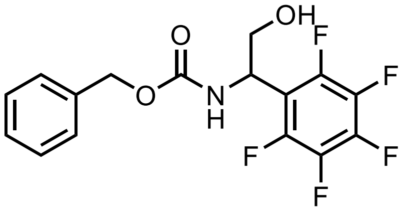

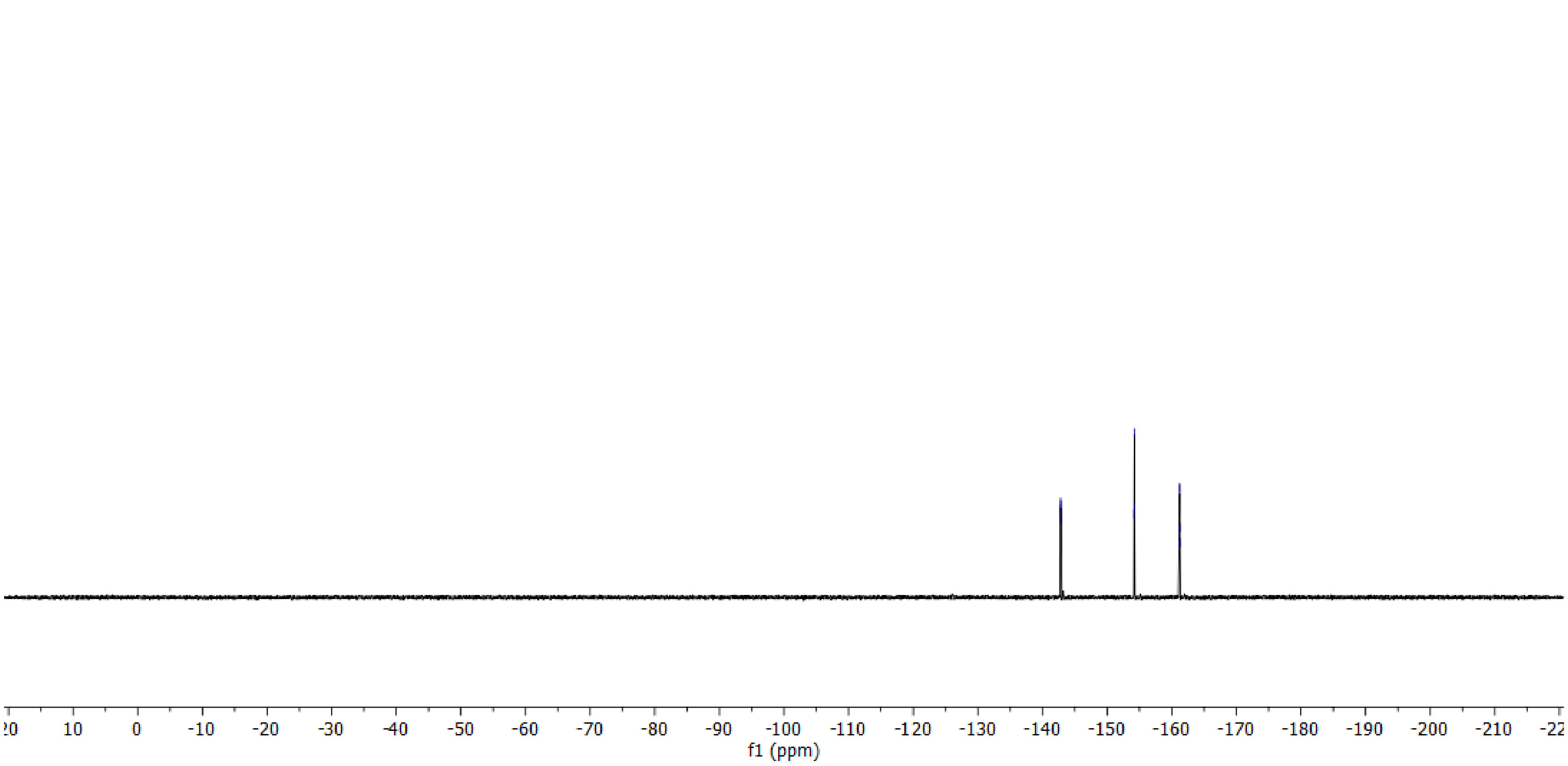

^{1}H NMR of MTV III derivative 9

$CDCl_3$, 23 ºC

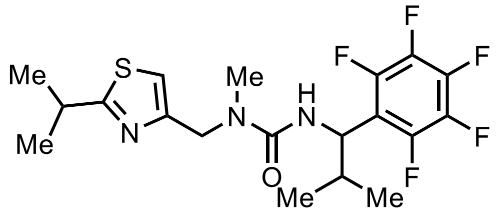

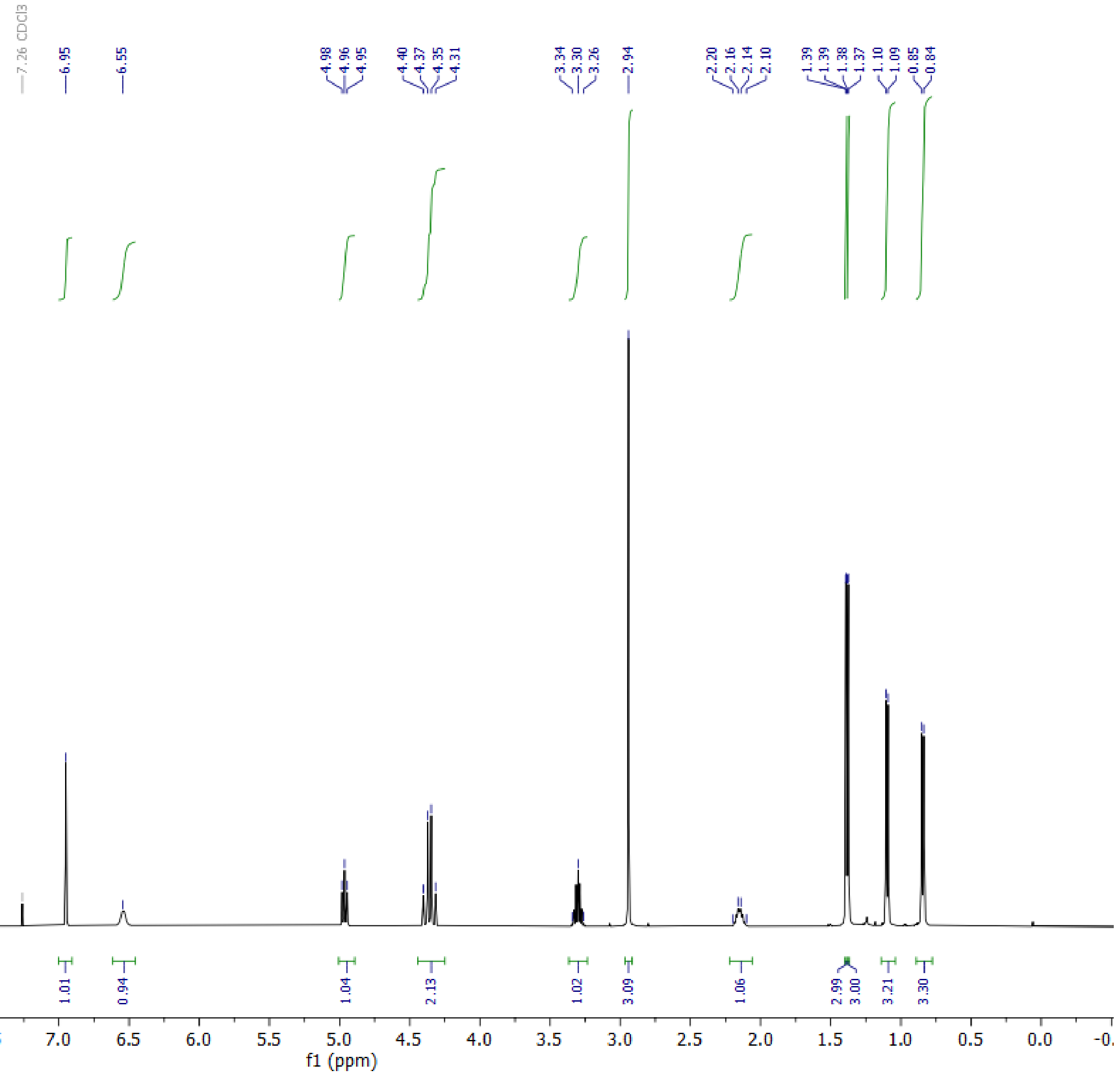

^{13}C NMR of MTV III derivative 9

CDCl$_3$, 23 ºC

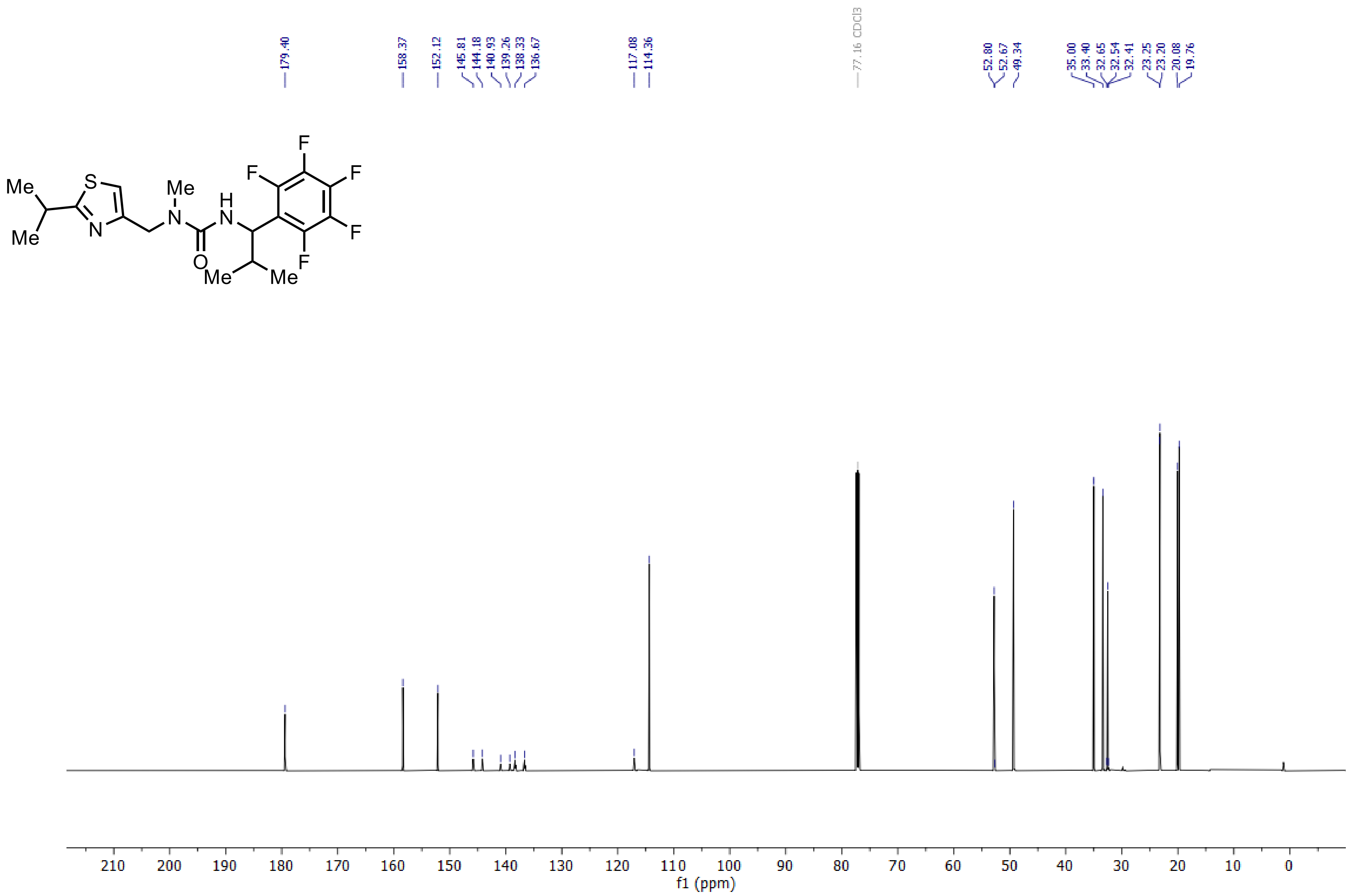

^{19}F NMR of MTV III derivative 9

$CDCl_3$, 23 ºC

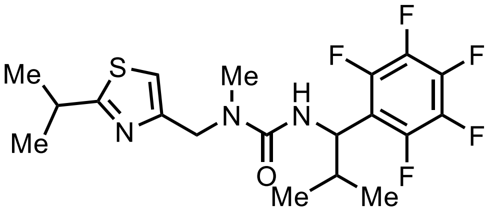

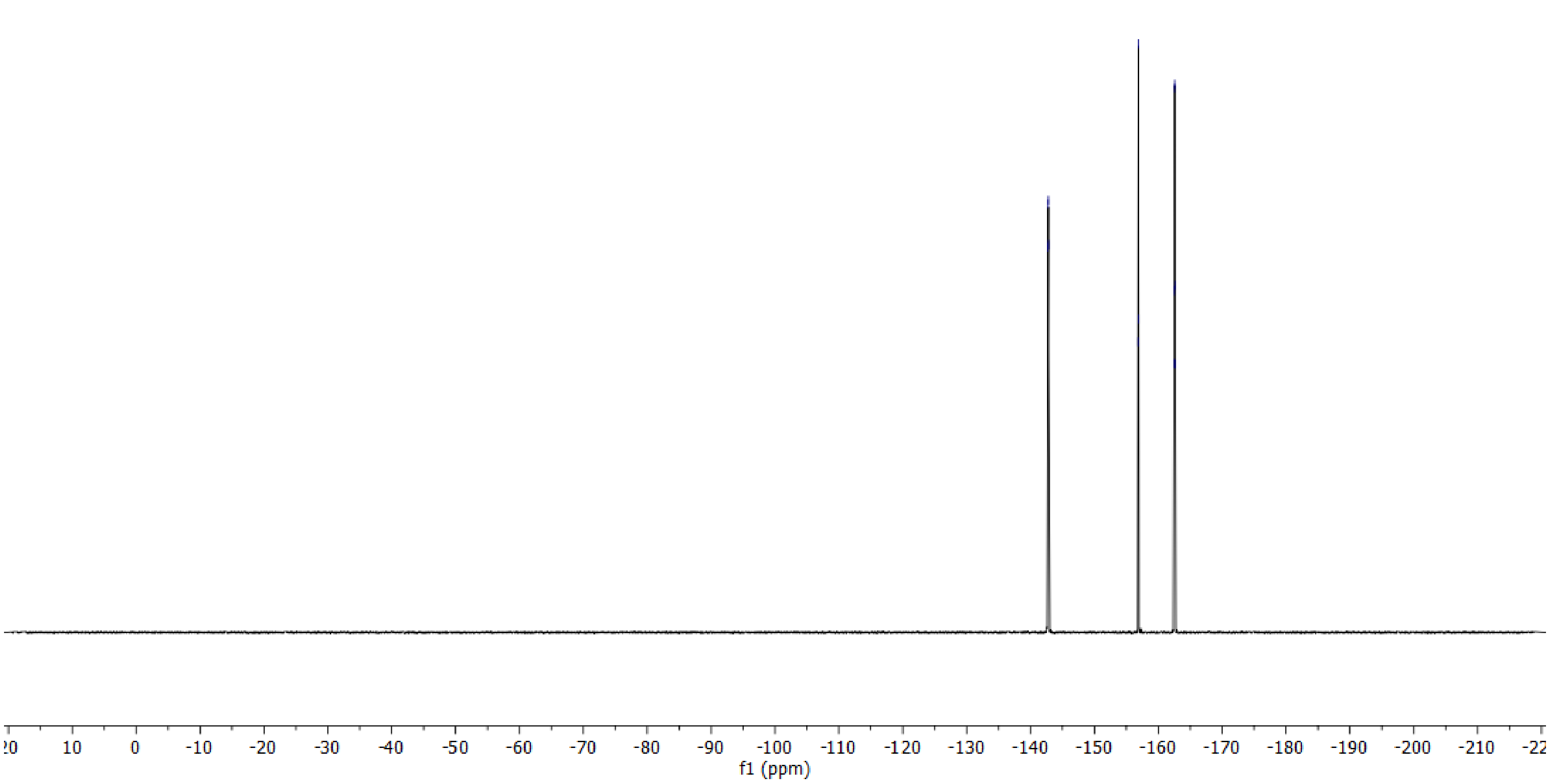

^{1}H NMR of 1-(Perfluorophenyl)ethanol (10)

$CDCl_3$, 23 ºC

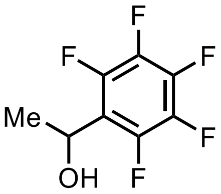

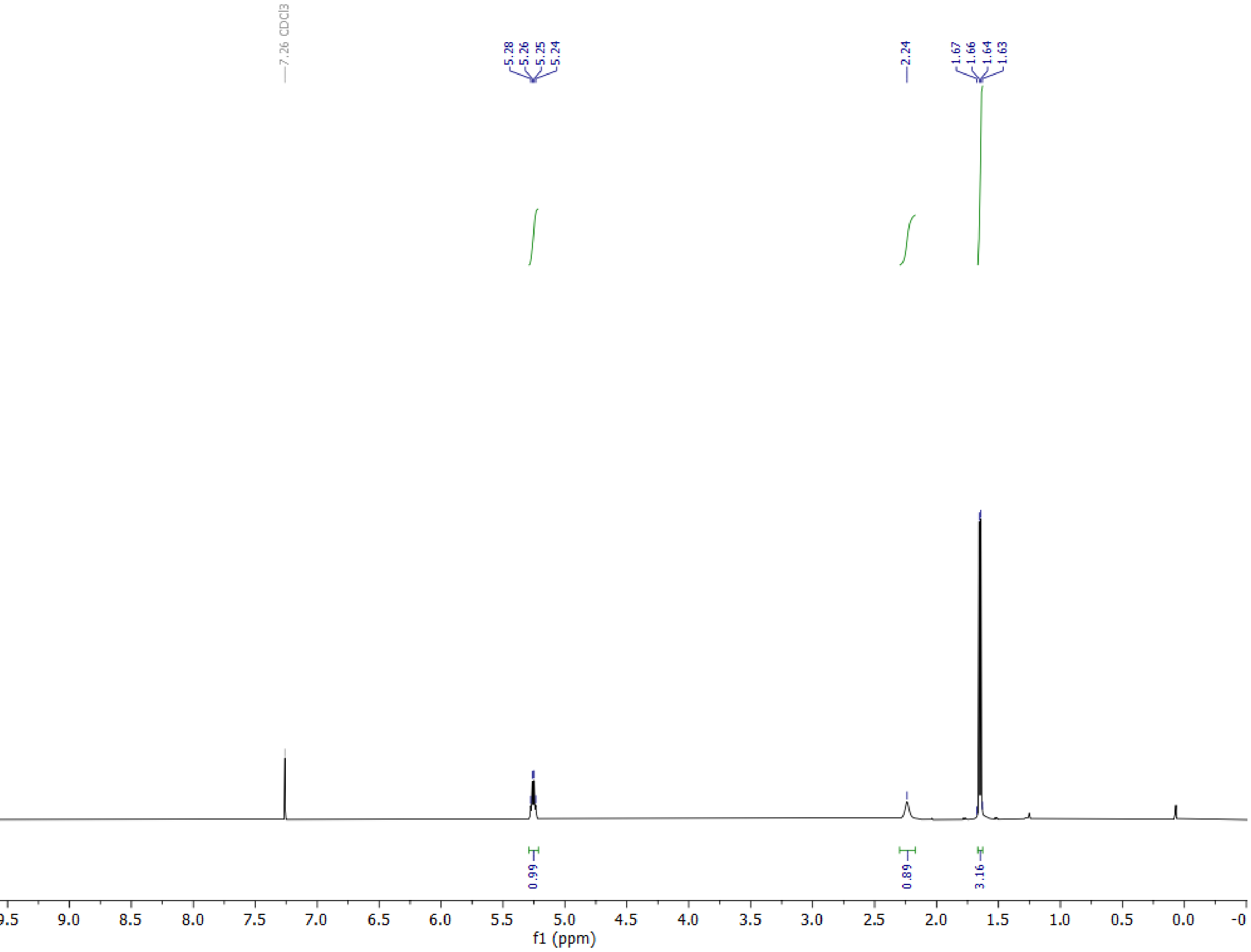

^{13}C NMR of 1-(Perfluorophenyl)ethanol (10)

$CDCl_3$, 23 ºC

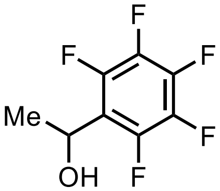

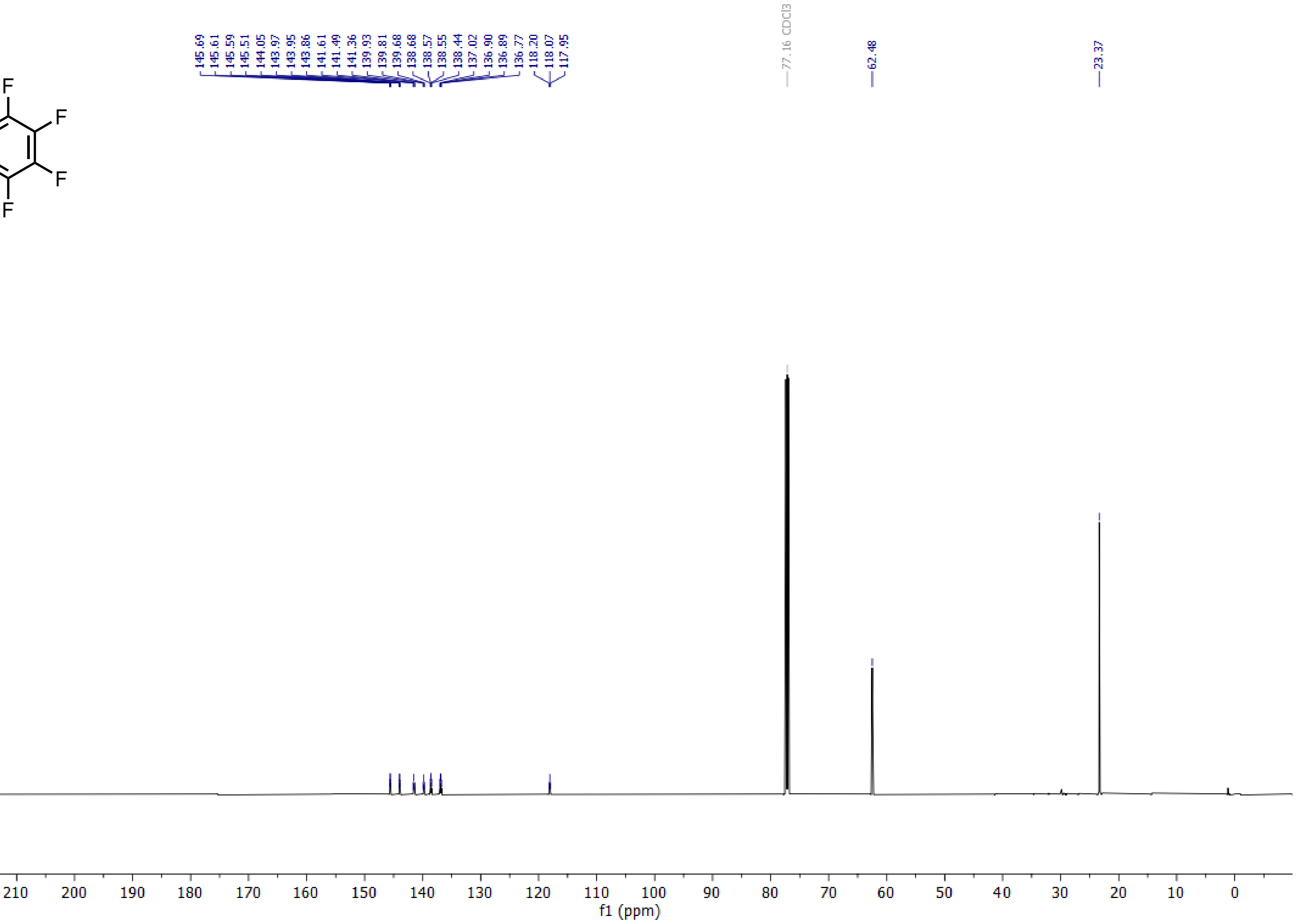

^{19}F NMR of 1-(Perfluorophenyl)ethanol (10)

CDCl$_3$, 23 ºC

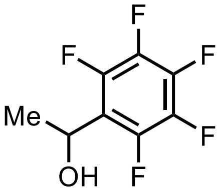

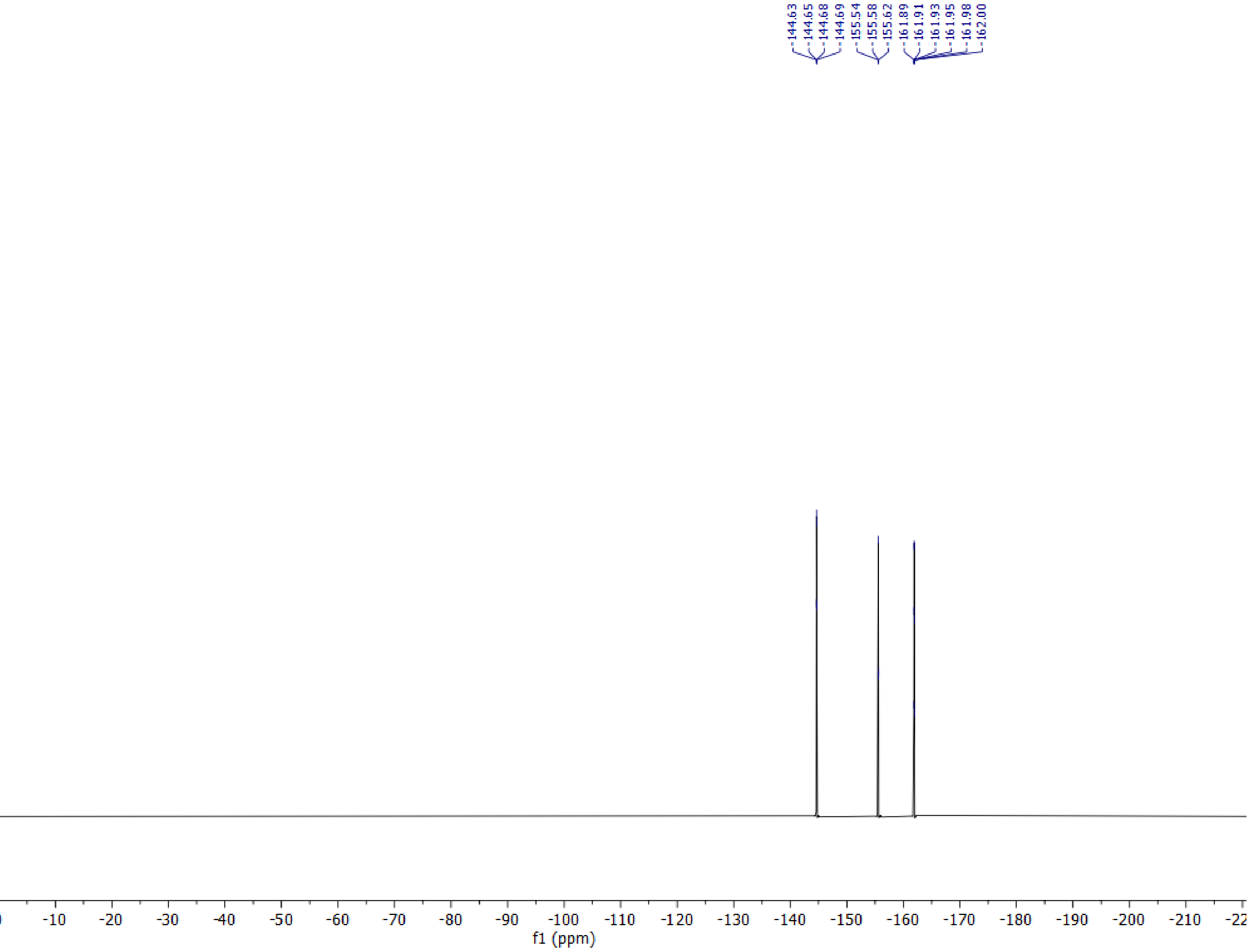

^{1}H NMR of 6-Fluorochromane-2-pentafluorobenzene (11)

$CDCl_3$, 23 ºC

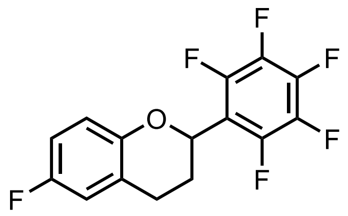

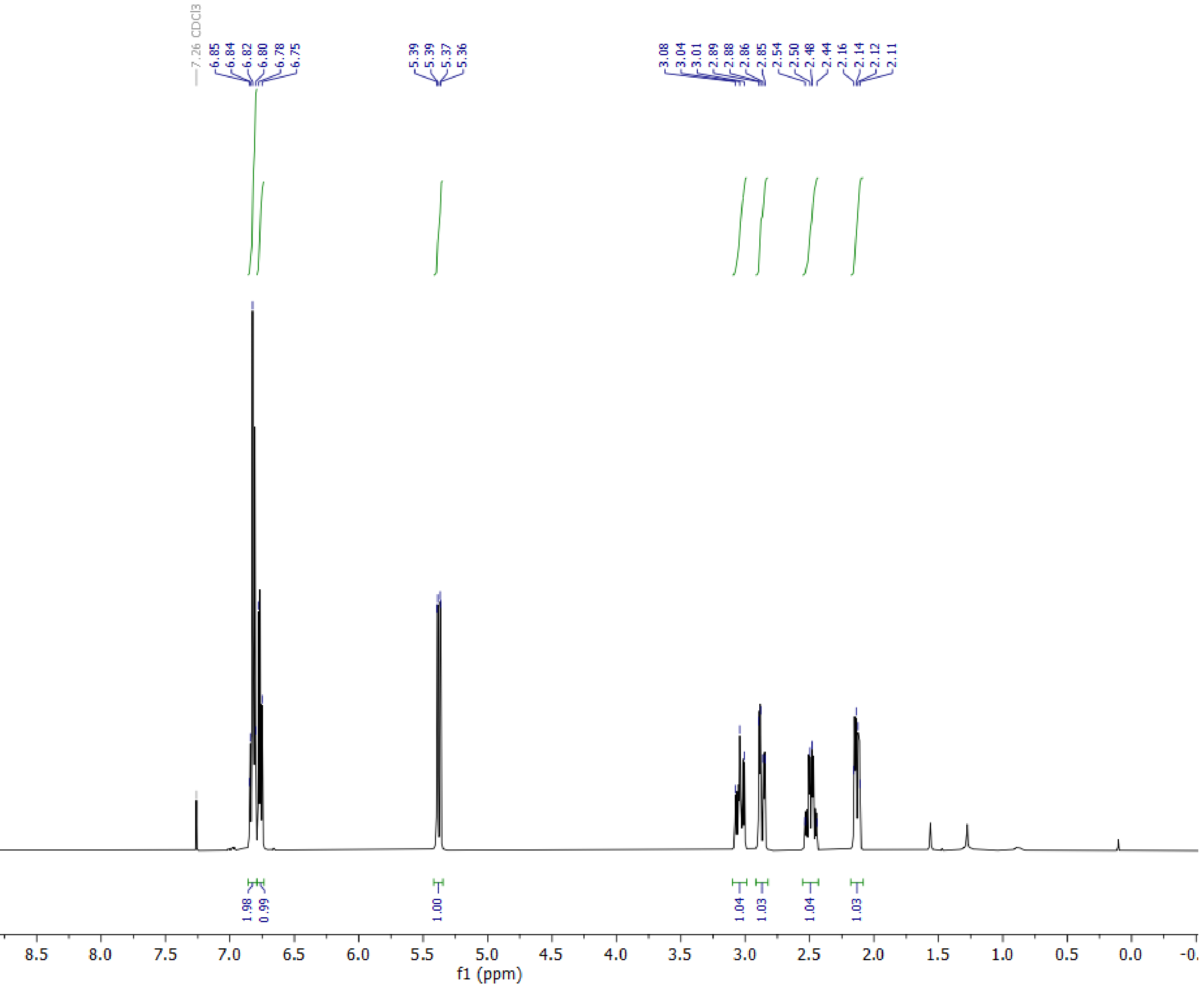

^{13}C NMR of 6-Fluorochromane-2-pentafluorobenzene (11)

$CDCl_3$, 23 ºC

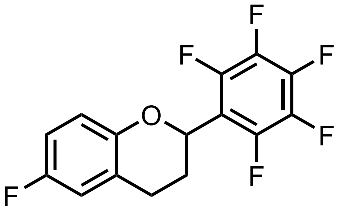

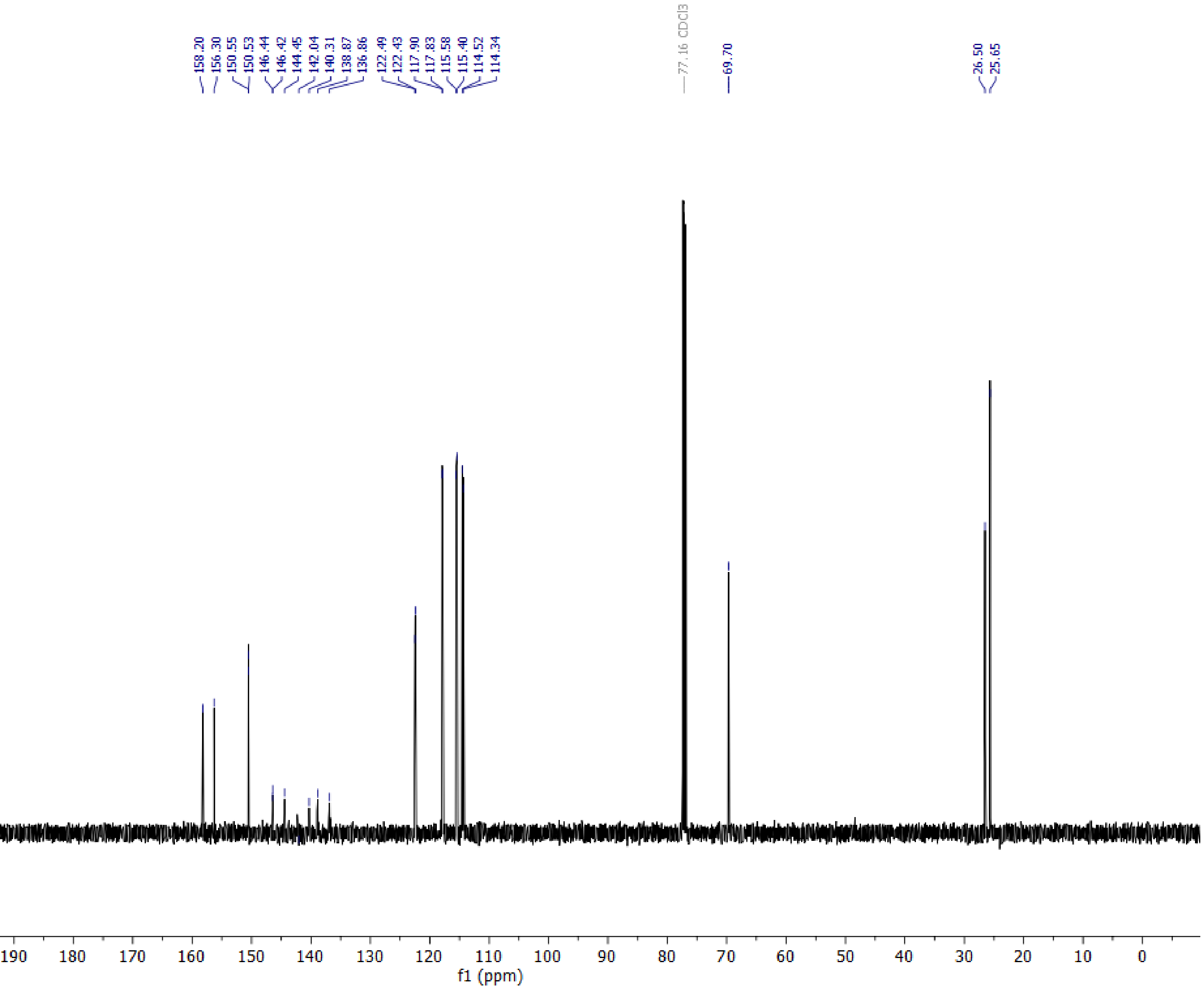

^{19}F NMR of 6-Fluorochromane-2-pentafluorobenzene (11)

$CDCl_3$, 23 ºC

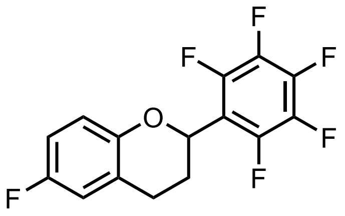

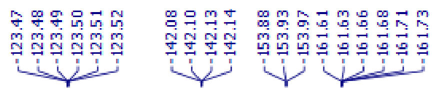

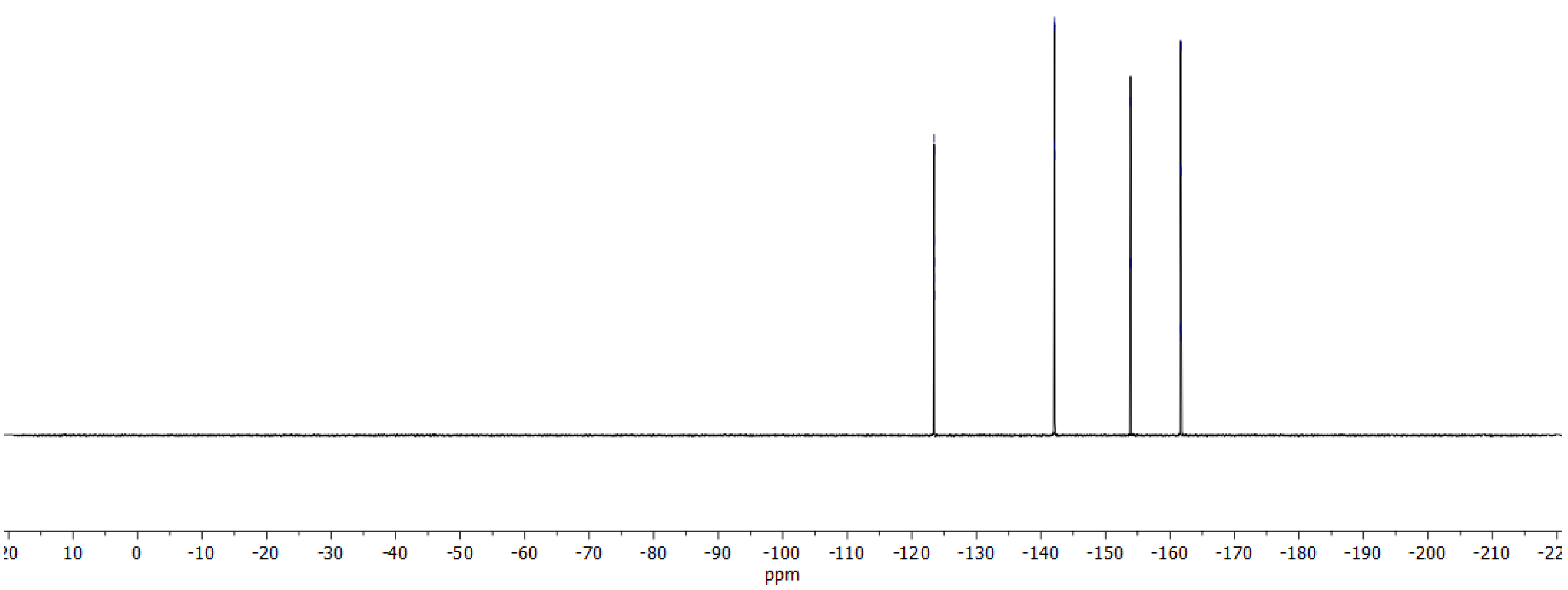

^{1}H NMR of 2,2-Dimethyl-4-(perfluorophenyl)-1,3-dioxolane (12)

$CDCl_3$, 23 ºC

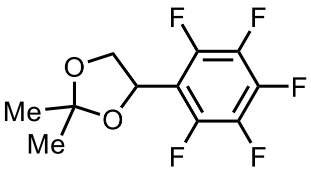

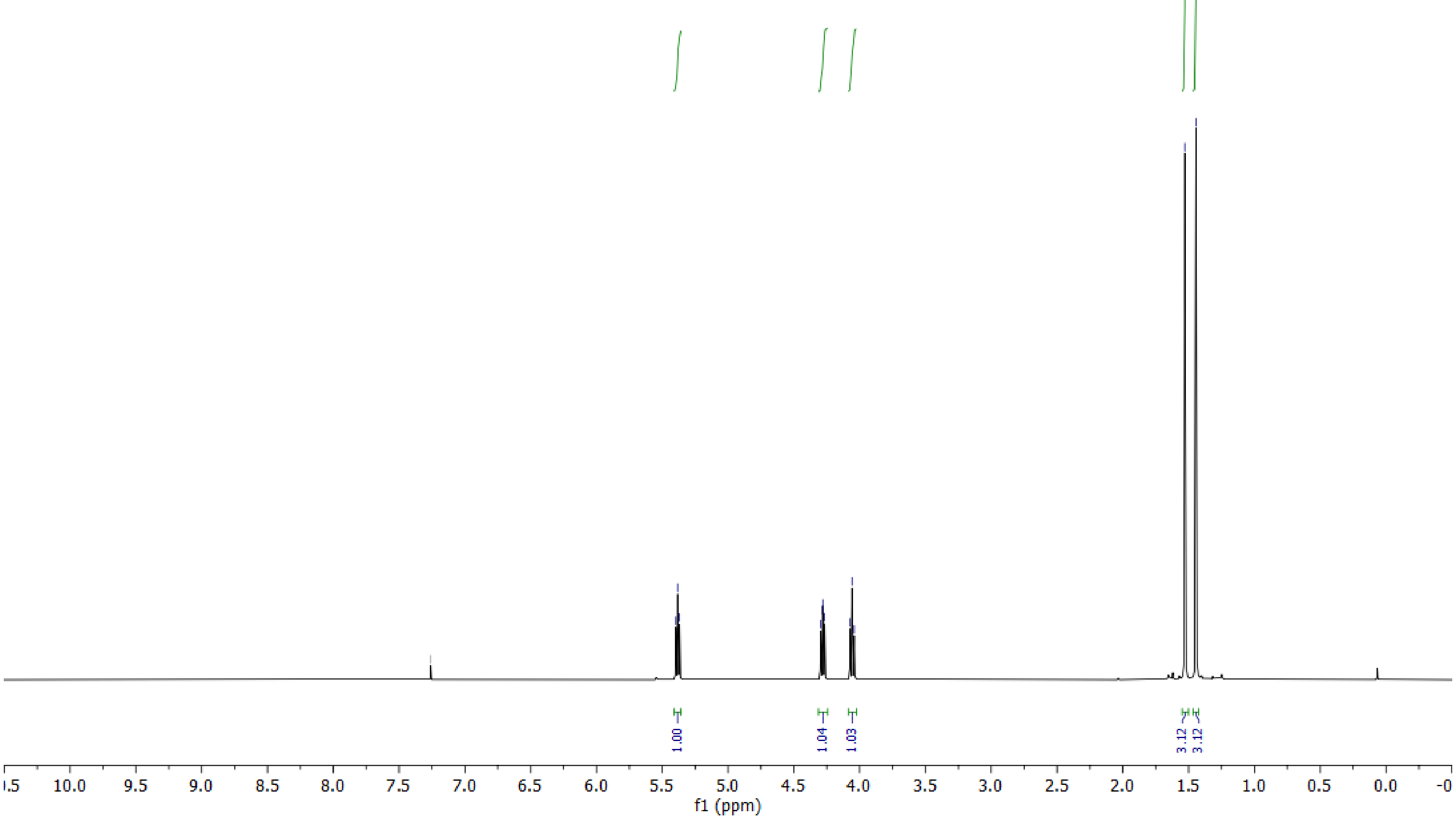

^{13}C NMR of 2,2-Dimethyl-4-(perfluorophenyl)-1,3-dioxolane (12)

$CDCl_3$, 23 ºC

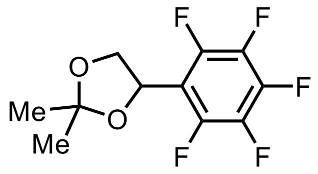

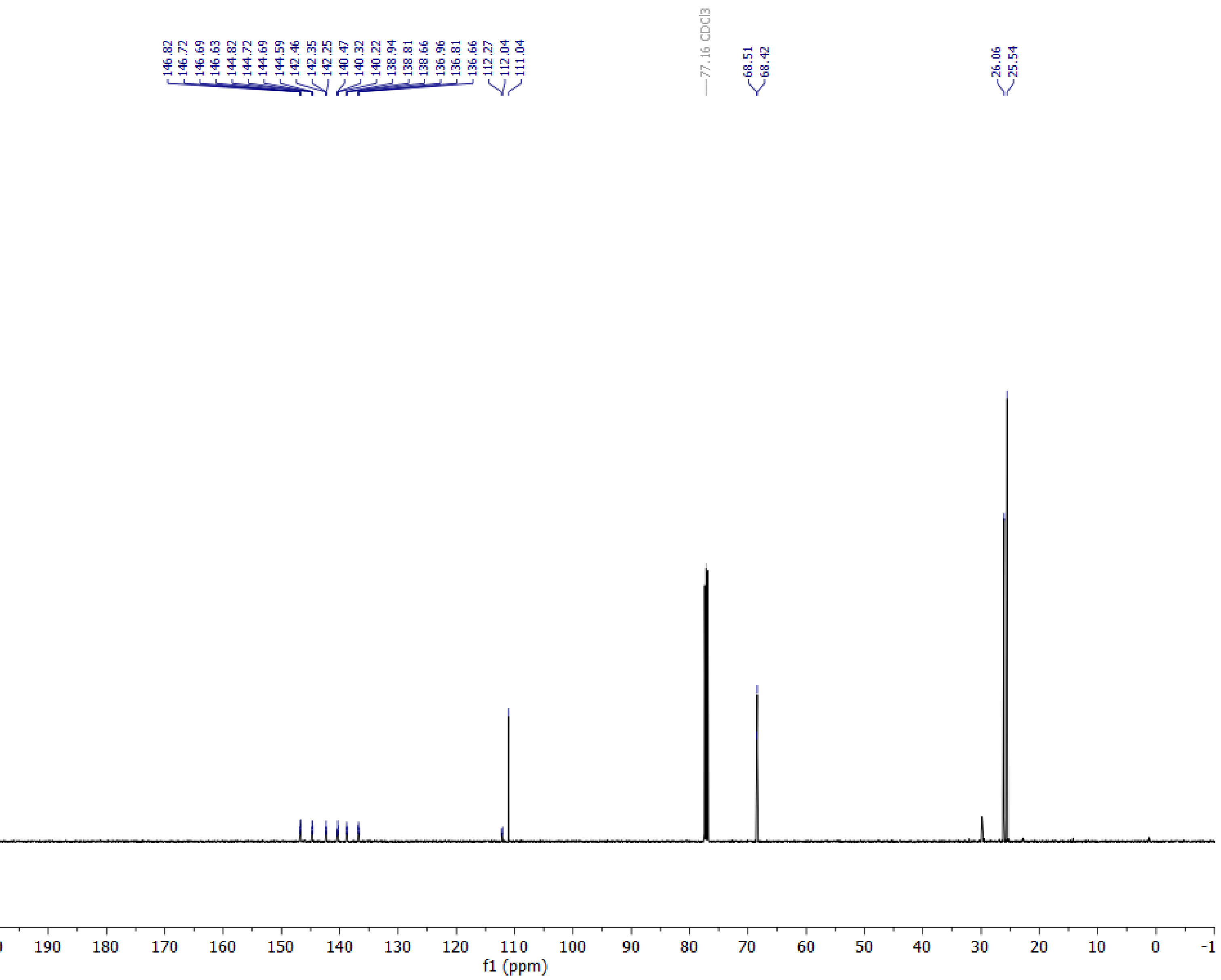

^{19}F NMR of 2,2-Dimethyl-4-(perfluorophenyl)-1,3-dioxolane (12)

$CDCl_3$, 23 ºC

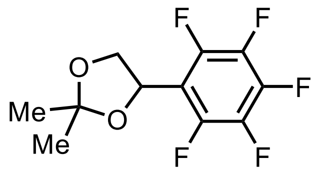

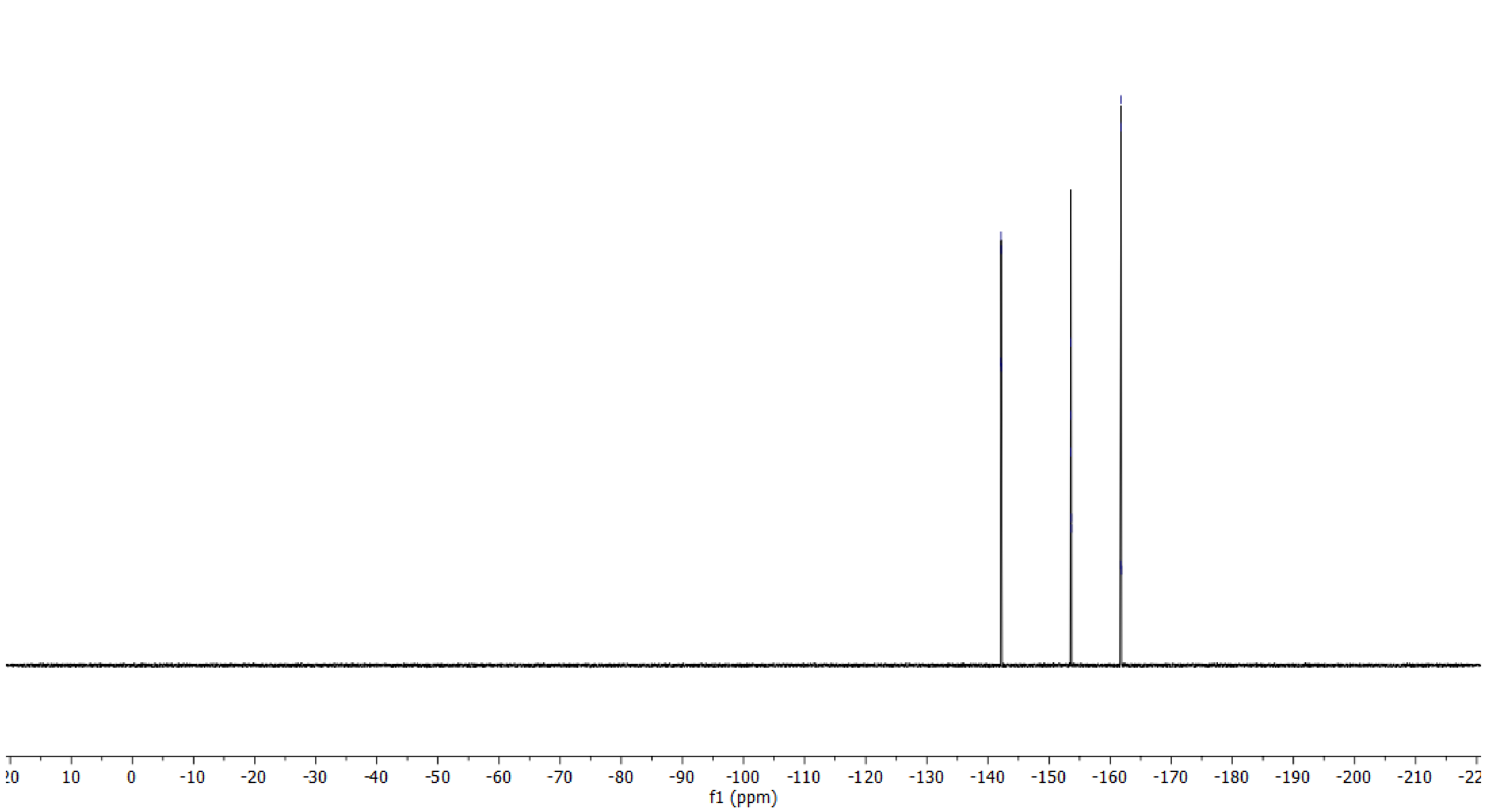

^{1}H NMR of Bezafibrate derivative 13

$CDCl_3$, 23 ºC

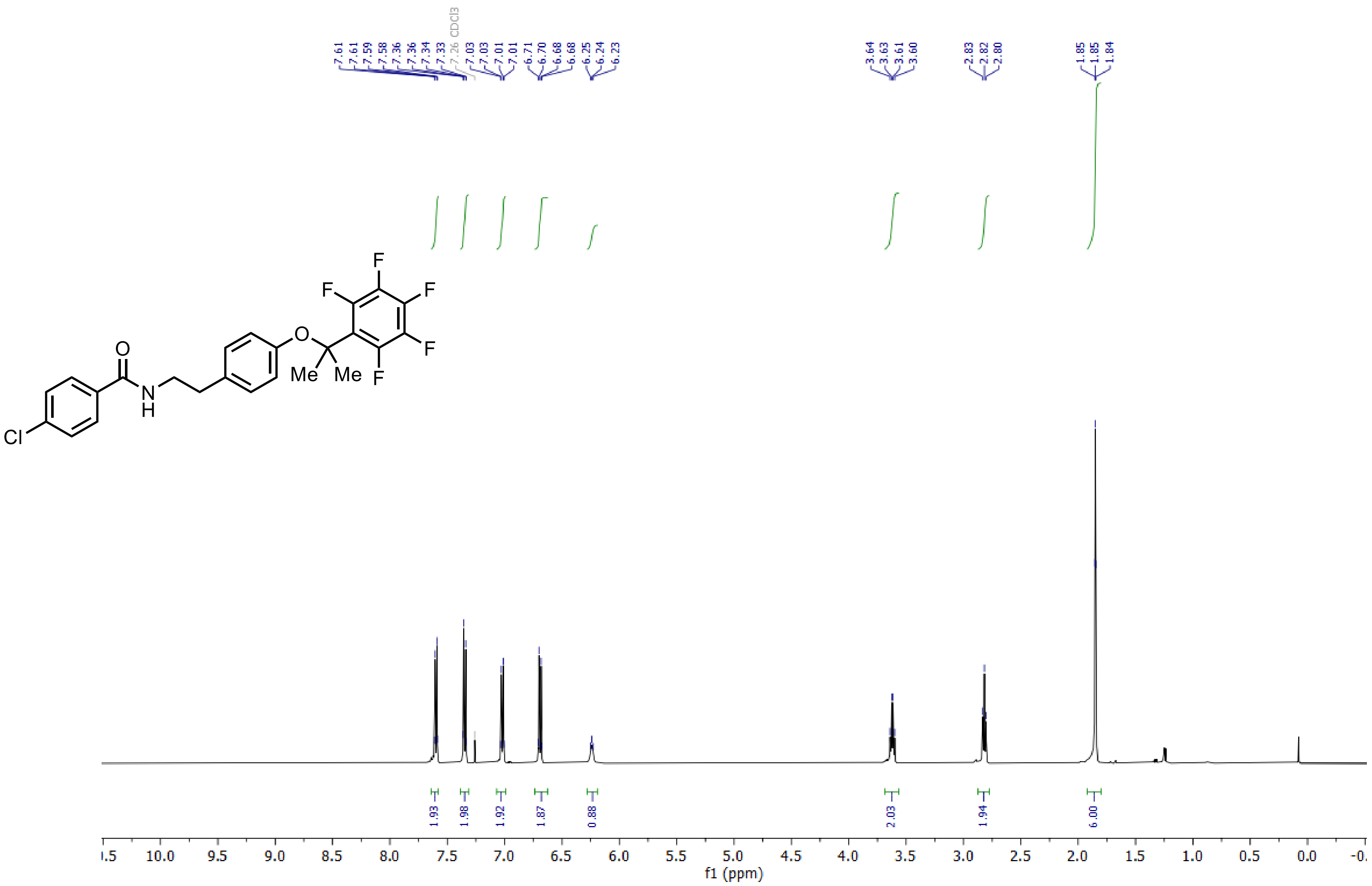

^{13}C NMR of Bezafibrate derivative 13

$CDCl_3$, 23 ºC

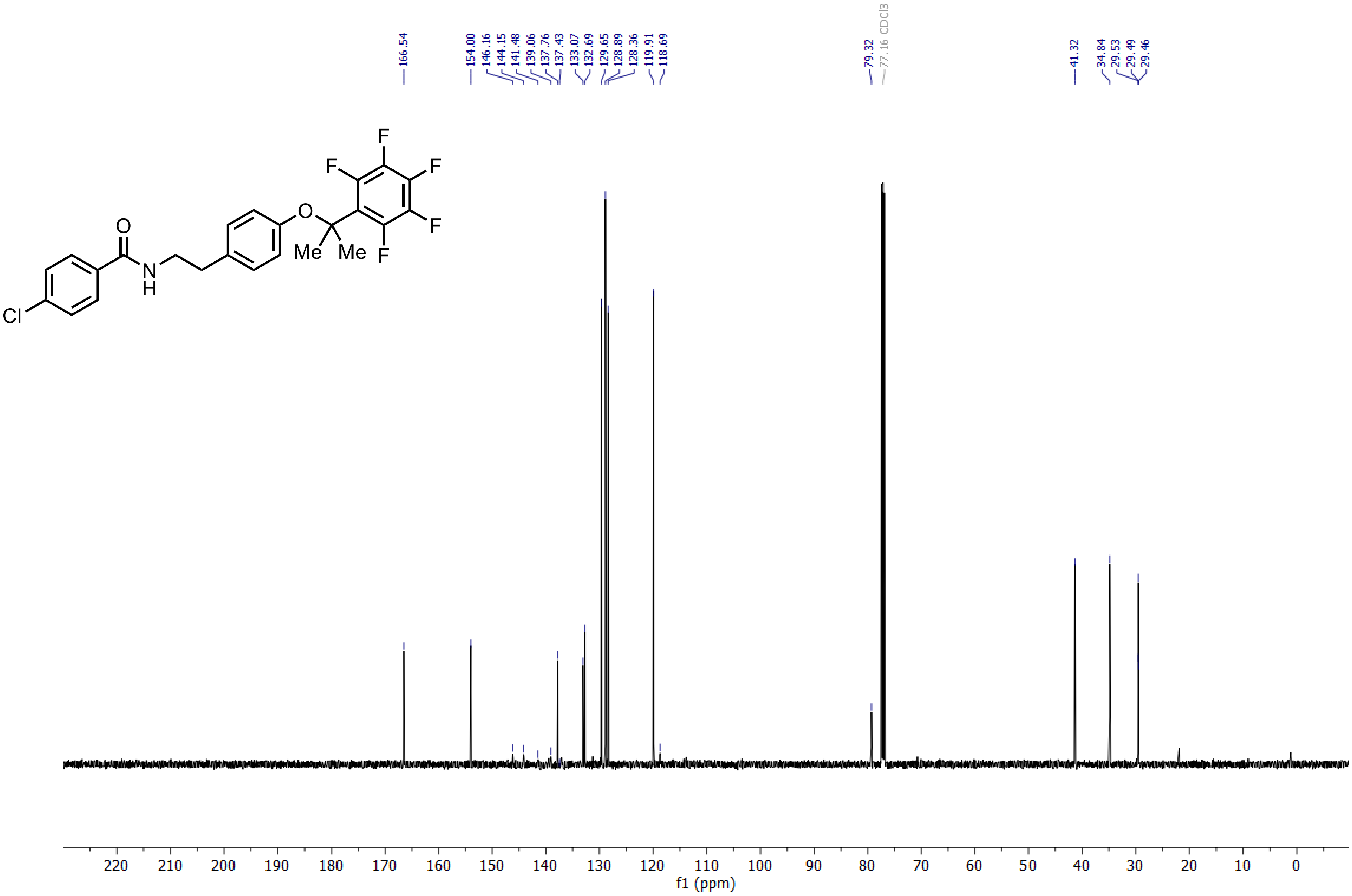

^{19}F NMR of Bezafibrate derivative 13

CDCl$_3$, 23 ºC

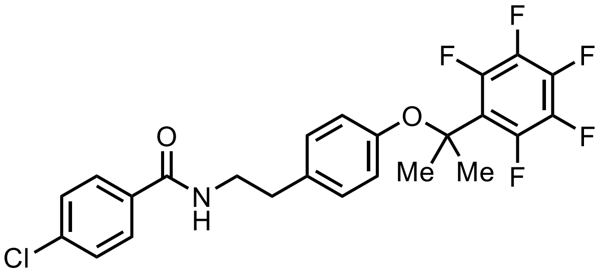

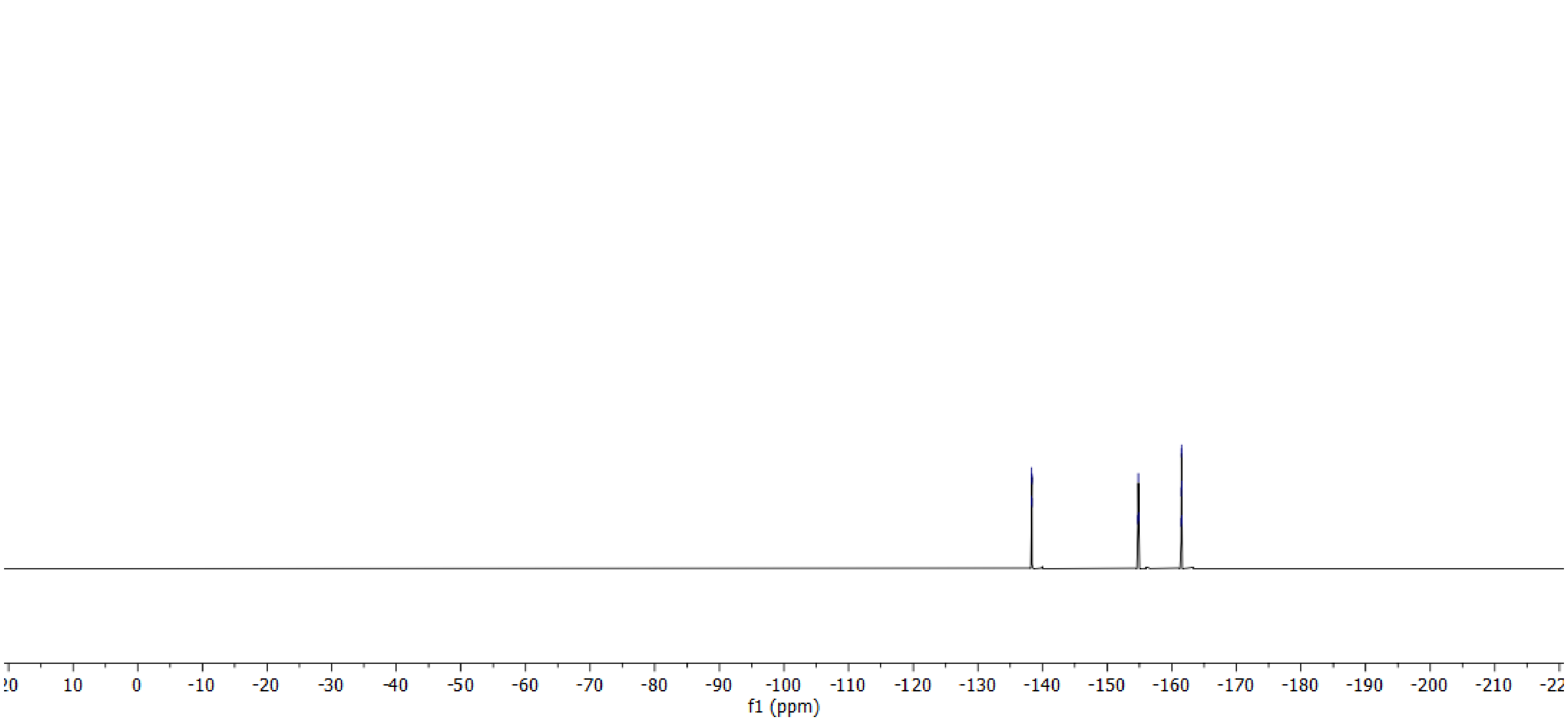

^{1}H NMR of 1-(Perfluorophenyl)adamantane (14)

$CDCl_3$, 23 ºC

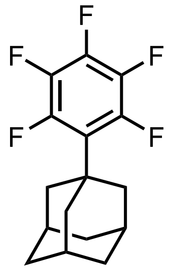

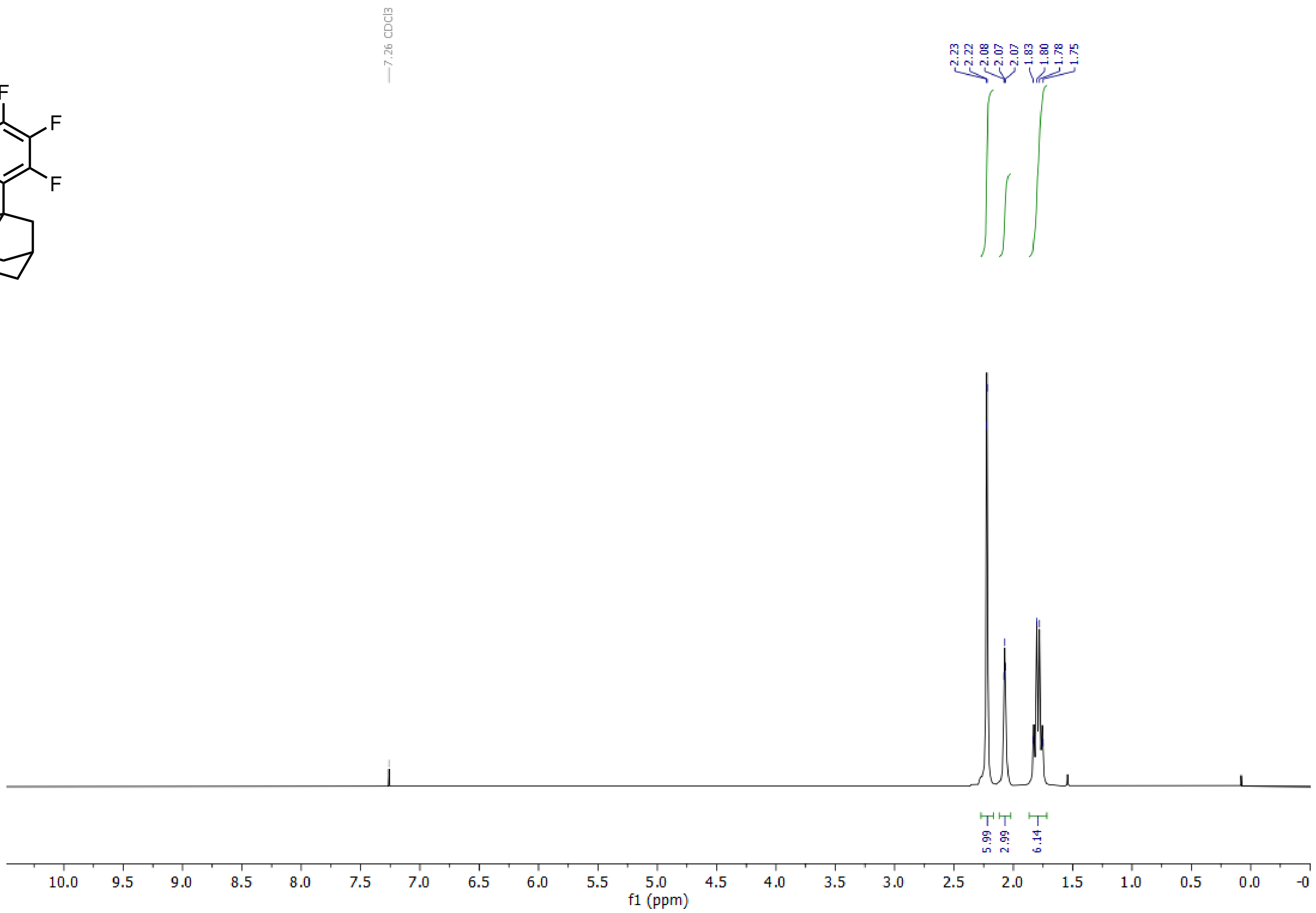

^{13}C NMR of 1-(Perfluorophenyl)adamantane (14)

$CDCl_3$, 23 ºC

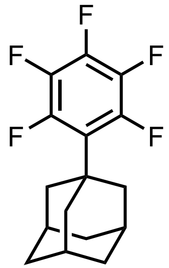

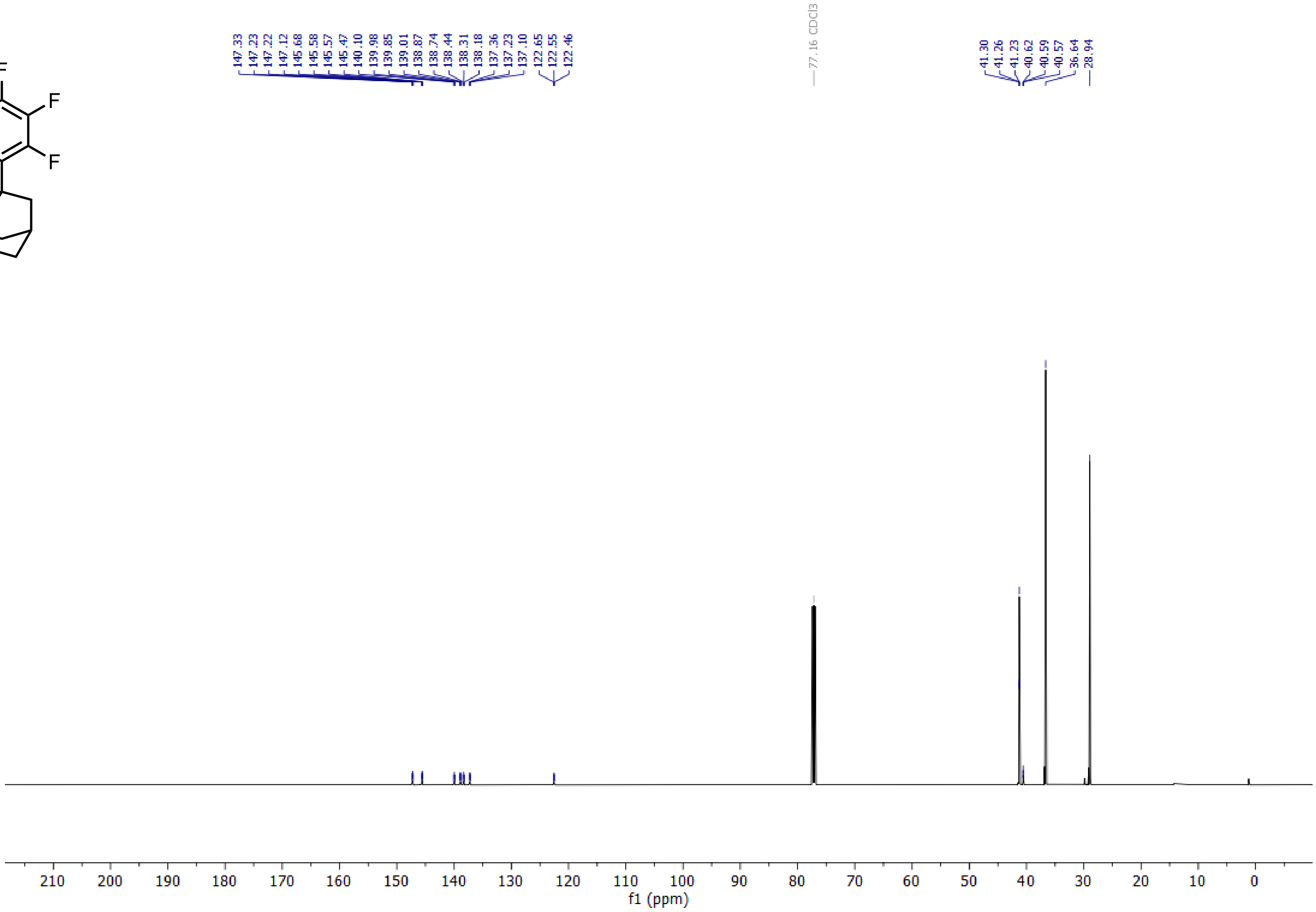

^{19}F NMR of 1-(Perfluorophenyl)adamantane (14)

$CDCl_3$, 23 ºC

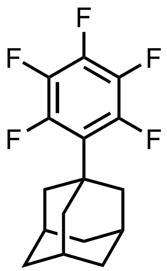

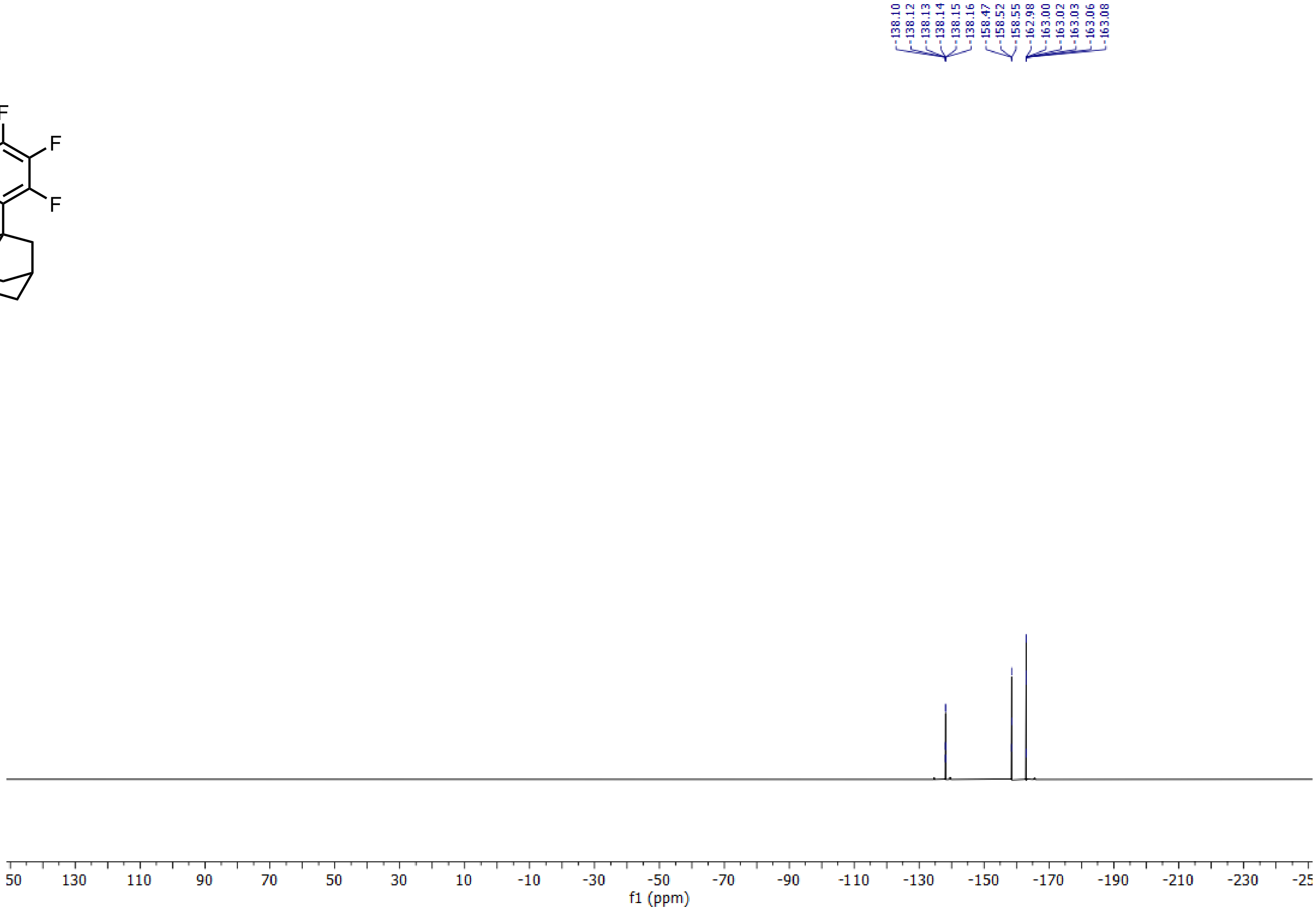

^{1}H NMR of Pentafluoro-(2-phenylpropyl)benzene (15)

$CDCl_3$, 23 ºC

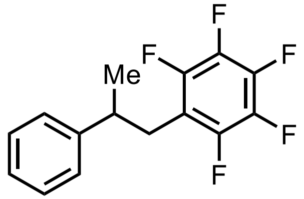

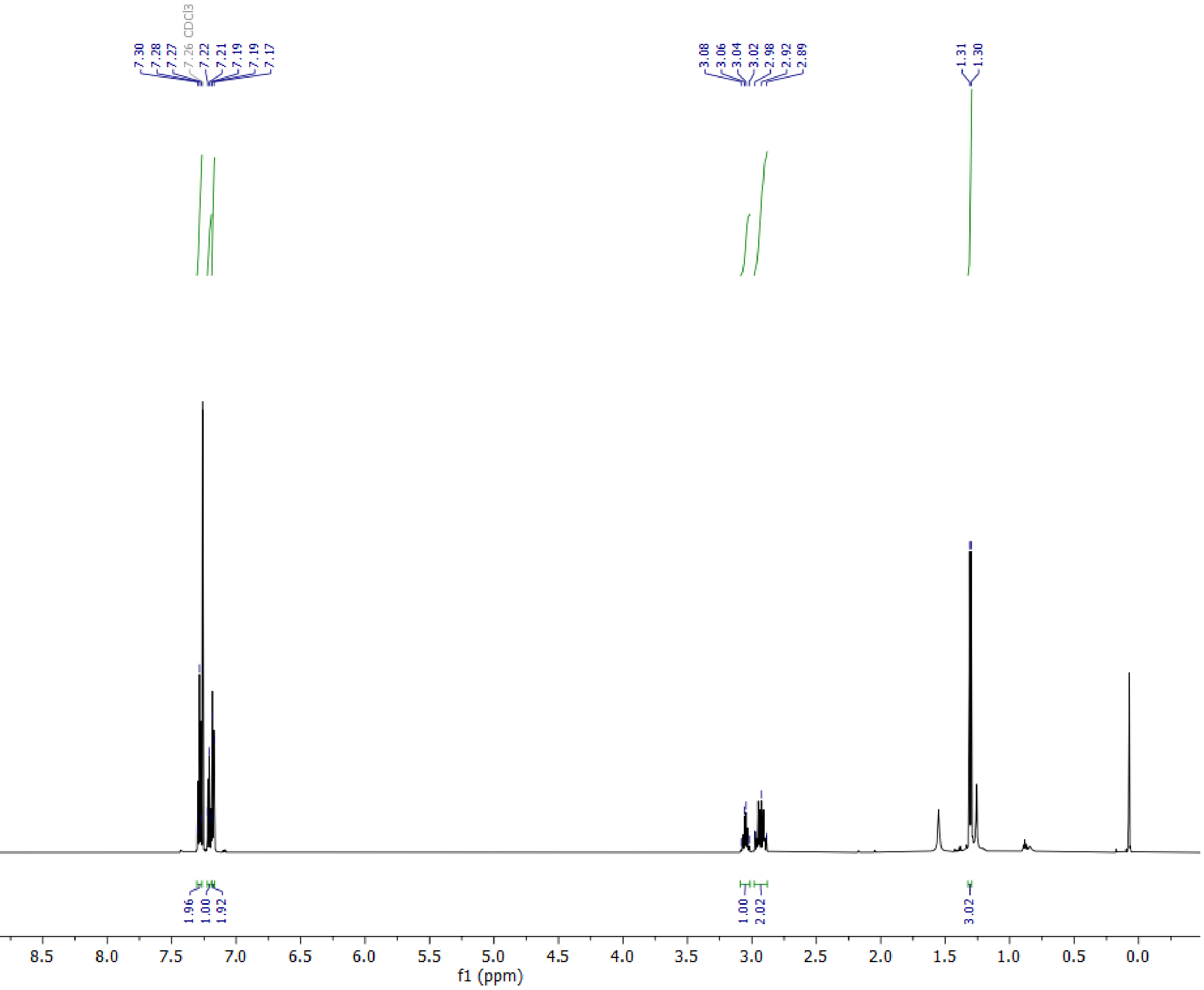

^{13}C NMR of Pentafluoro-(2-phenylpropyl)benzene (15)

$CDCl_3$, 23 ºC

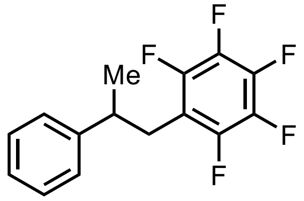

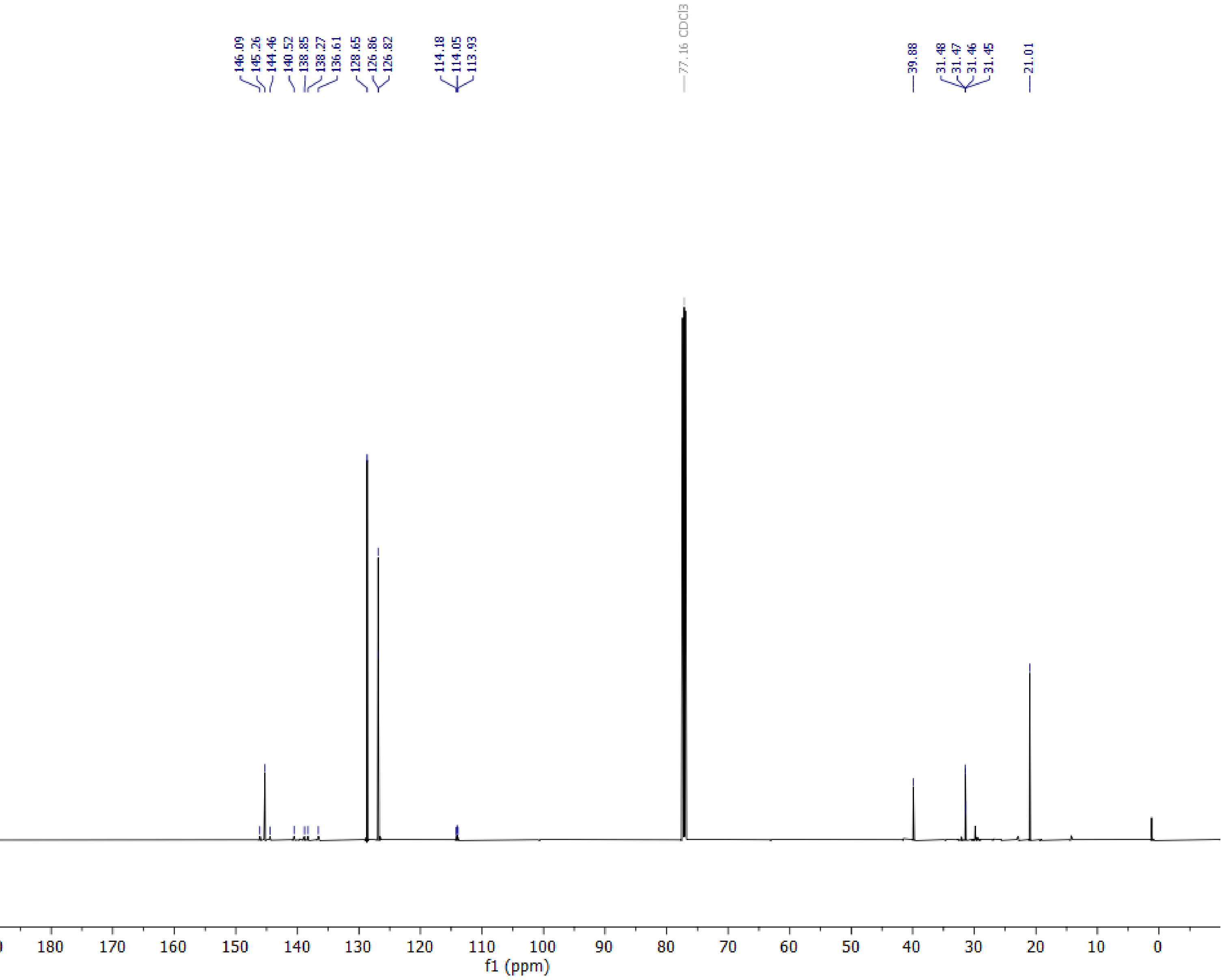

^{19}F NMR of Pentafluoro-(2-phenylpropyl)benzene (15)

$CDCl_3$, 23 ºC

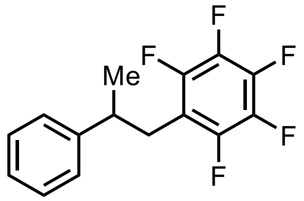

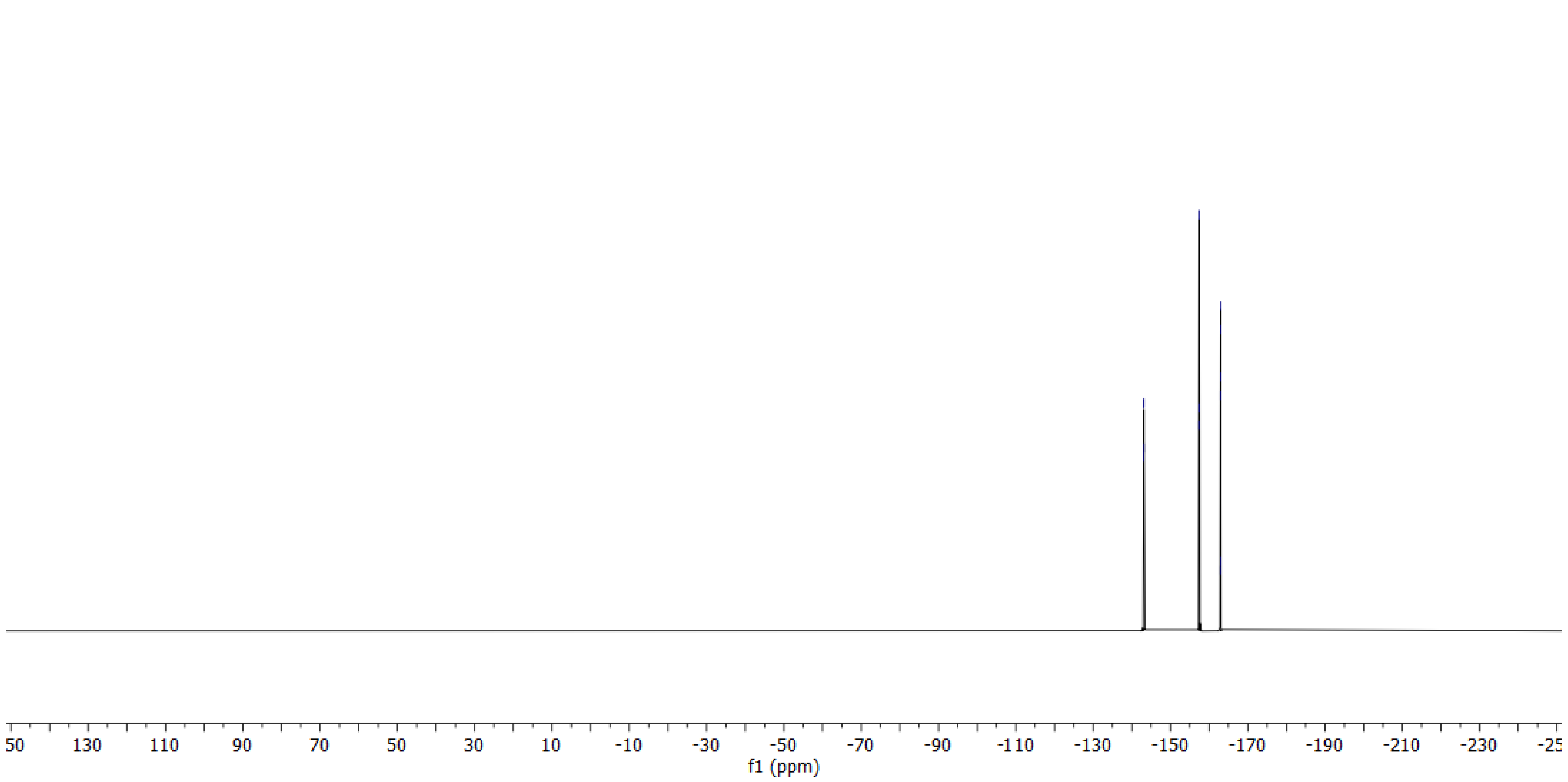

^{1}H NMR of 1,3-Bis(perfluorophenyl)adamantane (16)

$CDCl_3$, 23 ºC

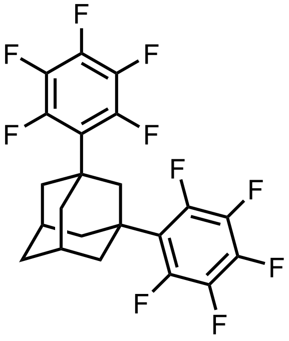

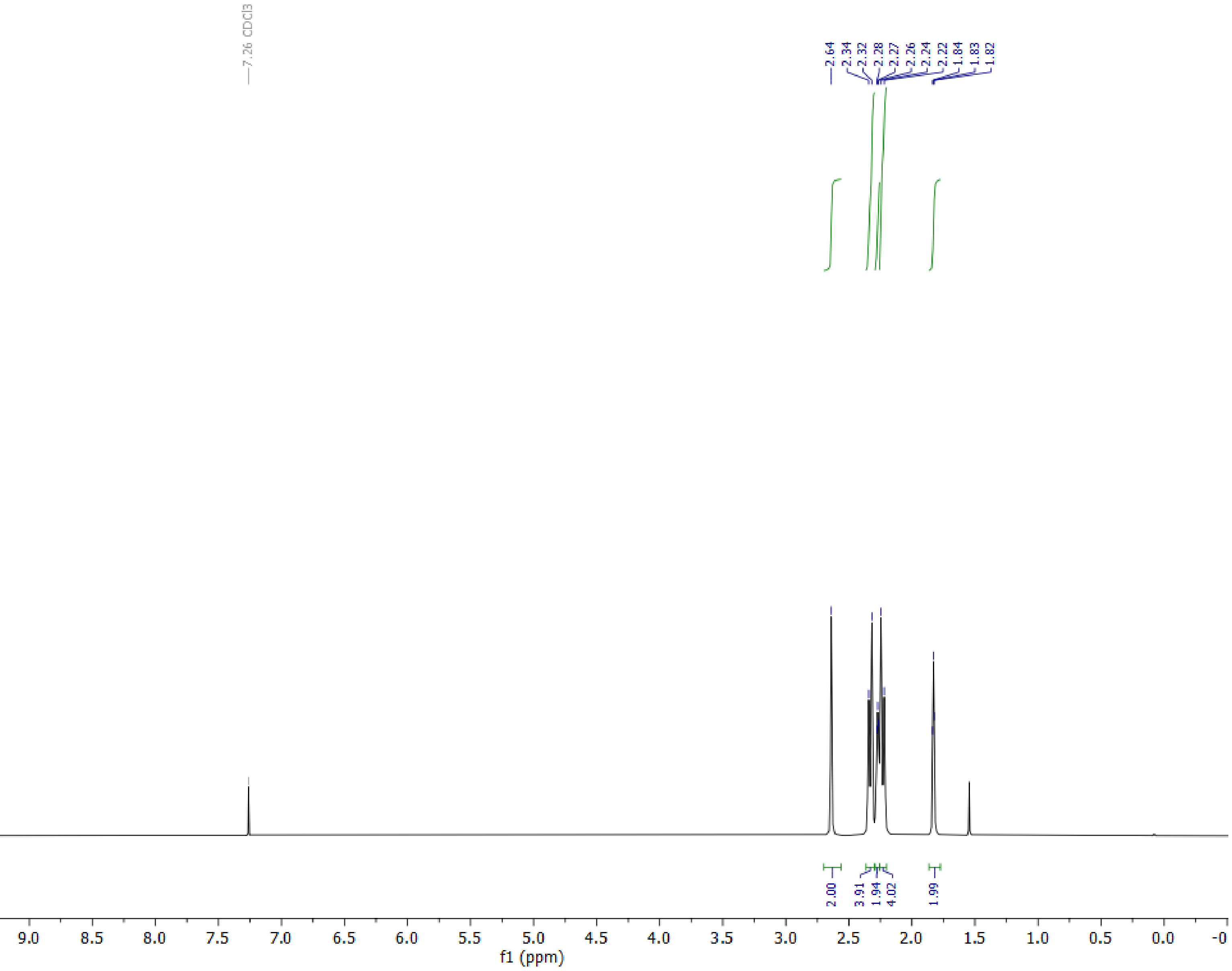

^{13}C NMR of 1,3-Bis(perfluorophenyl)adamantane (16)

$CDCl_3$, 23 ºC

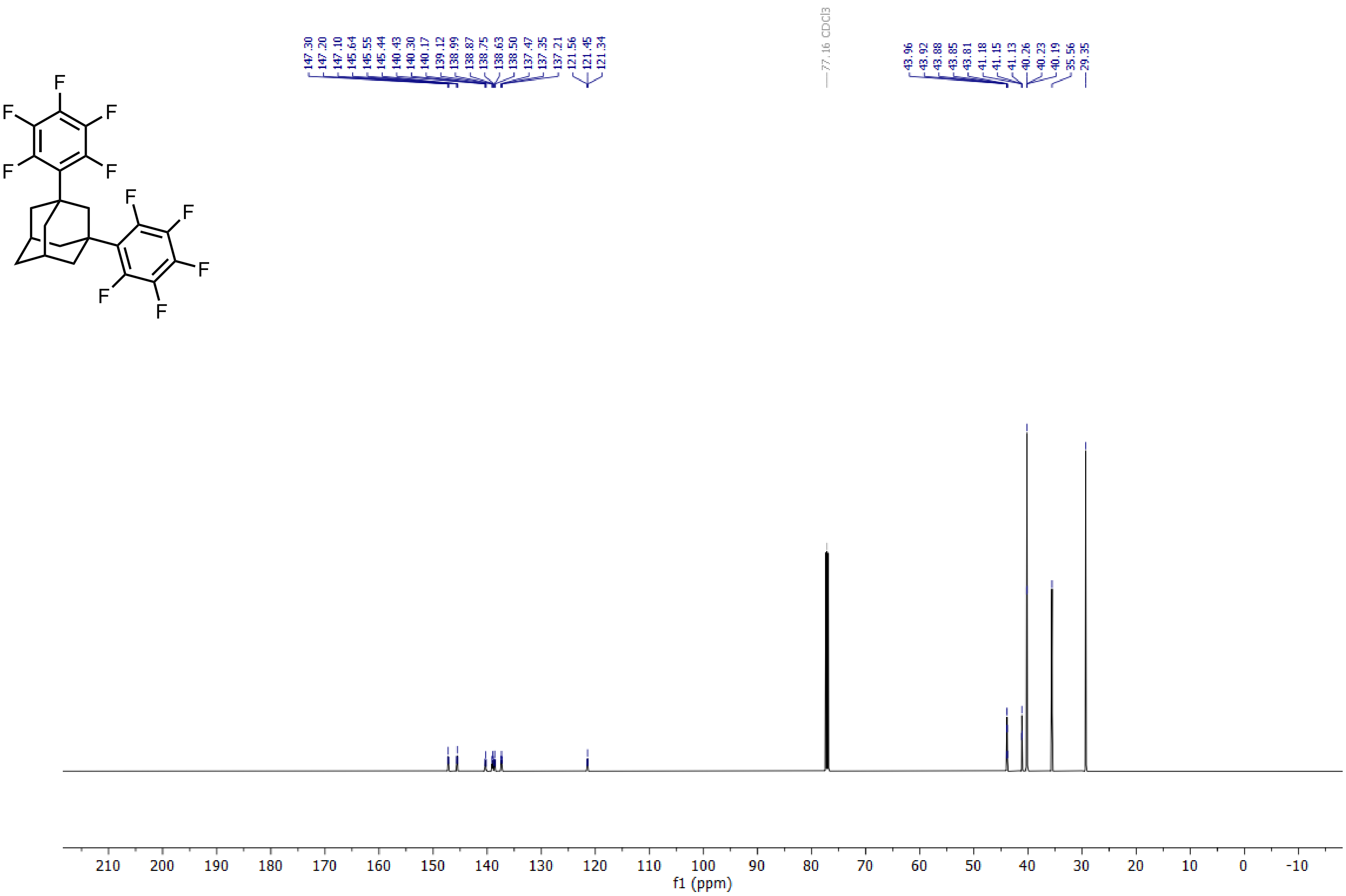

^{19}F NMR of 1,3-Bis(perfluorophenyl)adamantane (16)

$CDCl_3$, 23 ºC

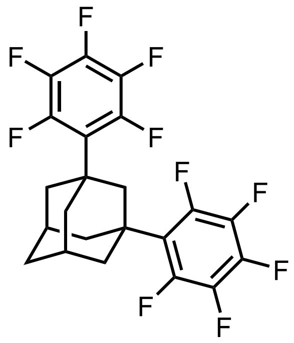

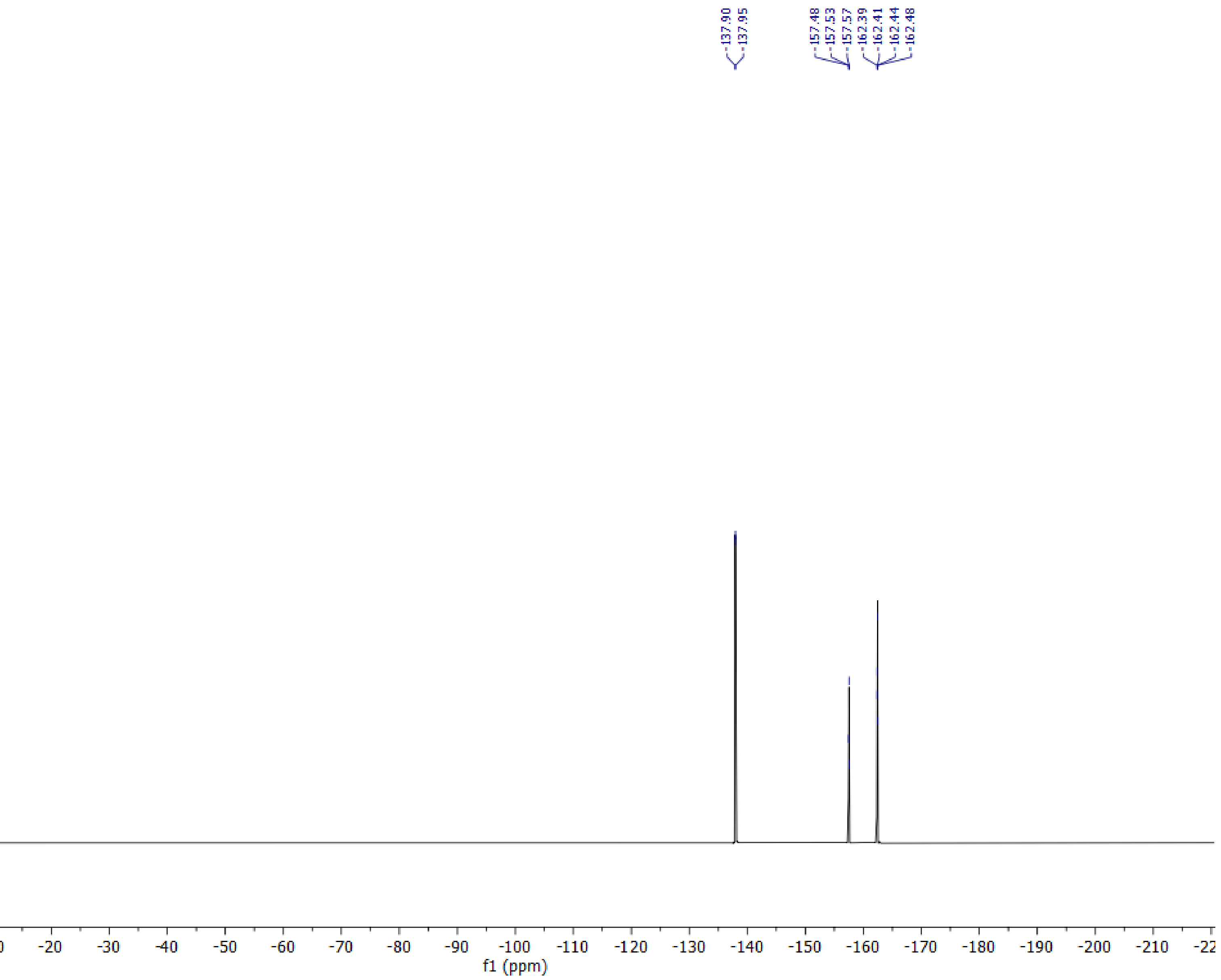

^{1}H NMR of Ciprofibrate derivative 17

$CDCl_3$, 23 ºC

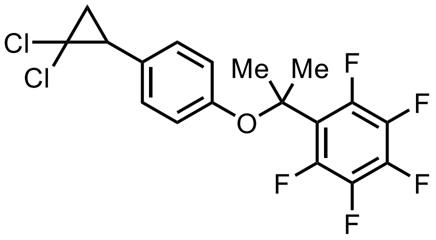

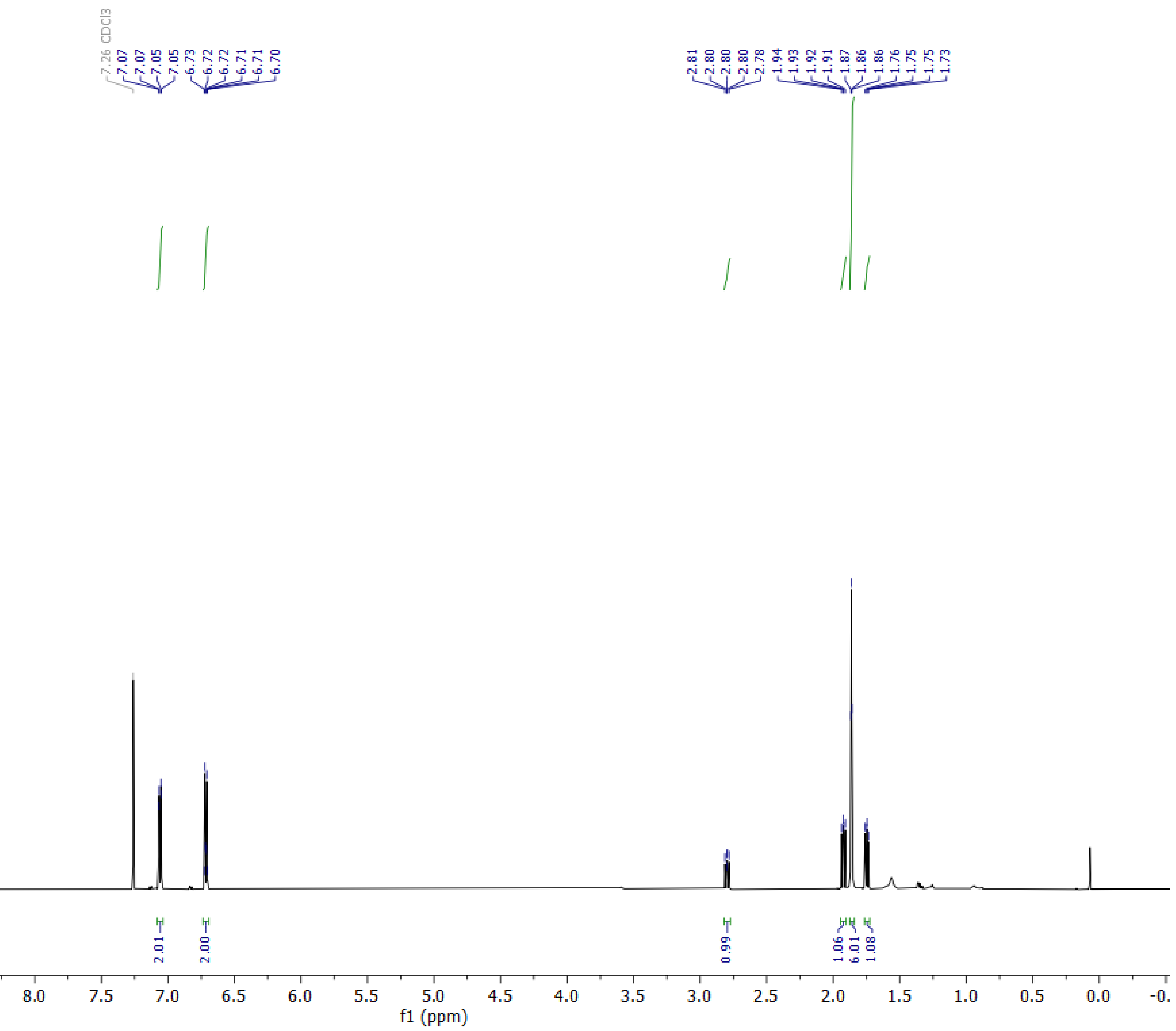

^{13}C NMR of Ciprofibrate derivative 17

$CDCl_3$, 23 ºC

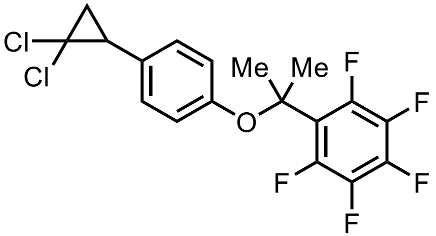

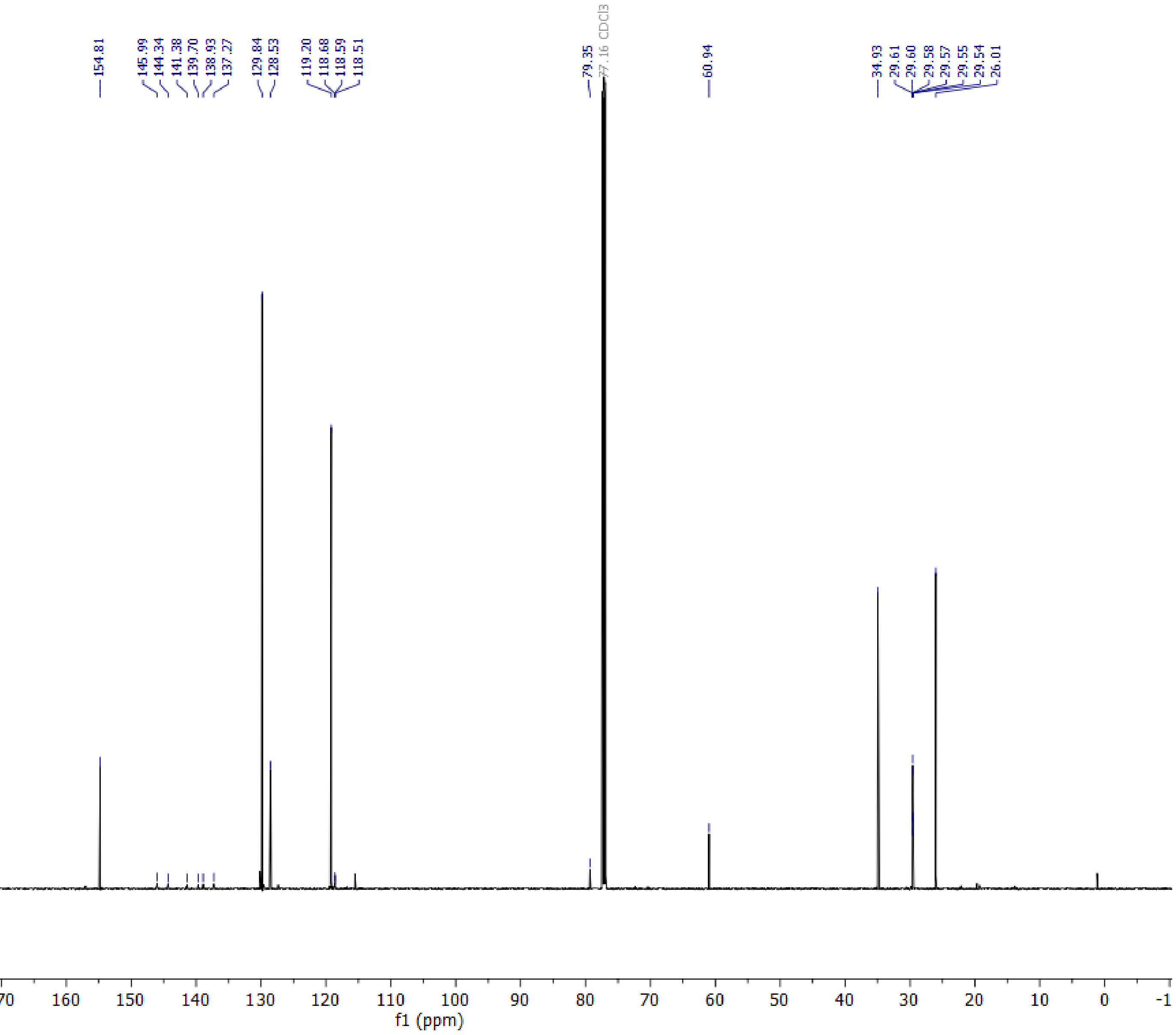

^{19}F NMR of Ciprofibrate derivative 17

$CDCl_3$, 23 ºC

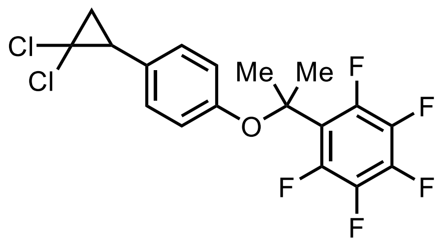

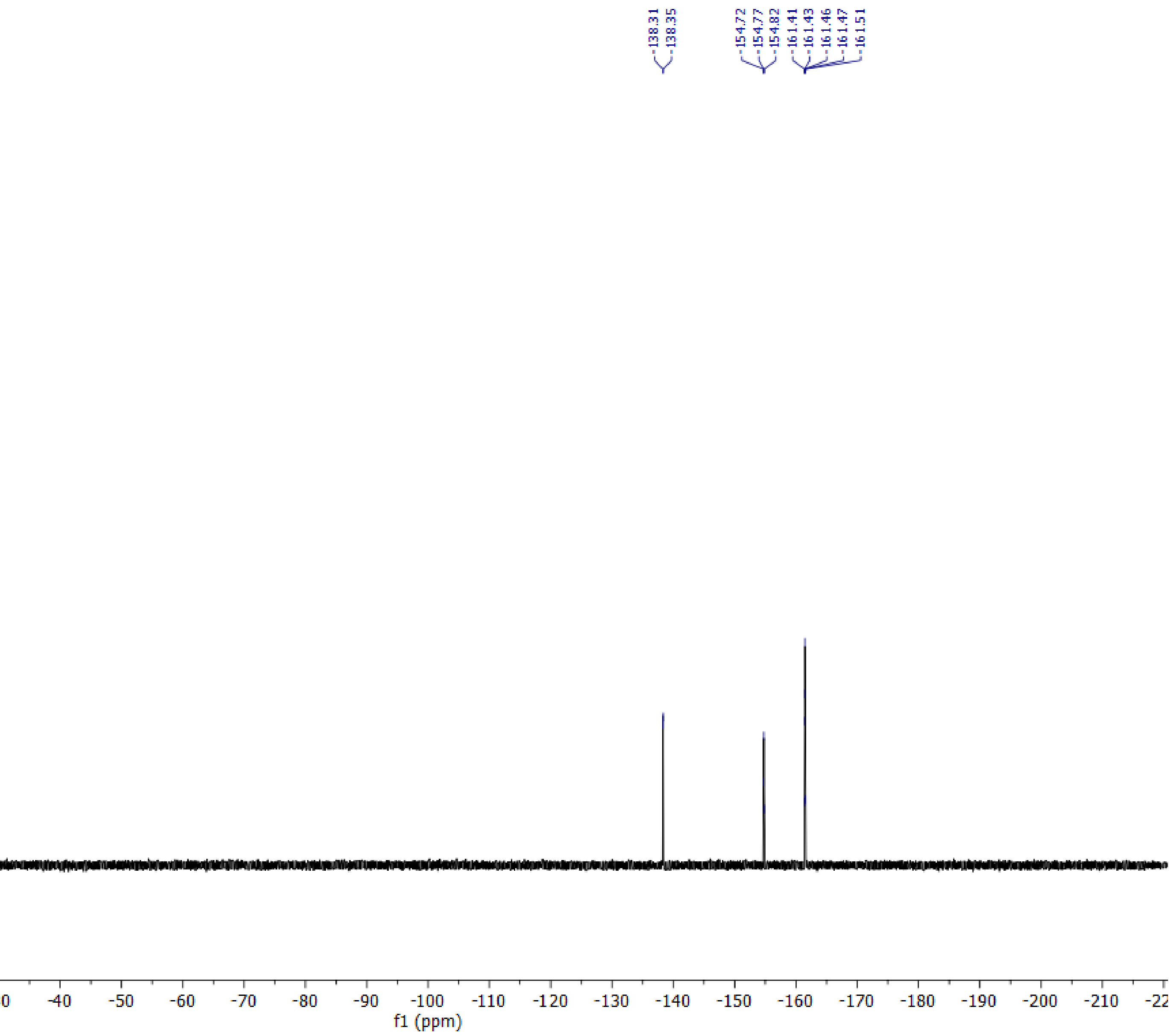

^{1}H NMR of (2S,3R)-2-(Methylcarboxyl)-3-pentafluorophenylnorbornan (18)

$CDCl_3$, 23 ºC

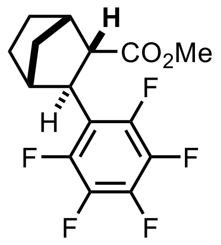

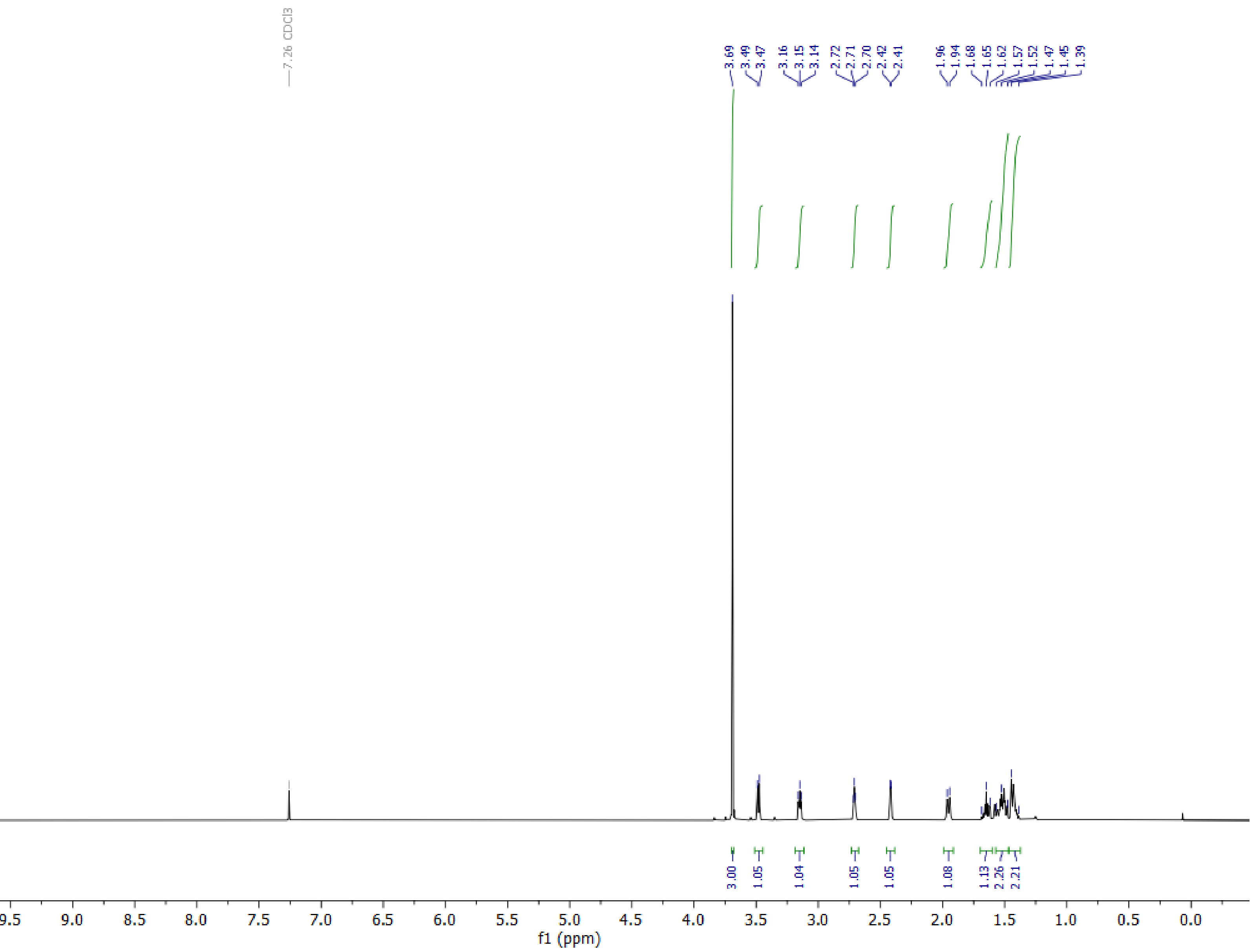

^{13}C NMR of (2S,3R)-2-(Methylcarboxyl)-3-pentafluorophenylnorbornan (18)

$CDCl_3$, 23 ºC

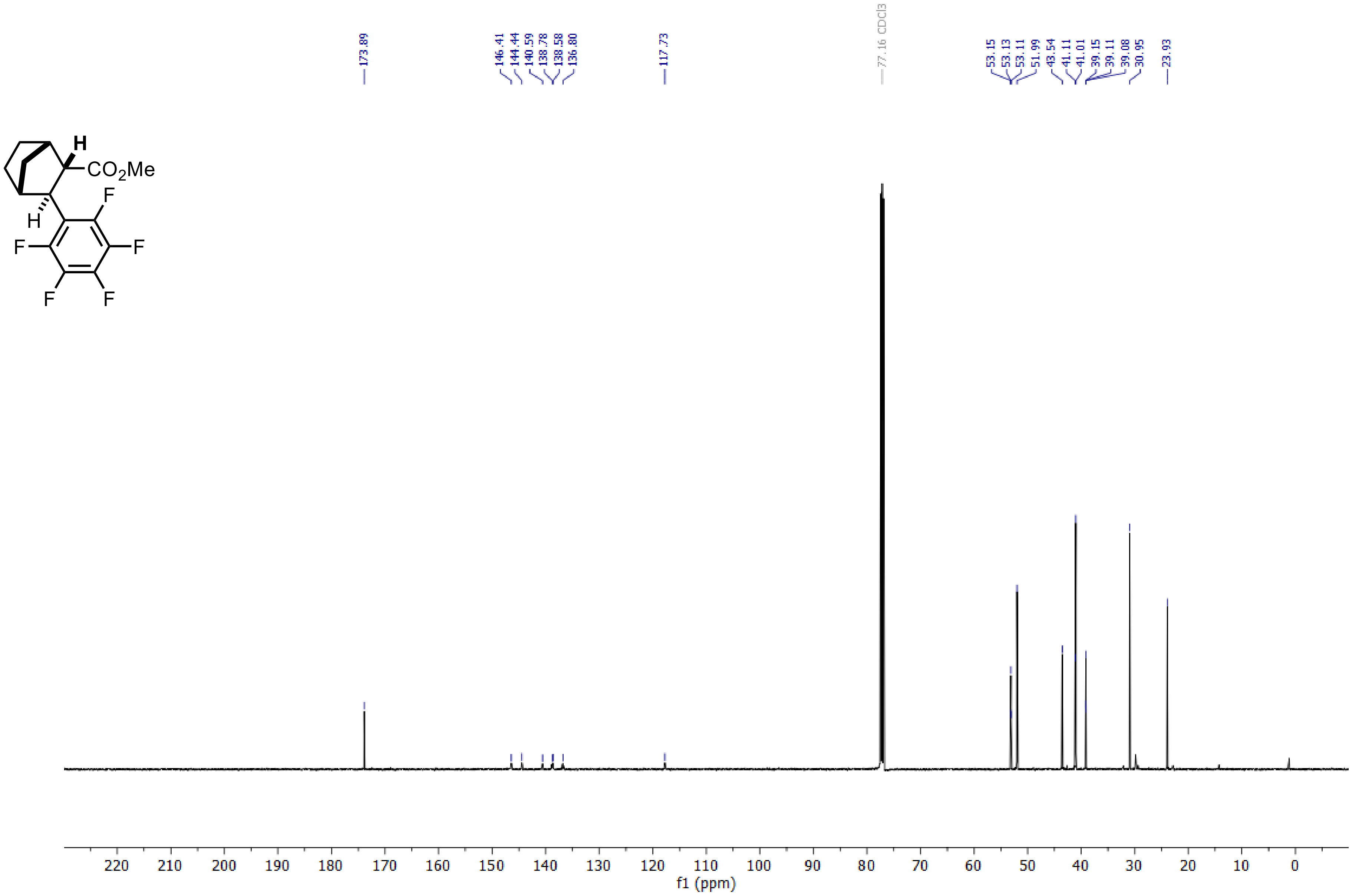

^{19}F NMR of (2S,3R)-2-(Methylcarboxyl)-3-pentafluorophenylnorbornan (18)

$CDCl_3$, 23 ºC

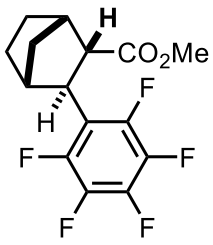

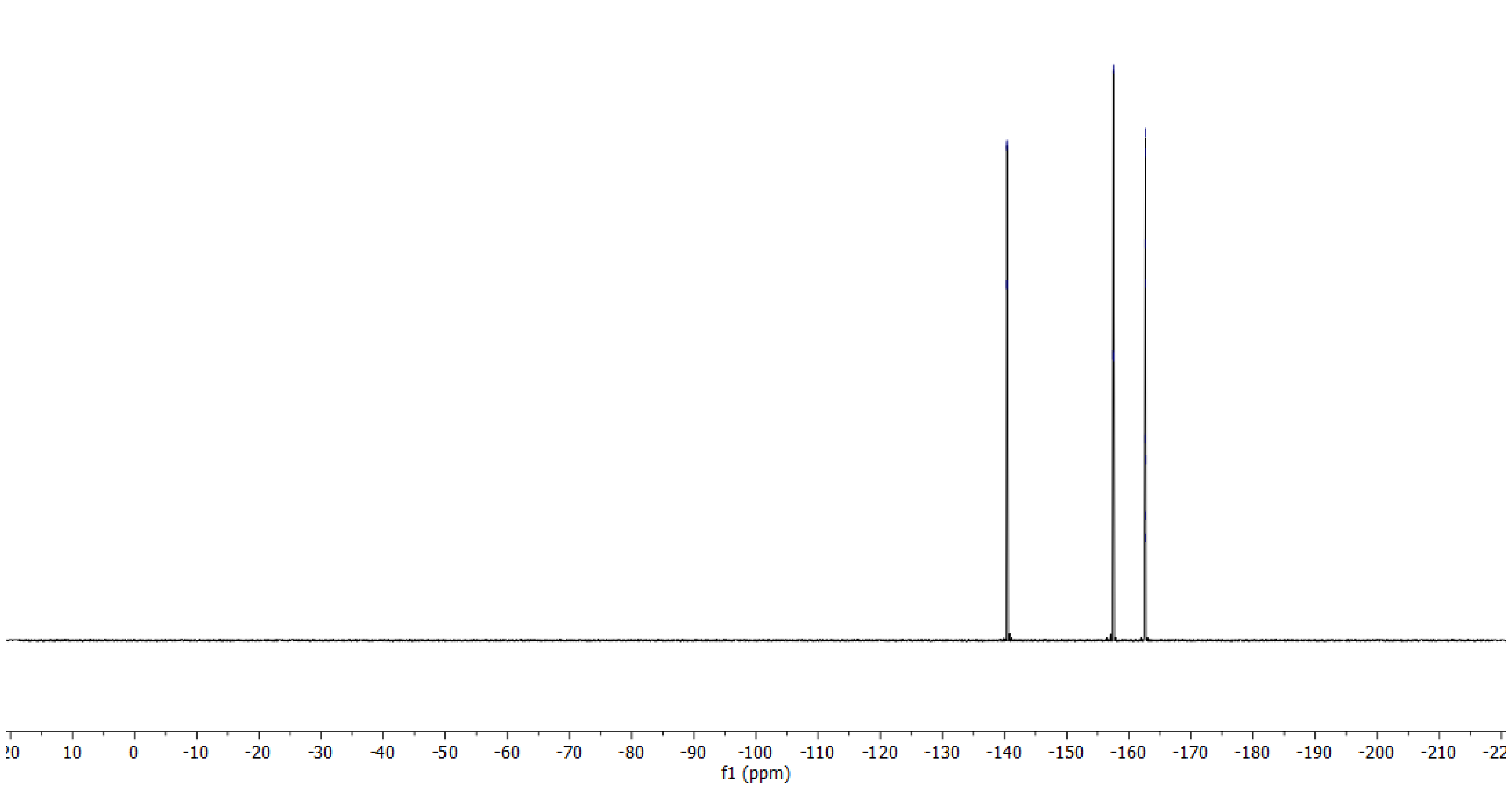

H-H-NOESY spectrum of (2S,3R)-2-(Methylcarboxyl)-3-pentafluorophenylnorbornan (18)

$CDCl_3$, 23 ºC

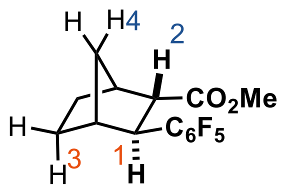

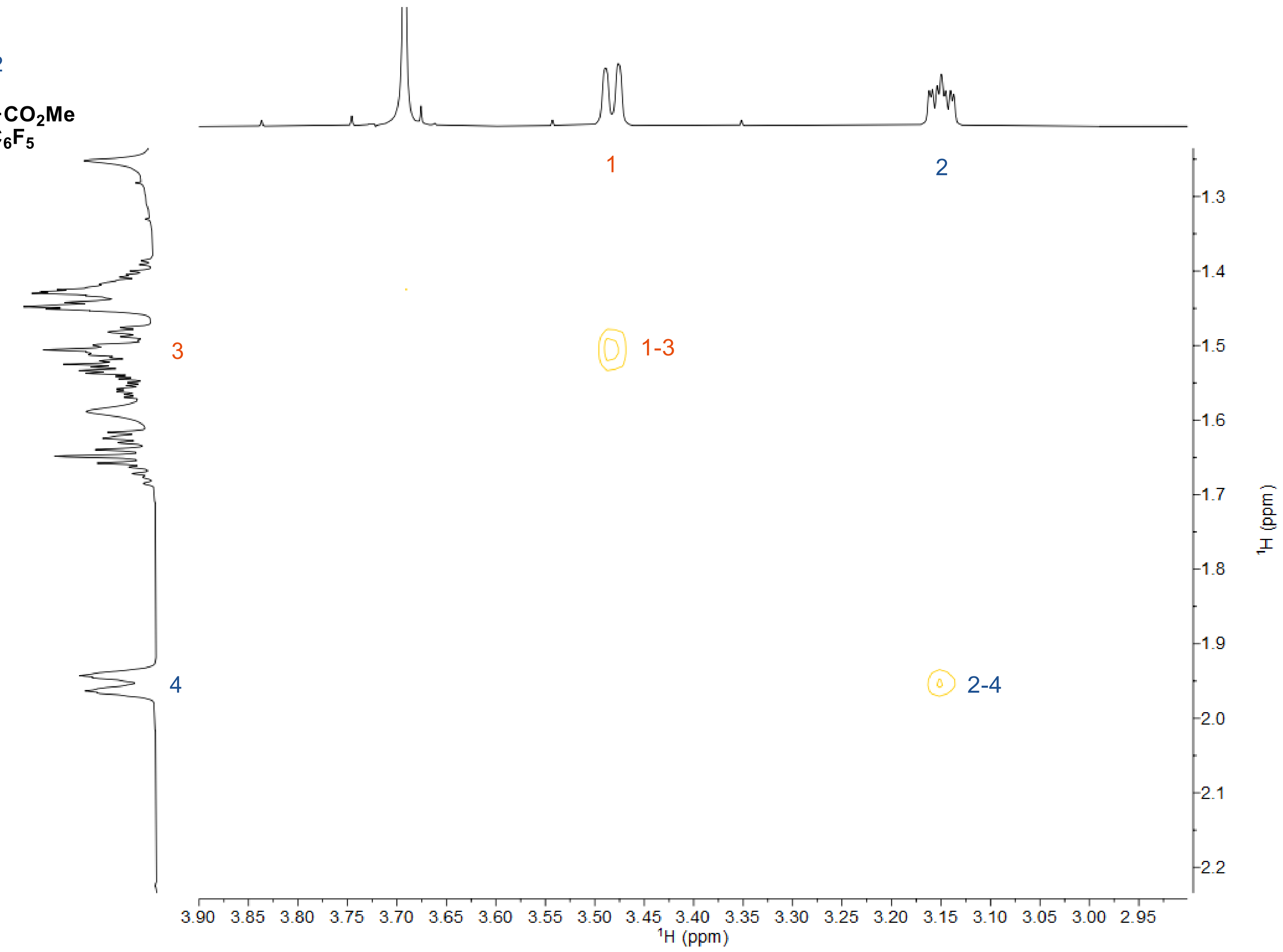

H-F-NOESY spectrum of (2S,3R)-2-(Methylcarboxyl)-3-pentafluorophenylnorbornan (18)

CDCl3, 23 ºC

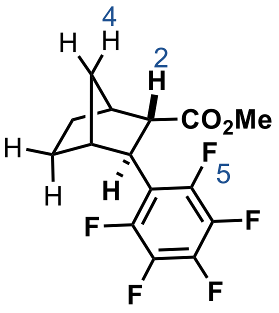

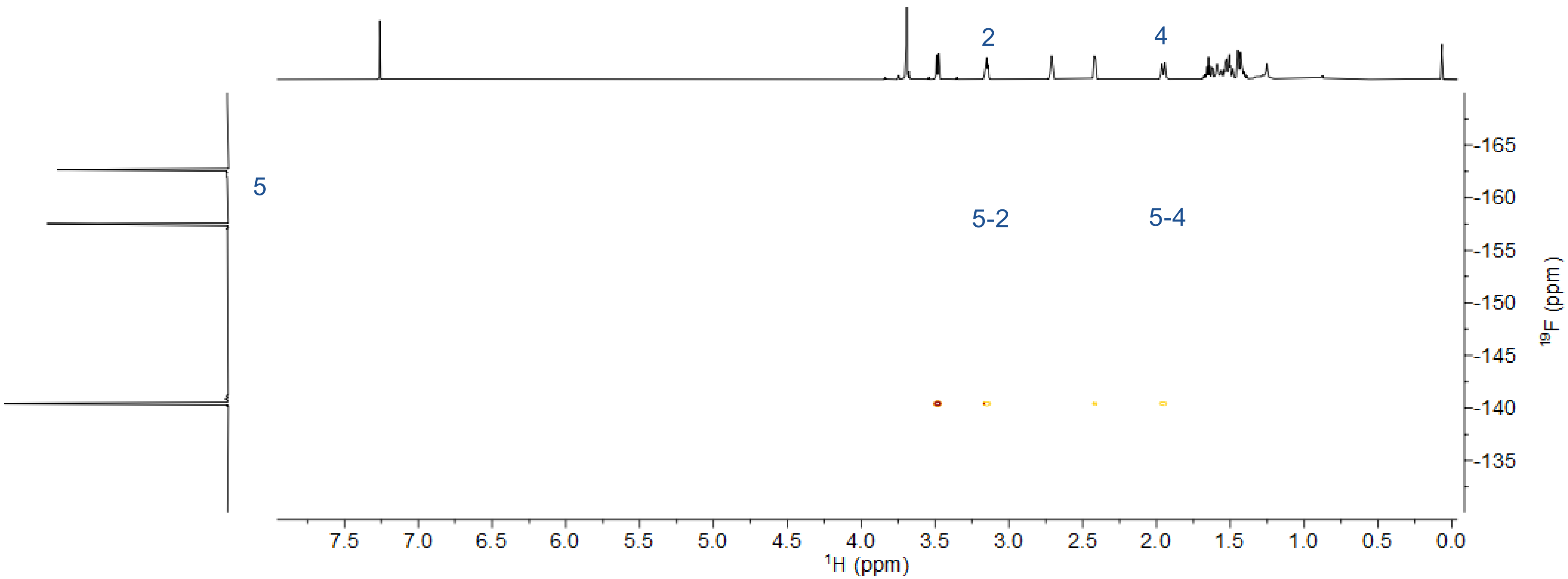

^{1}H NMR of (4S,5S)-*N*-Boc-2,2-dimethyl-4-phenyl-5-pentafluorophenyloxazolidine (19)

DMSO-d_6, 80 ºC

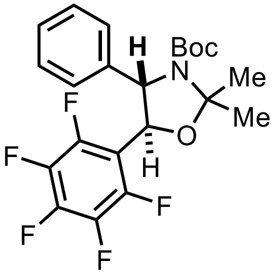

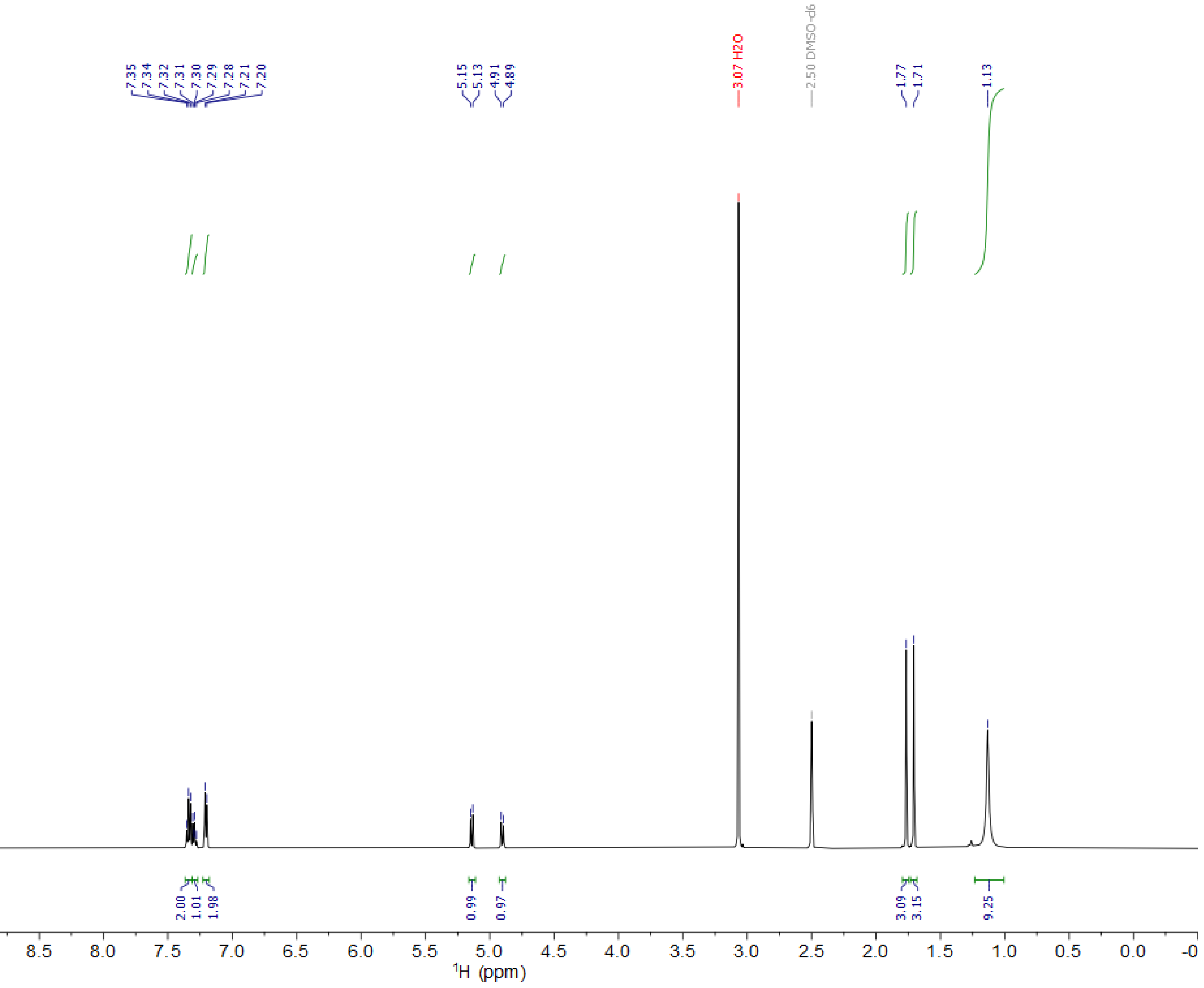

^{13}C NMR of (4S,5S)-*N*-Boc-2,2-dimethyl-4-phenyl-5-pentafluorophenyloxazolidine (19)

DMSO-d_6, 80 ºC

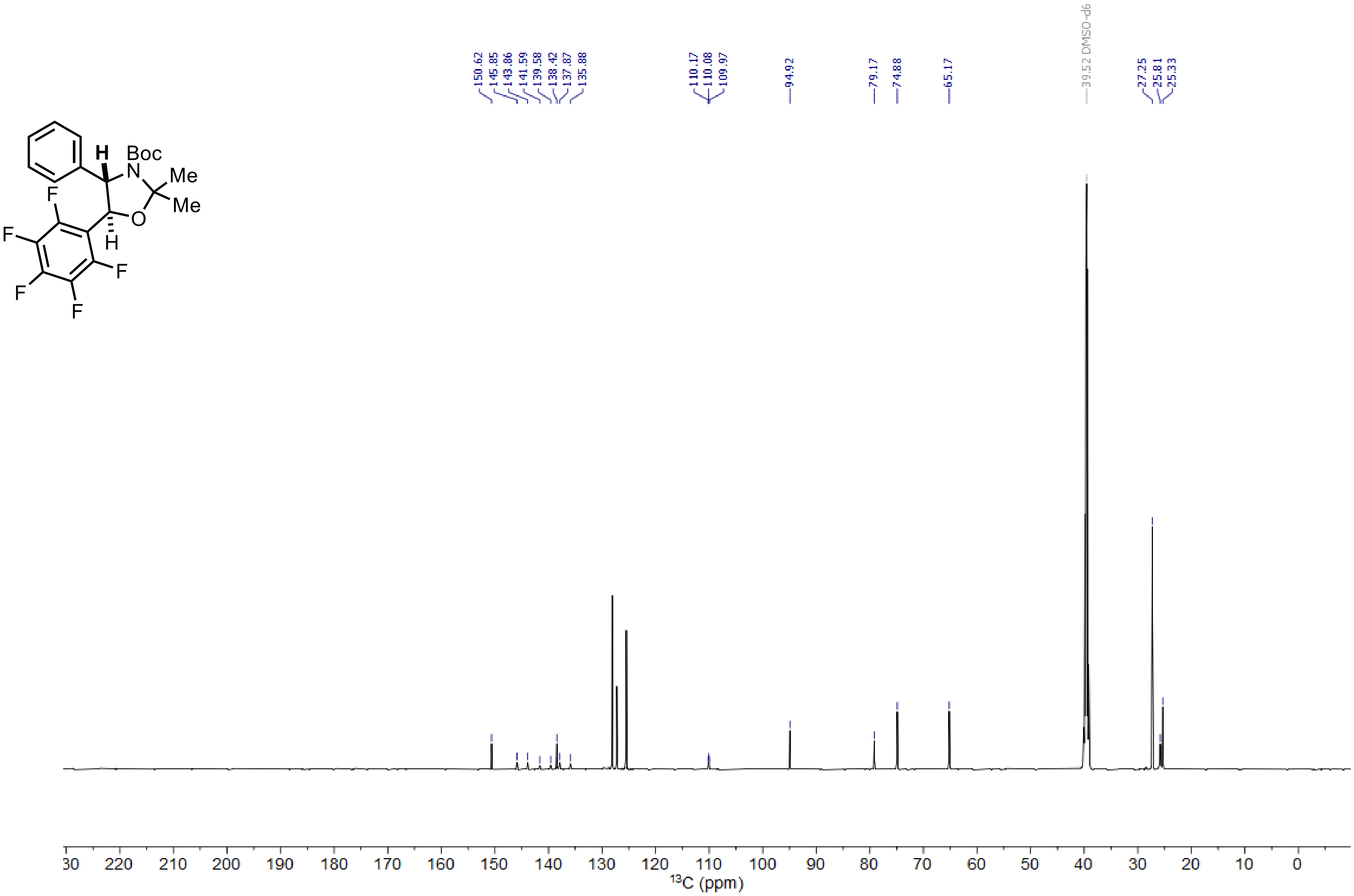

^{19}F NMR of (4S,5S)-*N*-Boc-2,2-dimethyl-4-phenyl-5-pentafluorophenyloxazolidine (19)

DMSO-d_6, 80 ºC

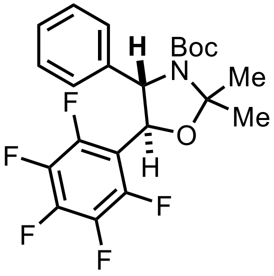

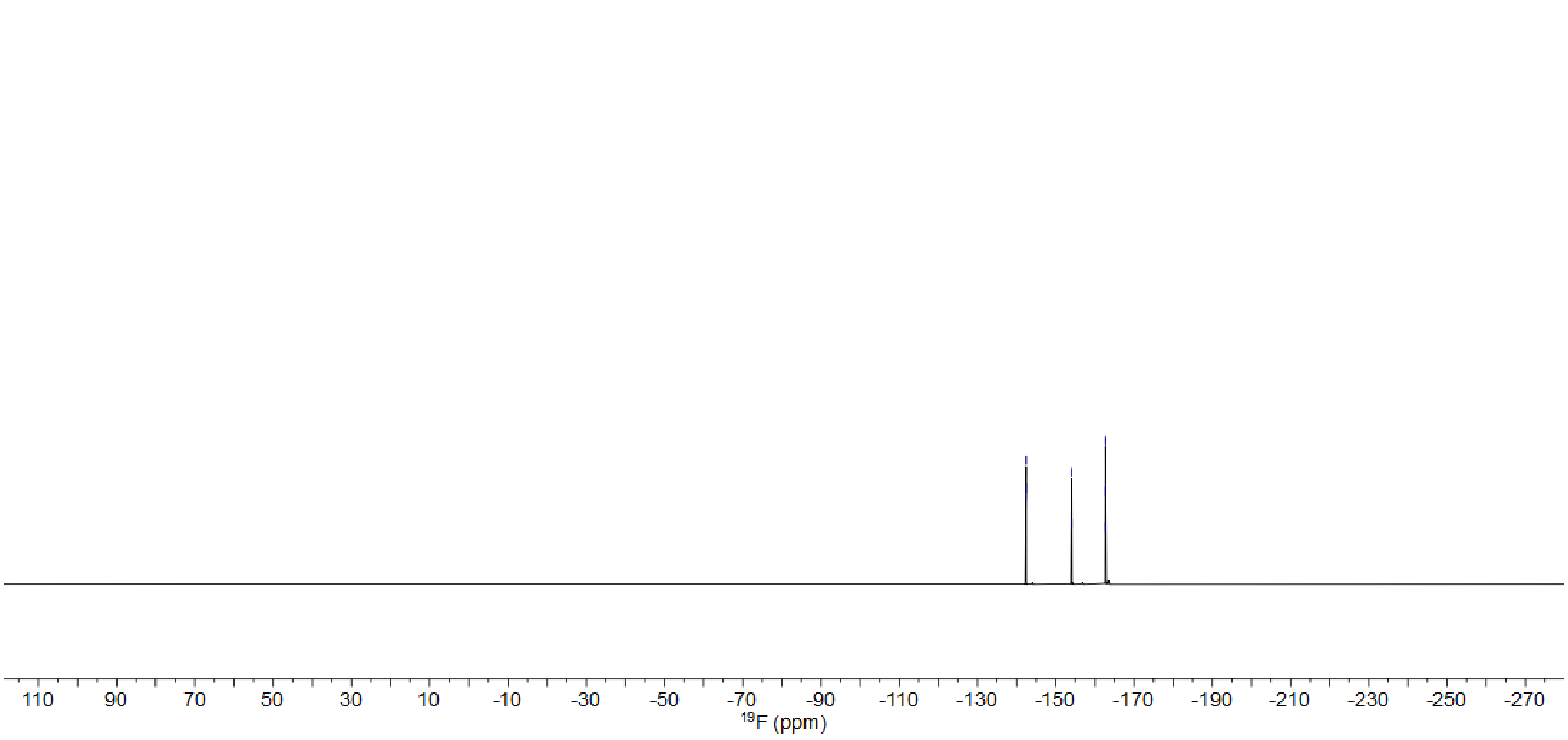

H-H-NOESY spectrum of (4S,5S)-*N*-Boc-2,2-dimethyl-4-phenyl-5-pentafluorophenyloxazolidine (19)

DMSO-d_6, 80 ºC

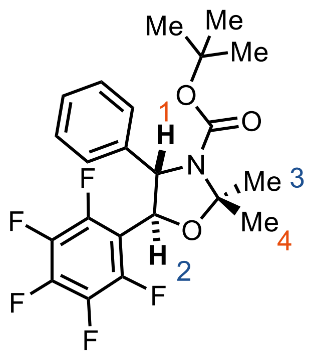

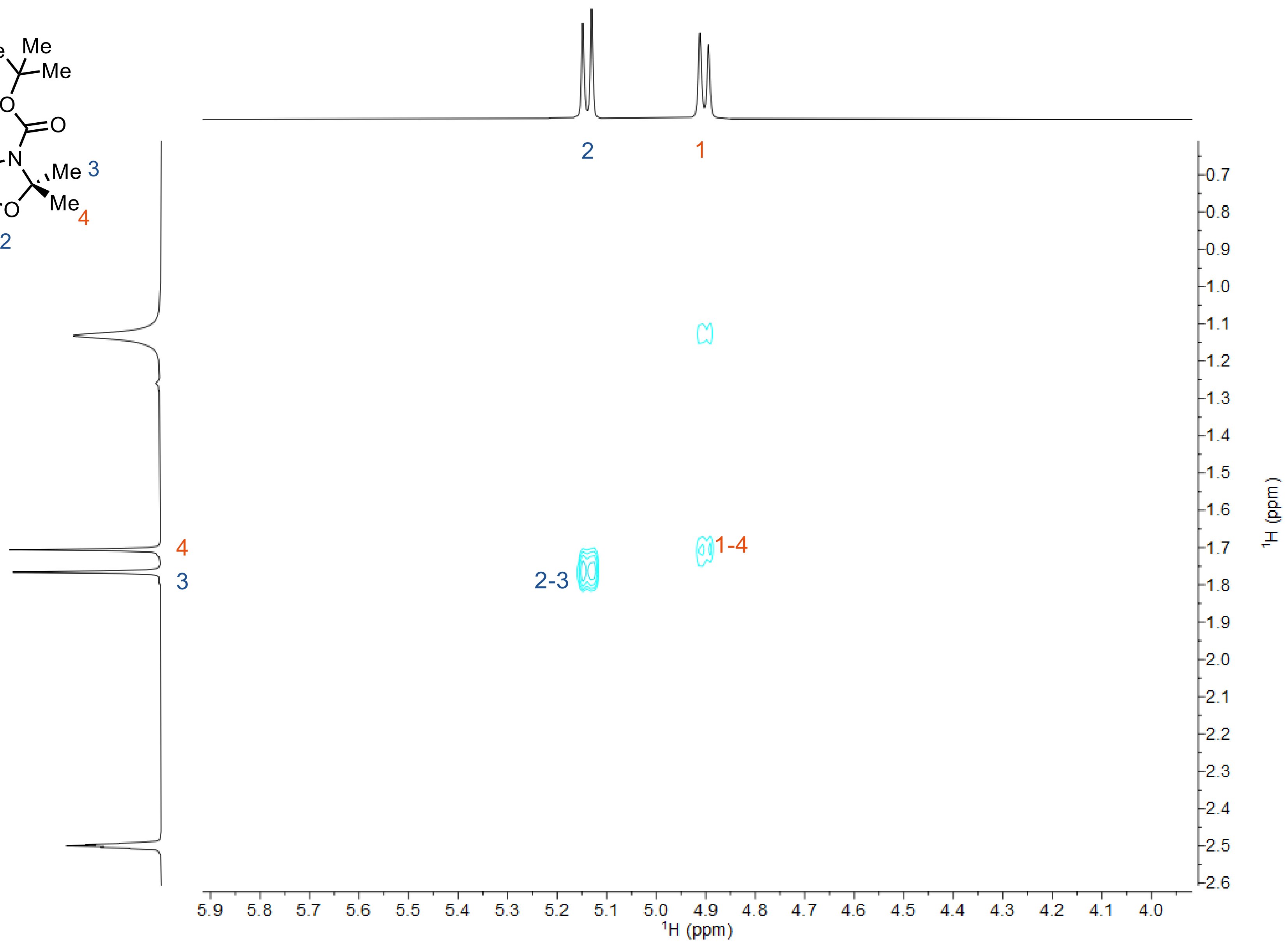

^{1}H NMR of Estrone derivative 20

$CDCl_3$, 23 ºC

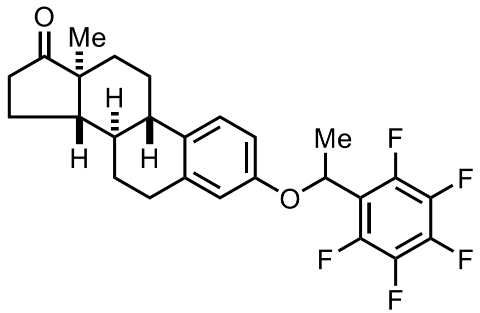

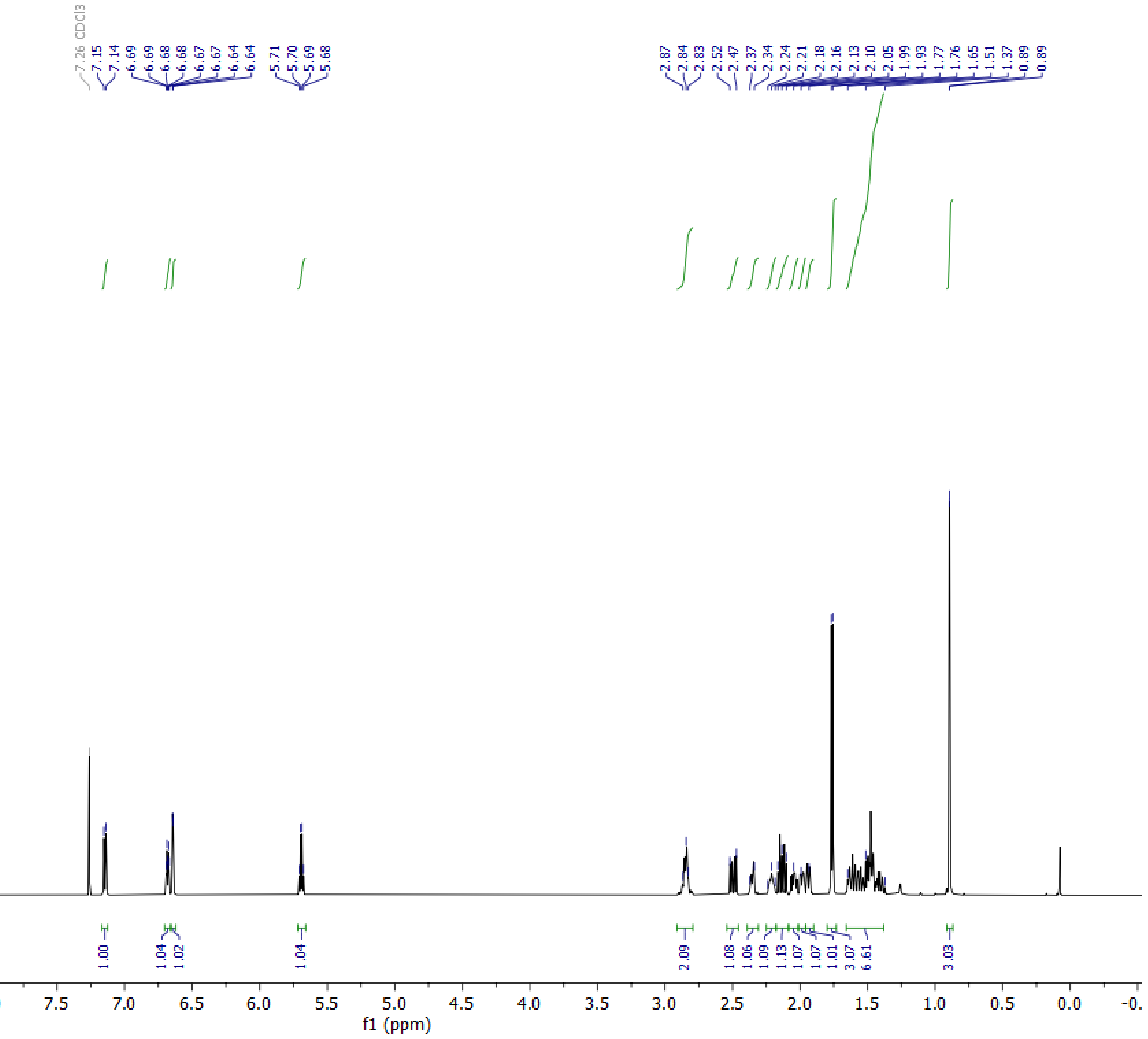

^{13}C NMR of Estrone derivative 20

$CDCl_3$, 23 ºC

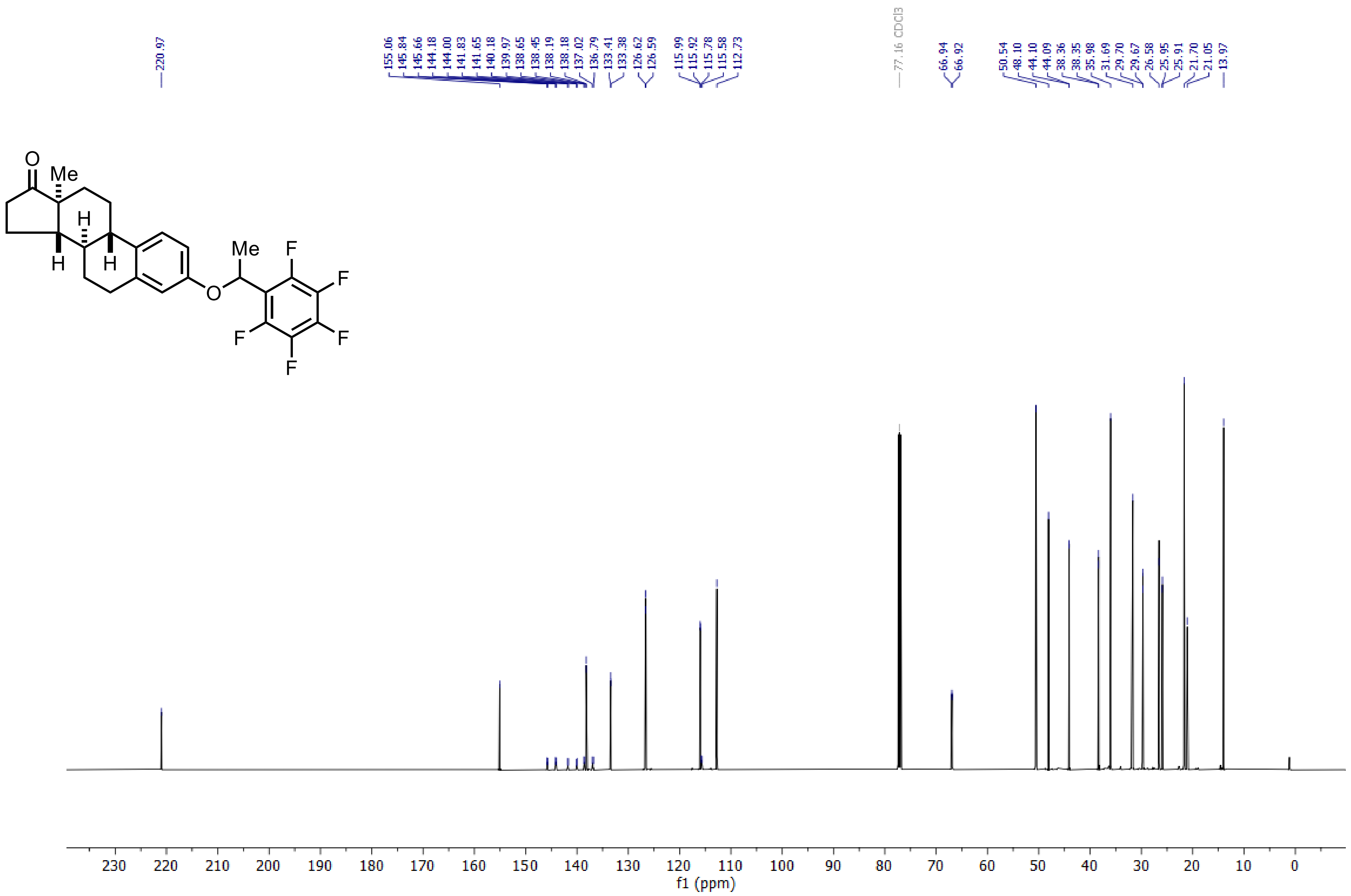

^{19}F NMR of Estrone derivative 20

$CDCl_3$, 23 ºC

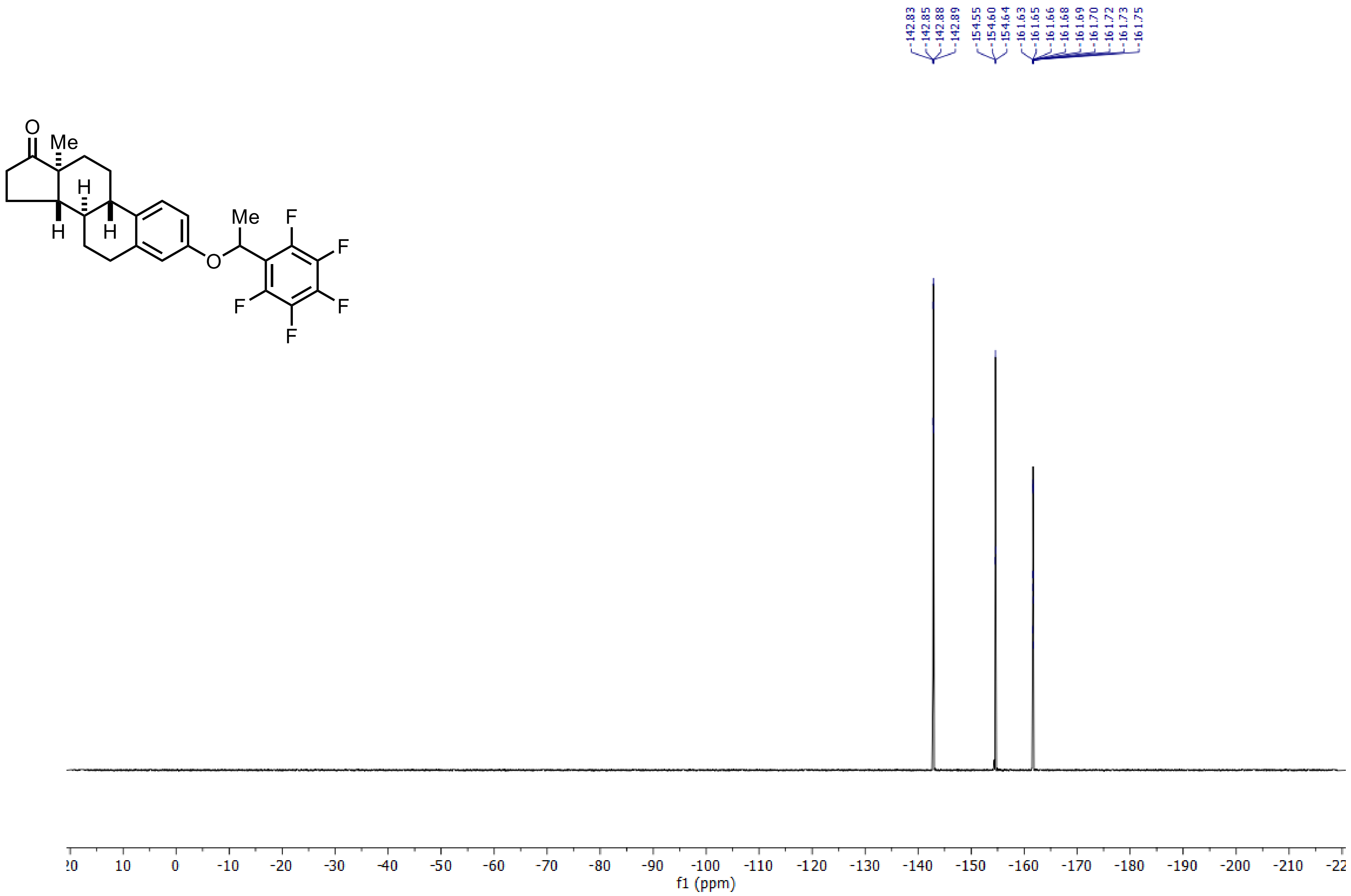

^{1}H NMR of Glycyrrhetic acid derivative 21

$CDCl_3$, 23 ºC

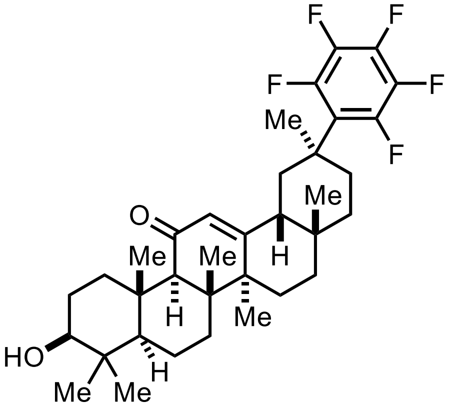

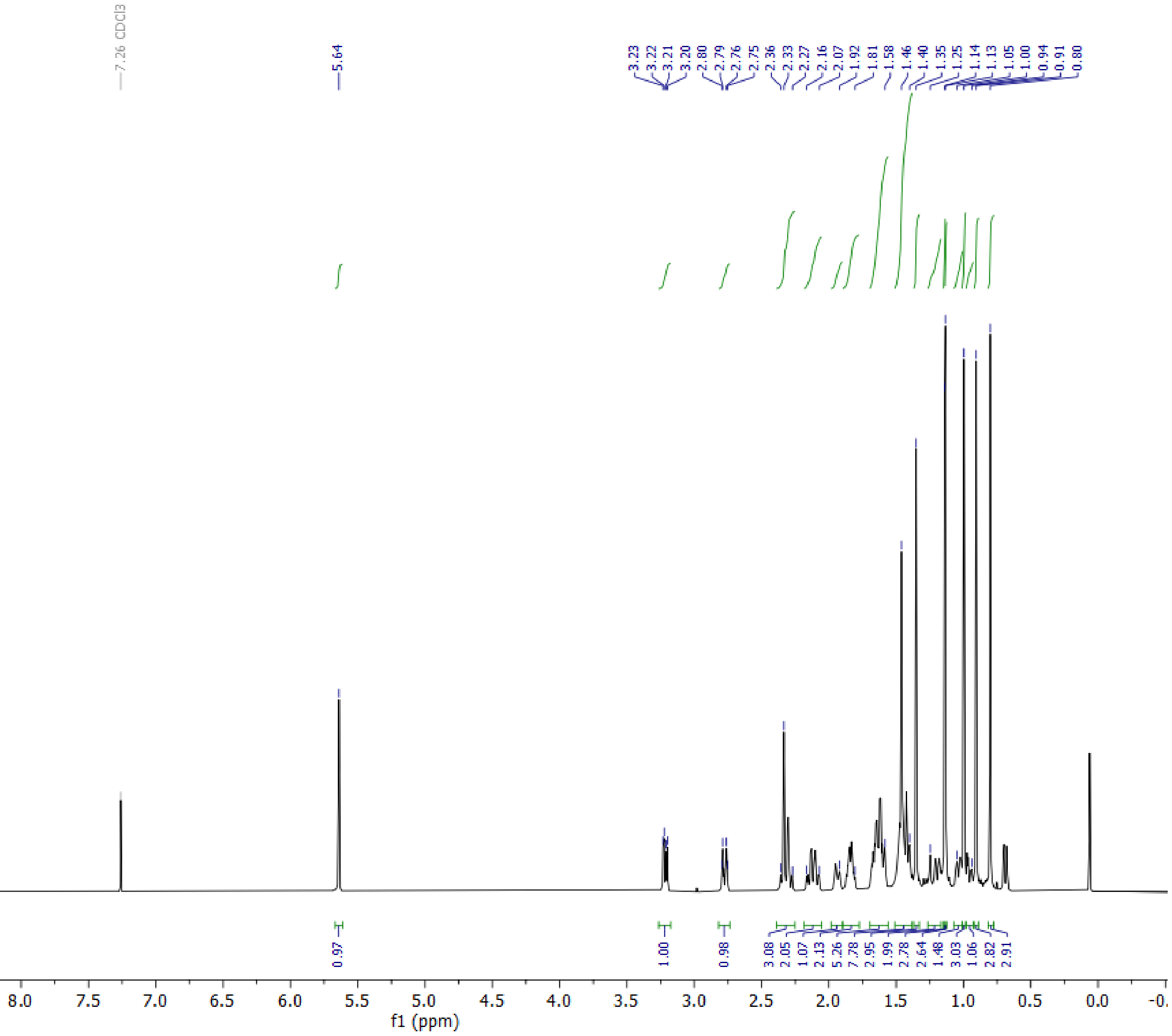

^{13}C NMR of Glycyrrhetic acid derivative 21

CDCl₃, 23 ºC

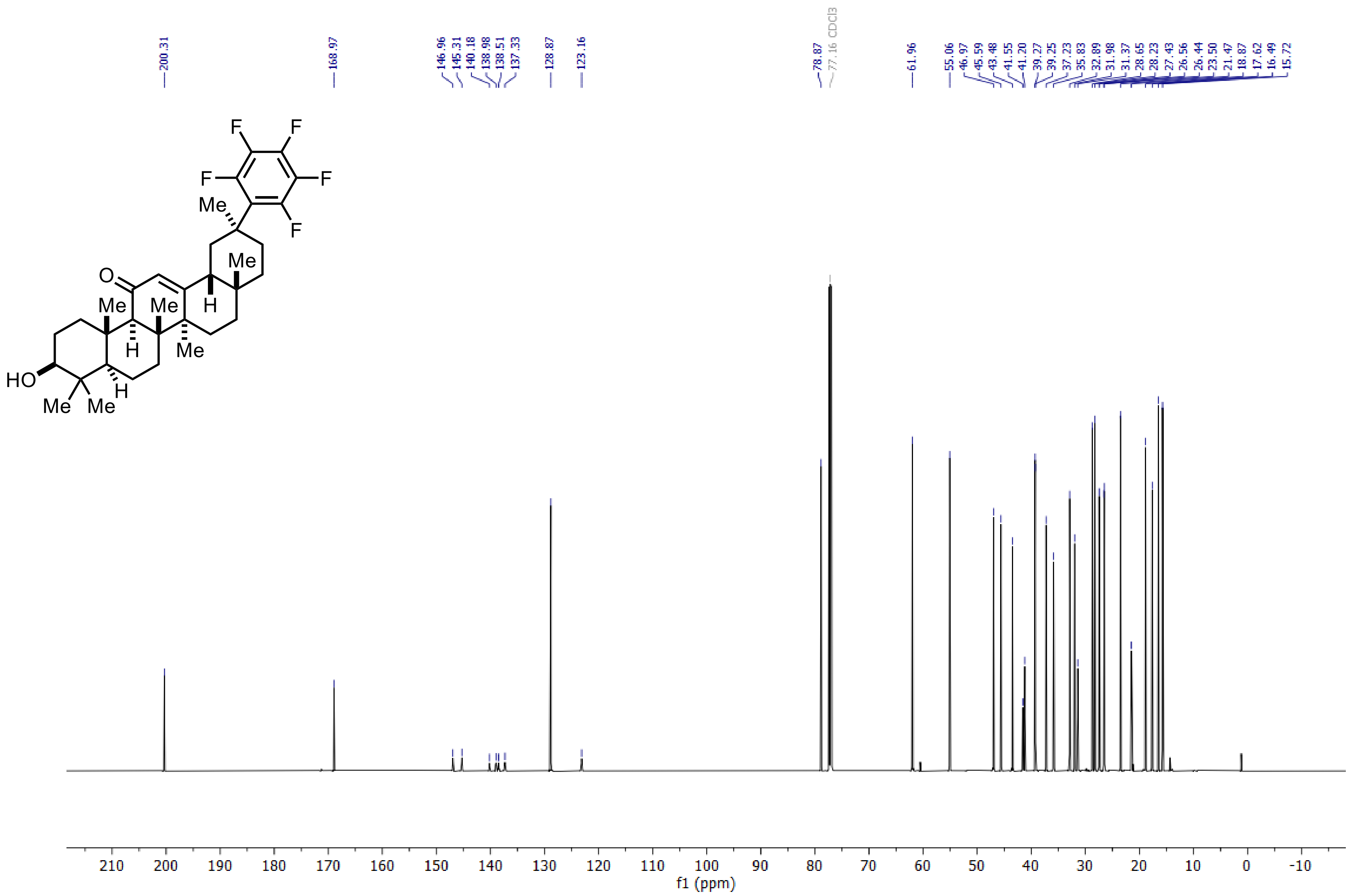

^{19}F NMR of Glycyrrhetic acid derivative 21

CDCl$_3$, 23 ºC

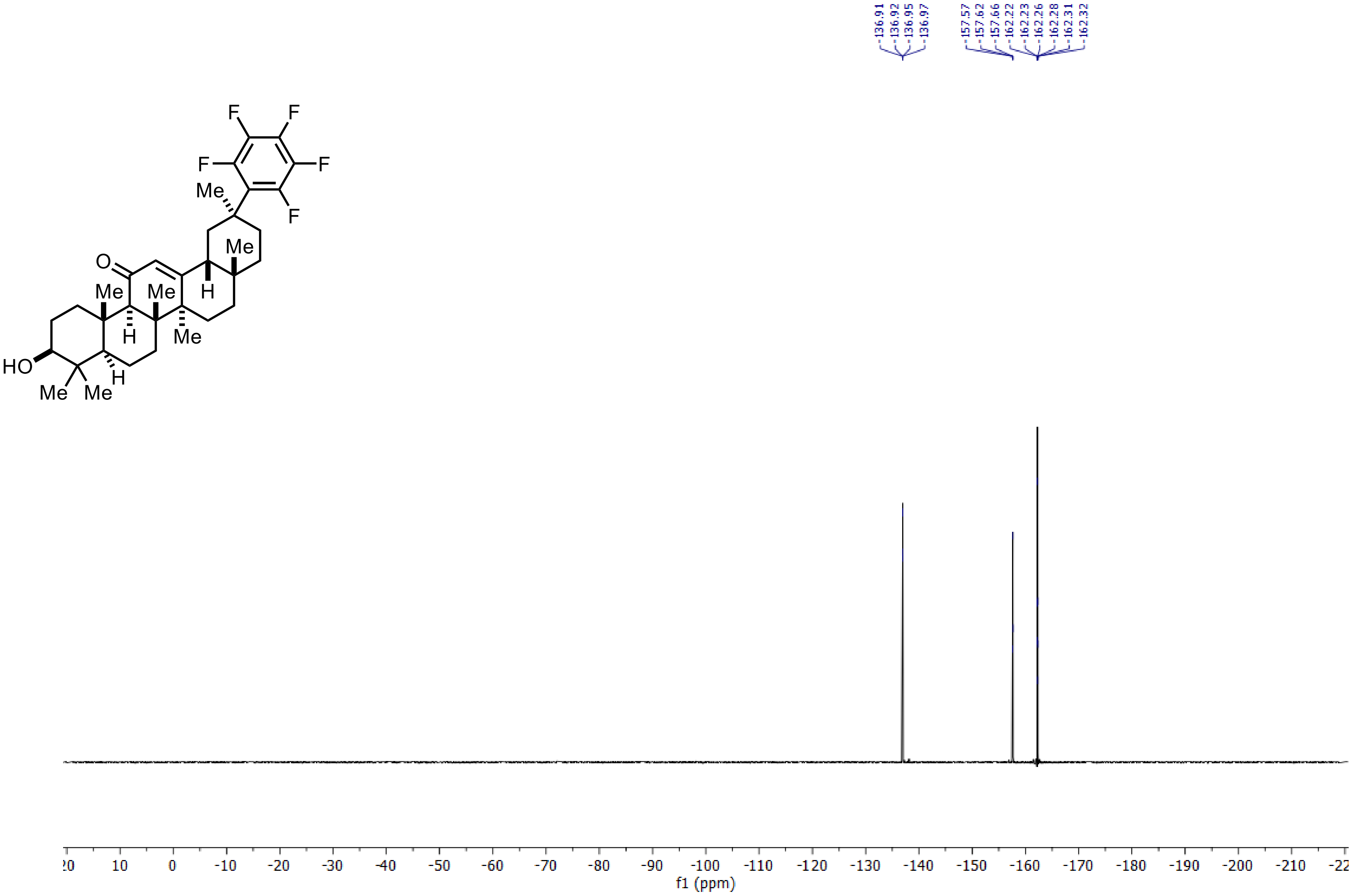

H-H-NOESY spectrum of Glycyrrhetic acid derivative 21

$CDCl_3$, 23 ºC

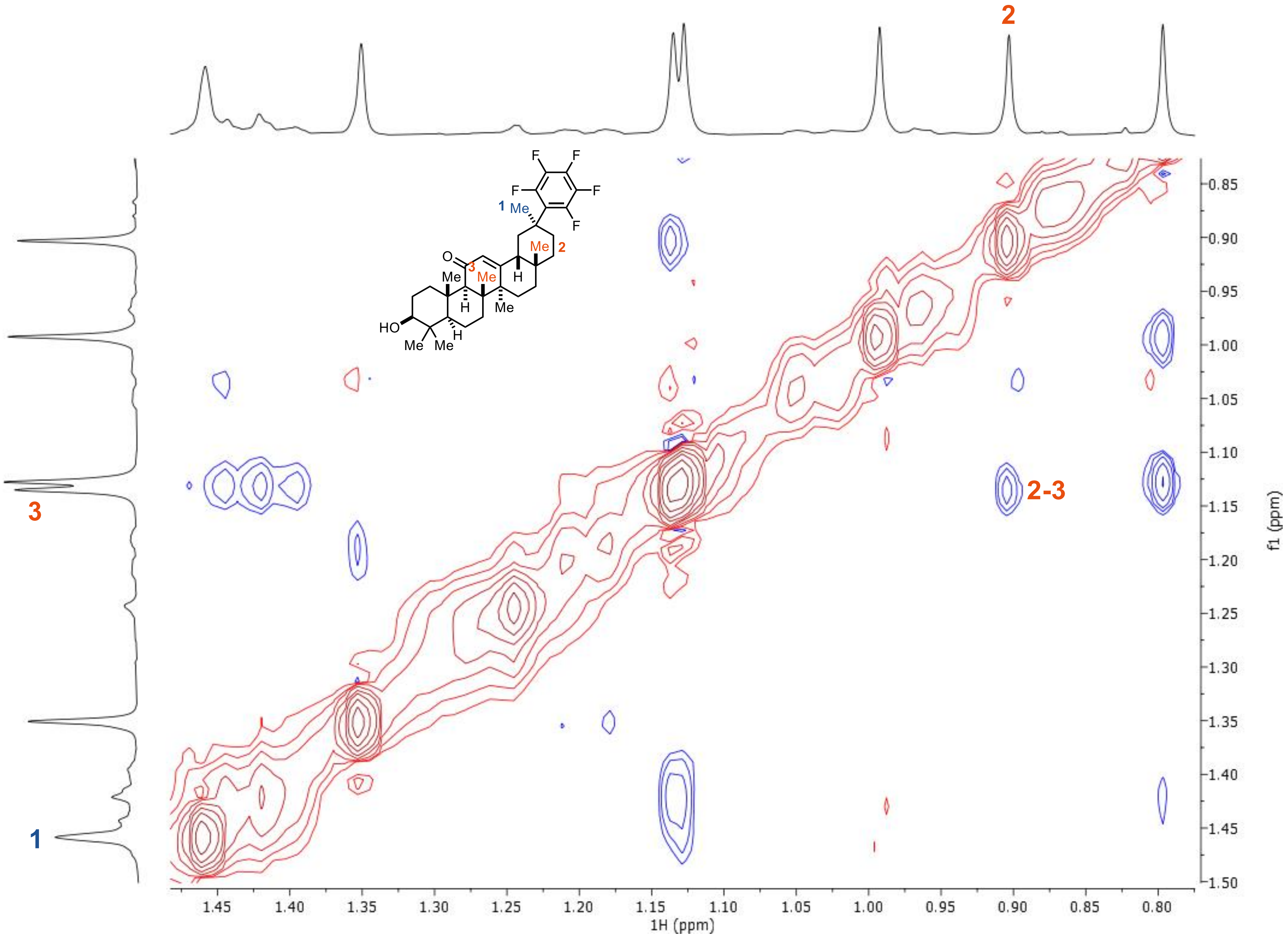

^{1}H NMR of *N*-Boc-pentafluorobenzylamine (22)

$CDCl_3$, 23 ºC

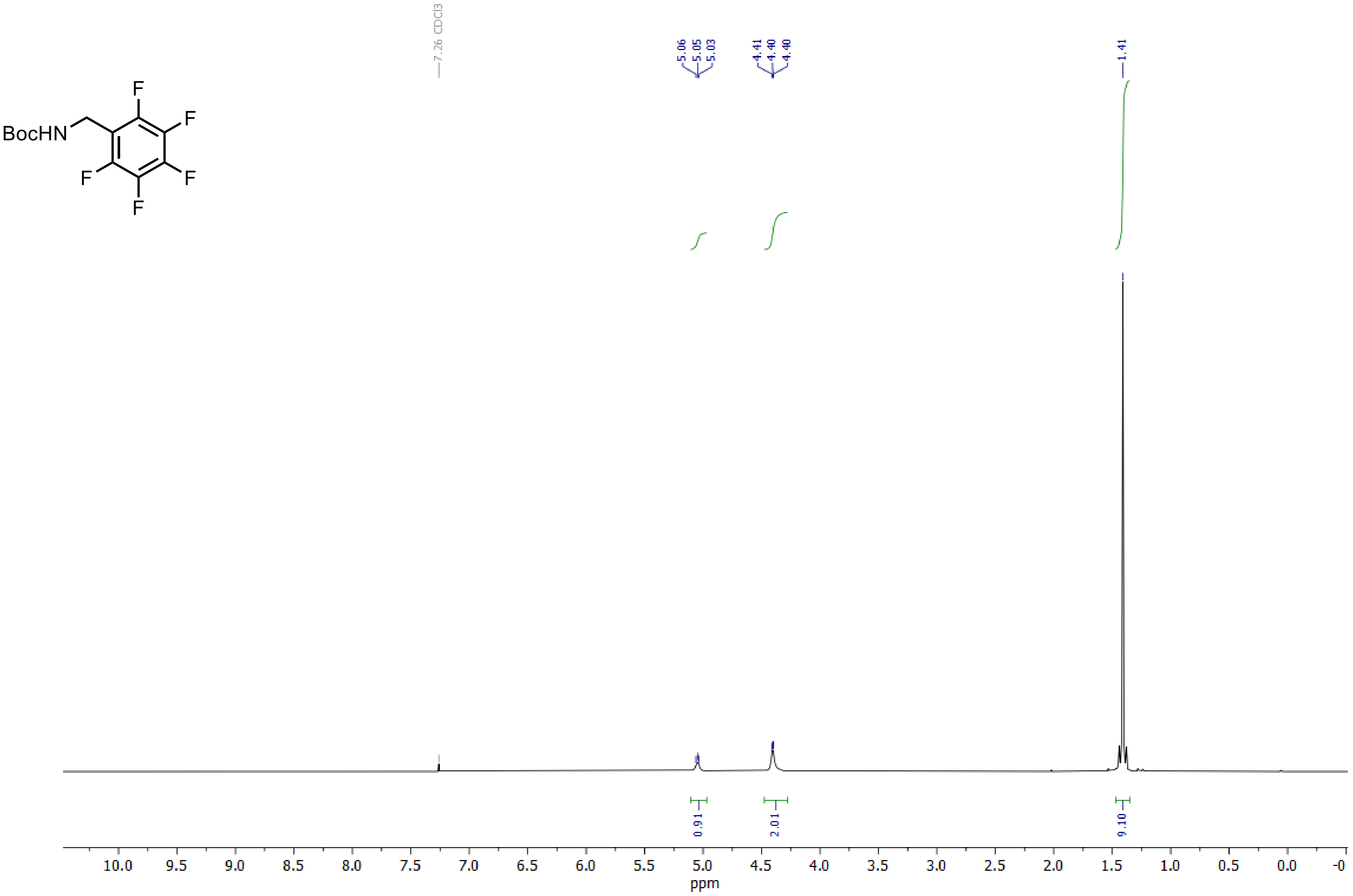

^{13}C NMR of *N*-Boc-pentafluorobenzylamine (22)

$CDCl_3$, 23 ºC

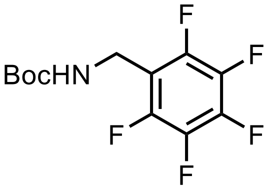

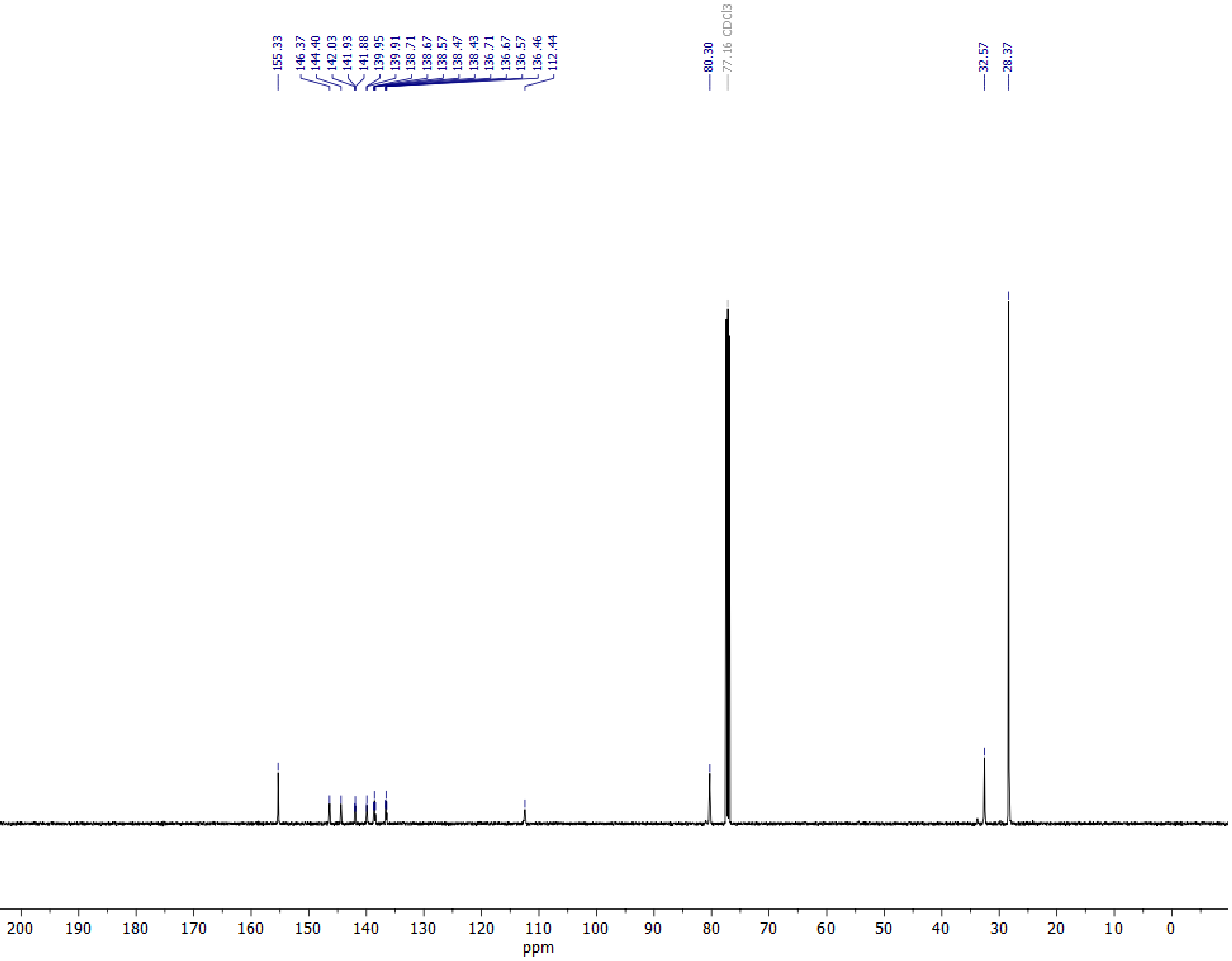

^{19}F NMR of *N*-Boc-pentafluorobenzylamine (22)

$CDCl_3$, 23 ºC

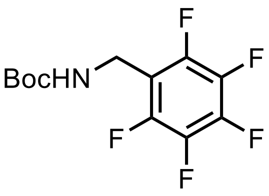

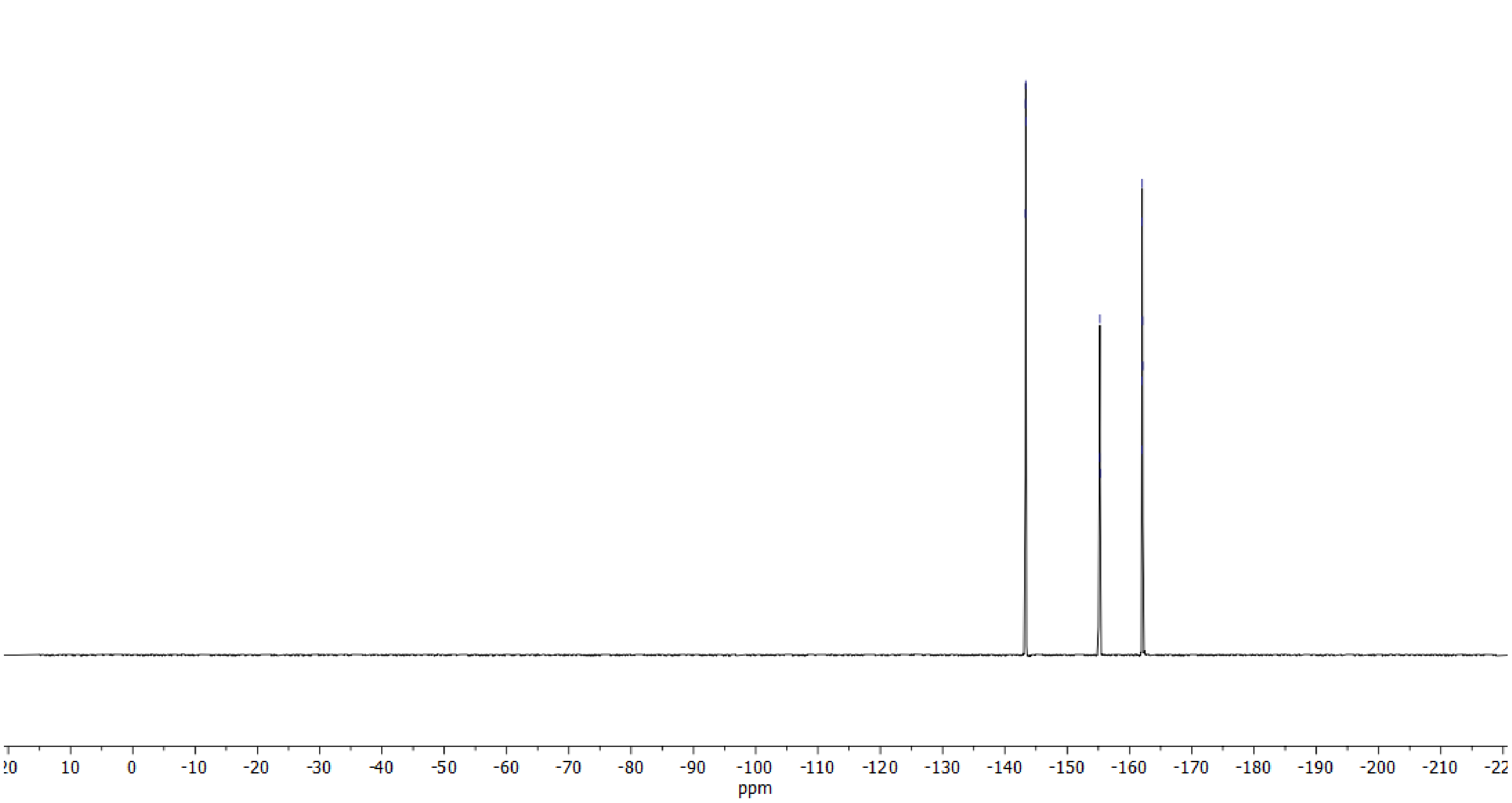

^{1}H NMR of *N*-Boc-4-chlorotetrafluorobenzylamine (23a)

$CDCl_3$, 23 ºC

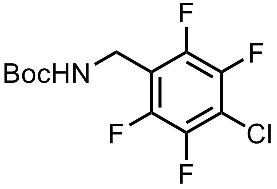

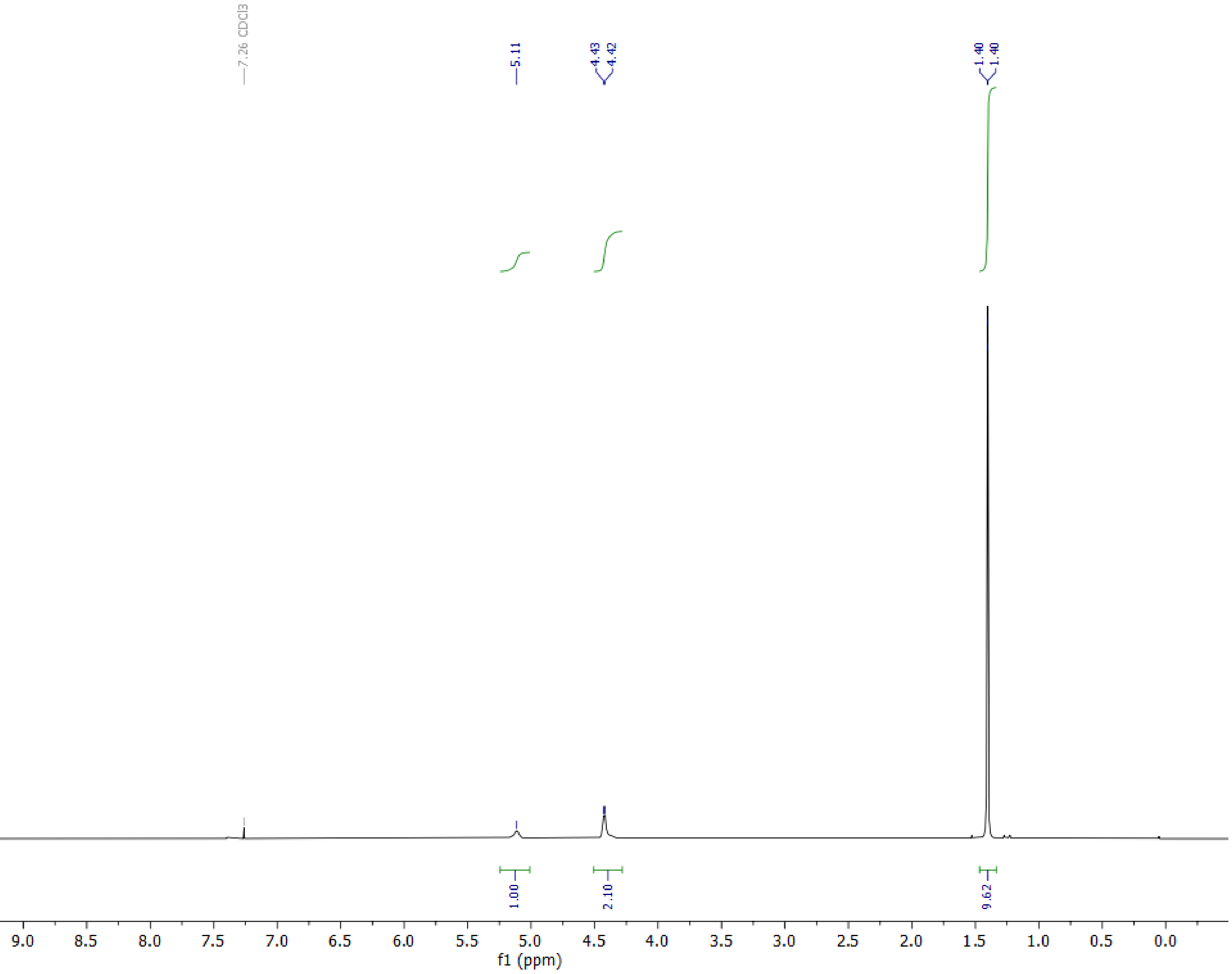

^{13}C NMR of *N*-Boc-4-chlorotetrafluorobenzylamine (23a)

$CDCl_3$, 23 ºC

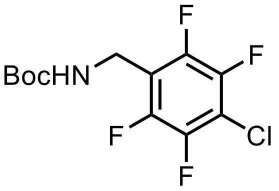

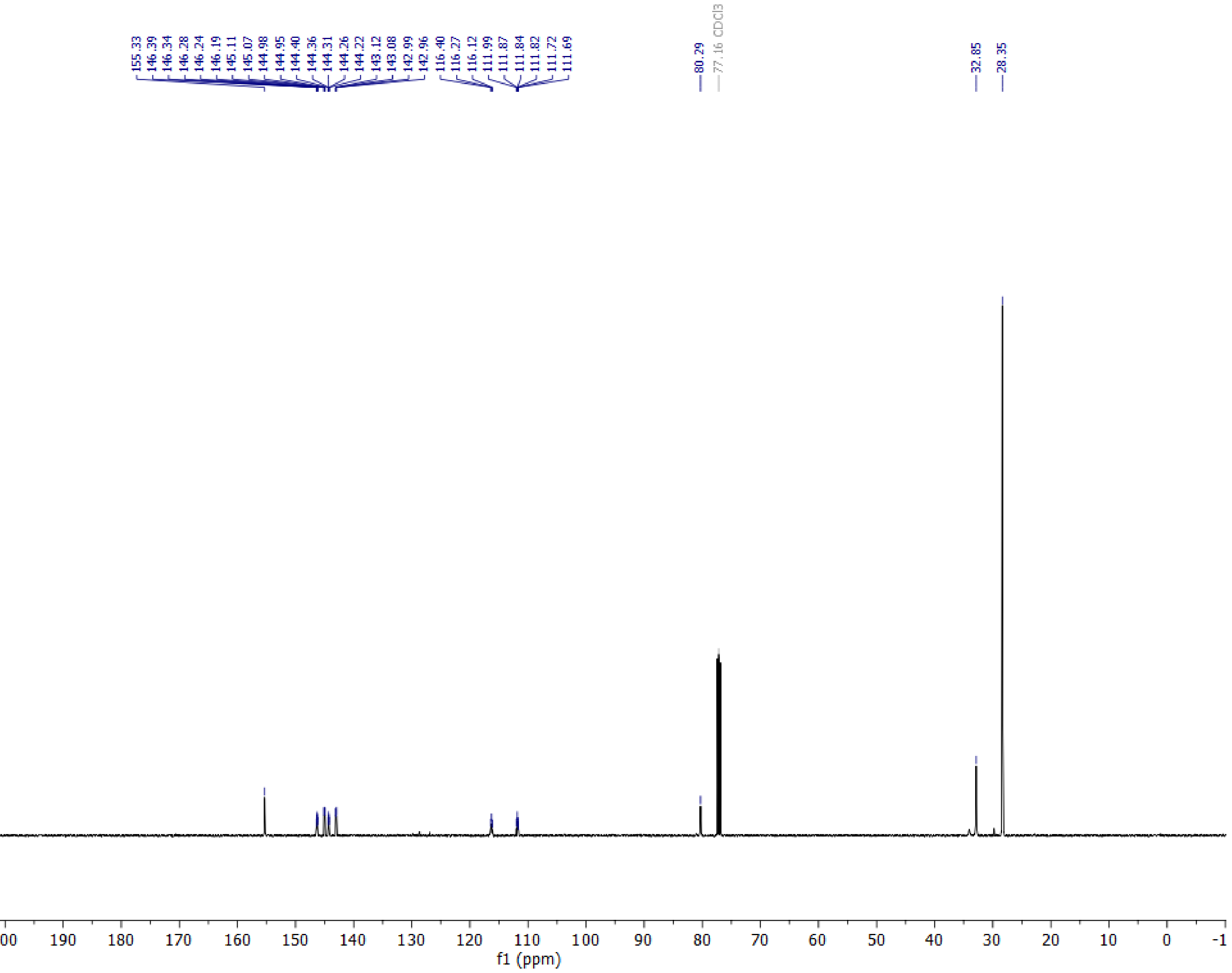

^{19}F NMR of *N*-Boc-4-chlorotetrafluorobenzylamine (23a)

CDCl$_3$, 23 ºC

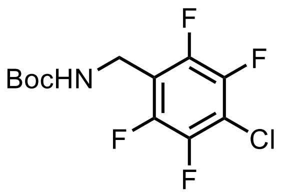

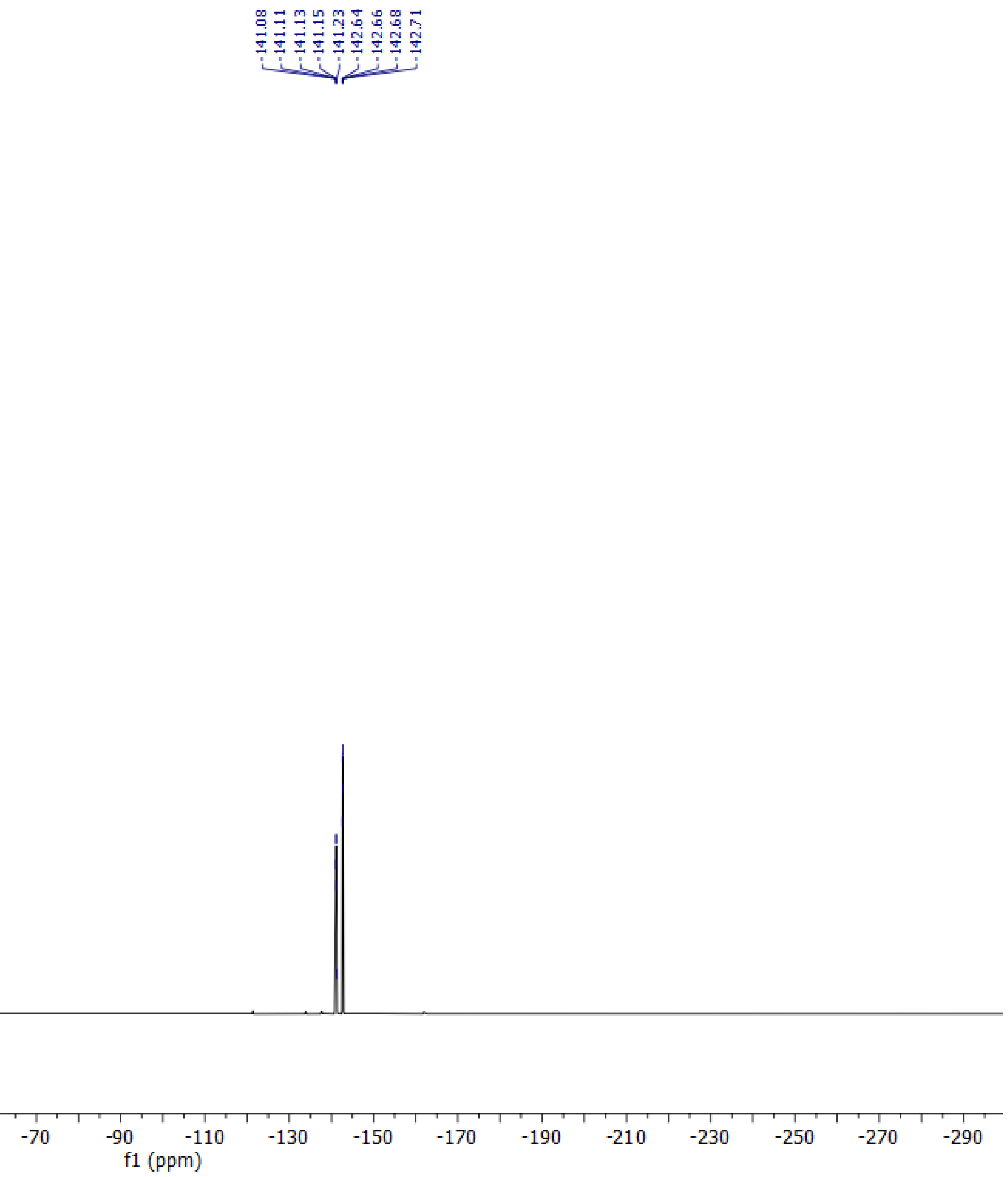

^{1}H NMR of *N*-Boc-2-chlorotetrafluorobenzylamine (23b)

$CDCl_3$, 23 ºC

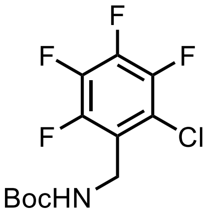

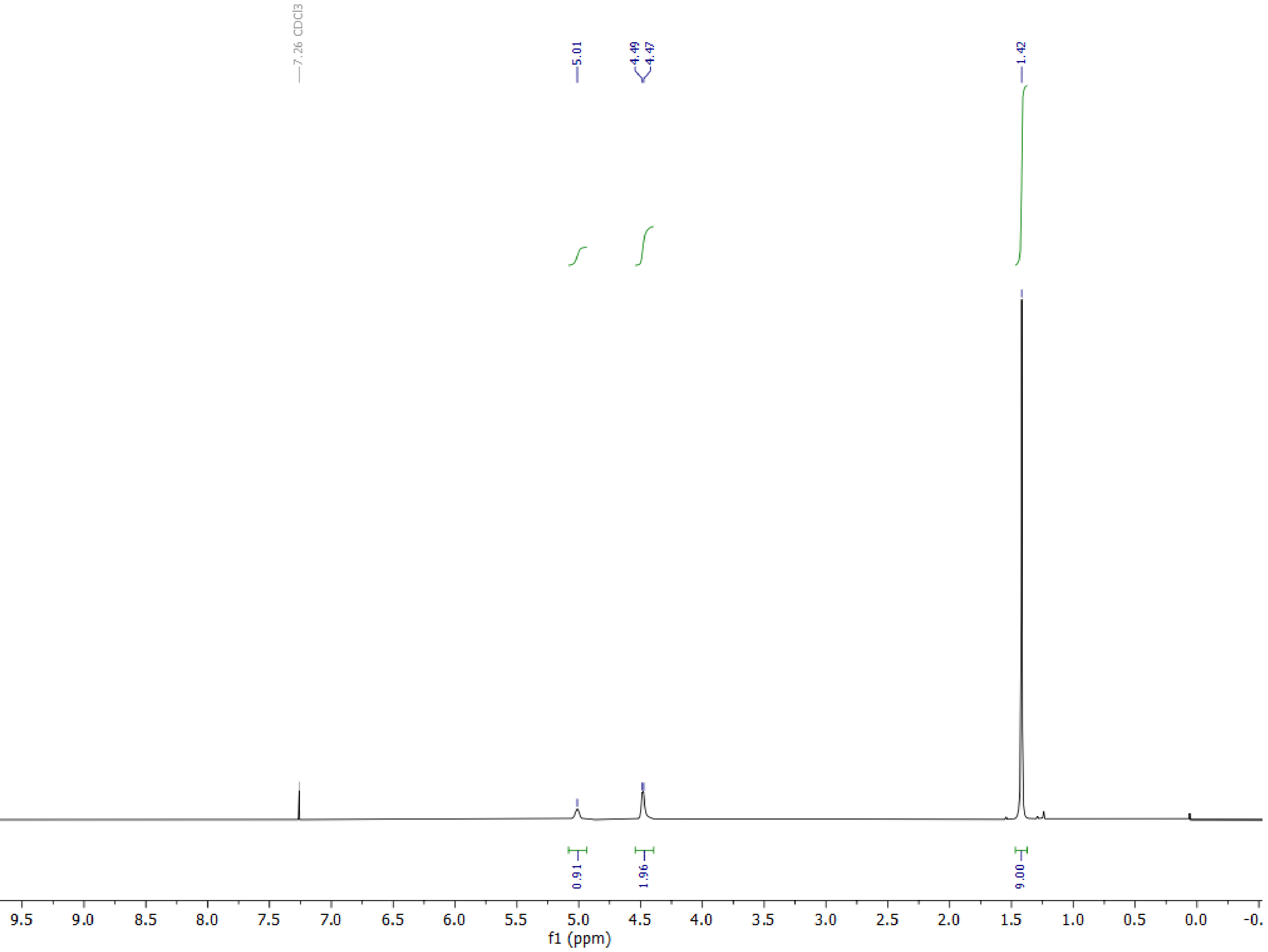

^{13}C NMR of *N*-Boc-2-chlorotetrafluorobenzylamine (23b)

$CDCl_3$, 23 ºC

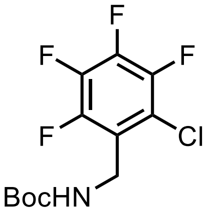

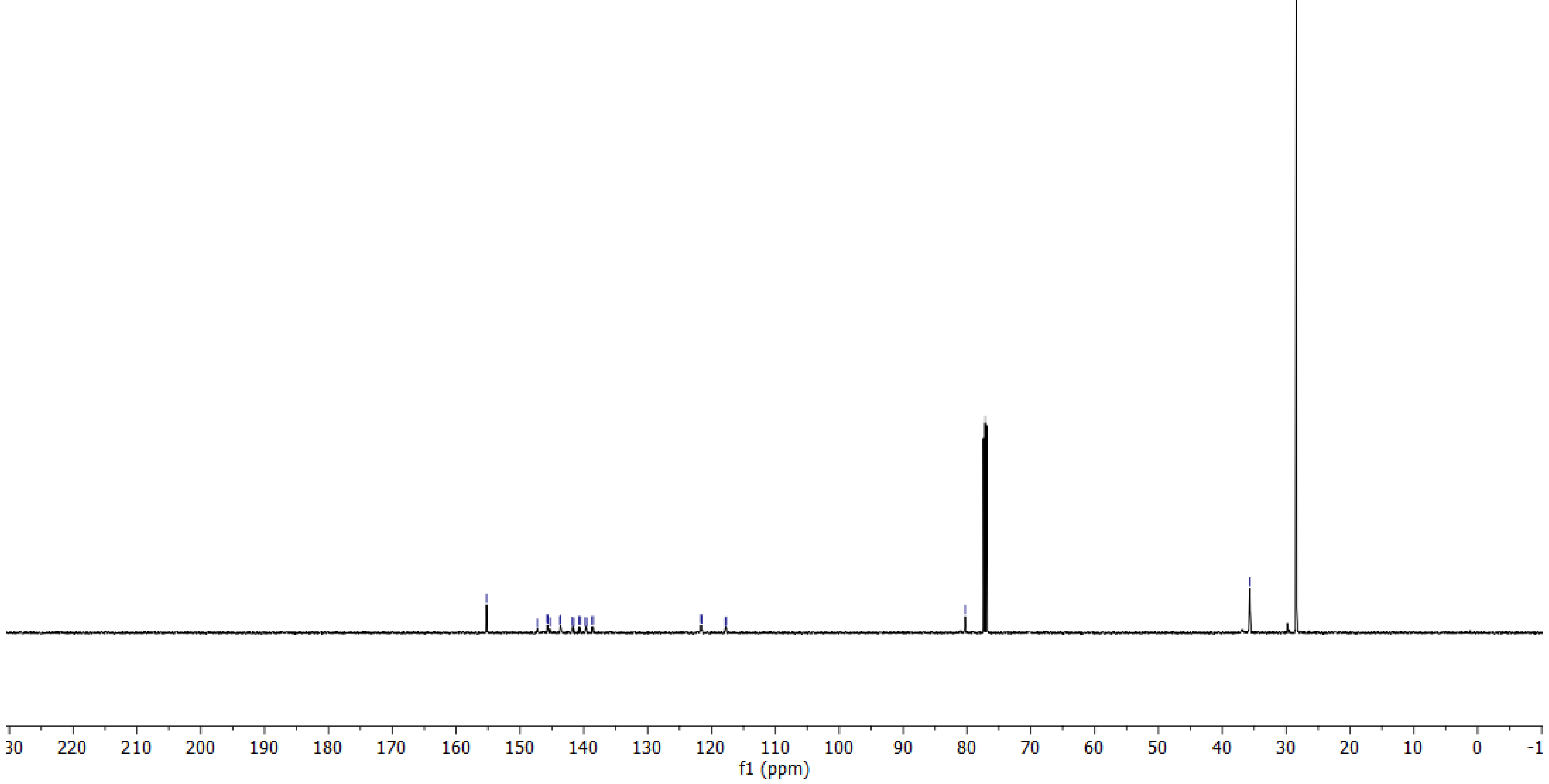

^{19}F NMR of *N*-Boc-2-chlorotetrafluorobenzylamine (23b)

$CDCl_3$, 23 ºC

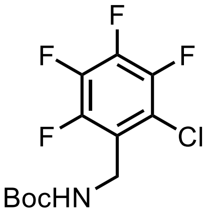

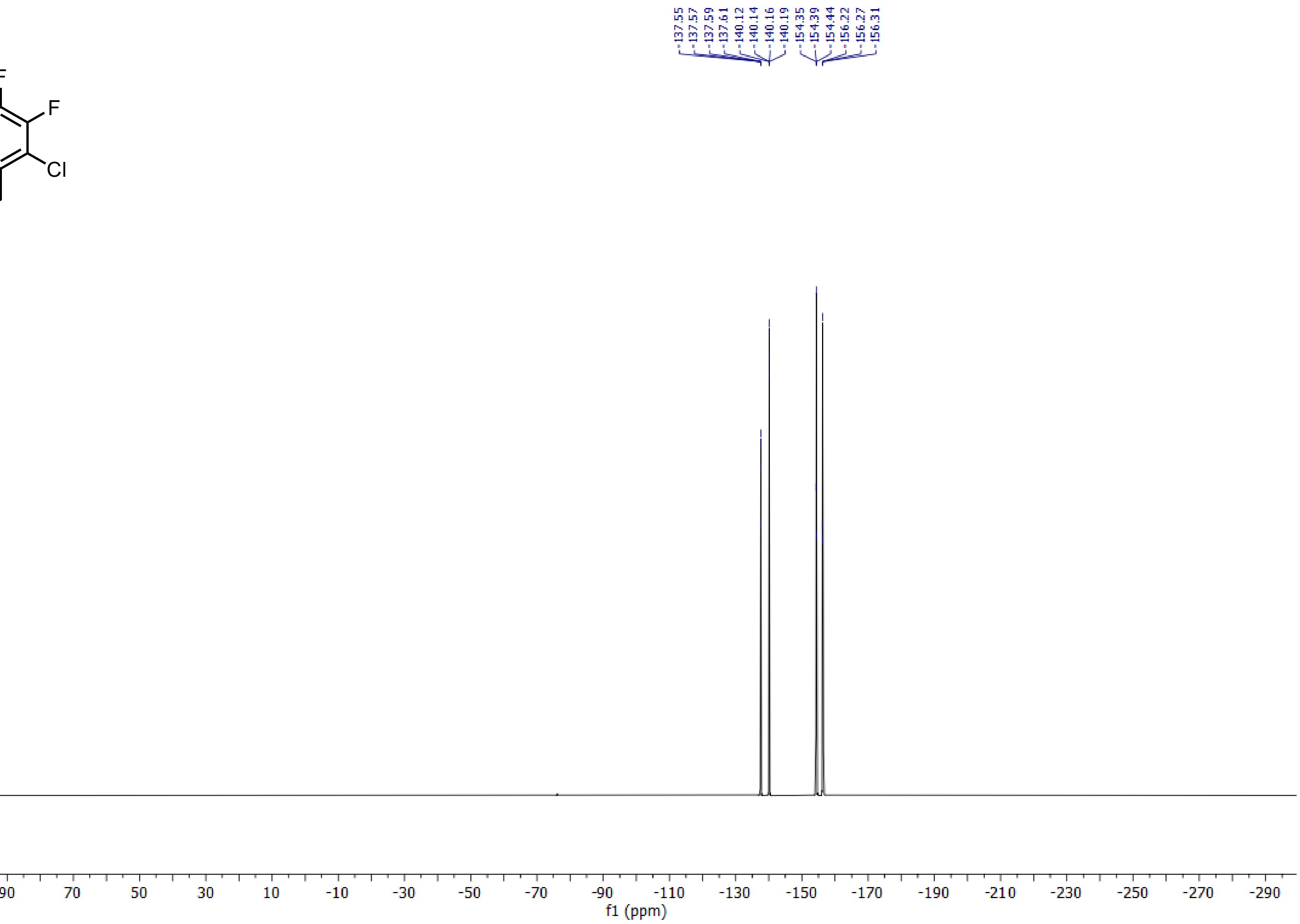

^{1}H NMR of *N*-Boc-3-chlorotetrafluorobenzylamine (23c)

$CDCl_3$, 23 ºC

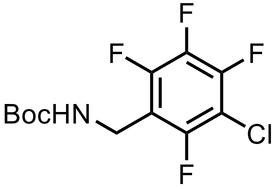

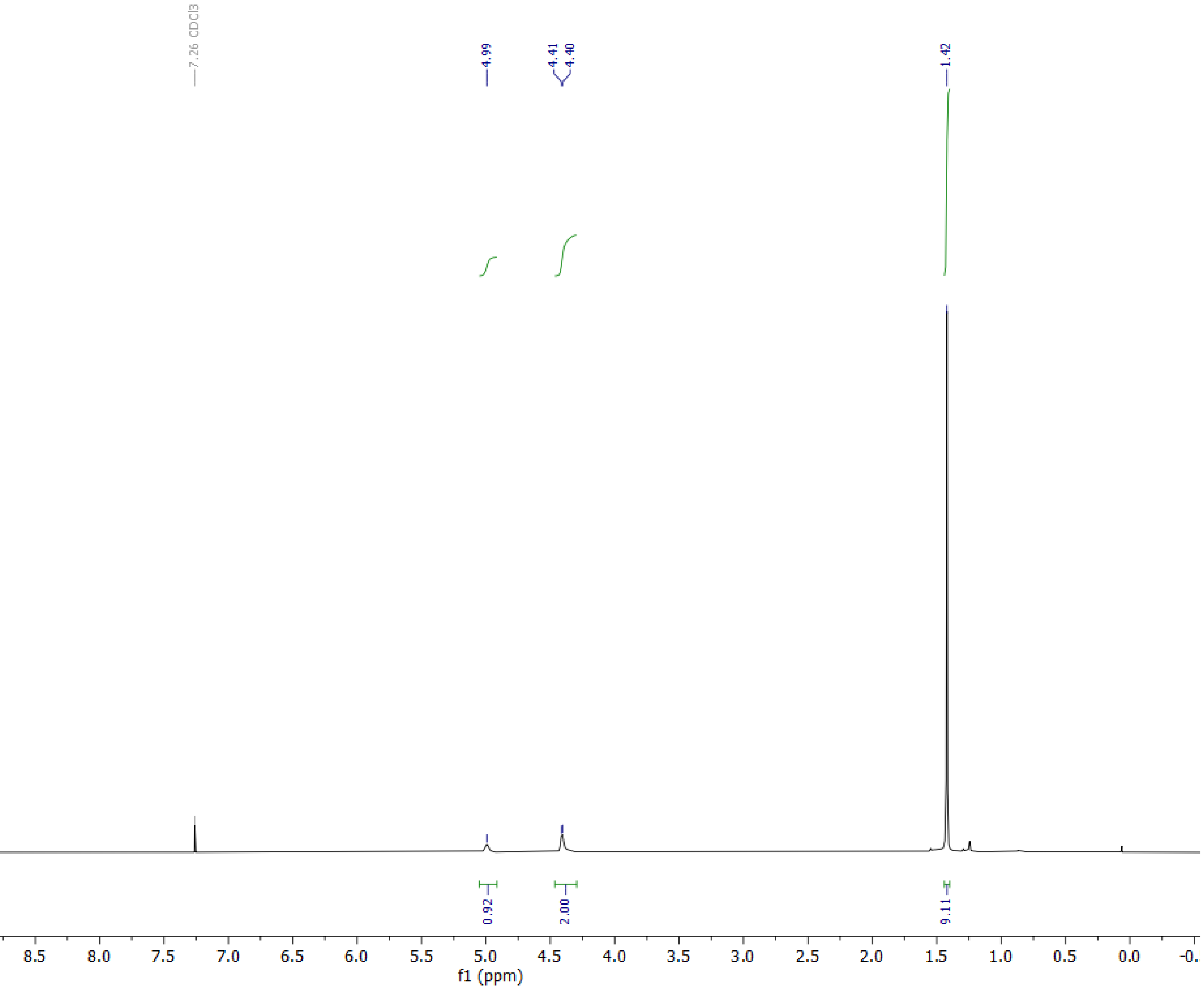

^{13}C NMR of *N*-Boc-3-chlorotetrafluorobenzylamine (23c)

$CDCl_3$, 23 ºC

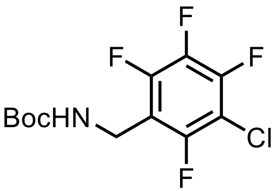

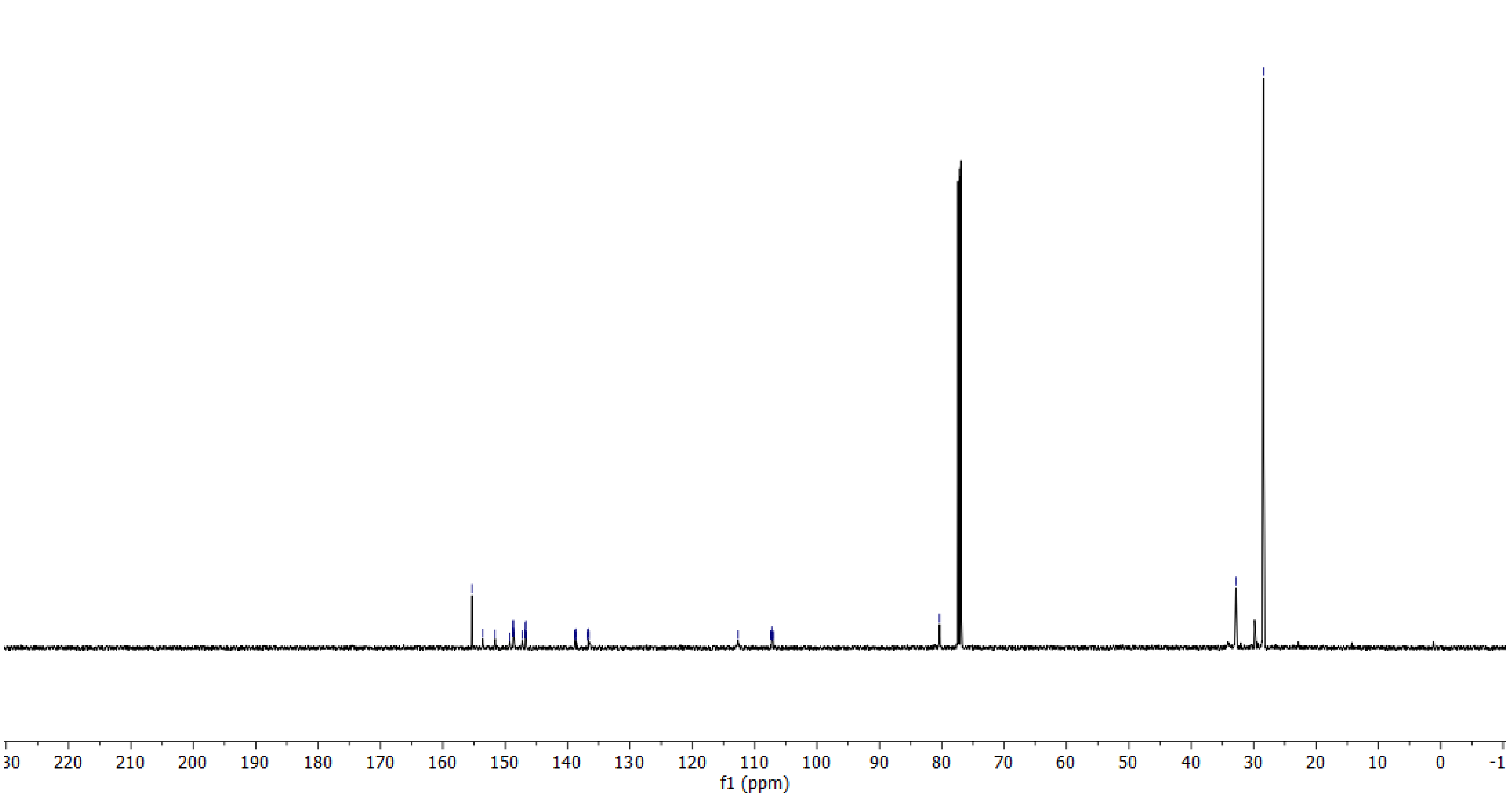

^{19}F NMR of *N*-Boc-3-chlorotetrafluorobenzylamine (23c)

$CDCl_3$, 23 ºC

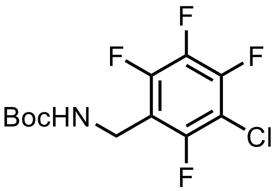

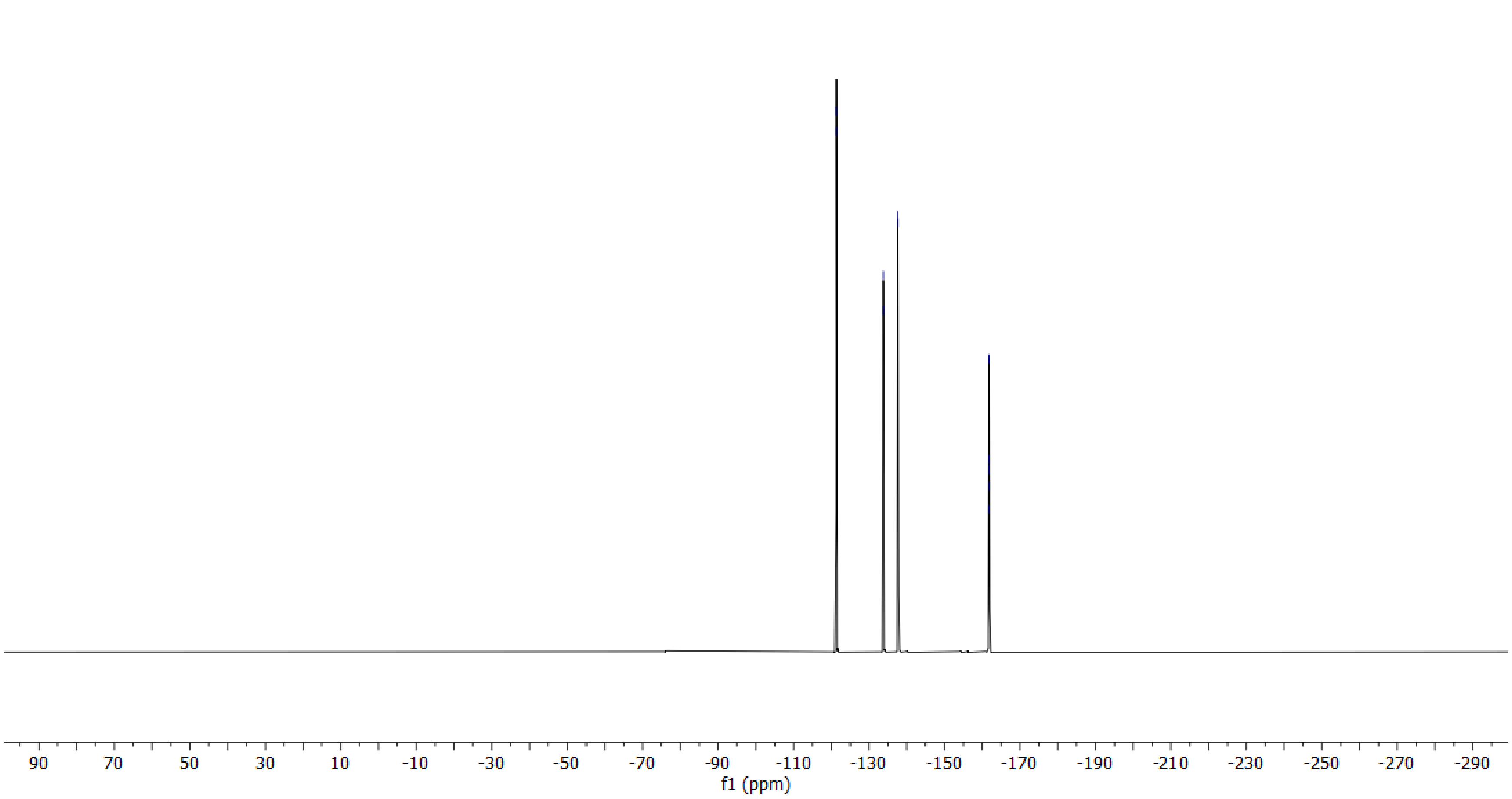

^{1}H NMR of *N*-Boc-2,3,5,6-tetrafluorobenzylamine (24a)

$CDCl_3$, 23 ºC

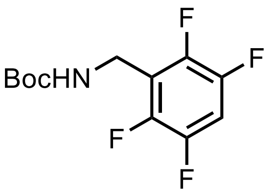

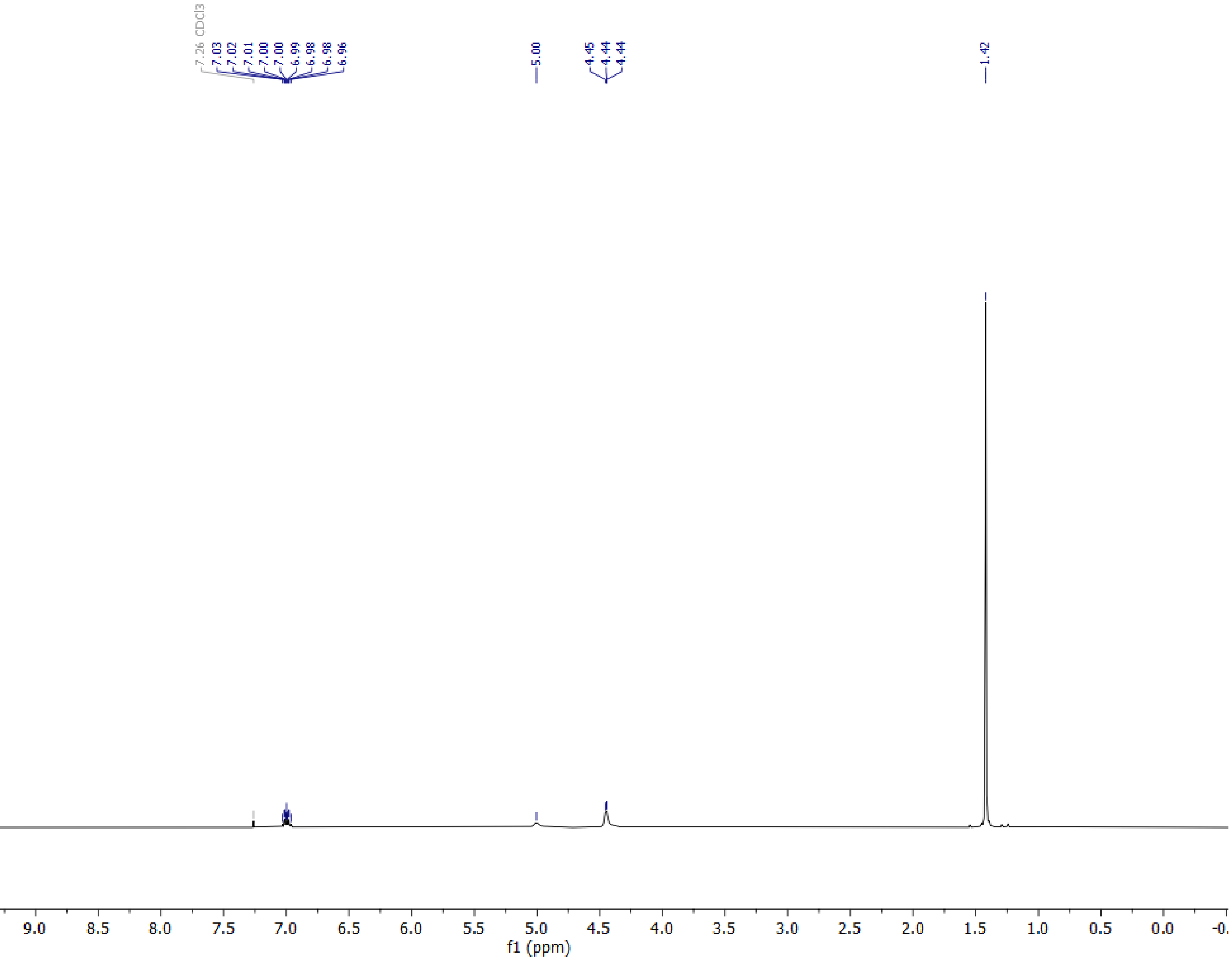

^{13}C NMR of *N*-Boc-2,3,5,6-tetrafluorobenzylamine (24a)

$CDCl_3$, 23 ºC

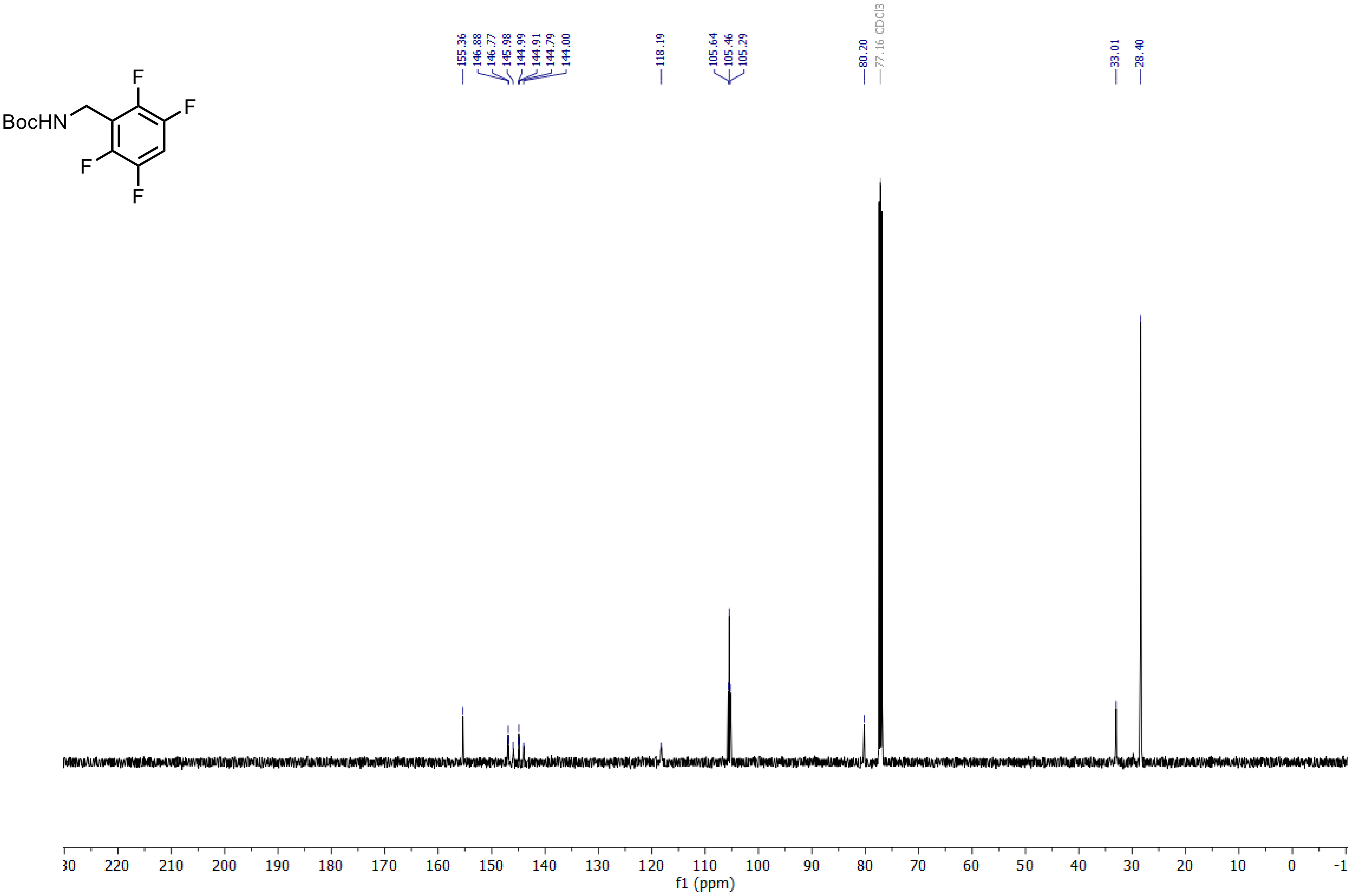

^{19}F NMR of *N*-Boc-2,3,5,6-tetrafluorobenzylamine (24a)

$CDCl_3$, 23 ºC

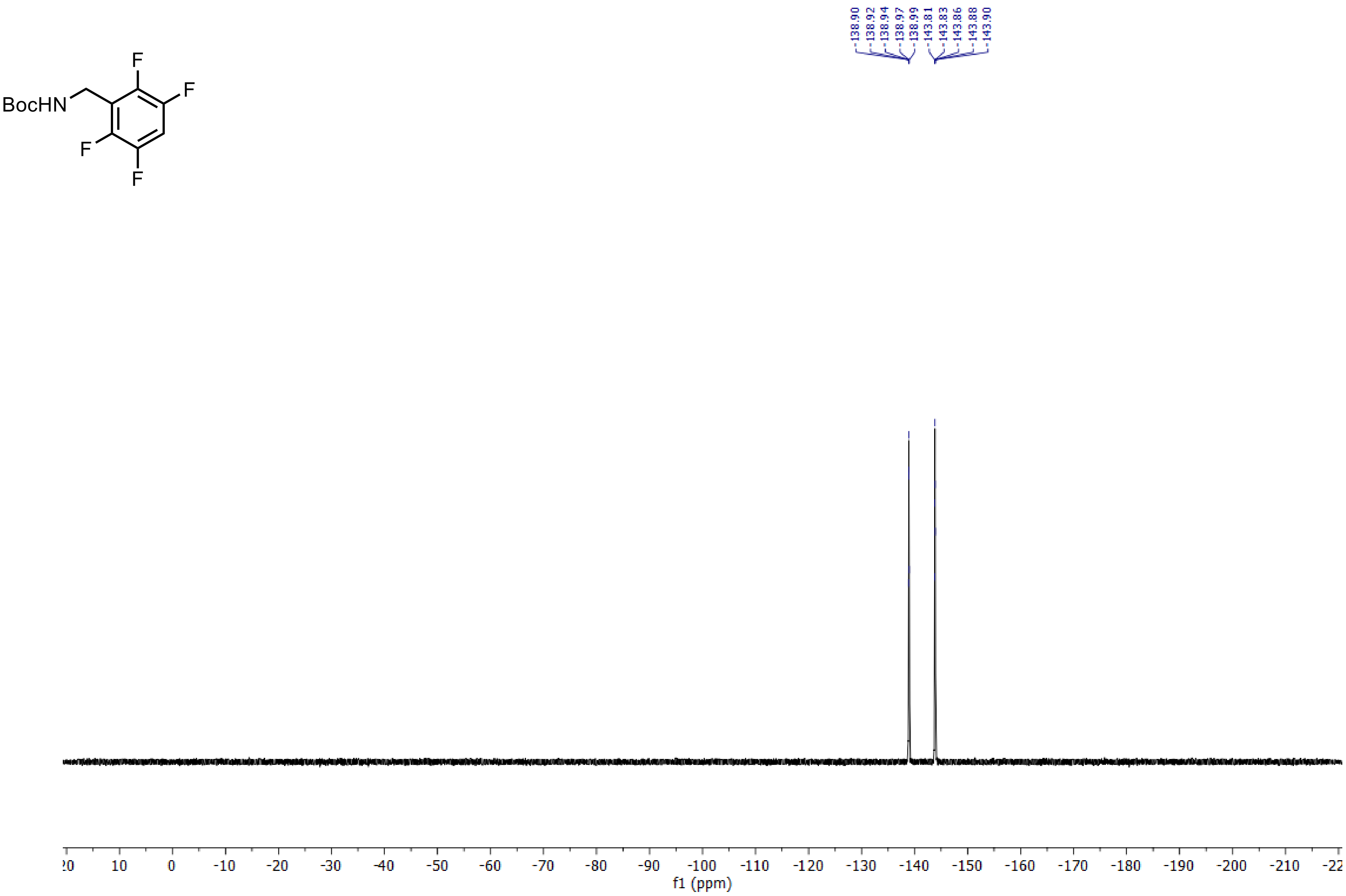

^{1}H NMR of *N*-Boc-2,3,4,6-tetrafluorobenzylamine (24b)

$CDCl_3$, 23 ºC

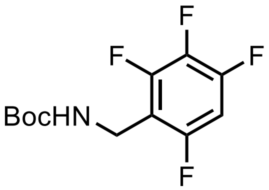

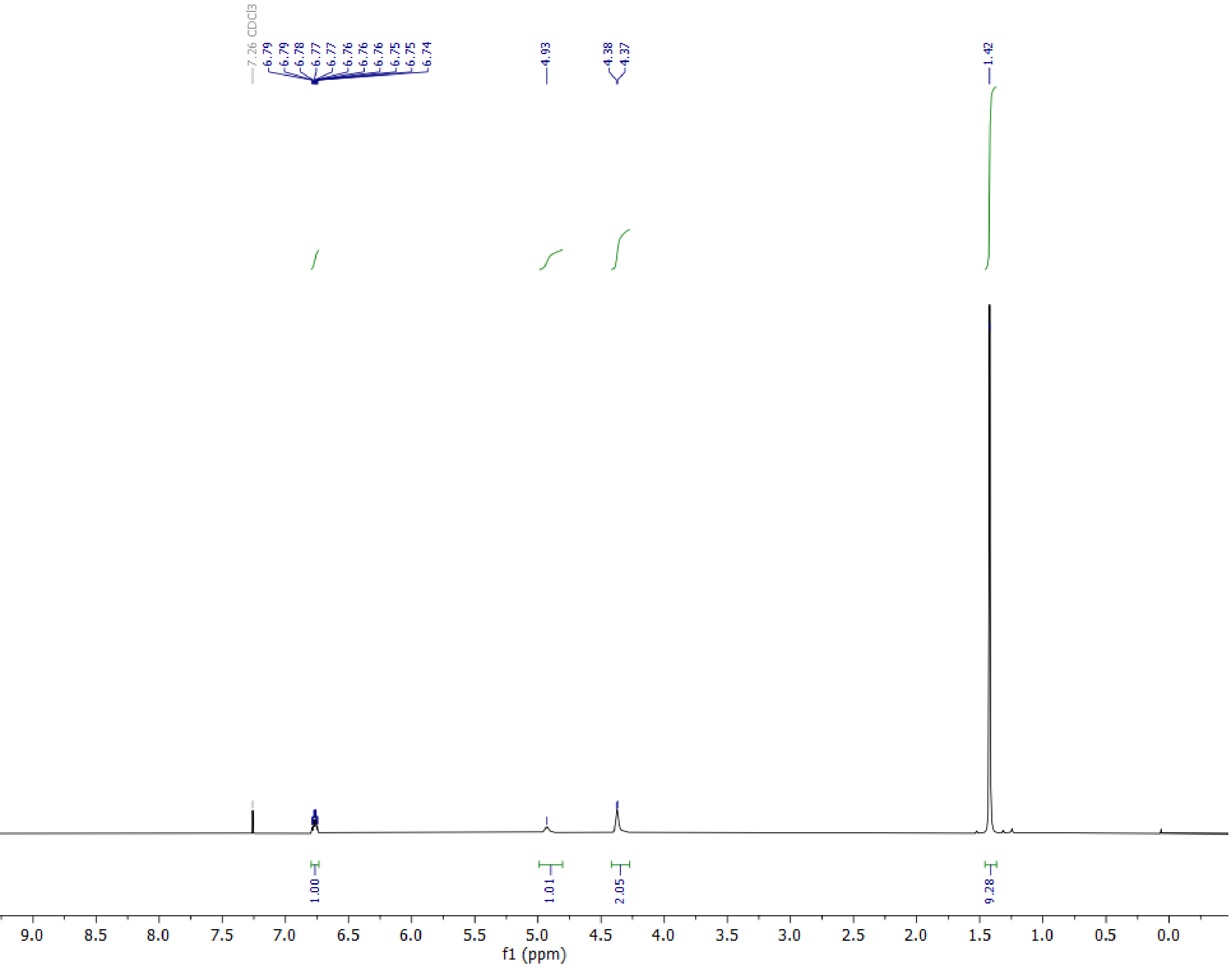

^{13}C NMR of *N*-Boc-2,3,4,6-tetrafluorobenzylamine (24b)

$CDCl_3$, 23 ºC

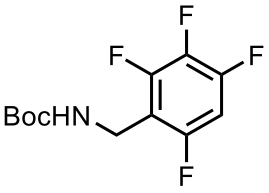

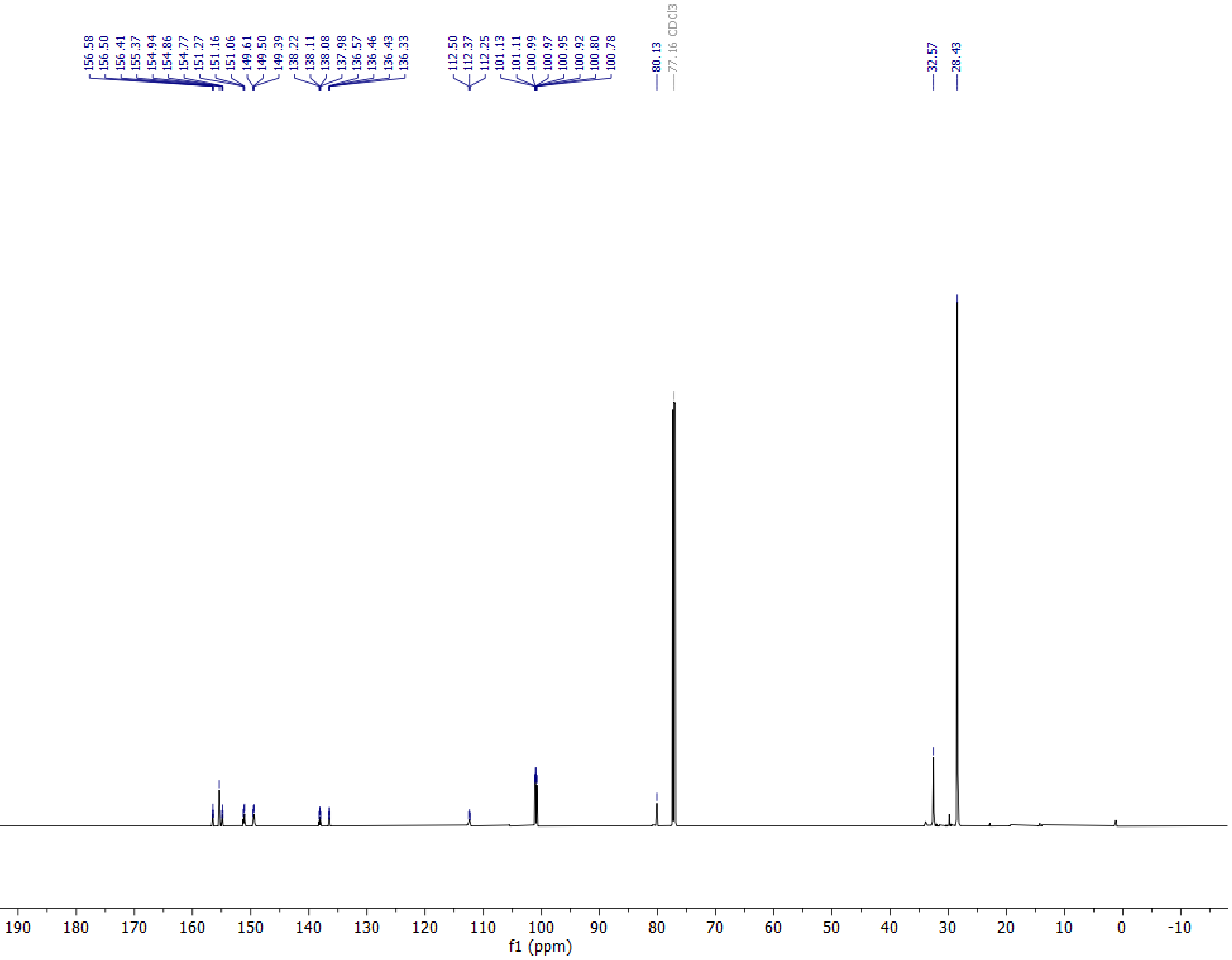

^{19}F NMR of *N*-Boc-2,3,4,6-tetrafluorobenzylamine (24b)

$CDCl_3$, 23 ºC

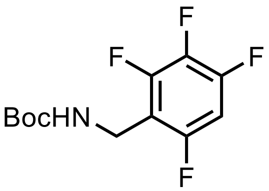

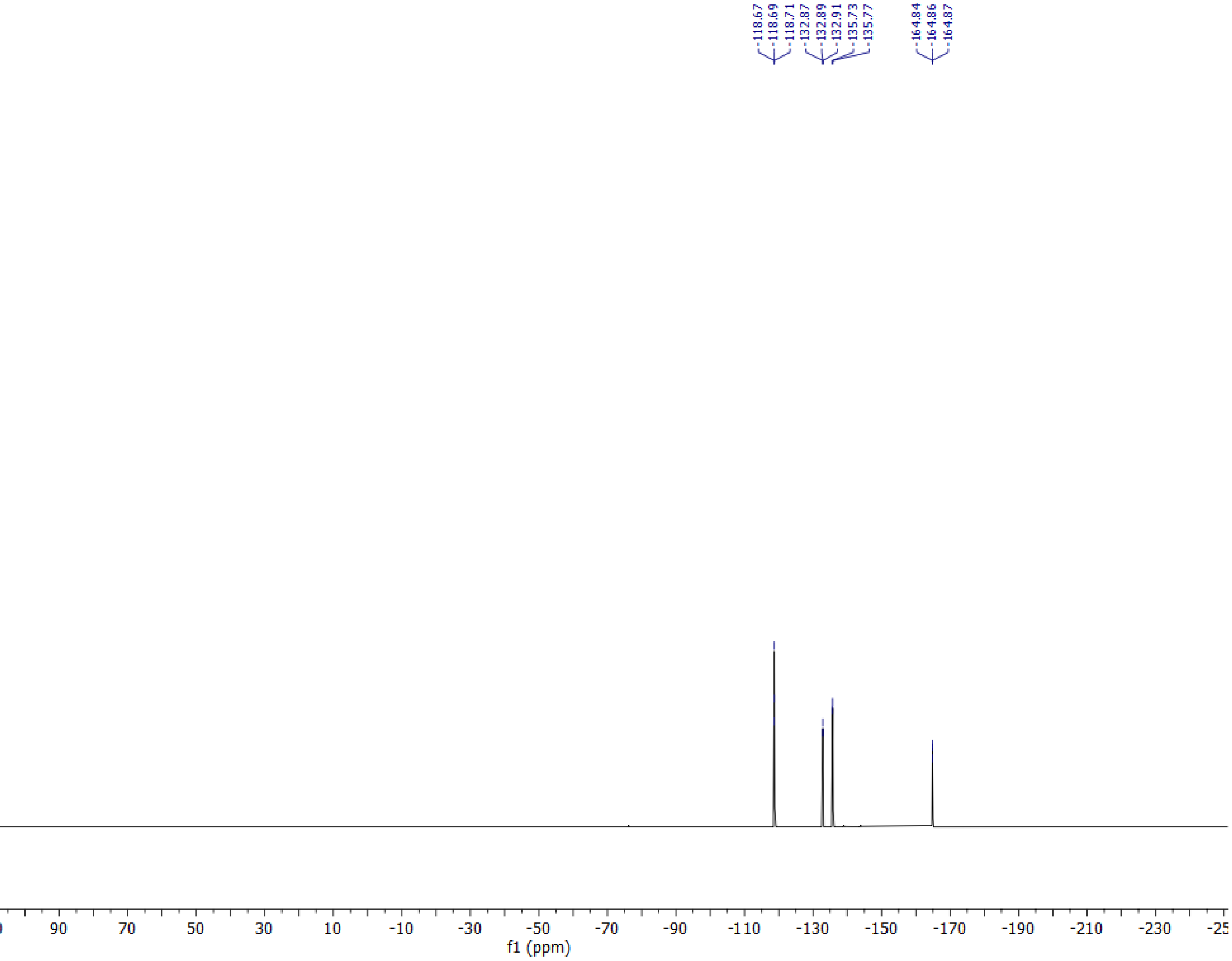

^{1}H NMR of *N*-Boc-2,3,6-trifluoro-5-(trifluoromethyl)benzylamine (25a)

$CDCl_3$, 23 ºC

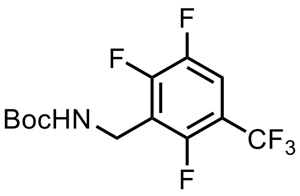

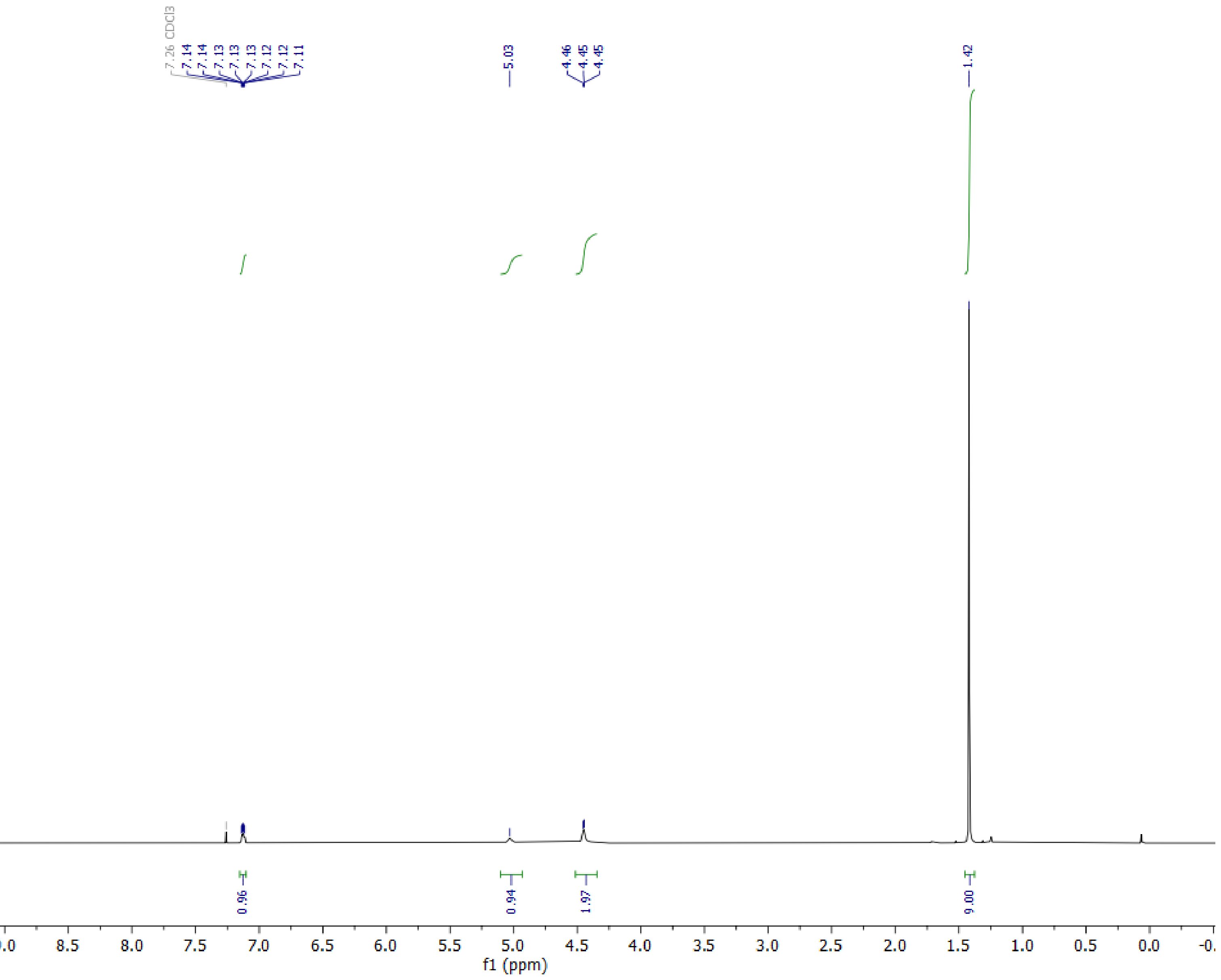

^{13}C NMR of *N*-Boc-2,3,6-trifluoro-5-(trifluoromethyl)benzylamine (25a)

$CDCl_3$, 23 ºC

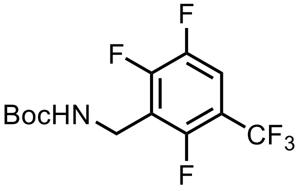

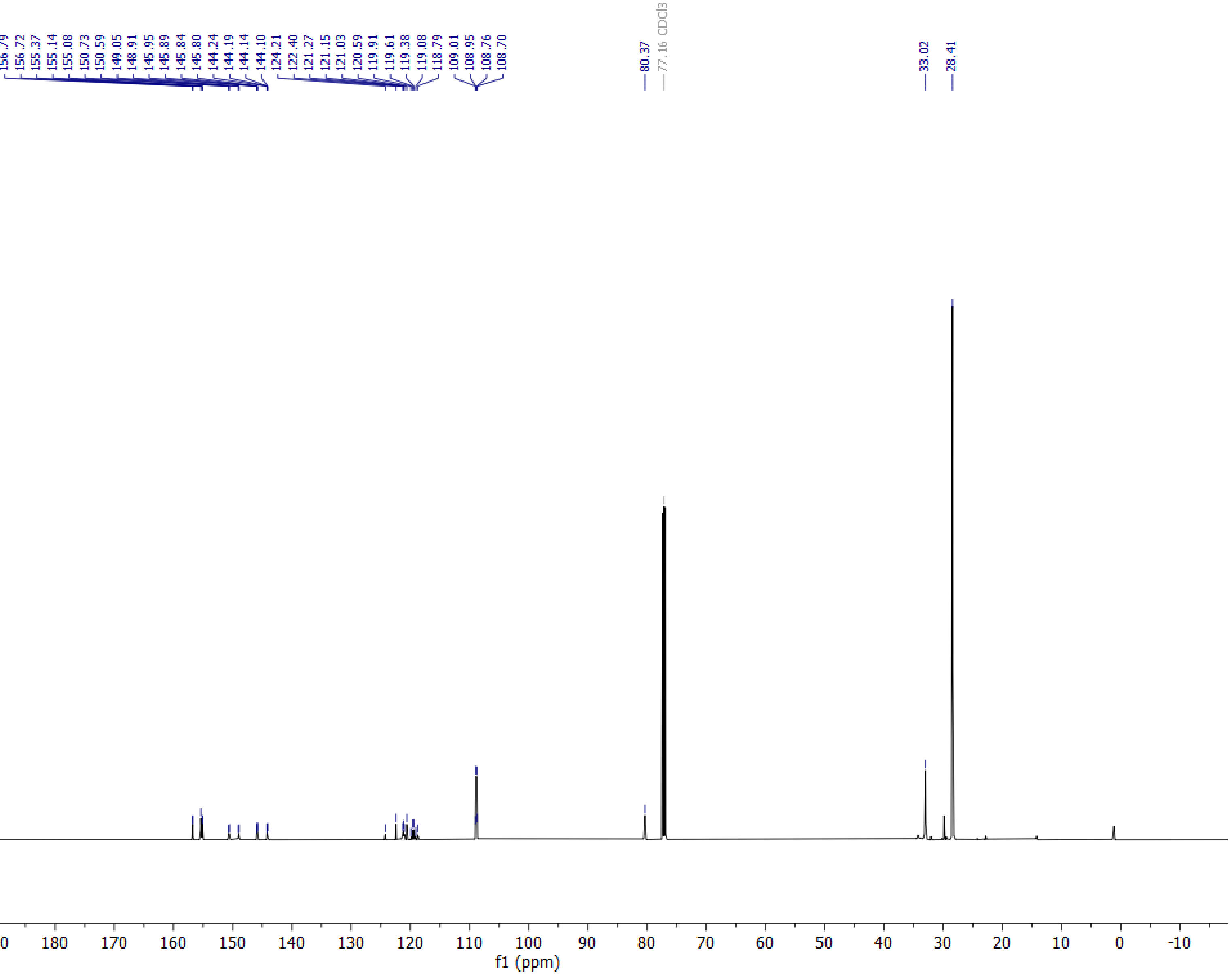

^{19}F NMR of *N*-Boc-2,3,6-trifluoro-5-(trifluoromethyl)benzylamine (25a)

$CDCl_3$, 23 ºC

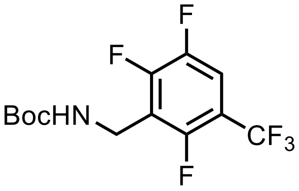

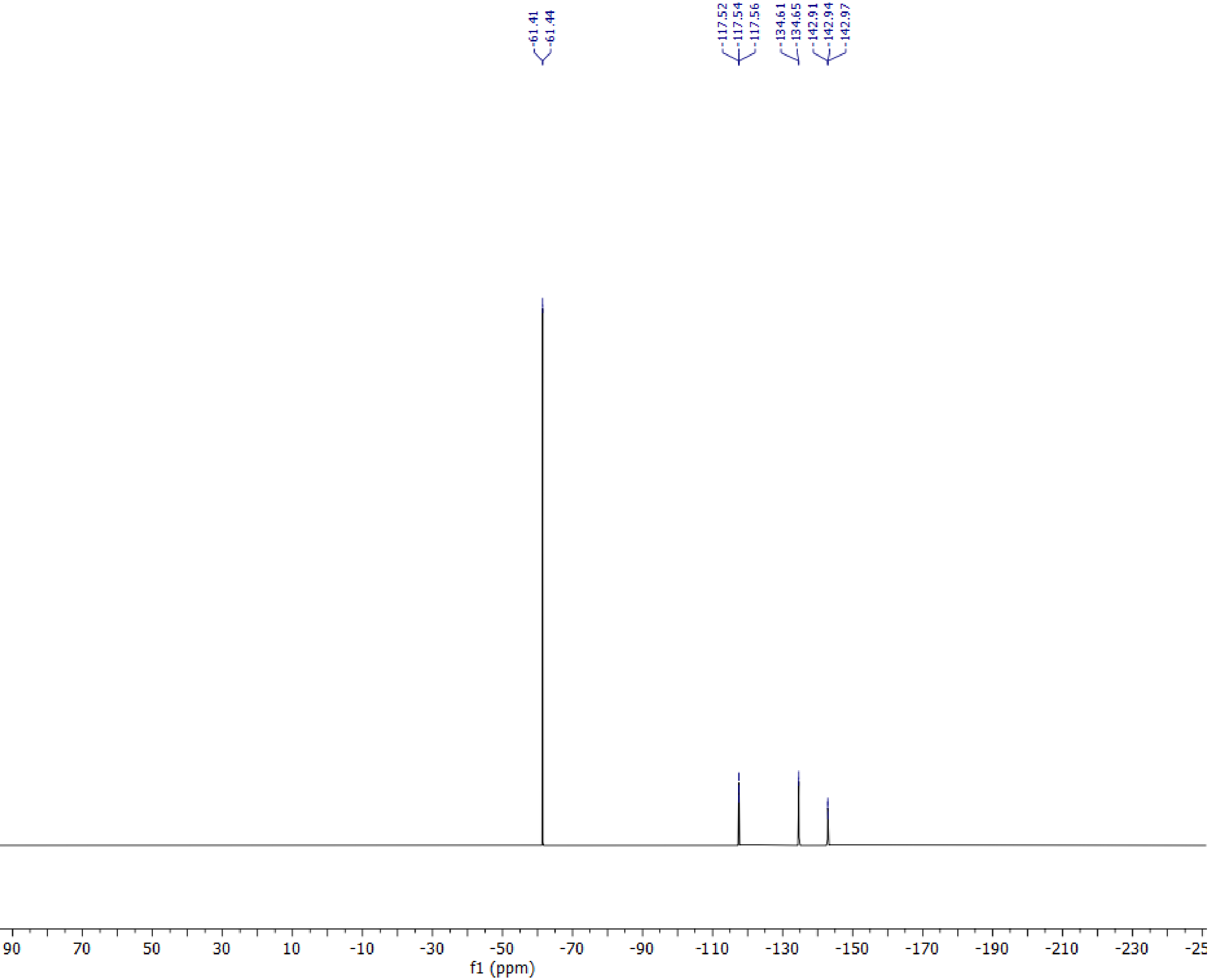

^{1}H NMR of *N*-Boc-2,3,6-trifluoro-4-(trifluoromethyl)benzylamine (25b)

$CDCl_3$, 23 ºC

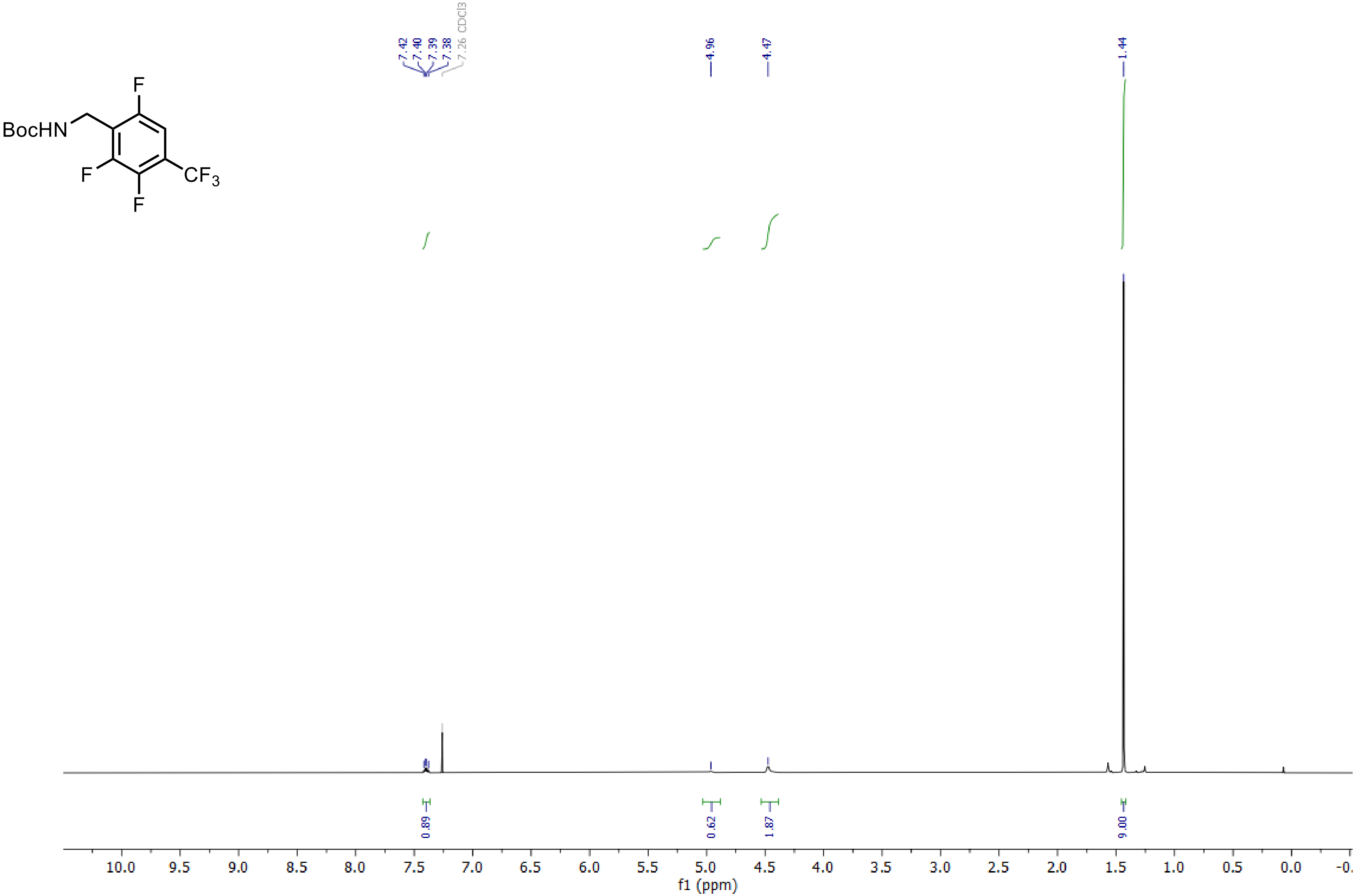

^{13}C NMR of *N*-Boc-2,3,6-trifluoro-4-(trifluoromethyl)benzylamine (25b)

$CDCl_3$, 23 ºC

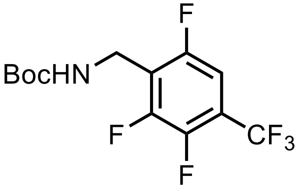

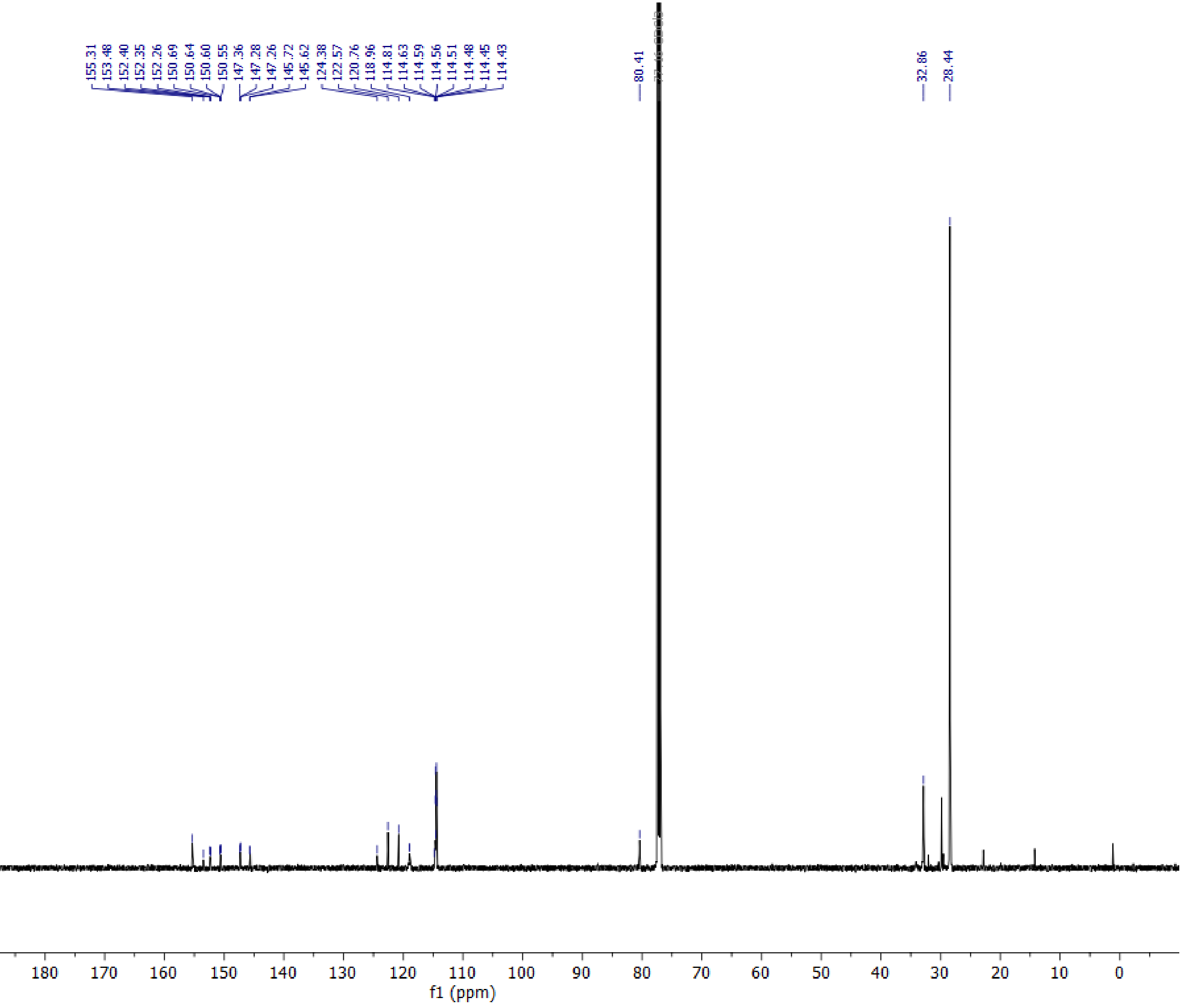

^{19}F NMR of *N*-Boc-2,3,6-trifluoro-4-(trifluoromethyl)benzylamine (25b)

$CDCl_3$, 23 ºC

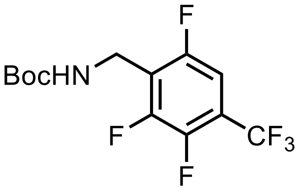

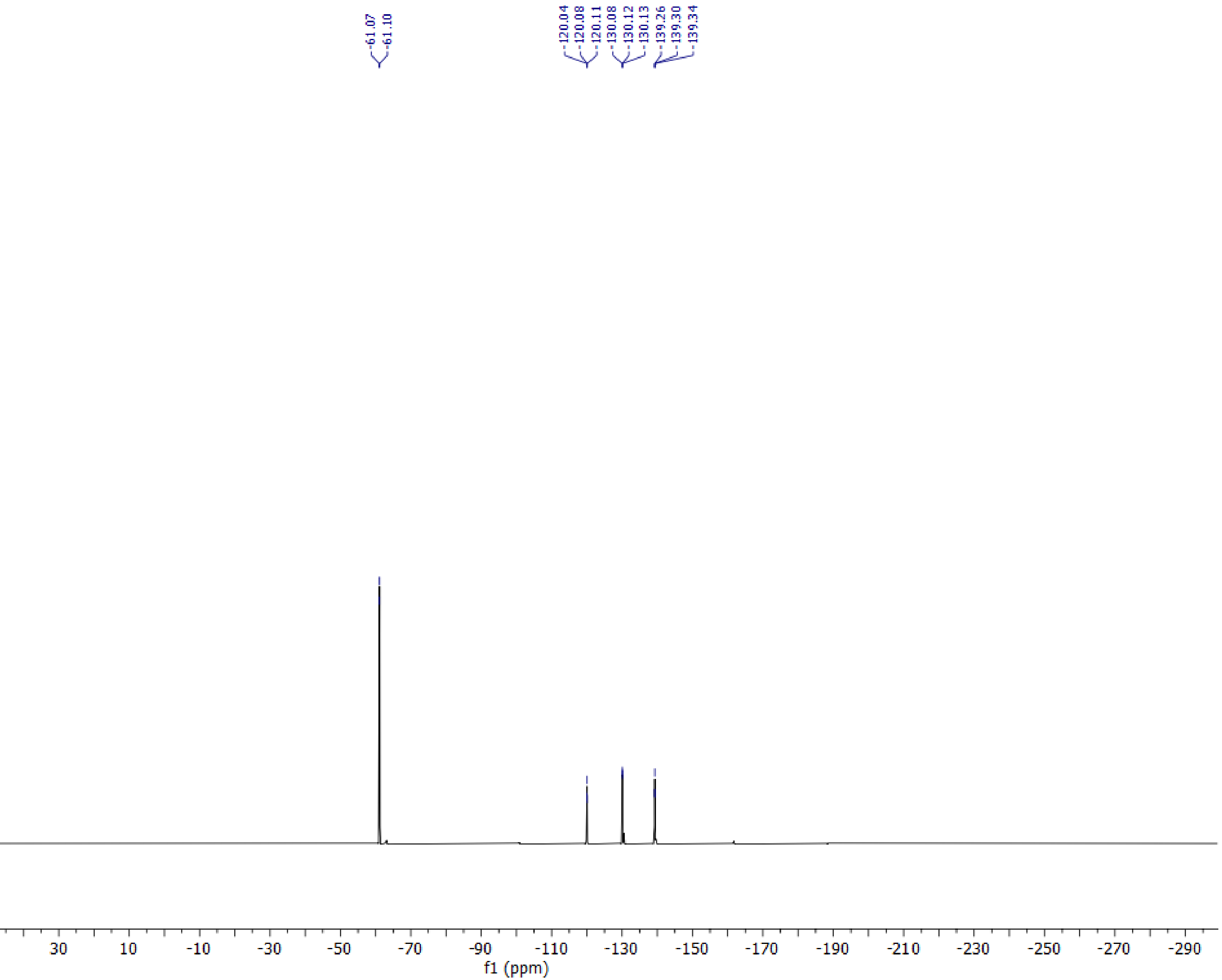

^{1}H NMR of *N*-Boc-2,3,4-trifluoro-6-(trifluoromethyl)benzylamine (25c)

$CDCl_3$, 23 ºC

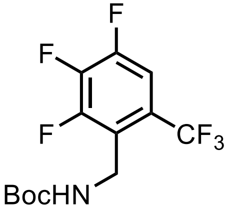

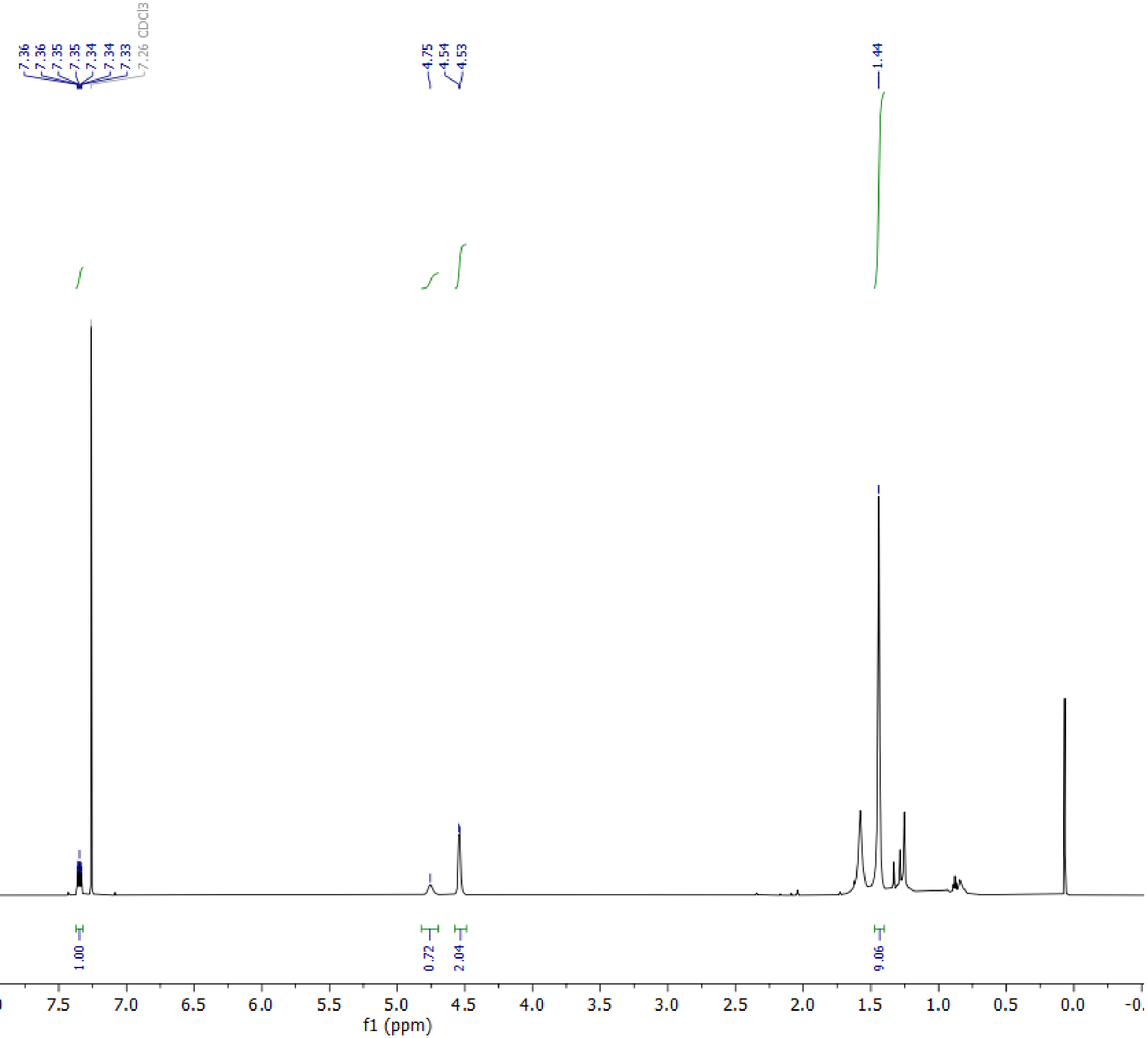

^{13}C NMR of *N*-Boc-2,3,4-trifluoro-6-(trifluoromethyl)benzylamine (25c)

$CDCl_3$, 23 ºC

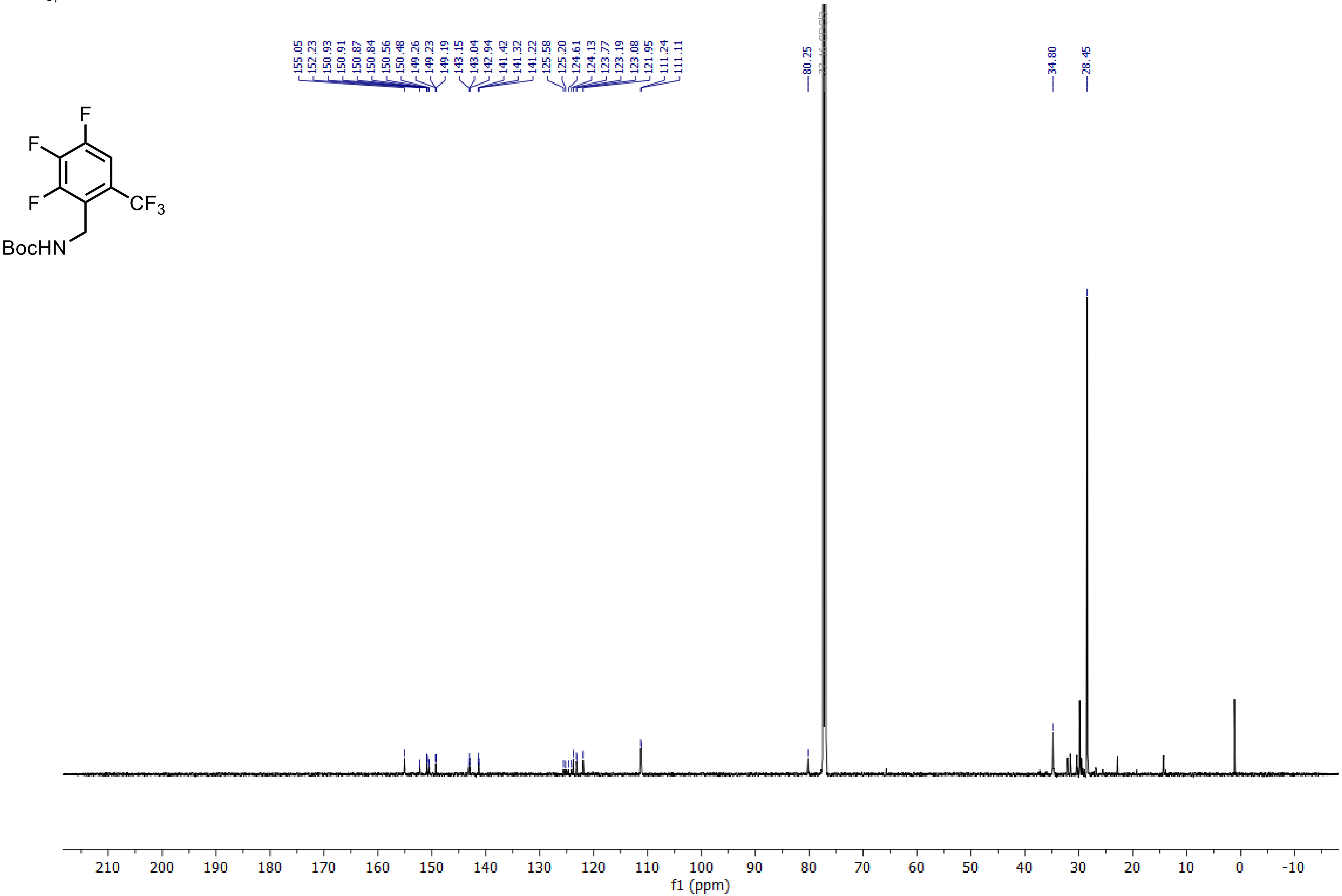

^{19}F NMR of *N*-Boc-2,3,4-trifluoro-6-(trifluoromethyl)benzylamine (25c)

$CDCl_3$, 23 ºC

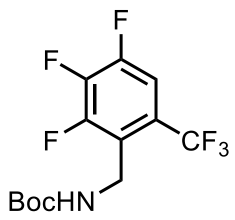

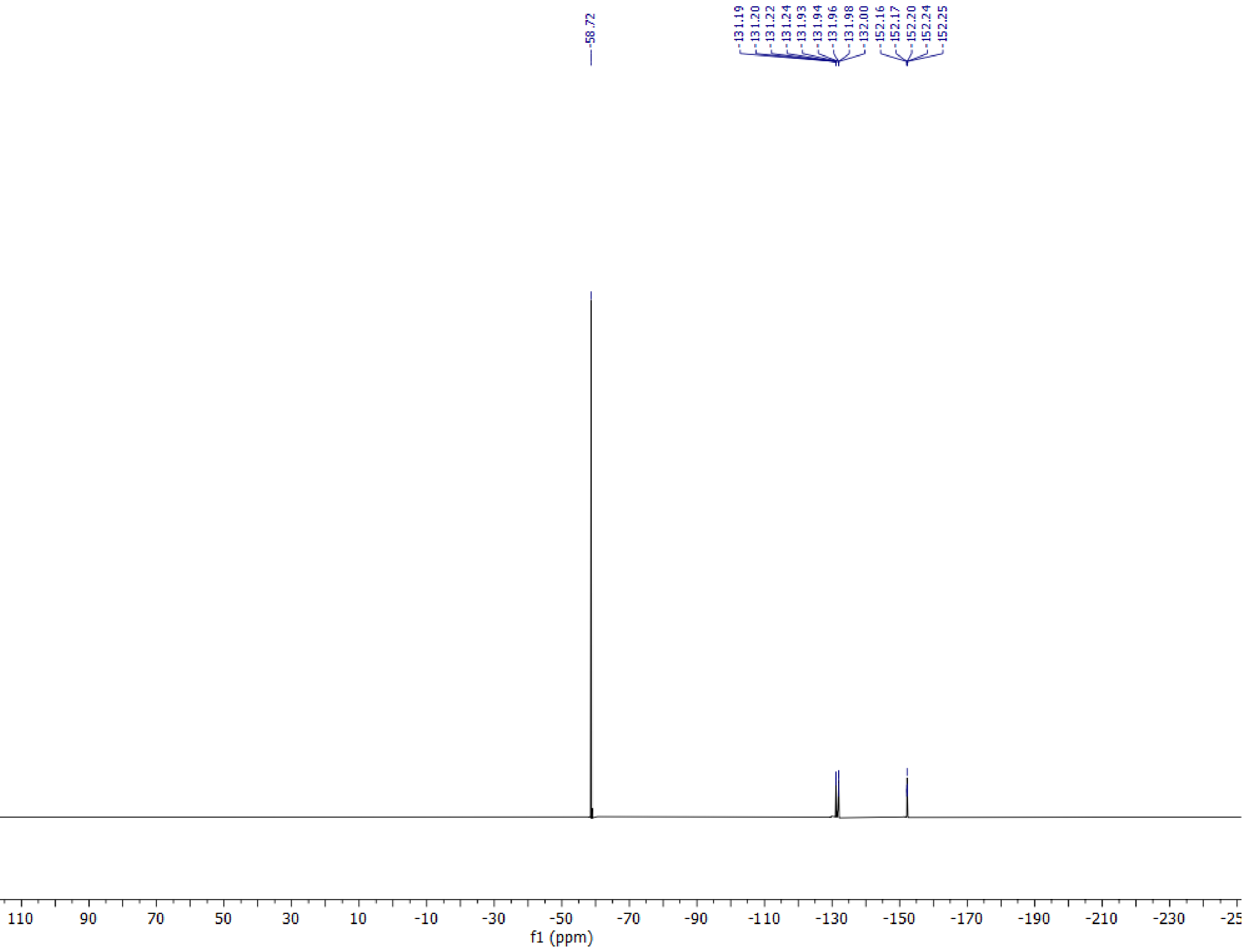

^{1}H NMR of *N*-Boc-2,3,6-trifluoro-4-(methyl)carboxylbenzylamine (26a)

$CDCl_3$, 23 ºC

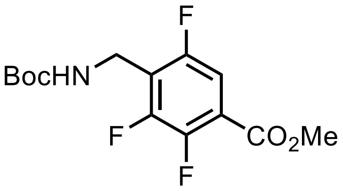

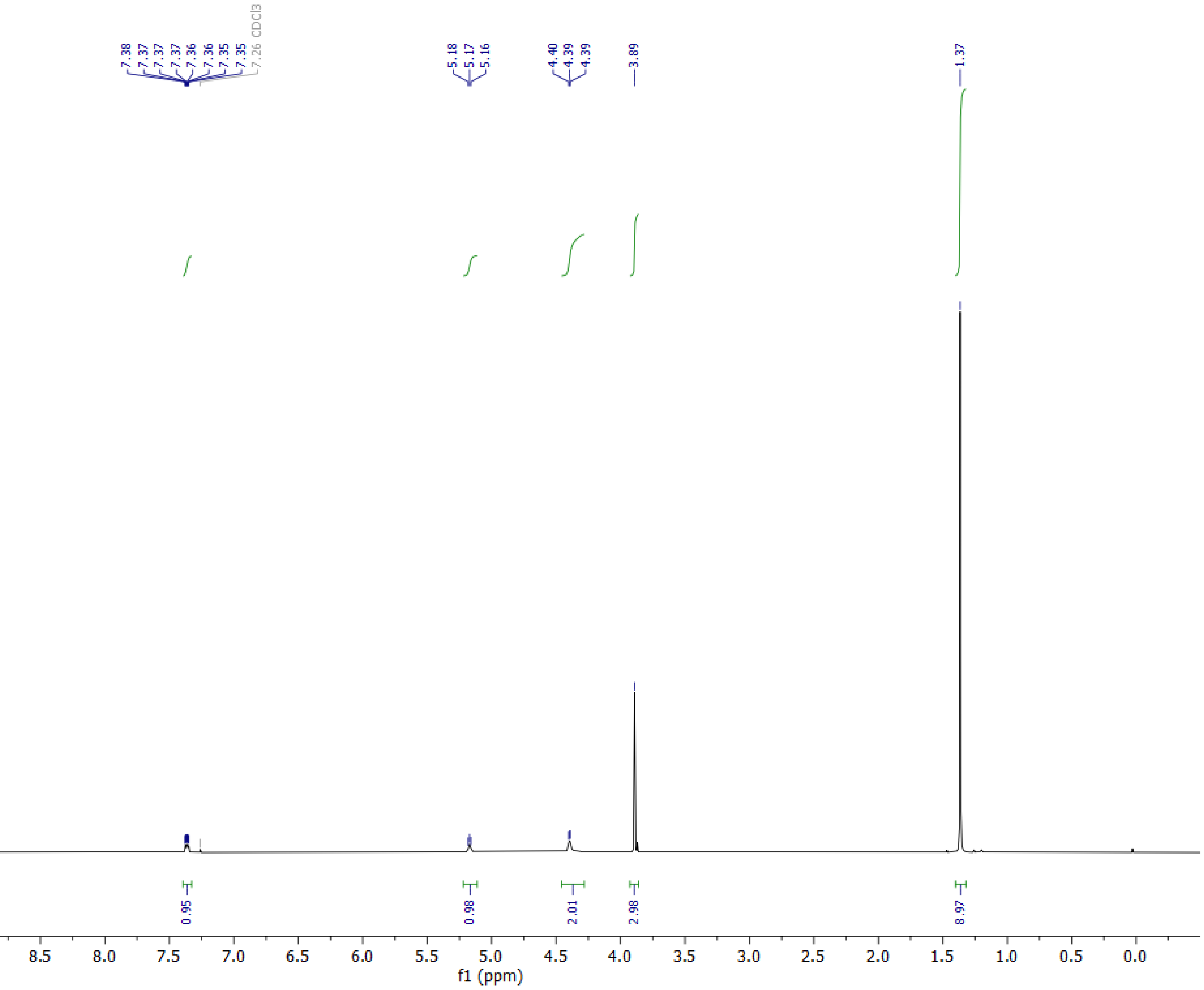

^{13}C NMR of *N*-Boc-2,3,6-trifluoro-4-(methyl)carboxylbenzylamine (26a)

$CDCl_3$, 23 ºC

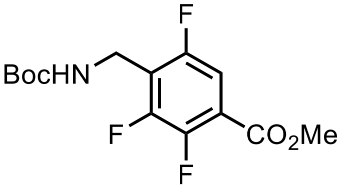

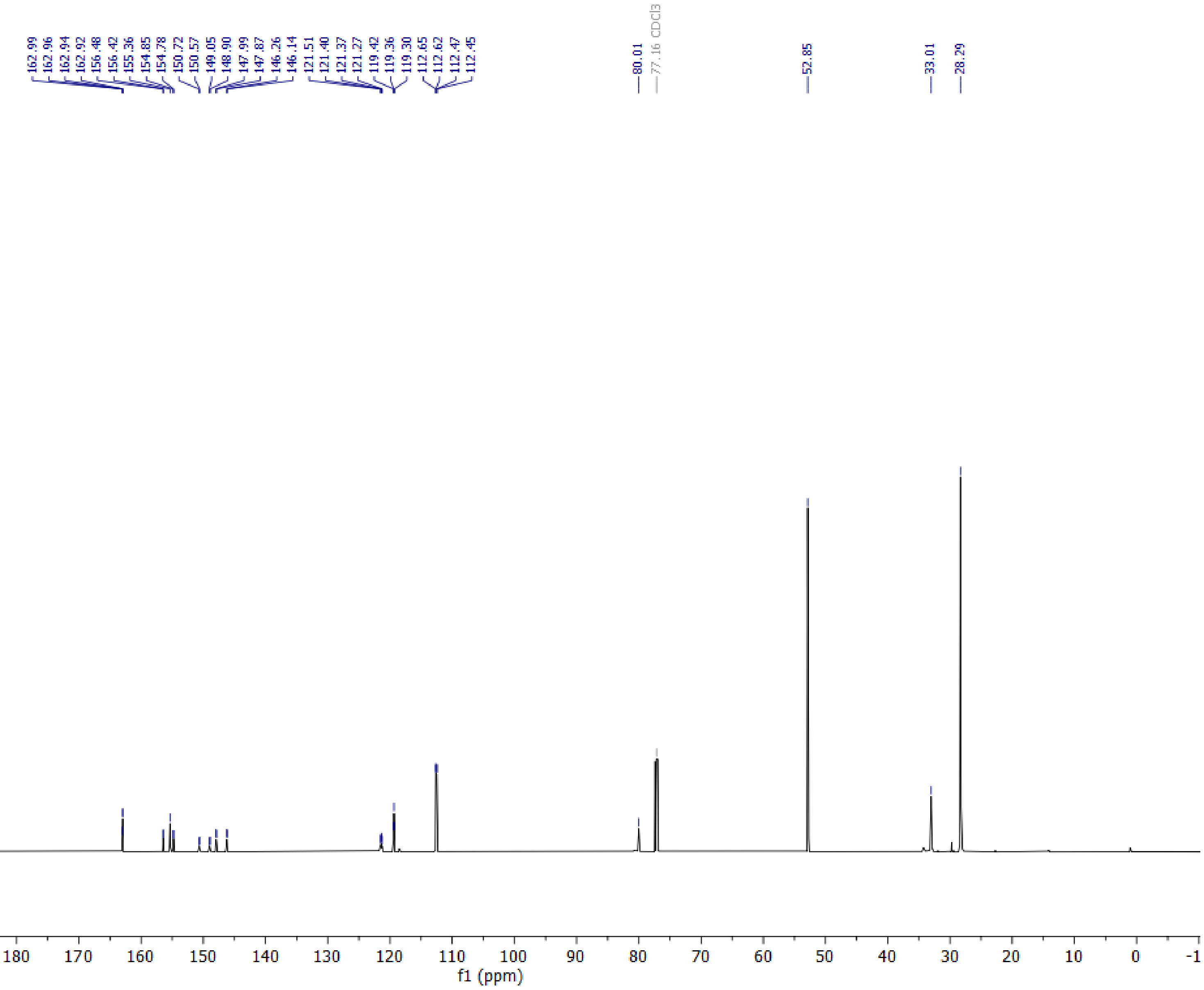

^{19}F NMR of *N*-Boc-2,3,6-trifluoro-4-(methyl)carboxylbenzylamine (26a)

$CDCl_3$, 23 ºC

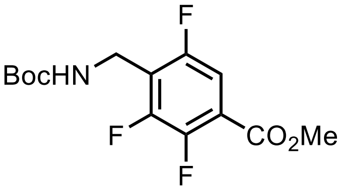

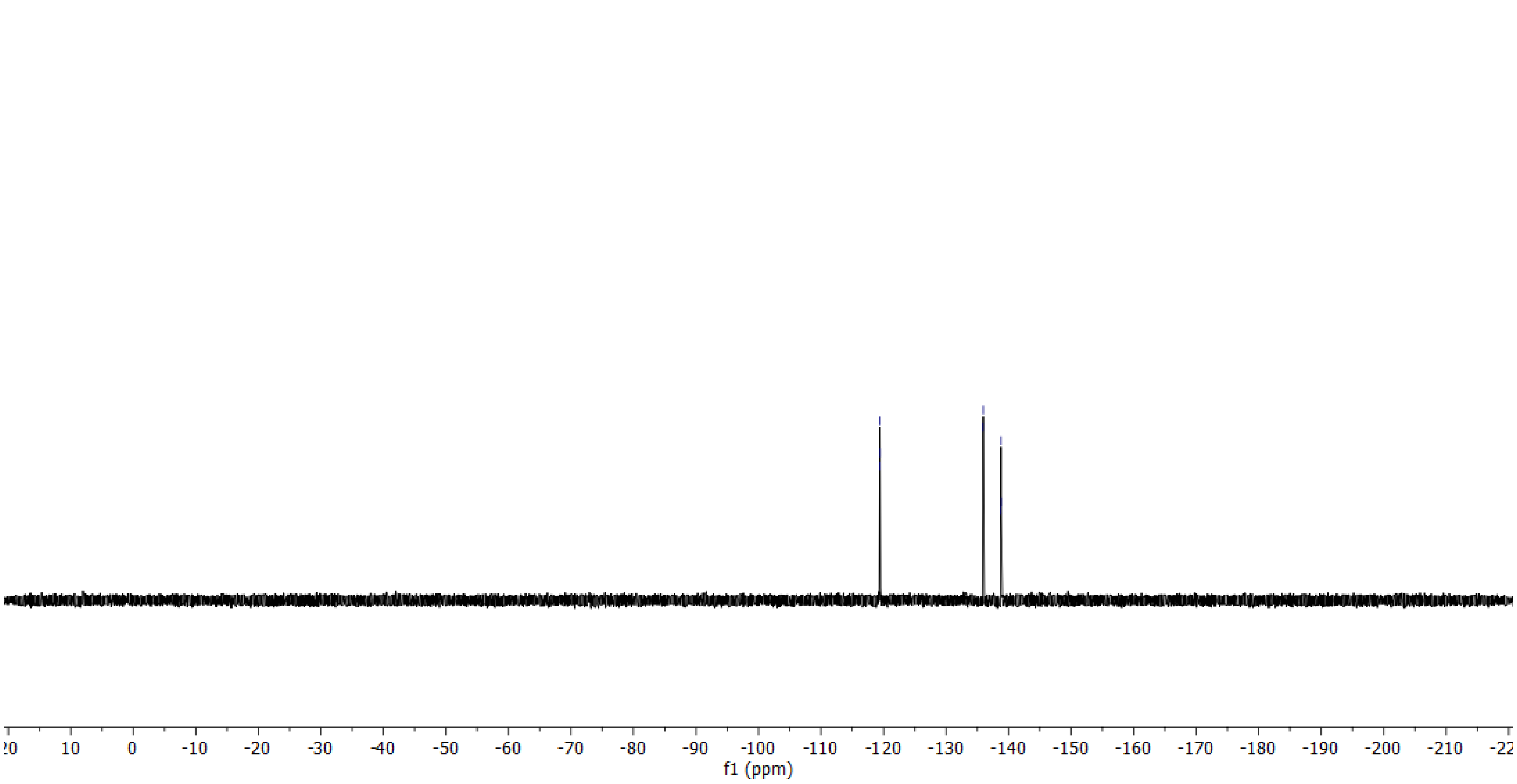

^{1}H NMR of *N*-Boc-2,3,4-trifluoro-6-(methyl)carboxylbenzylamine (26b)

$CDCl_3$, 23 ºC

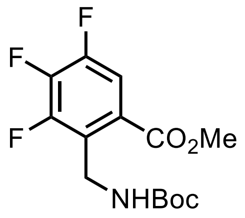

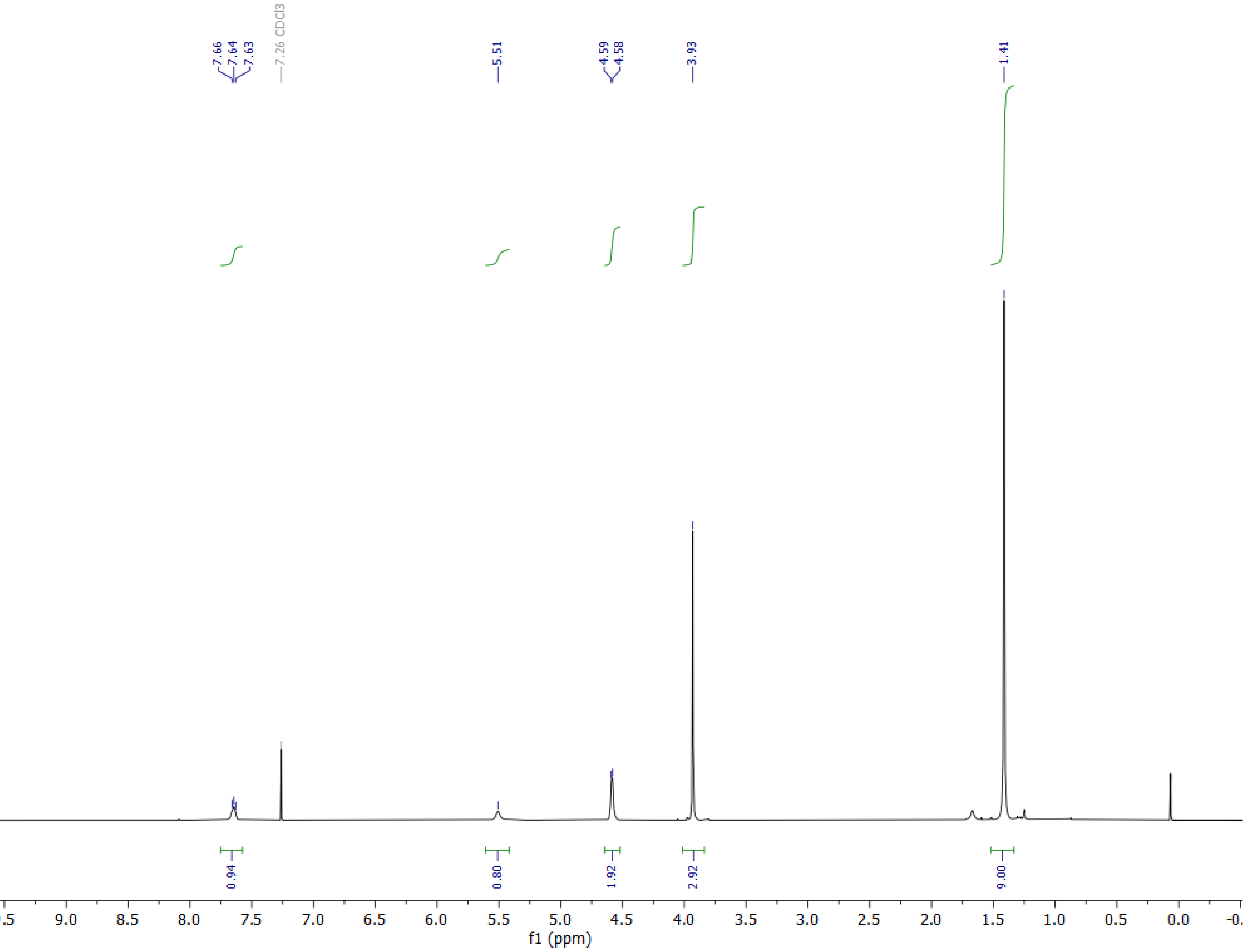

^{13}C NMR of *N*-Boc-2,3,4-trifluoro-6-(methyl)carboxylbenzylamine (26b)

$CDCl_3$, 23 ºC

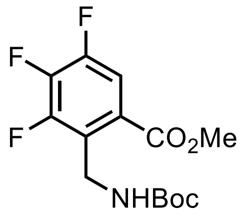

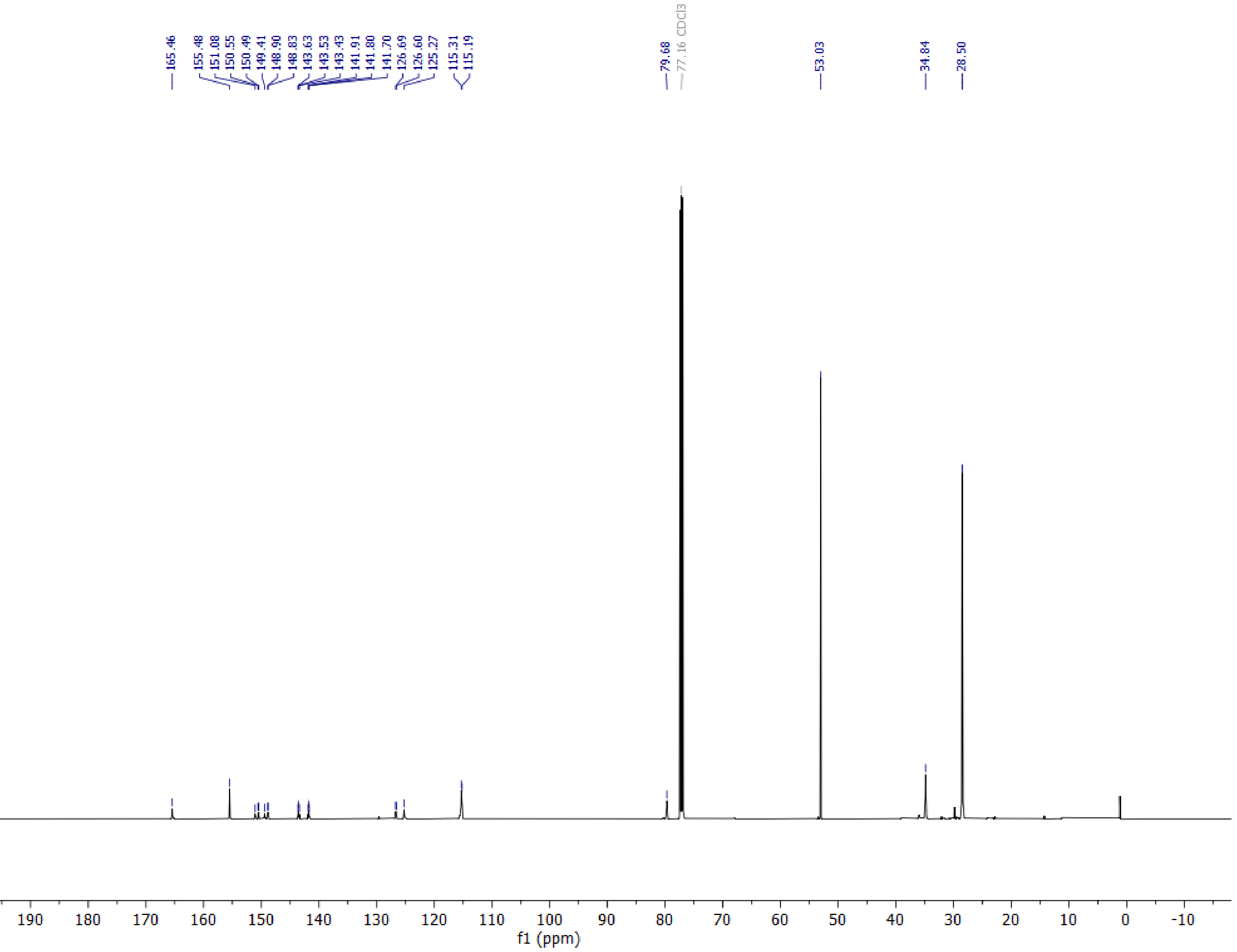

^{19}F NMR of *N*-Boc-2,3,4-trifluoro-6-(methyl)carboxylbenzylamine (26b)

$CDCl_3$, 23 ºC

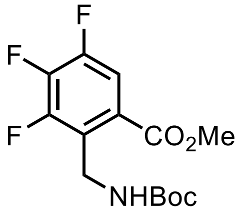

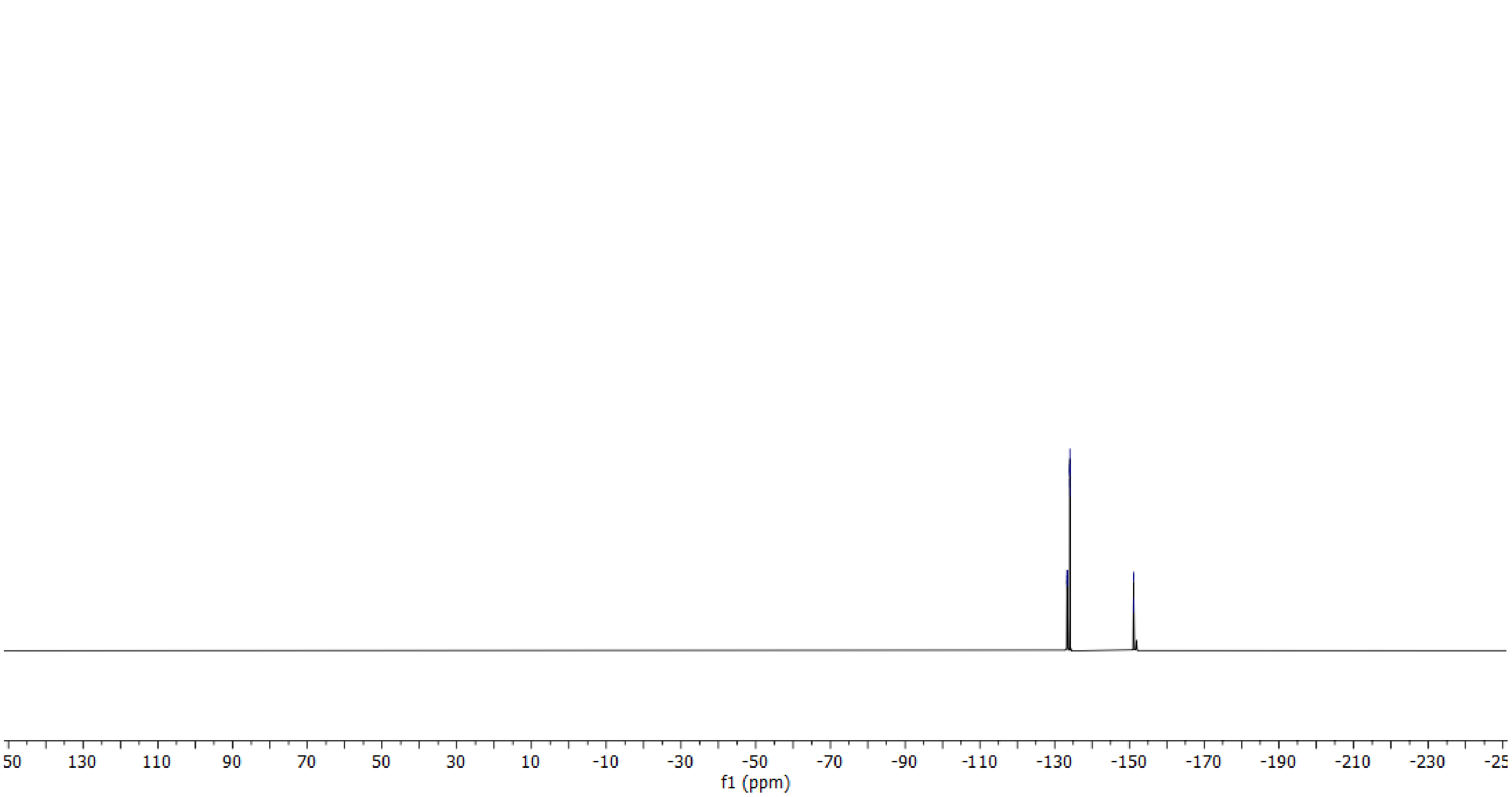

^{1}H NMR of *N*-Boc-2,3,6-trifluoro-5-(methyl)carboxylbenzylamine (26c)

$CDCl_3$, 23 ºC

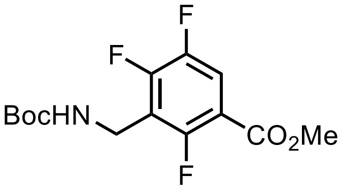

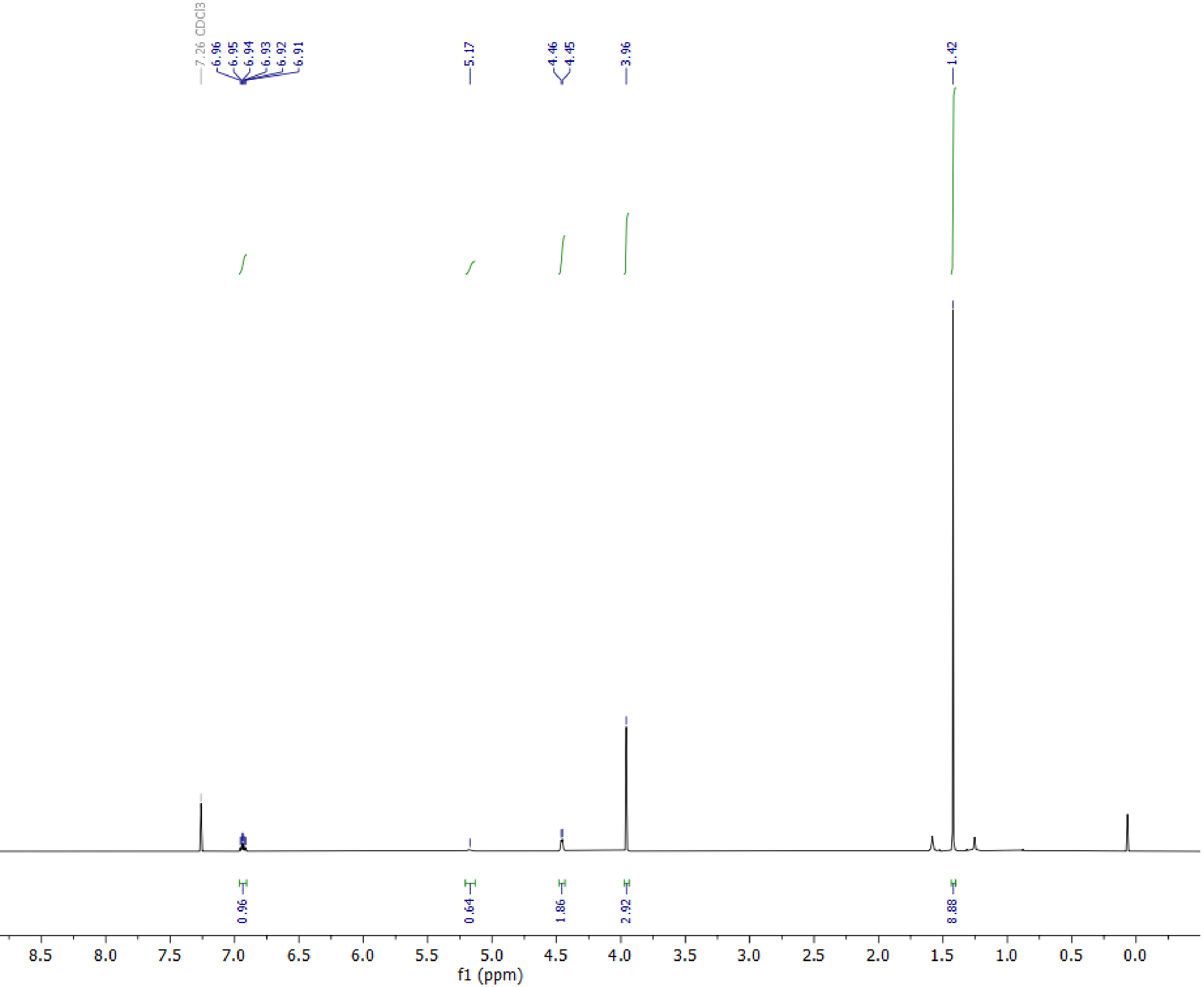

^{13}C NMR of *N*-Boc-2,3,6-trifluoro-5-(methyl)carboxylbenzylamine (26c)

$CDCl_3$, 23 ºC

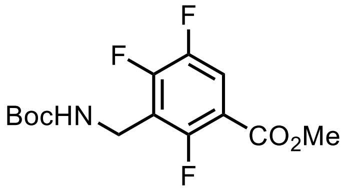

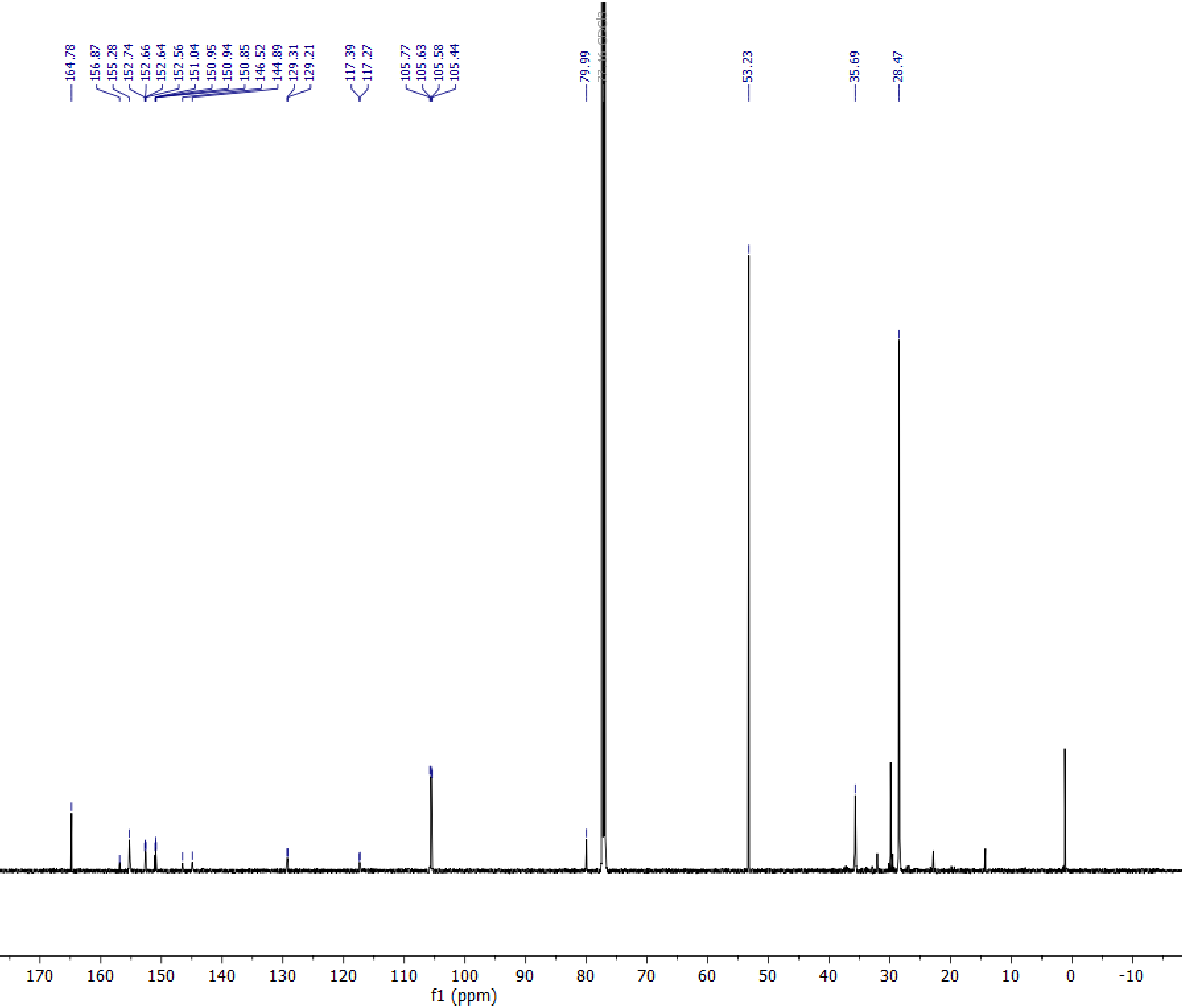

^{19}F NMR of *N*-Boc-2,3,6-trifluoro-5-(methyl)carboxylbenzylamine (26c)

$CDCl_3$, 23 ºC

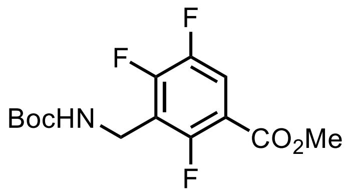

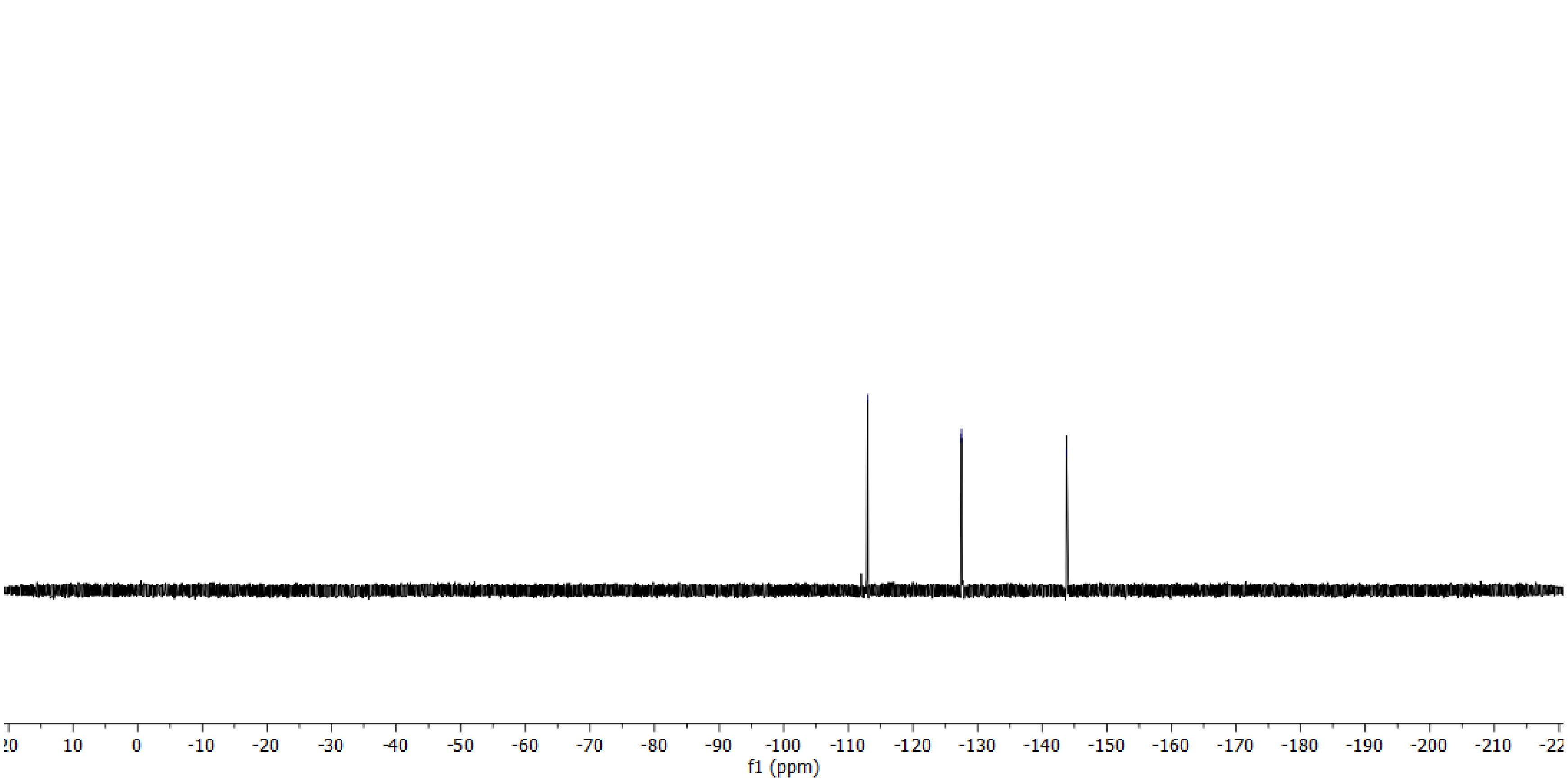

^{1}H NMR of *N*-Boc-2,3,6-trifluorobenzylamine (27)

$CDCl_3$, 23 ºC

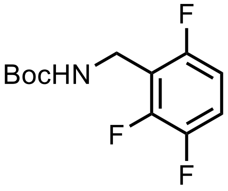

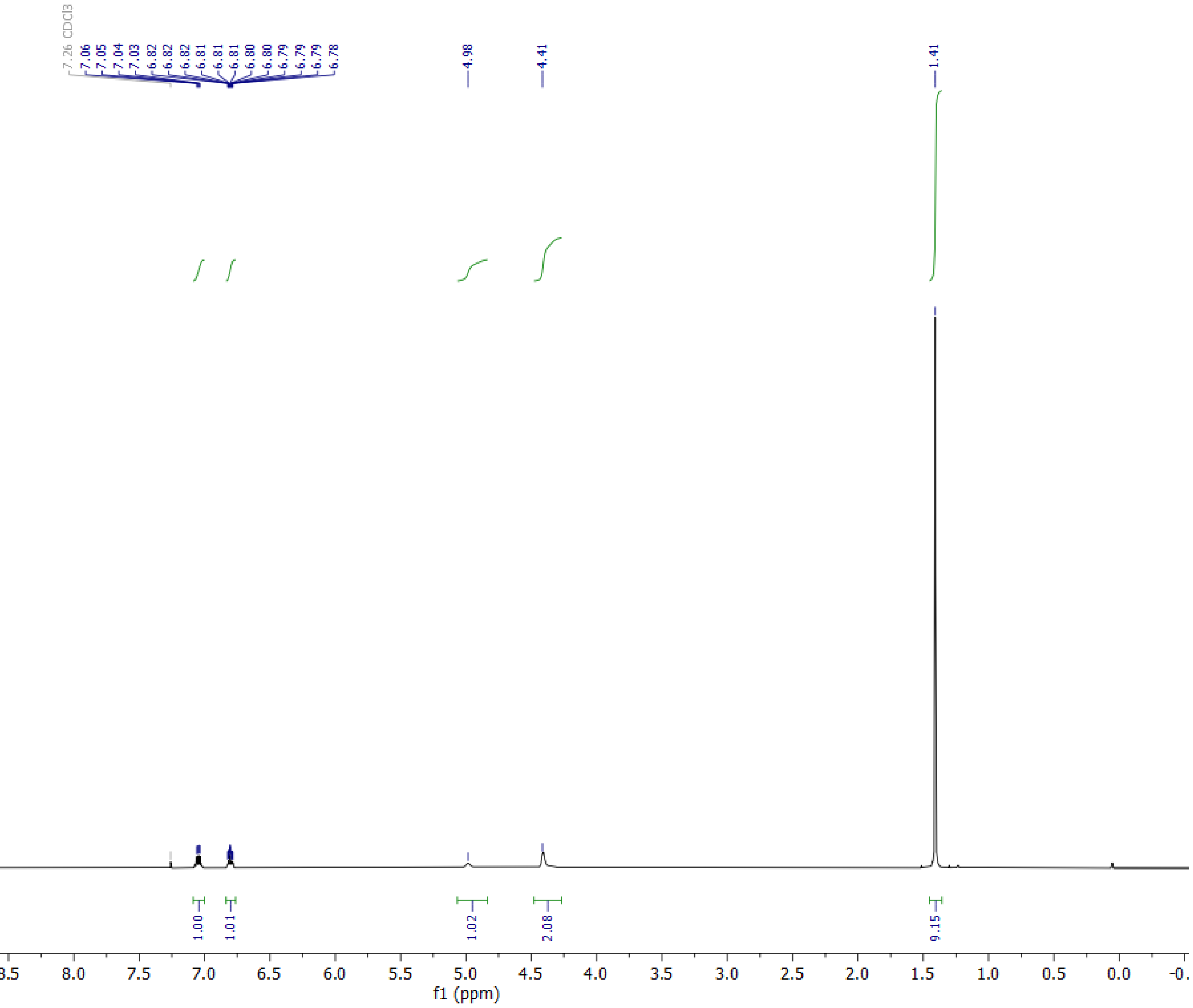

^{13}C NMR of *N*-Boc-2,3,6-trifluorobenzylamine (27)

$CDCl_3$, 23 ºC

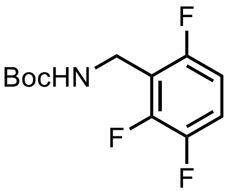

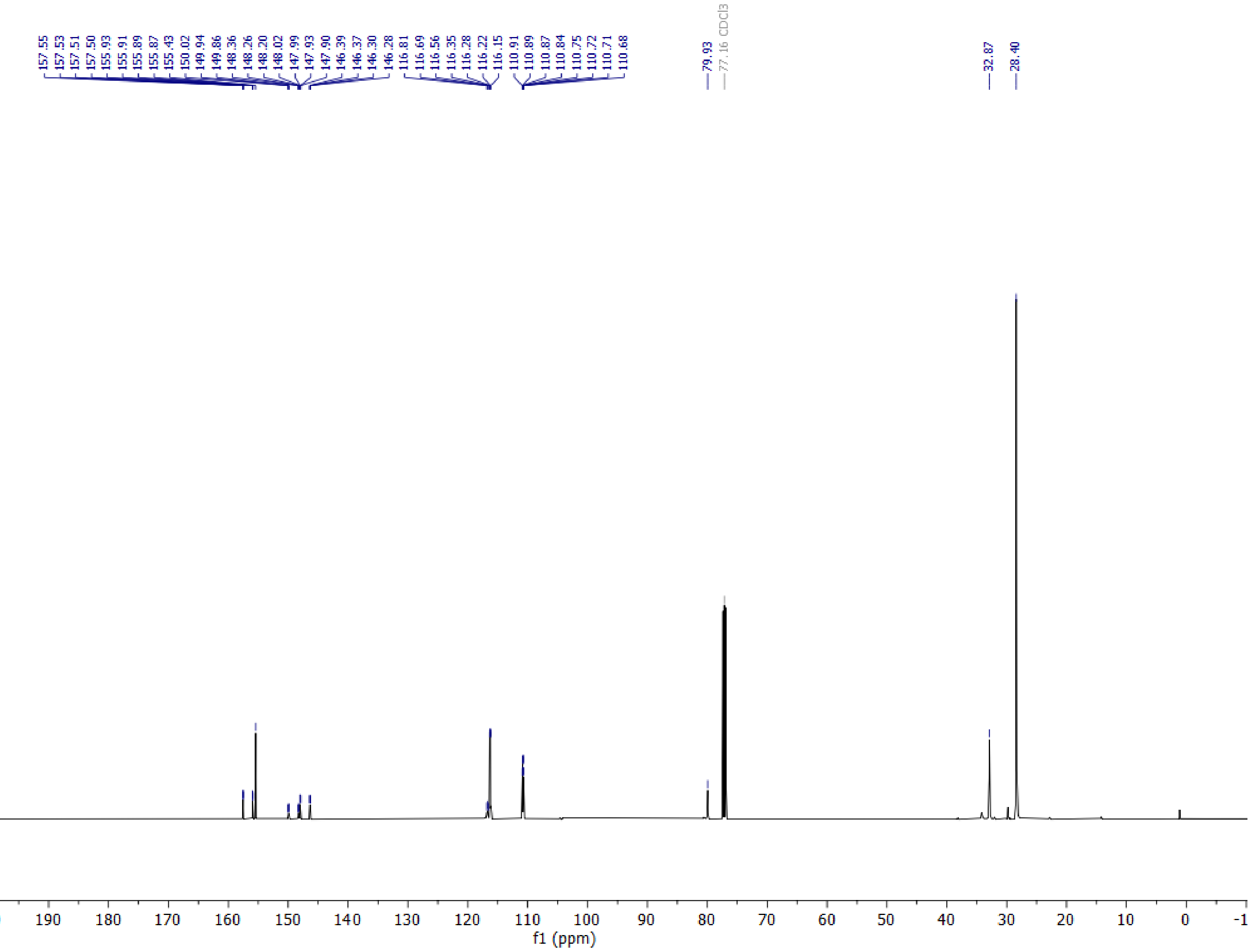

^{19}F NMR of *N*-Boc-2,3,6-trifluorobenzylamine (27)

$CDCl_3$, 23 ºC

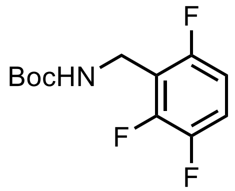

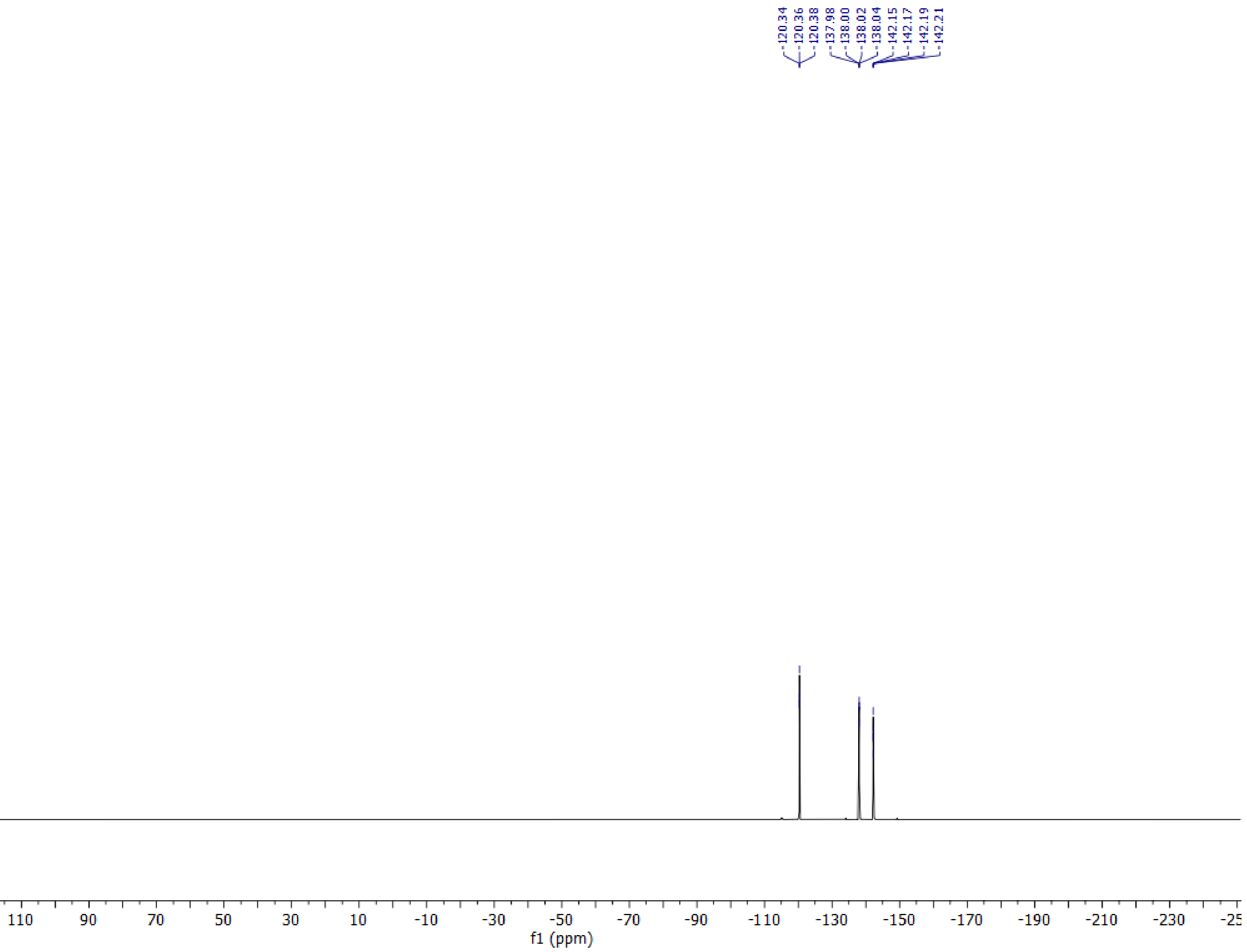

^{1}H NMR of *N*-Boc-2,4,6-trichloro-3,5-difluorobenzylamine (28)

$CDCl_3$, 23 ºC

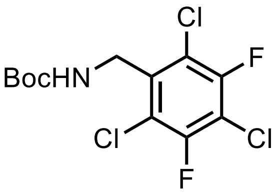

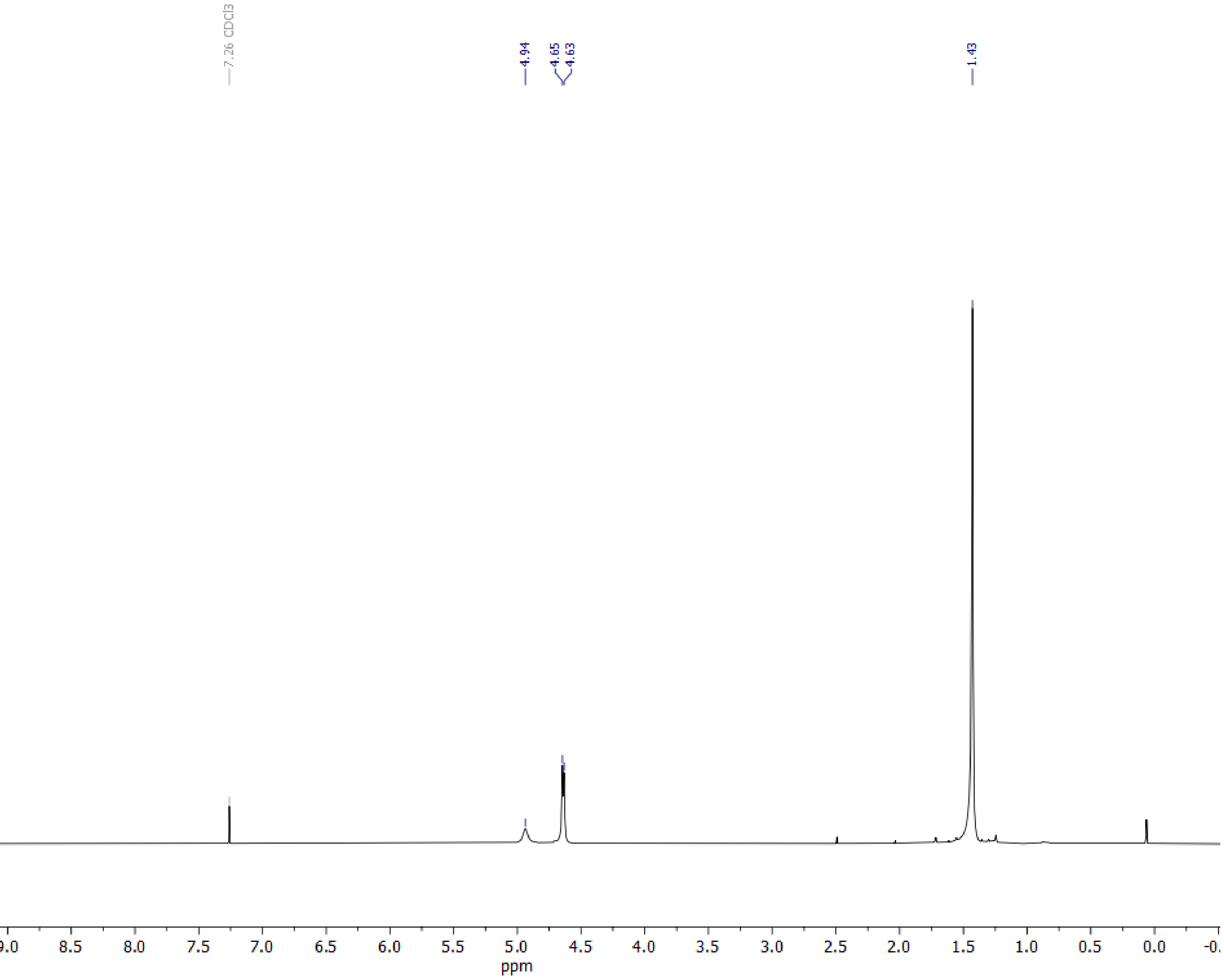

^{13}C NMR of *N*-Boc-2,4,6-trichloro-3,5-difluorobenzylamine (28)

$CDCl_3$, 23 ºC

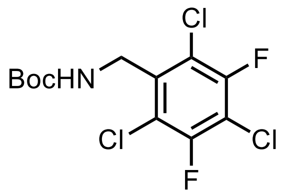

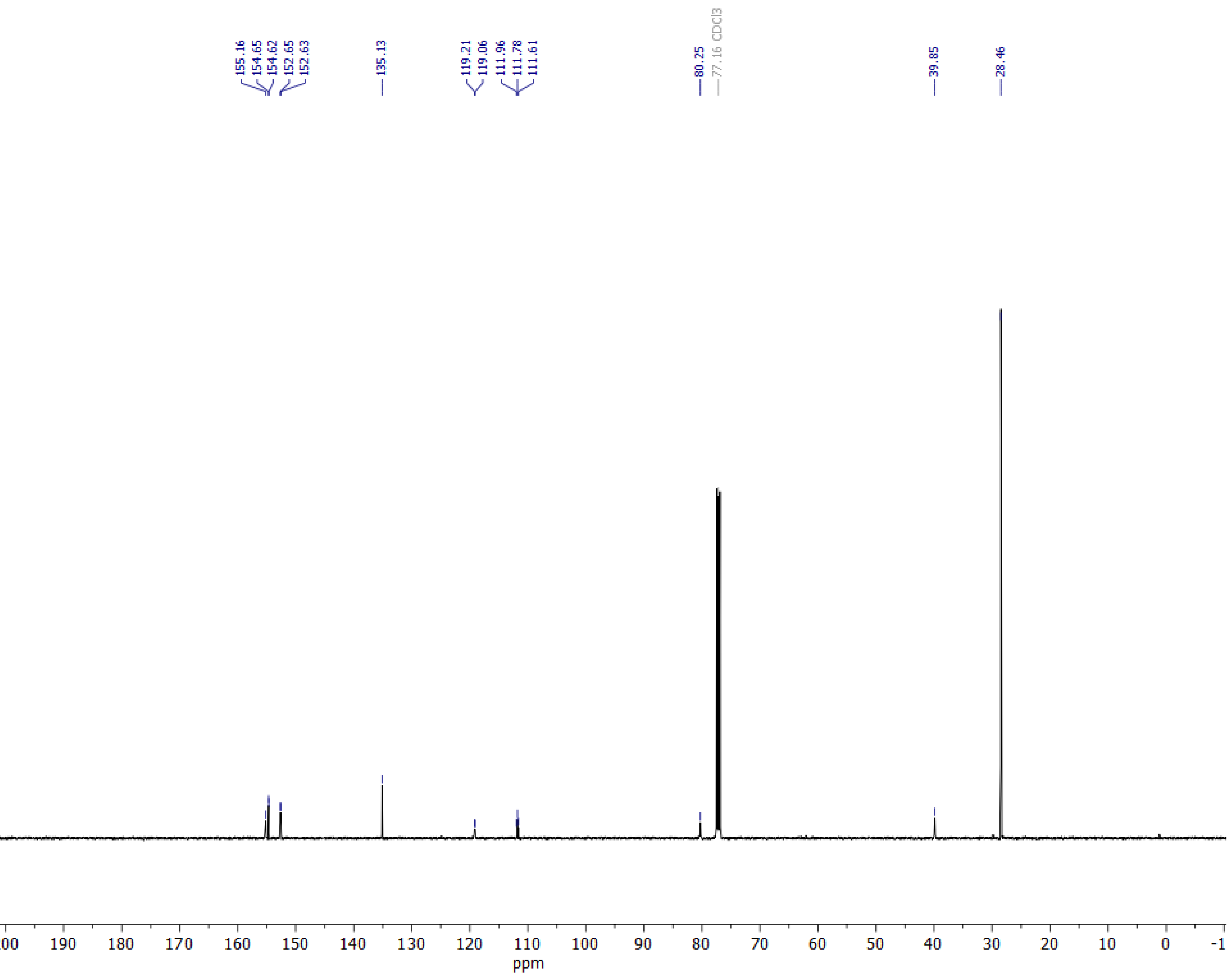

^{19}F NMR of *N*-Boc-2,4,6-trichloro-3,5-difluorobenzylamine (28)

$CDCl_3$, 23 ºC

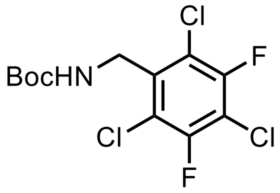

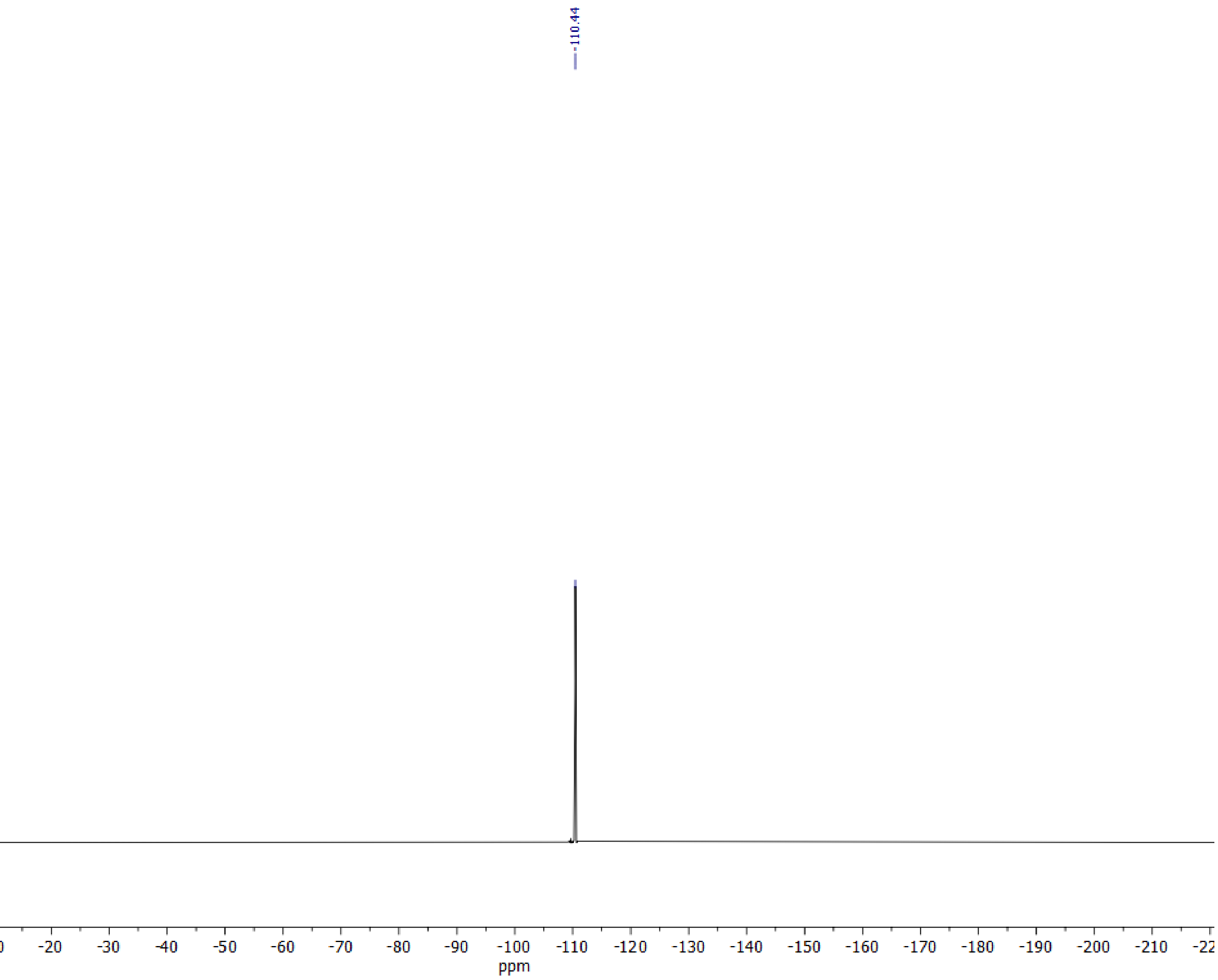

^{1}H NMR of *N*-Boc-4-trifluoromethyl-2,6-difluorobenzylamine (29)

$CDCl_3$, 23 ºC

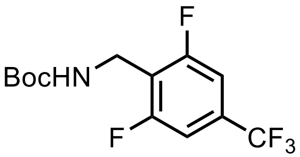

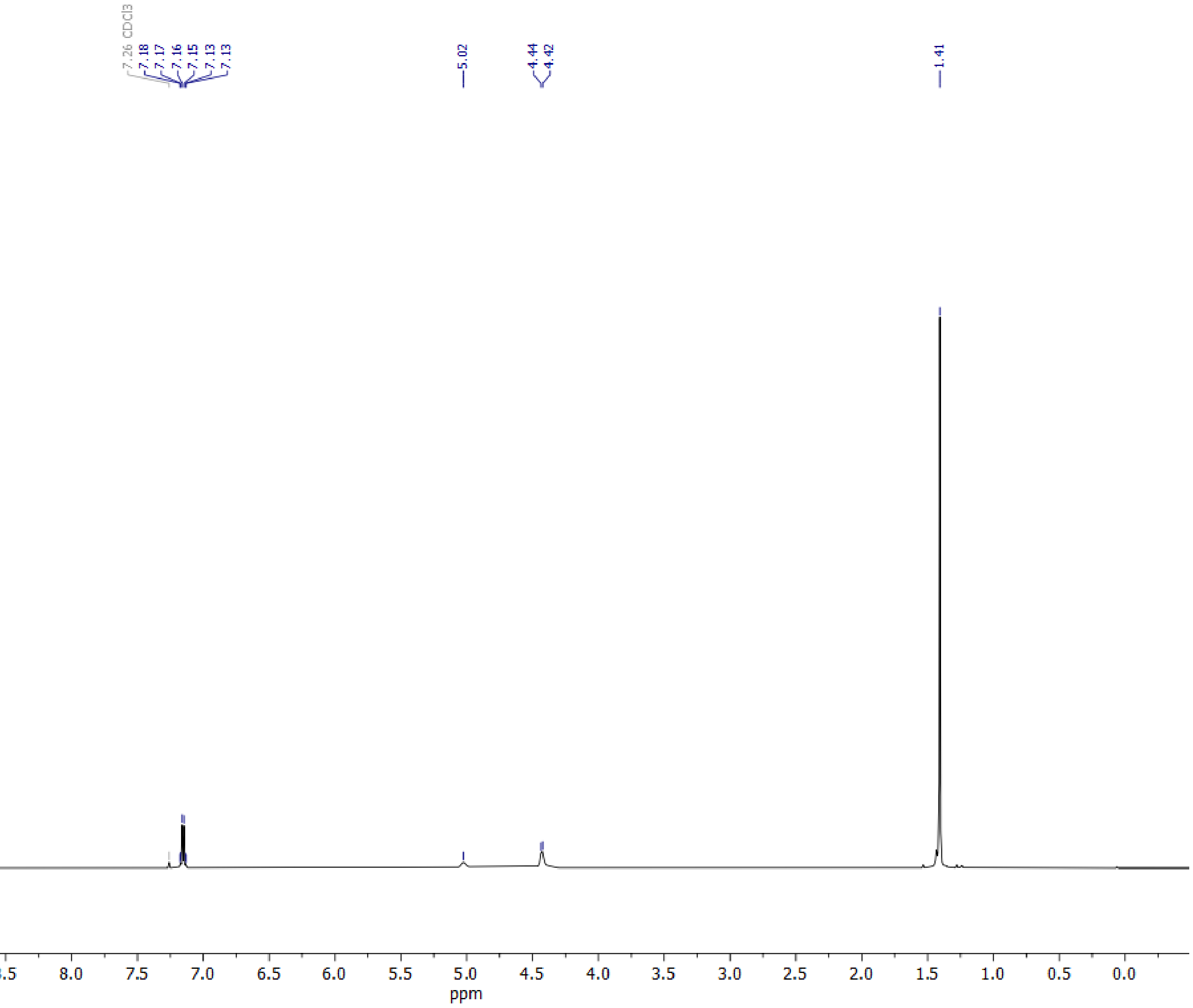

^{13}C NMR of *N*-Boc-4-trifluoromethyl-2,6-difluorobenzylamine (29)

$CDCl_3$, 23 ºC

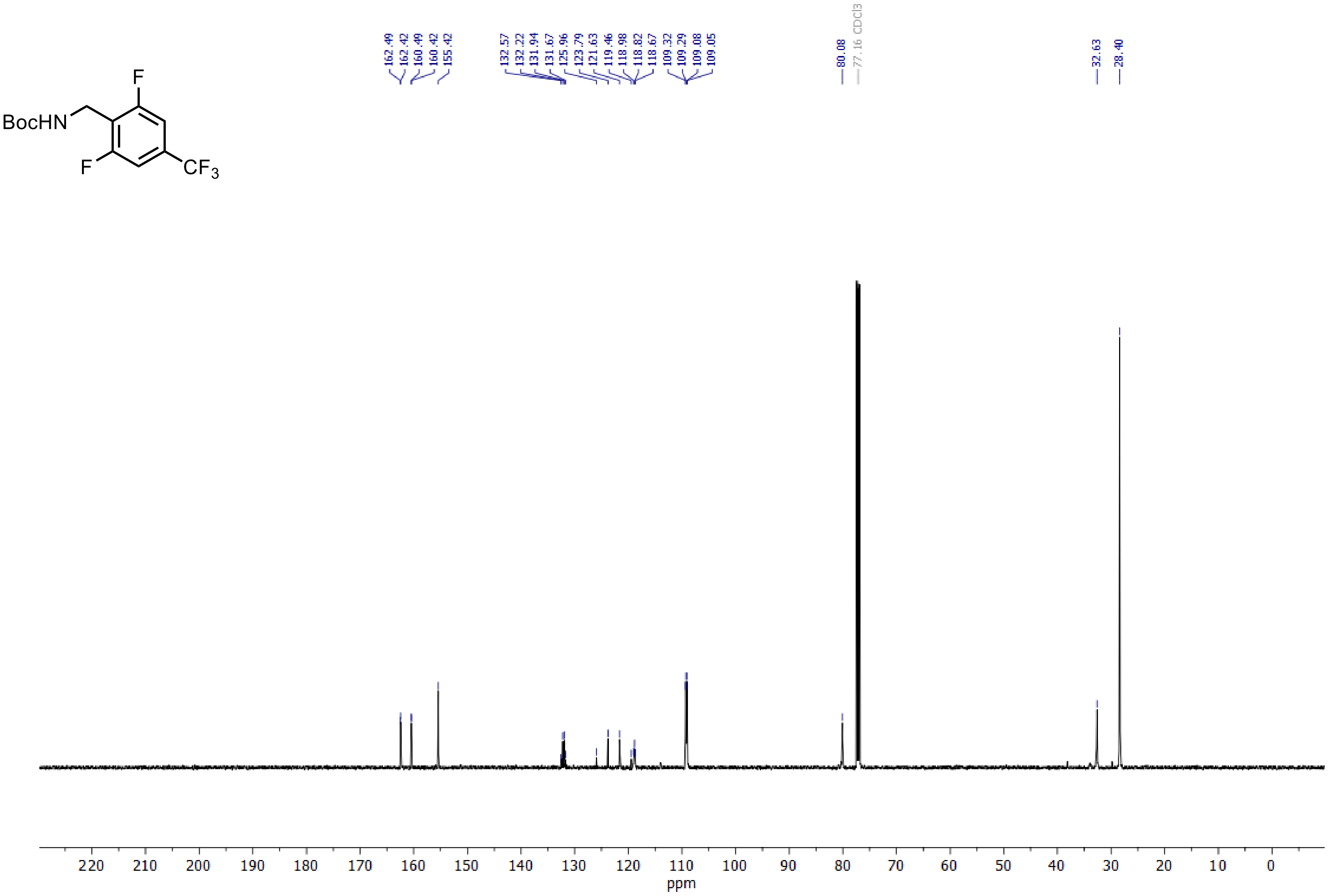

^{19}F NMR of *N*-Boc-4-trifluoromethyl-2,6-difluorobenzylamine (29)

$CDCl_3$, 23 ºC

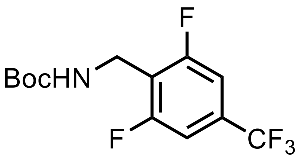

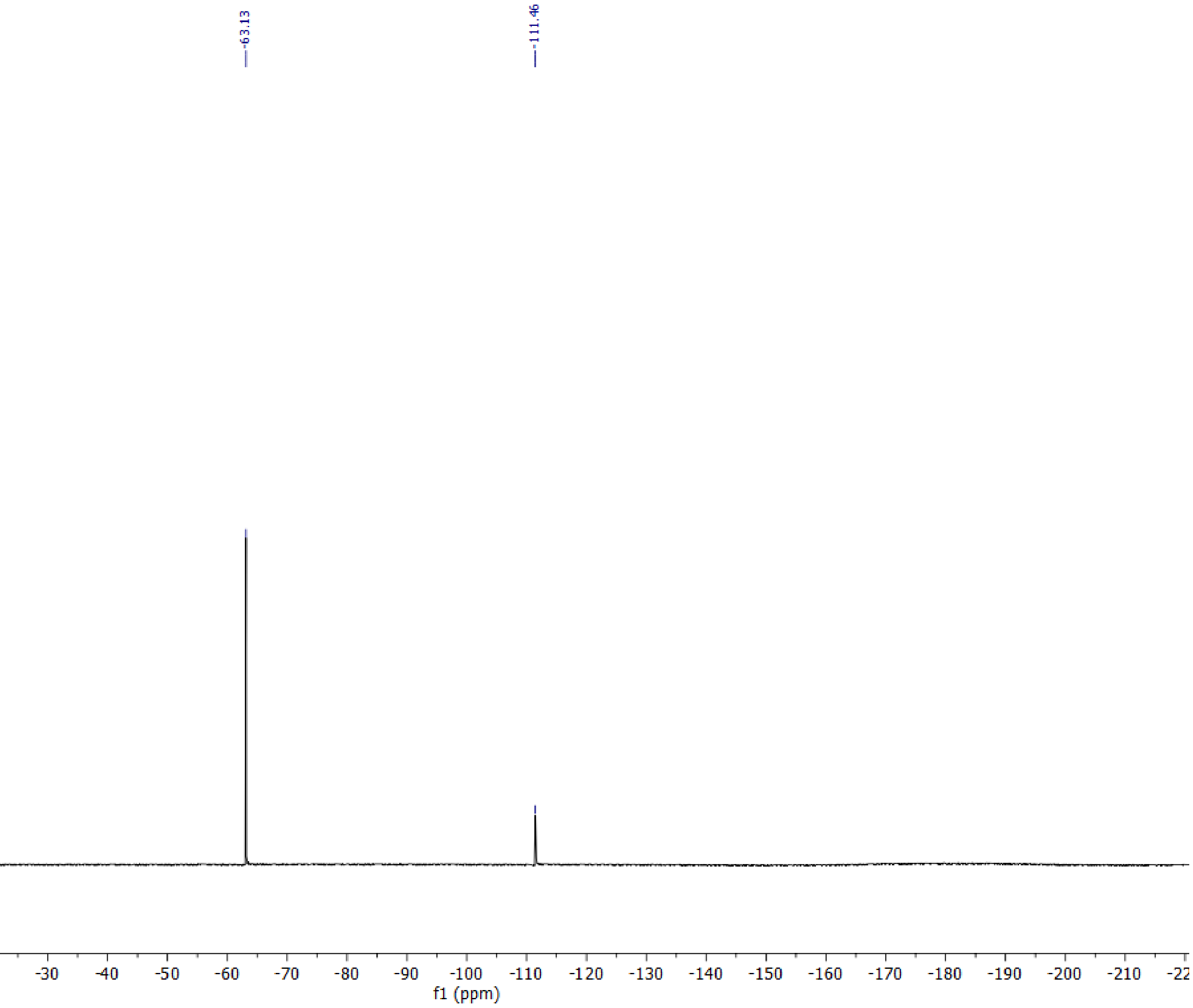

^{1}H NMR of *N*-Boc-4-trifluoromethyl-2,6-dichlorobenzylamine (30)

$CDCl_3$, 23 ºC

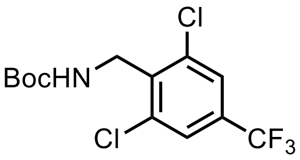

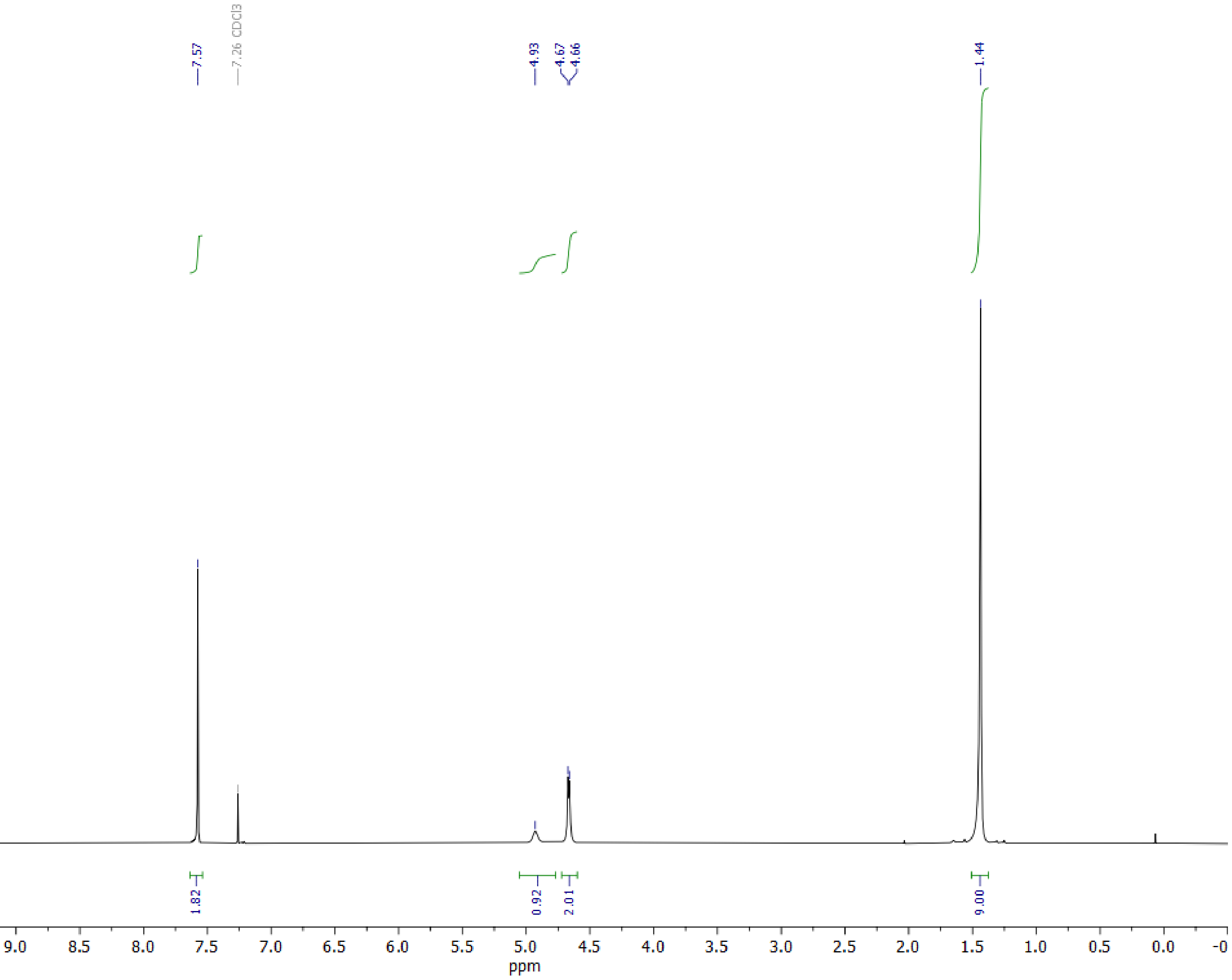

^{13}C NMR of *N*-Boc-4-trifluoromethyl-2,6-dichlorobenzylamine (30)

$CDCl_3$, 23 ºC

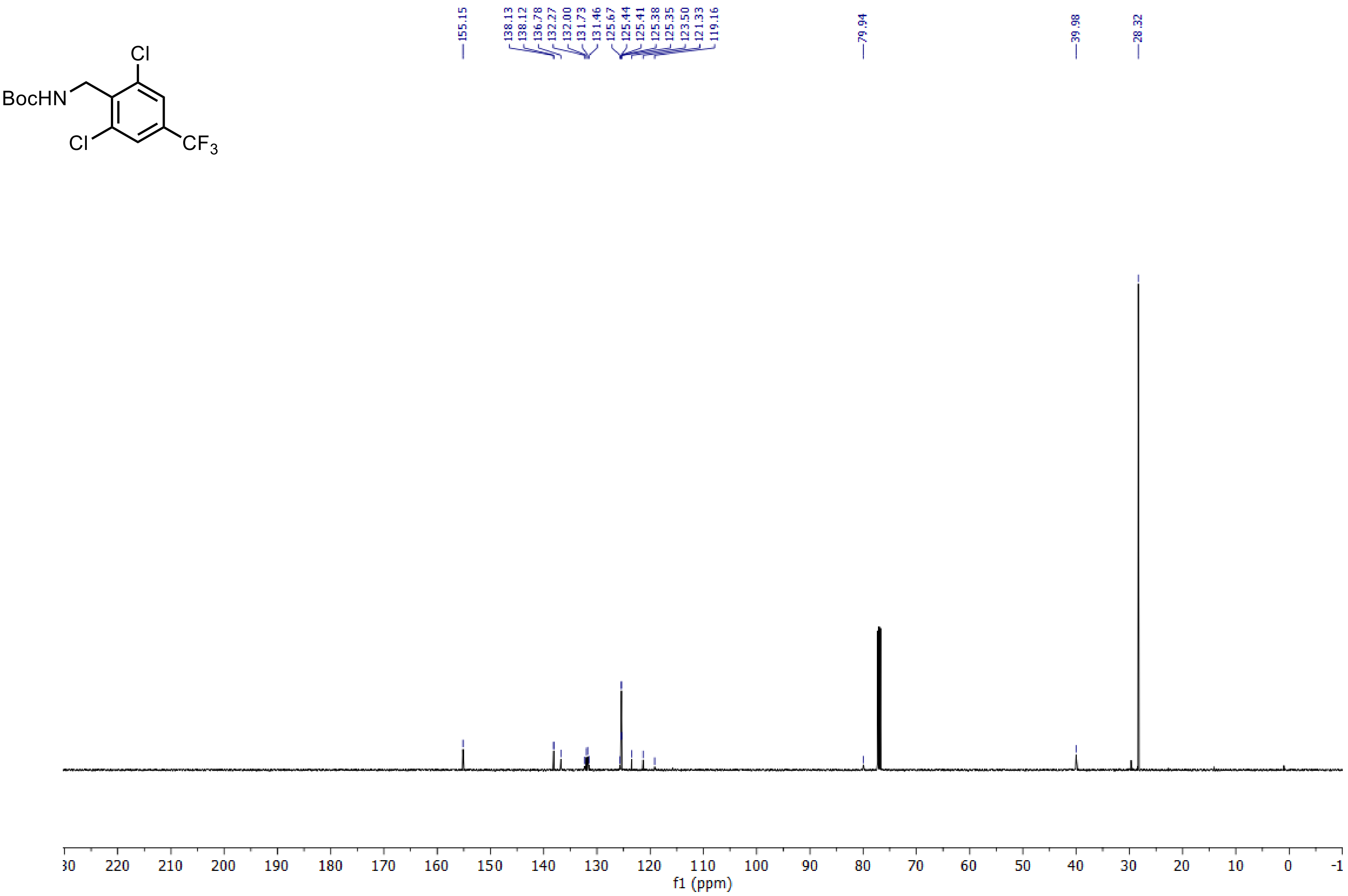

^{19}F NMR of *N*-Boc-4-trifluoromethyl-2,6-dichlorobenzylamine (30)

CDCl$_3$, 23 ºC

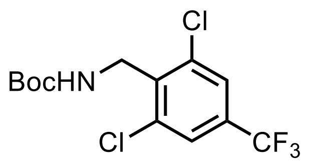

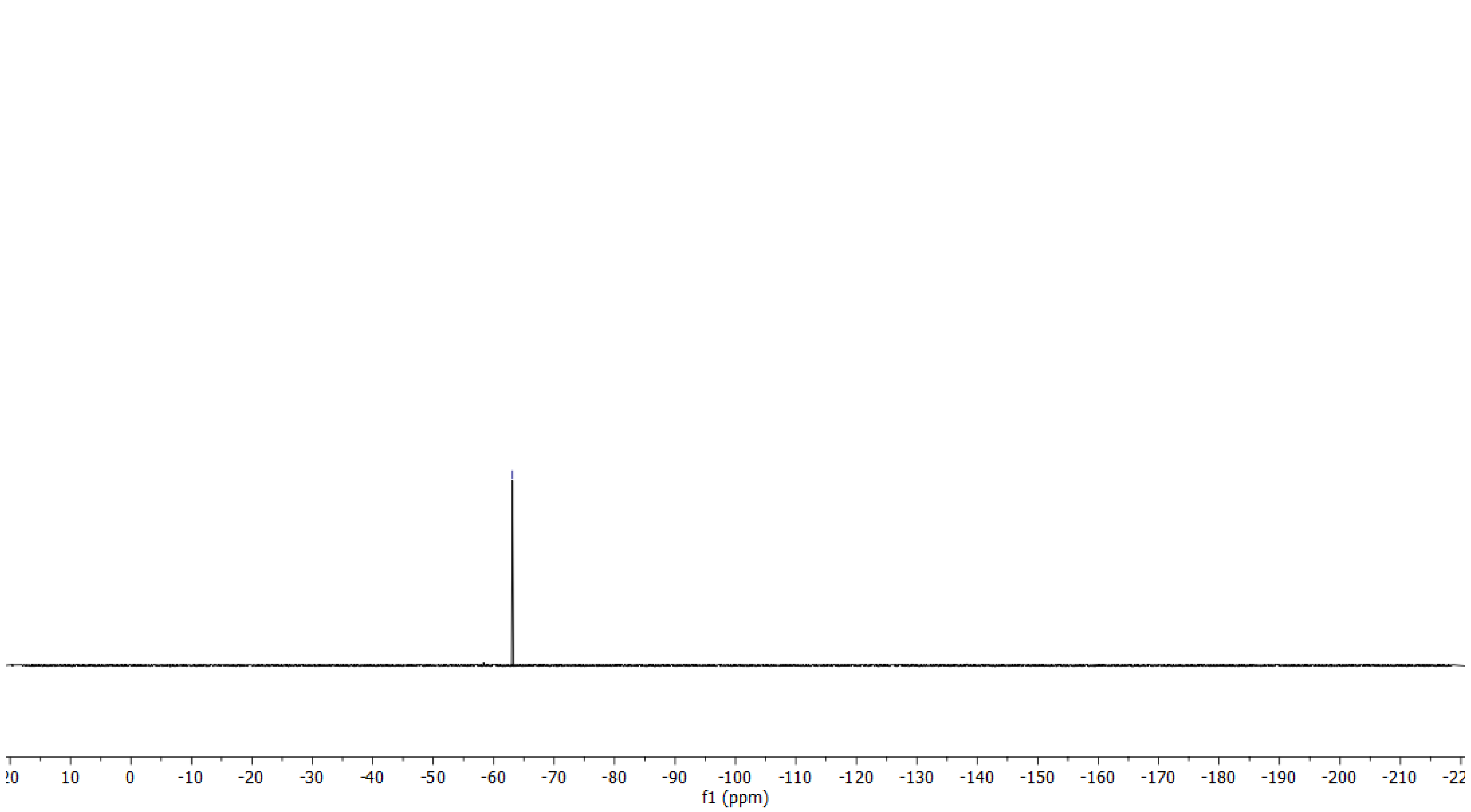

^{1}H NMR of *N*-Boc-4-allyl-2,3,5,6-tetrafluorobenzylamine (31a)

$CDCl_3$, 23 ºC

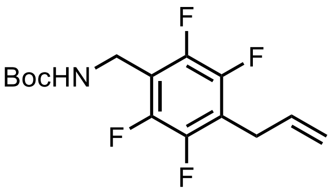

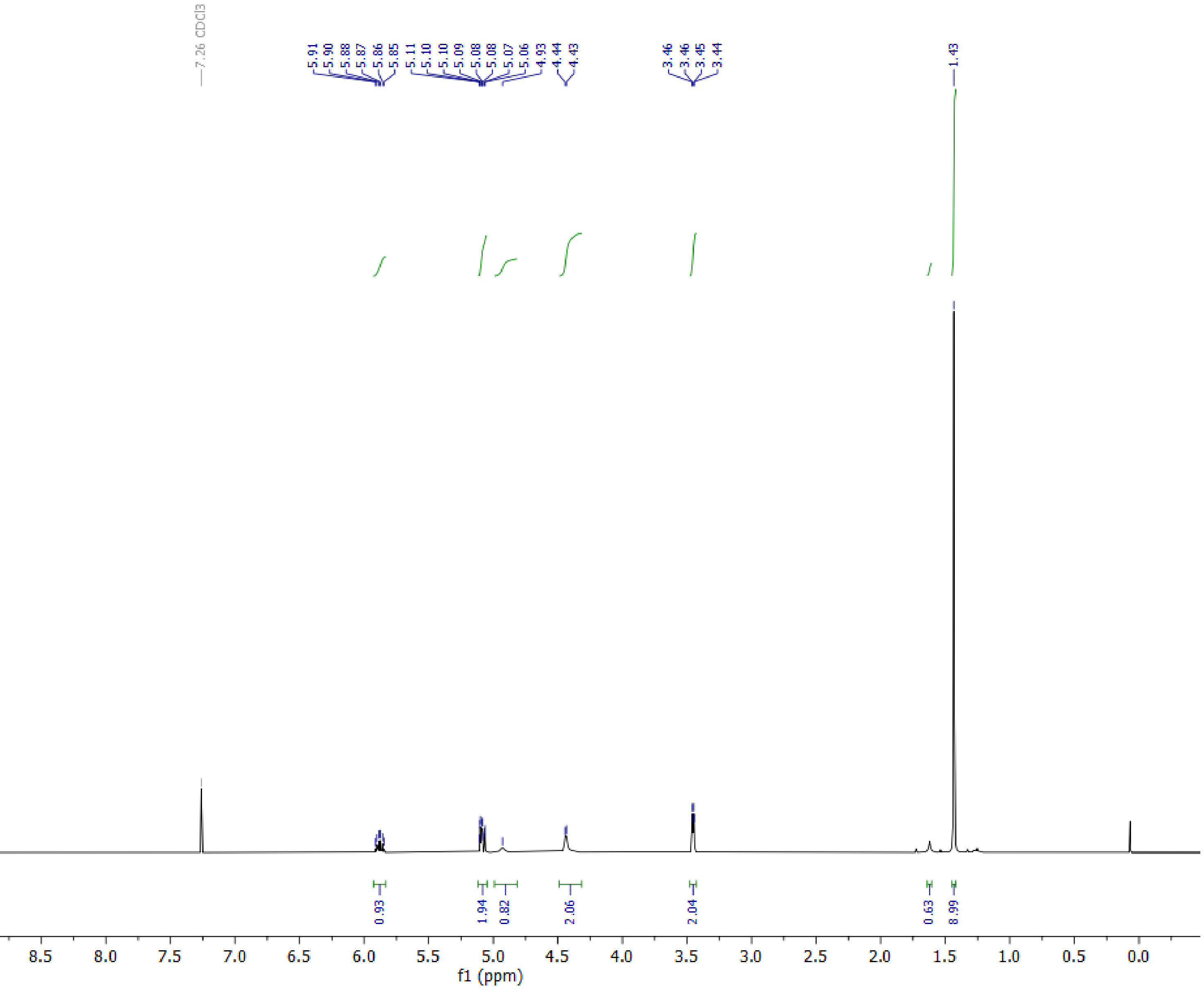

^{13}C NMR of *N*-Boc-4-allyl-2,3,5,6-tetrafluorobenzylamine (31a)

$CDCl_3$, 23 ºC

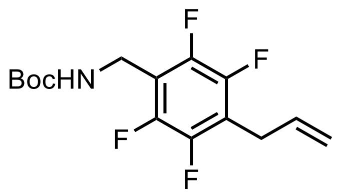

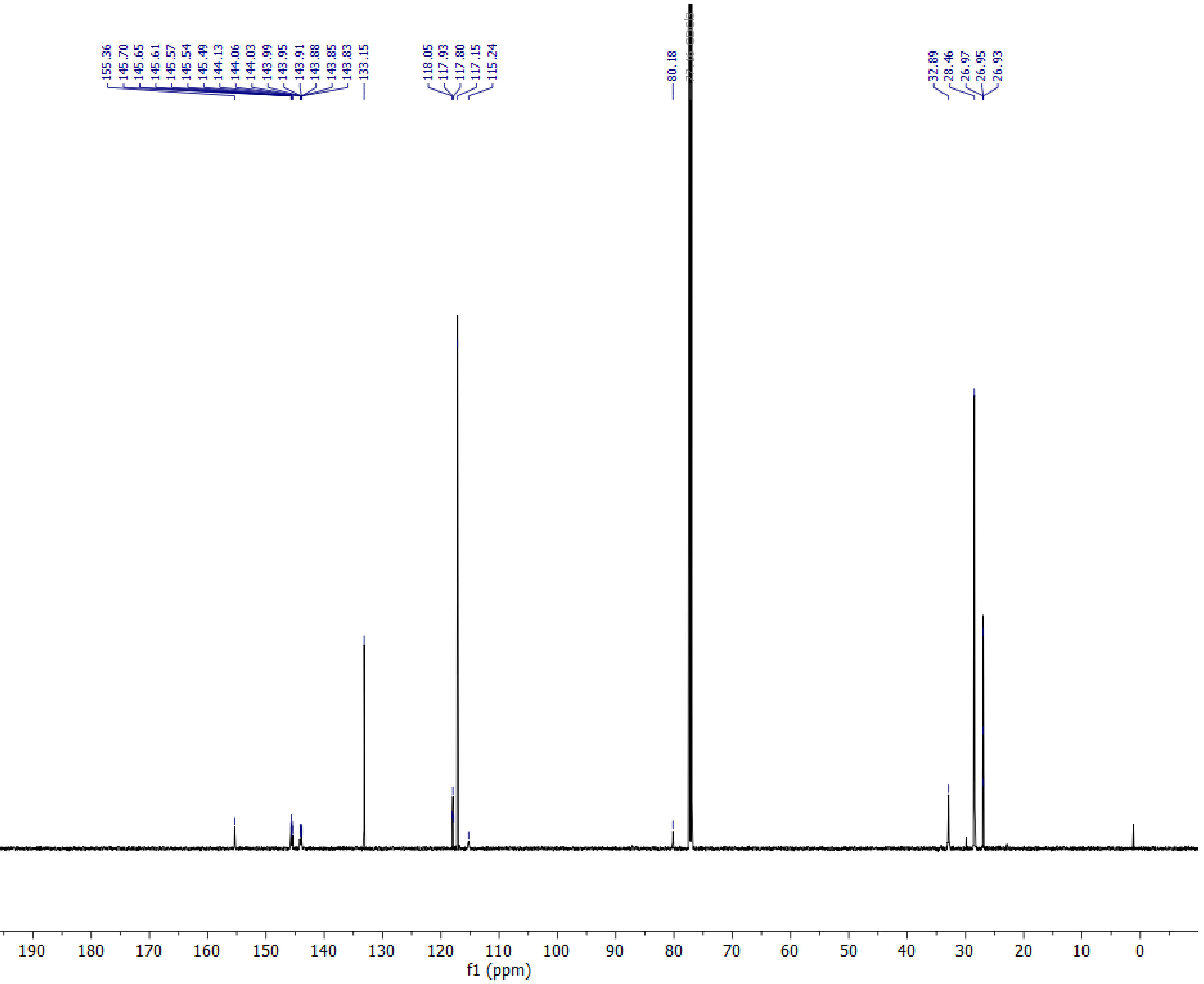

^{19}F NMR of *N*-Boc-4-allyl-2,3,5,6-tetrafluorobenzylamine (31a)

$CDCl_3$, 23 ºC

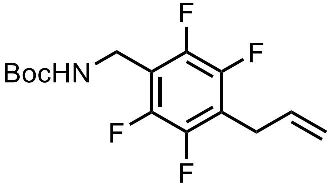

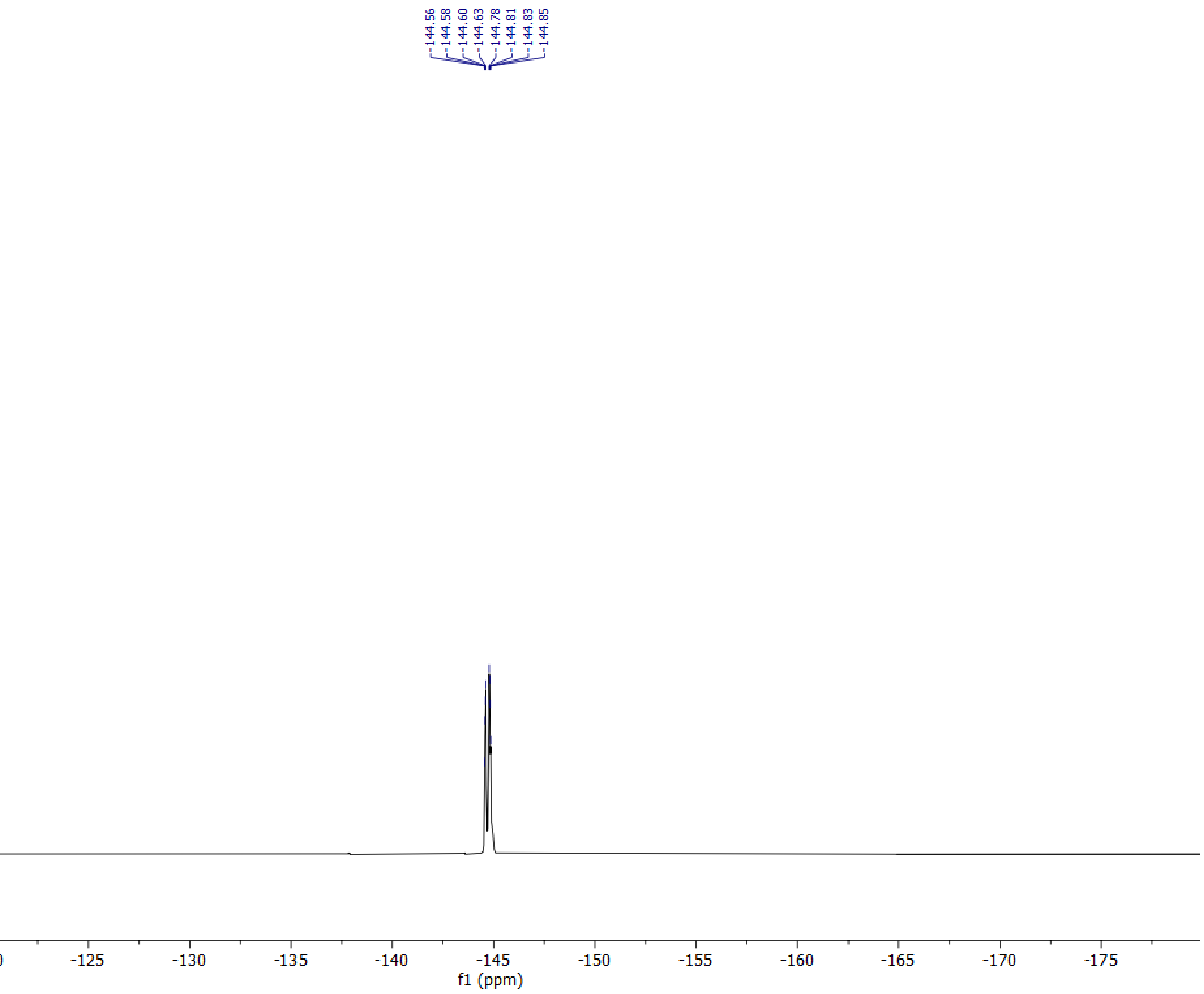

^{1}H NMR of *N*-Boc-5-allyl-2,3,4,6-tetrafluorobenzylamine (31b)

$CDCl_3$, 23 ºC

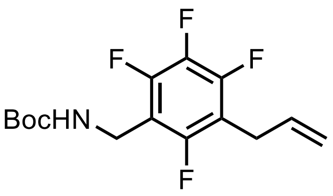

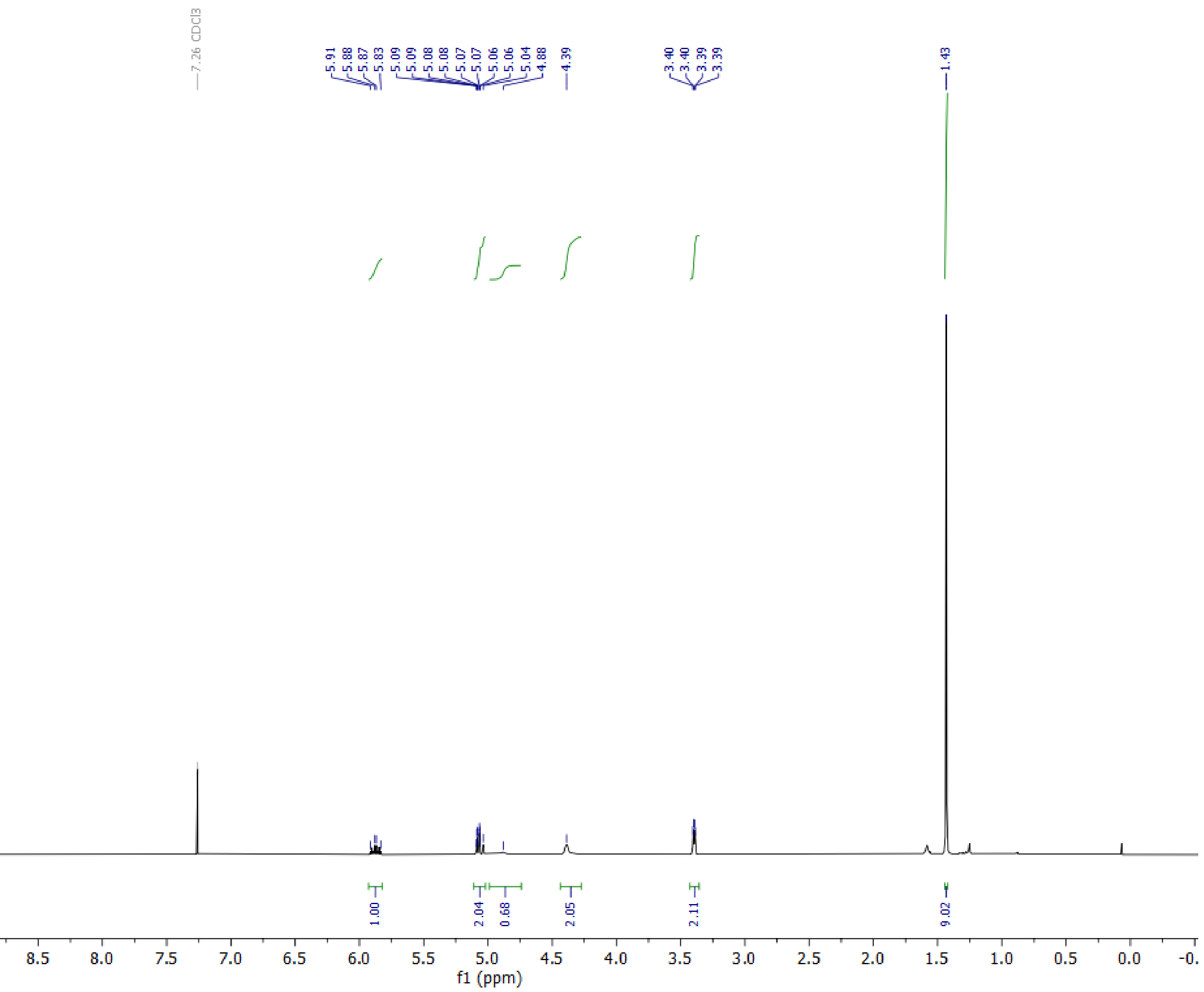

^{13}C NMR of *N*-Boc-5-allyl-2,3,4,6-tetrafluorobenzylamine (31b)

$CDCl_3$, 23 ºC

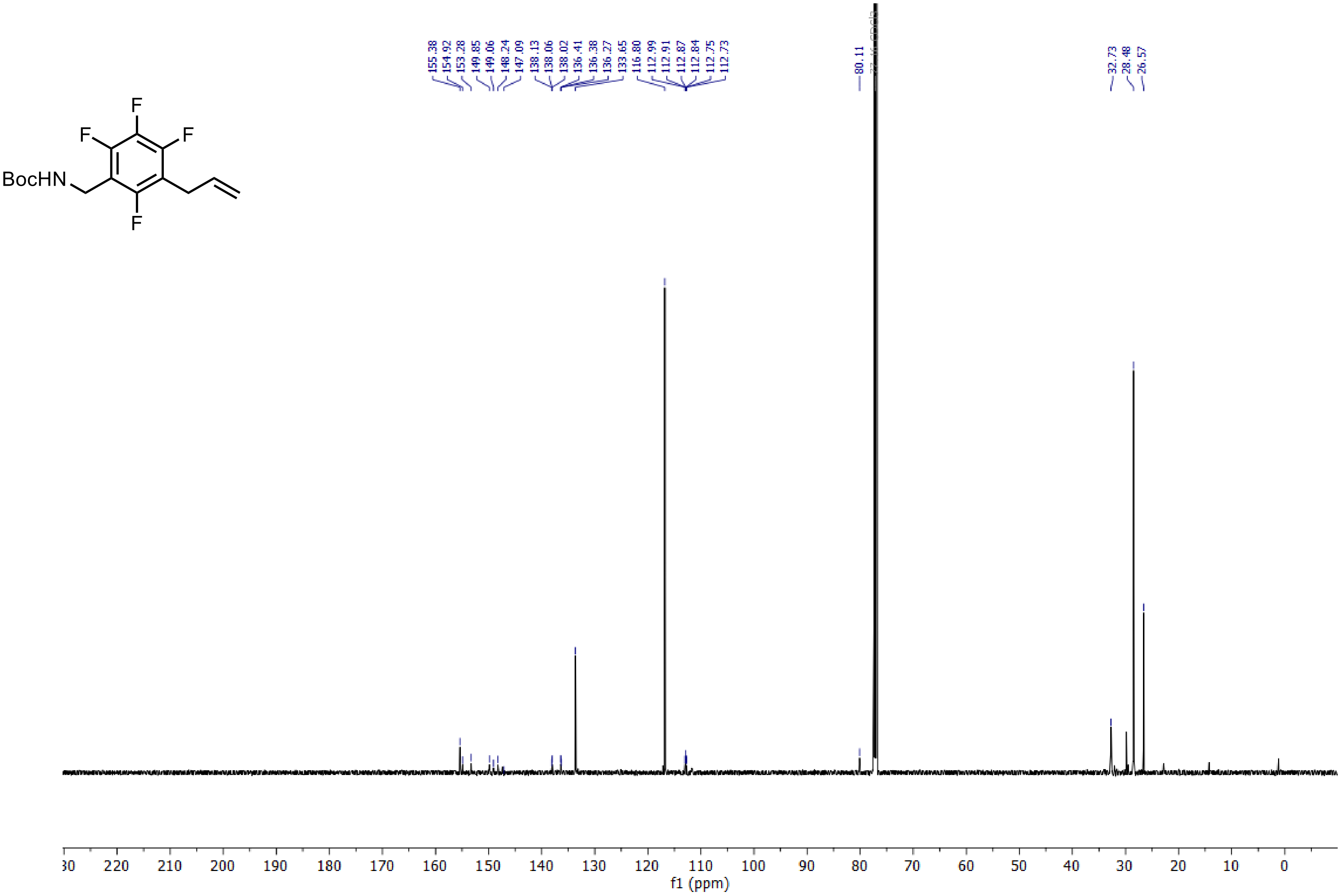

^{19}F NMR of *N*-Boc-5-allyl-2,3,4,6-tetrafluorobenzylamine (31b)

$CDCl_3$, 23 ºC

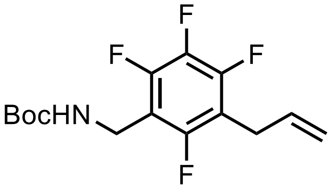

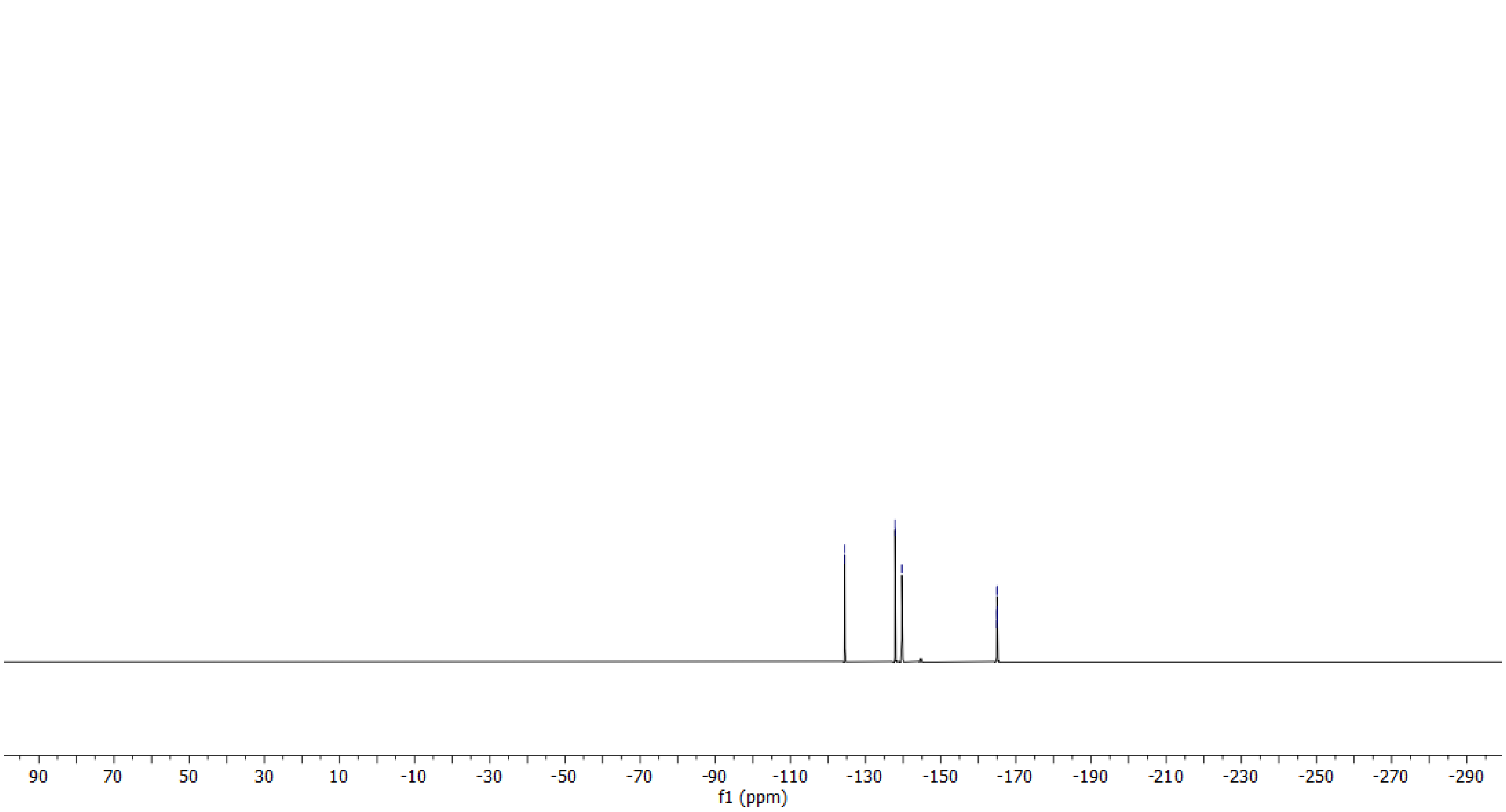

^{1}H NMR of *N*-Boc-6-allyl-2,3,4,5-tetrafluorobenzylamine (31c)

$CDCl_3$, 23 ºC

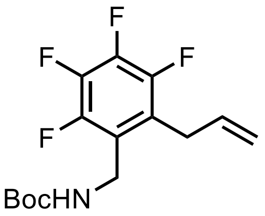

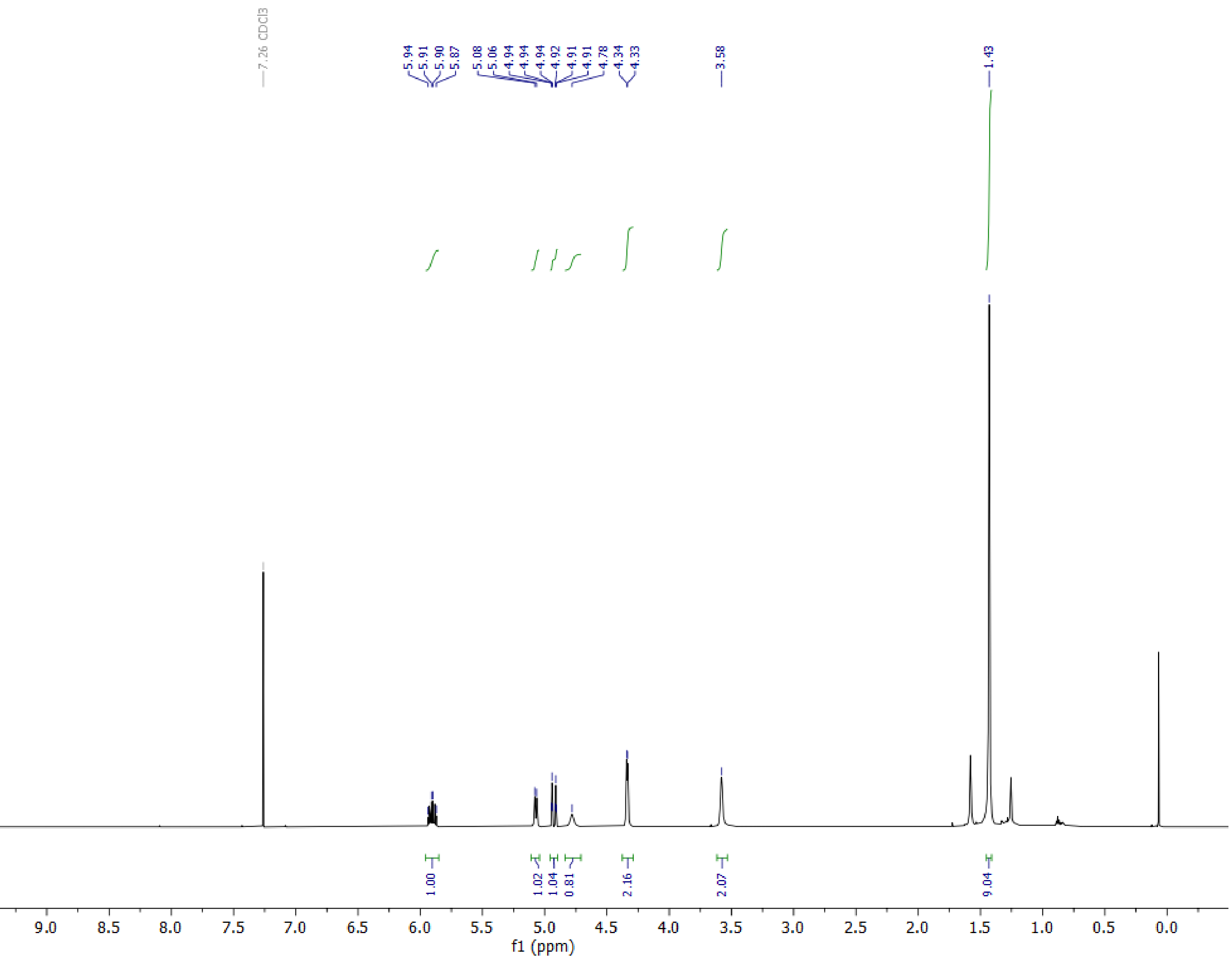

^{13}C NMR of *N*-Boc-6-allyl-2,3,4,5-tetrafluorobenzylamine (31c)

$CDCl_3$, 23 ºC

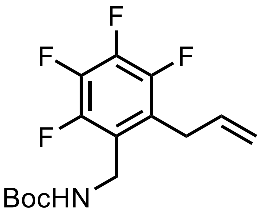

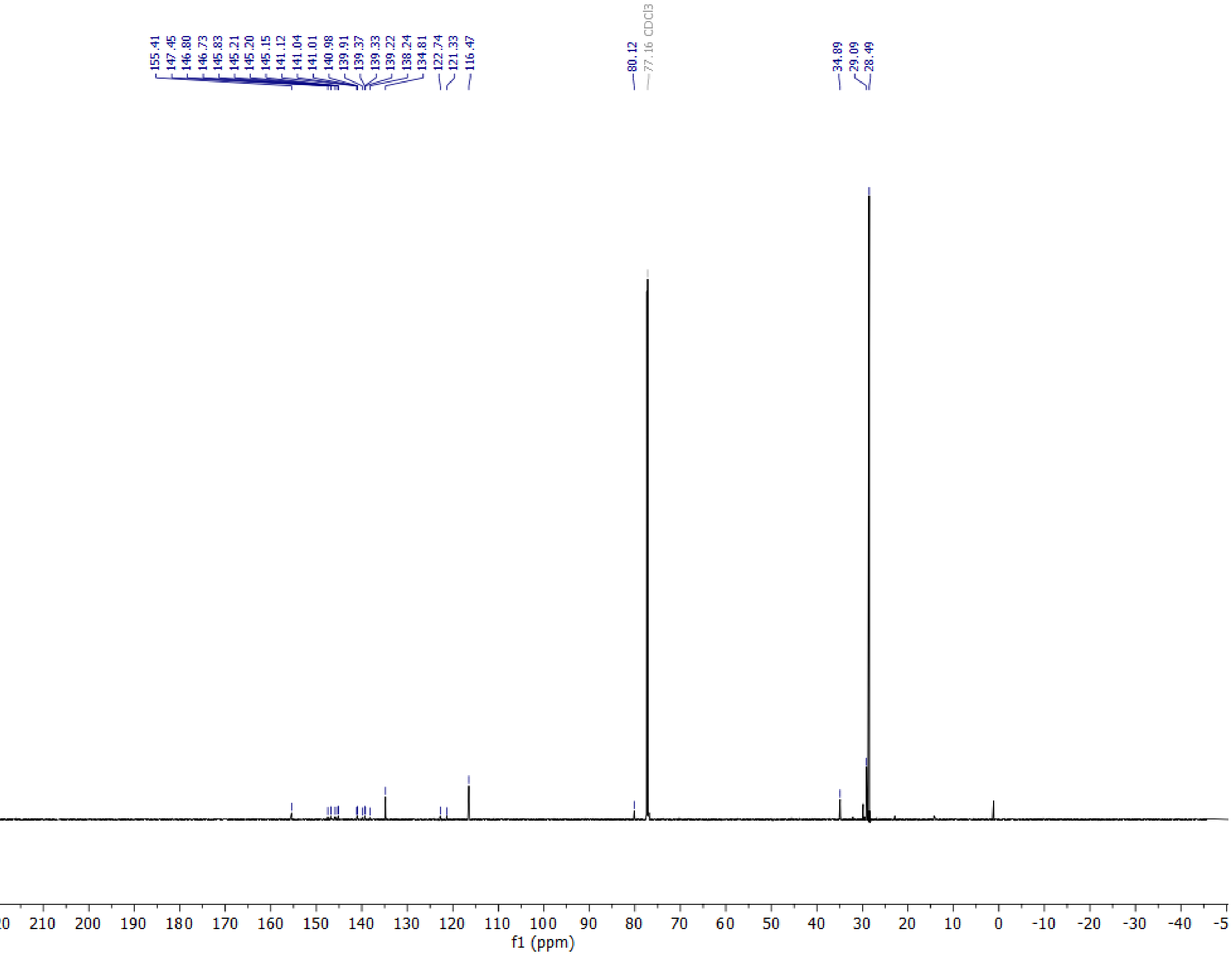

^{19}F NMR of *N*-Boc-6-allyl-2,3,4,5-tetrafluorobenzylamine (31c)

$CDCl_3$, 23 ºC

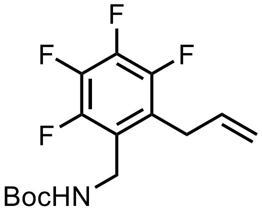

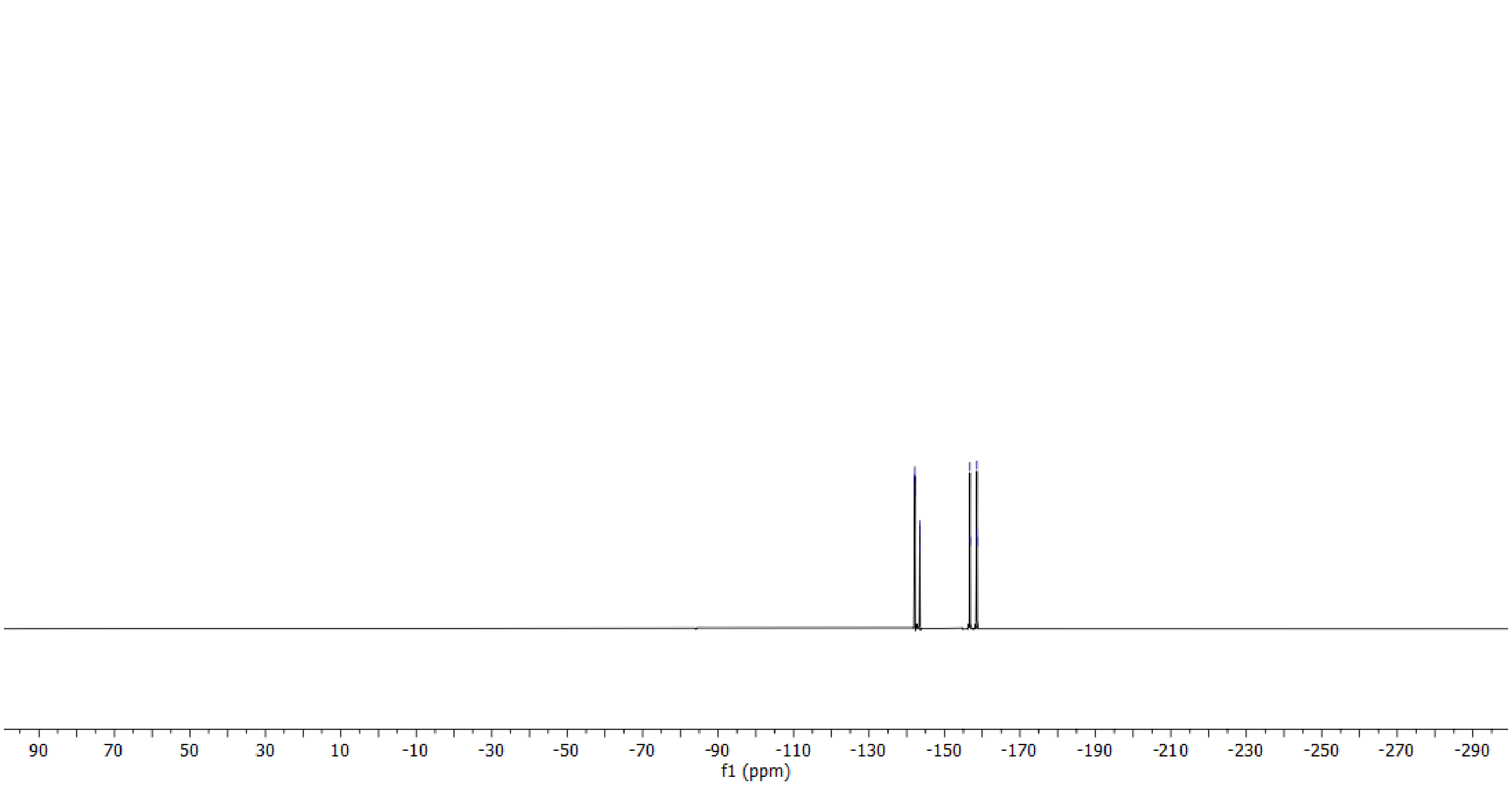

^{1}H NMR of *N*-Boc-2,3,5,6-tetrafluoro-4-(pentafluorophenyl)benzylamine (32a)

$CDCl_3$, 23 ºC

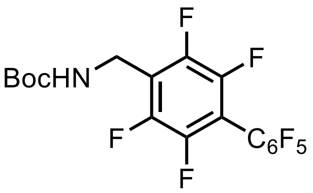

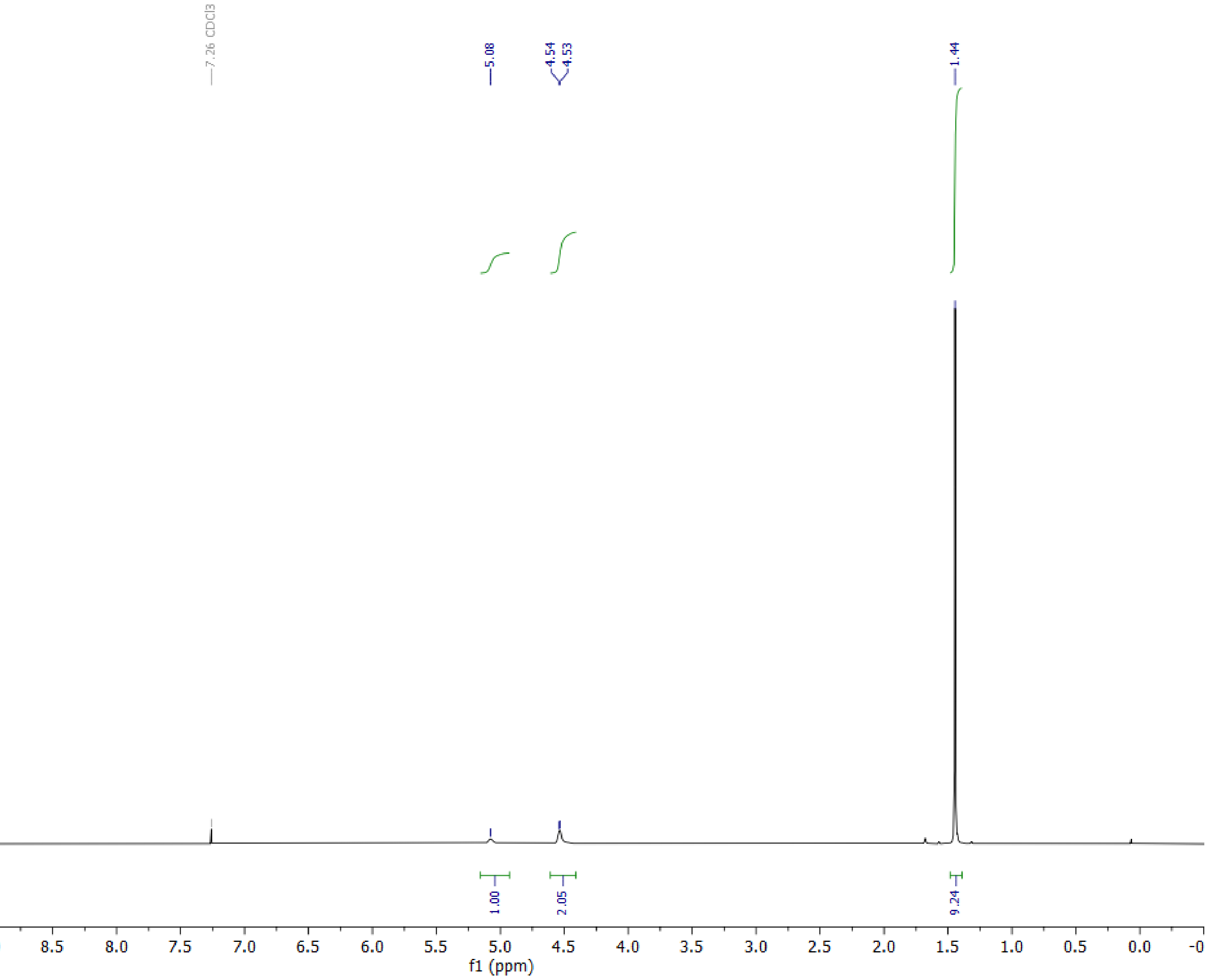

^{13}C NMR of *N*-Boc-2,3,5,6-tetrafluoro-4-(pentafluorophenyl)benzylamine (32a)

$CDCl_3$, 23 ºC

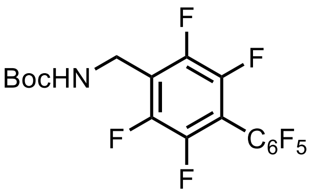

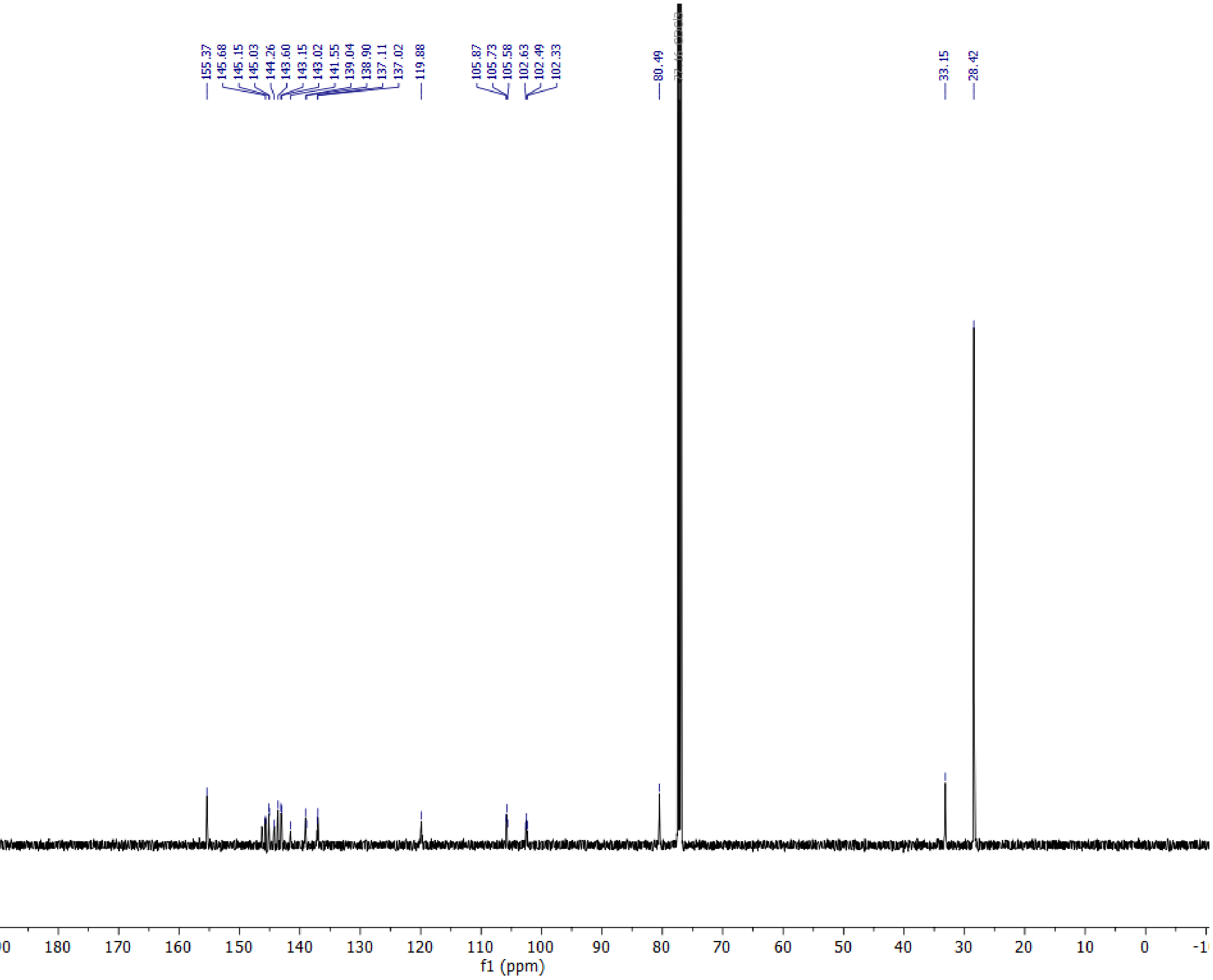

^{19}F NMR of *N*-Boc-2,3,5,6-tetrafluoro-4-(pentafluorophenyl)benzylamine (32a)

$CDCl_3$, 23 ºC

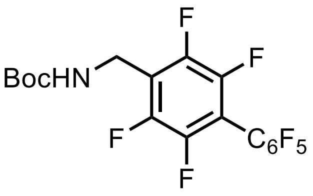

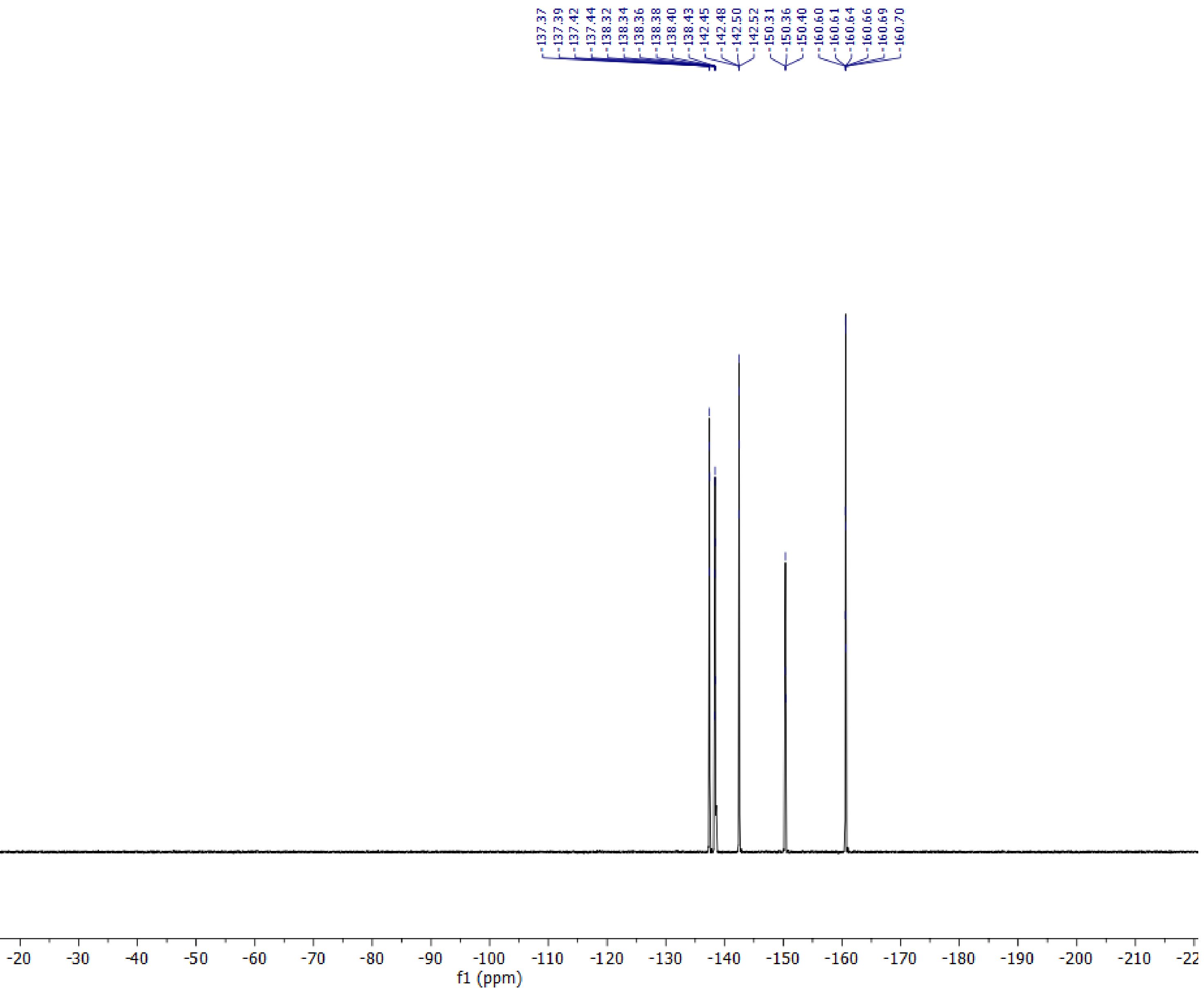

^{1}H NMR of *N*-Boc-2,3,4,6-tetrafluoro-5-(pentafluorophenyl)benzylamine (32b)

$CDCl_3$, 23 ºC

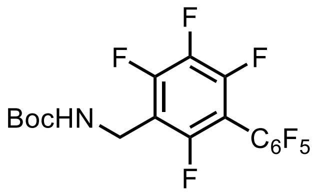

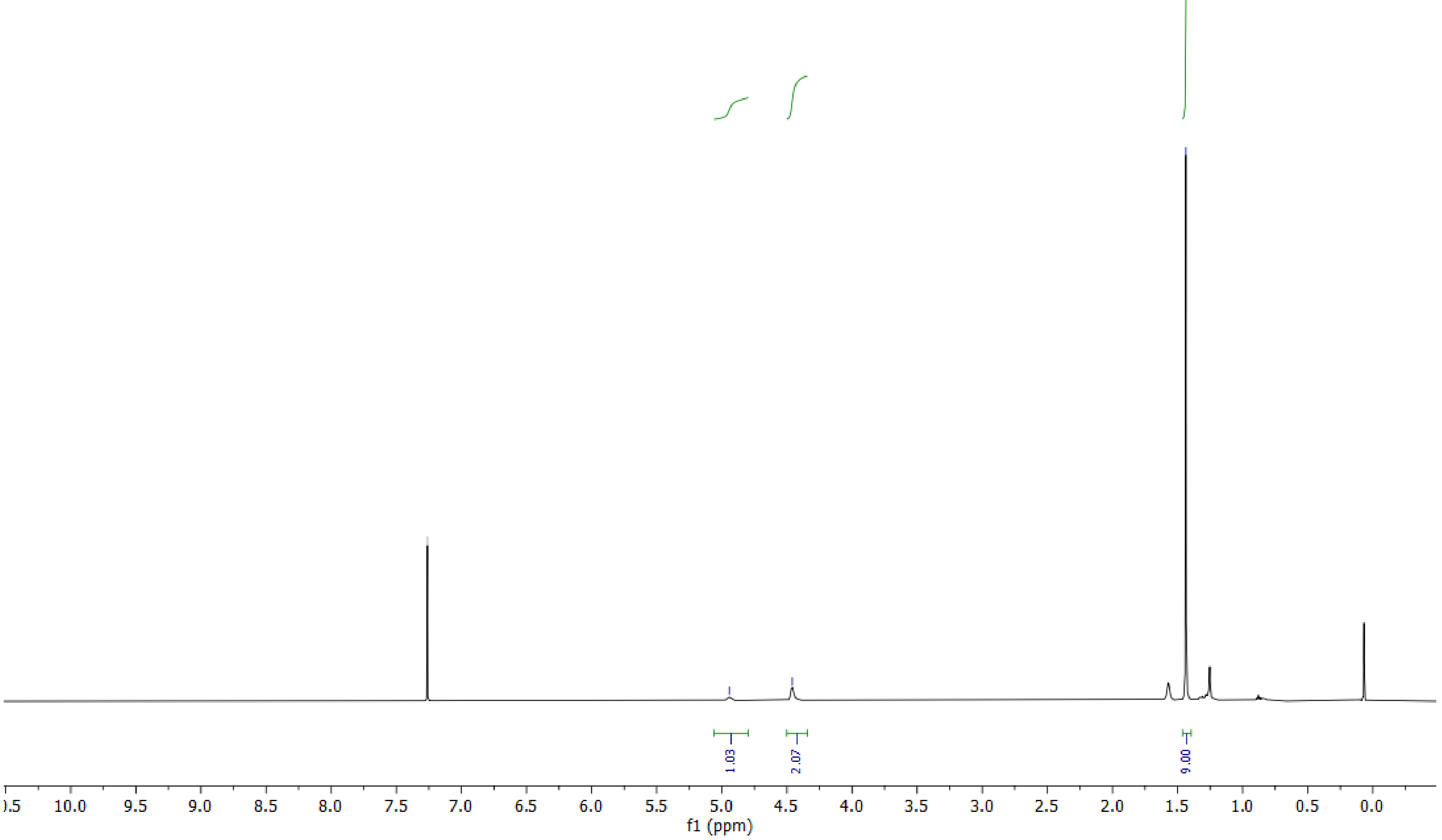

^{13}C NMR of *N*-Boc-2,3,4,6-tetrafluoro-5-(pentafluorophenyl)benzylamine (32b)

$CDCl_3$, 23 ºC

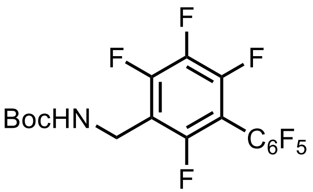

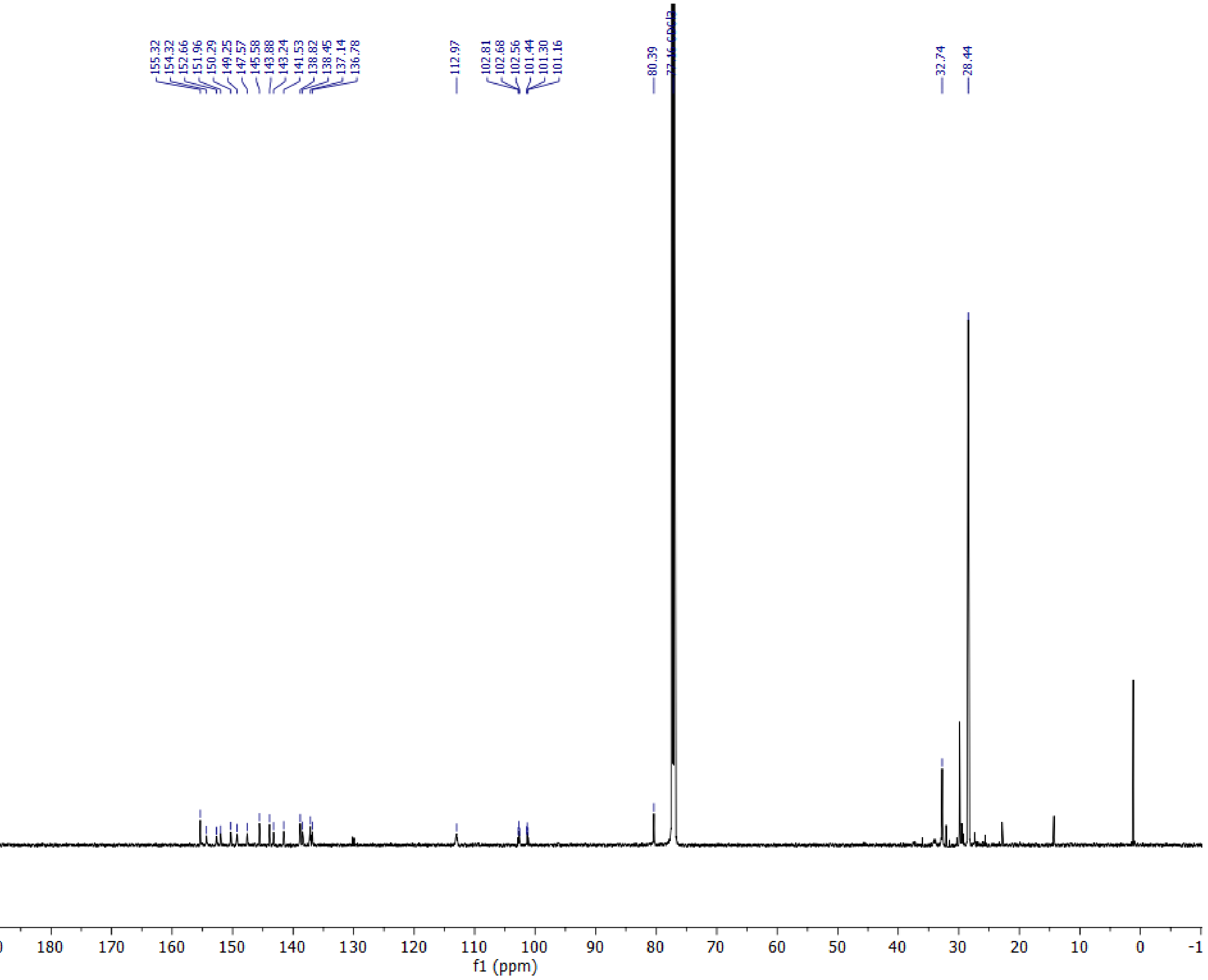

^{19}F NMR of *N*-Boc-2,3,4,6-tetrafluoro-5-(pentafluorophenyl)benzylamine (32b)

$CDCl_3$, 23 ºC

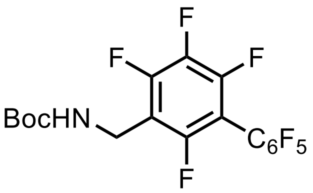

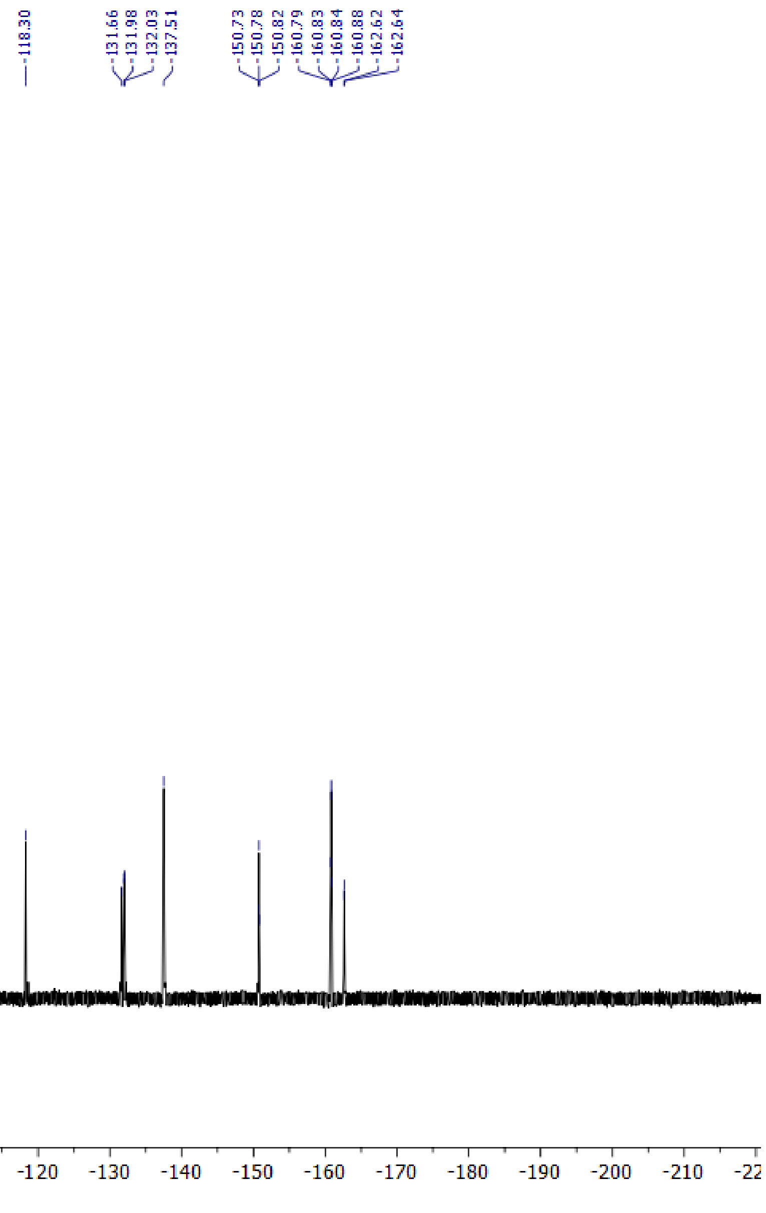

^{1}H NMR of *N*-Boc-2,3,5,6-tetrafluoro-4-((1'R,2'S,5'R)-2'-isopropyl-5'-methylcyclohexylcarboxyl)benzylamine (33a)

$CDCl_3$, 23 ºC

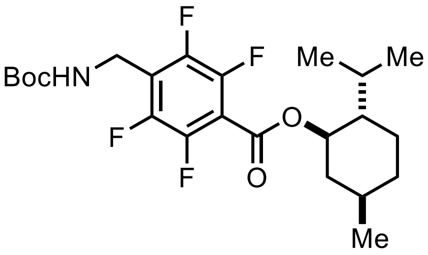

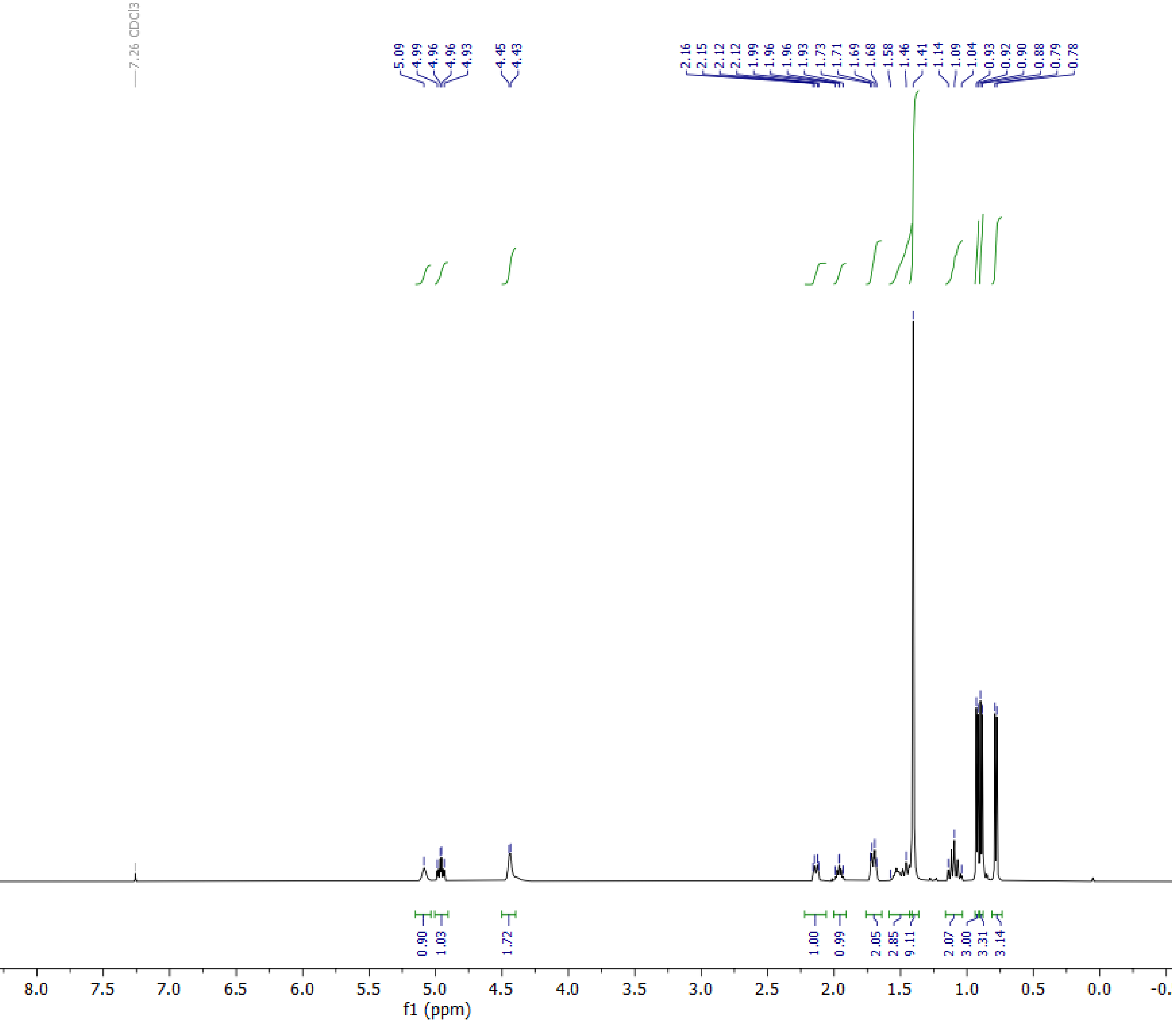

^{13}C NMR of *N*-Boc-2,3,5,6-tetrafluoro-4-((1'R,2'S,5'R)-2'-isopropyl-5'-methylcyclohexylcarboxyl)benzylamine (33a)

$CDCl_3$, 23 ºC

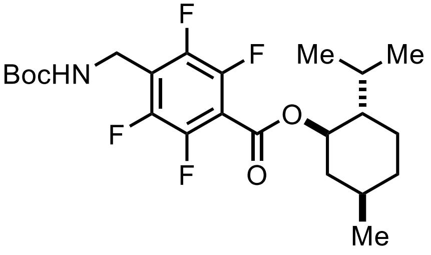

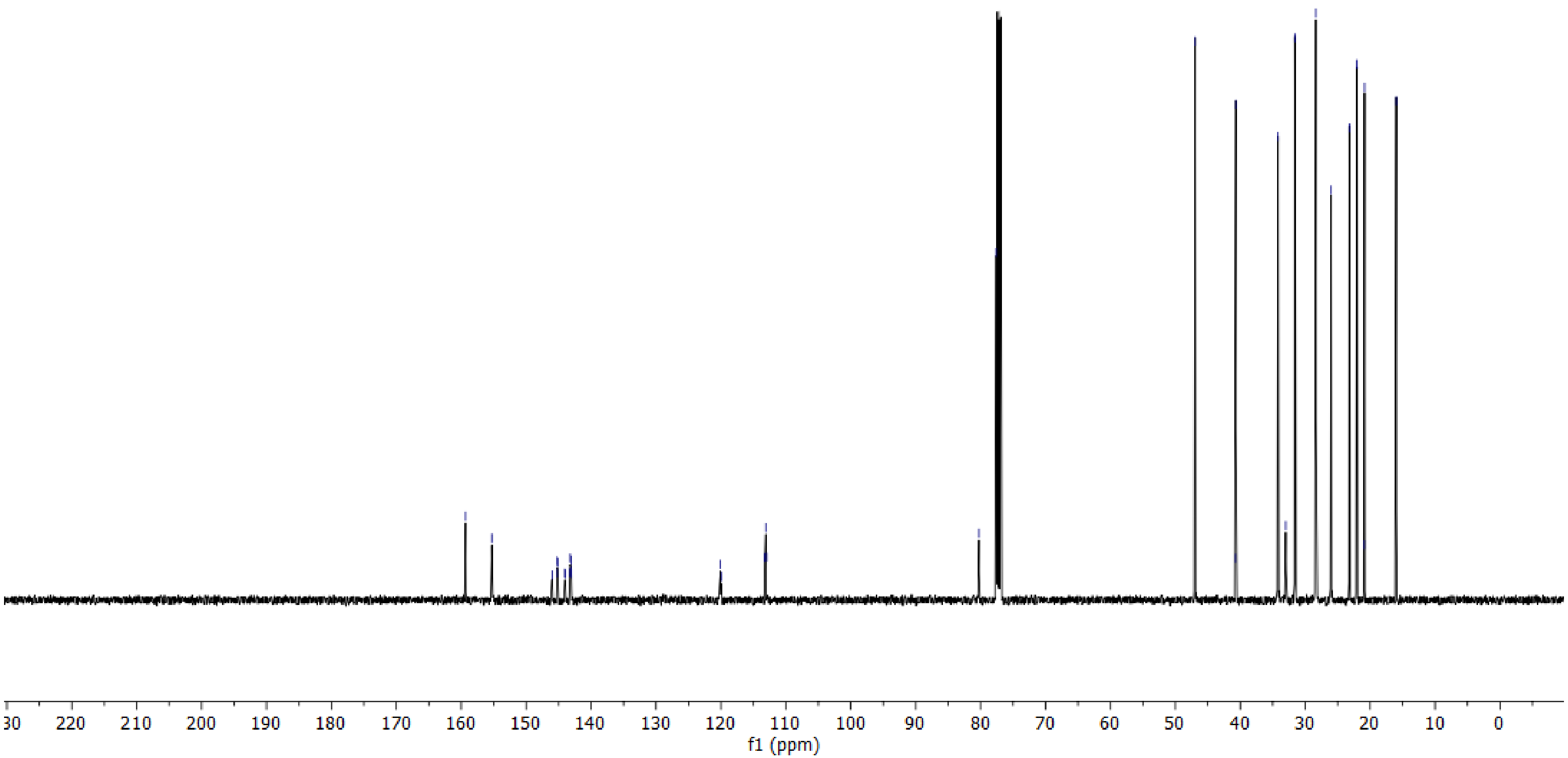

^{19}F NMR of *N*-Boc-2,3,5,6-tetrafluoro-4-((1'R,2'S,5'R)-2'-isopropyl-5'-methylcyclohexylcarboxyl)benzylamine (33a)

CDCl$_3$, 23 ºC

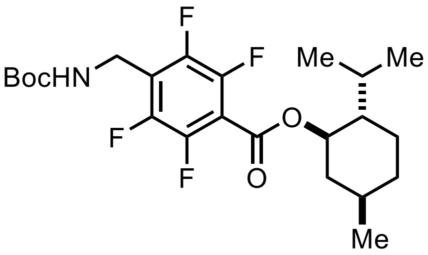

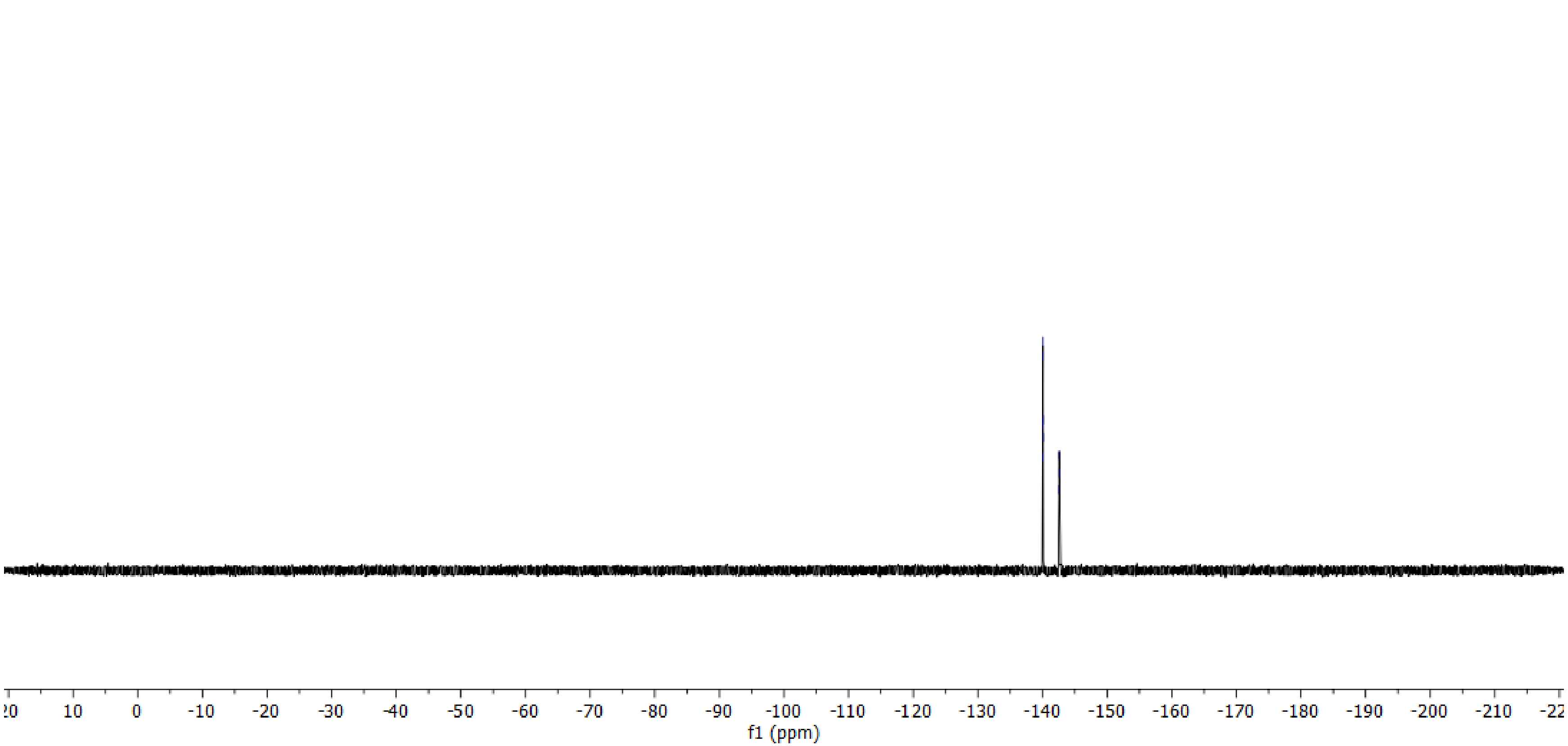

^{1}H NMR of *N*-Boc-2,3,4,5-tetrafluoro-6-((1'R,2'S,5'R)-2'-isopropyl-5'-methylcyclohexylcarboxyl)benzylamine (33b)

$CDCl_3$, 23 ºC

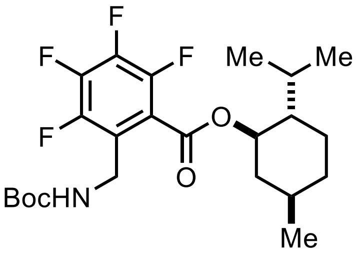

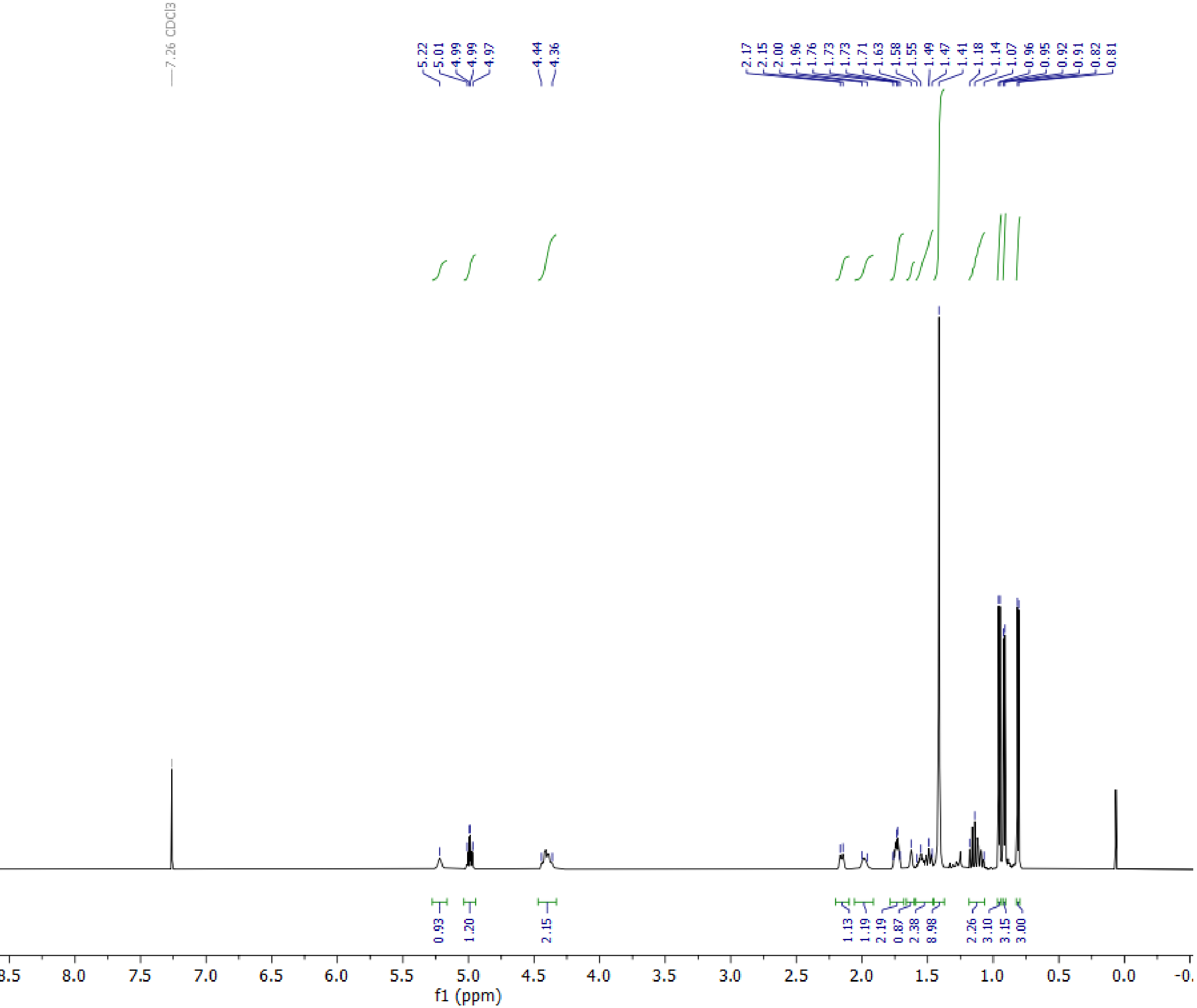

^{13}C NMR of *N*-Boc-2,3,4,5-tetrafluoro-6-((1'R,2'S,5'R)-2'-isopropyl-5'-methylcyclohexylcarboxyl)benzylamine (33b)

CDCl3, 23 ºC

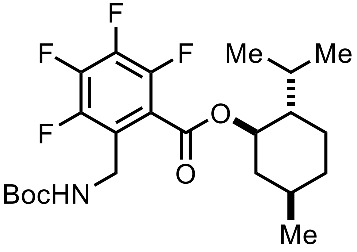

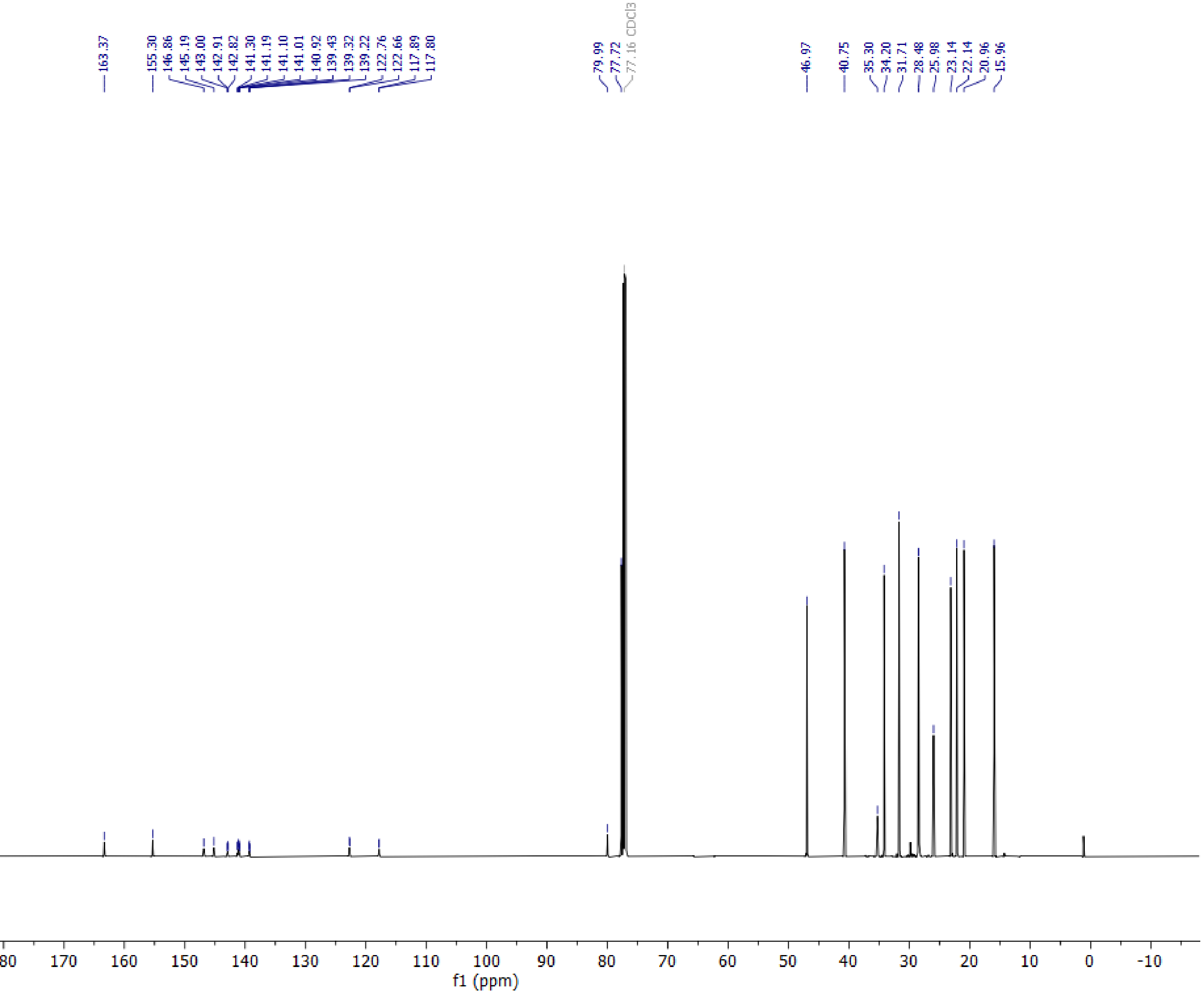

^{19}F NMR of *N*-Boc-2,3,4,5-tetrafluoro-6-((1’R,2’S,5’R)-2’-isopropyl-5’-methylcyclohexylcarboxyl)benzylamine (33b)

$CDCl_3$, 23 ºC

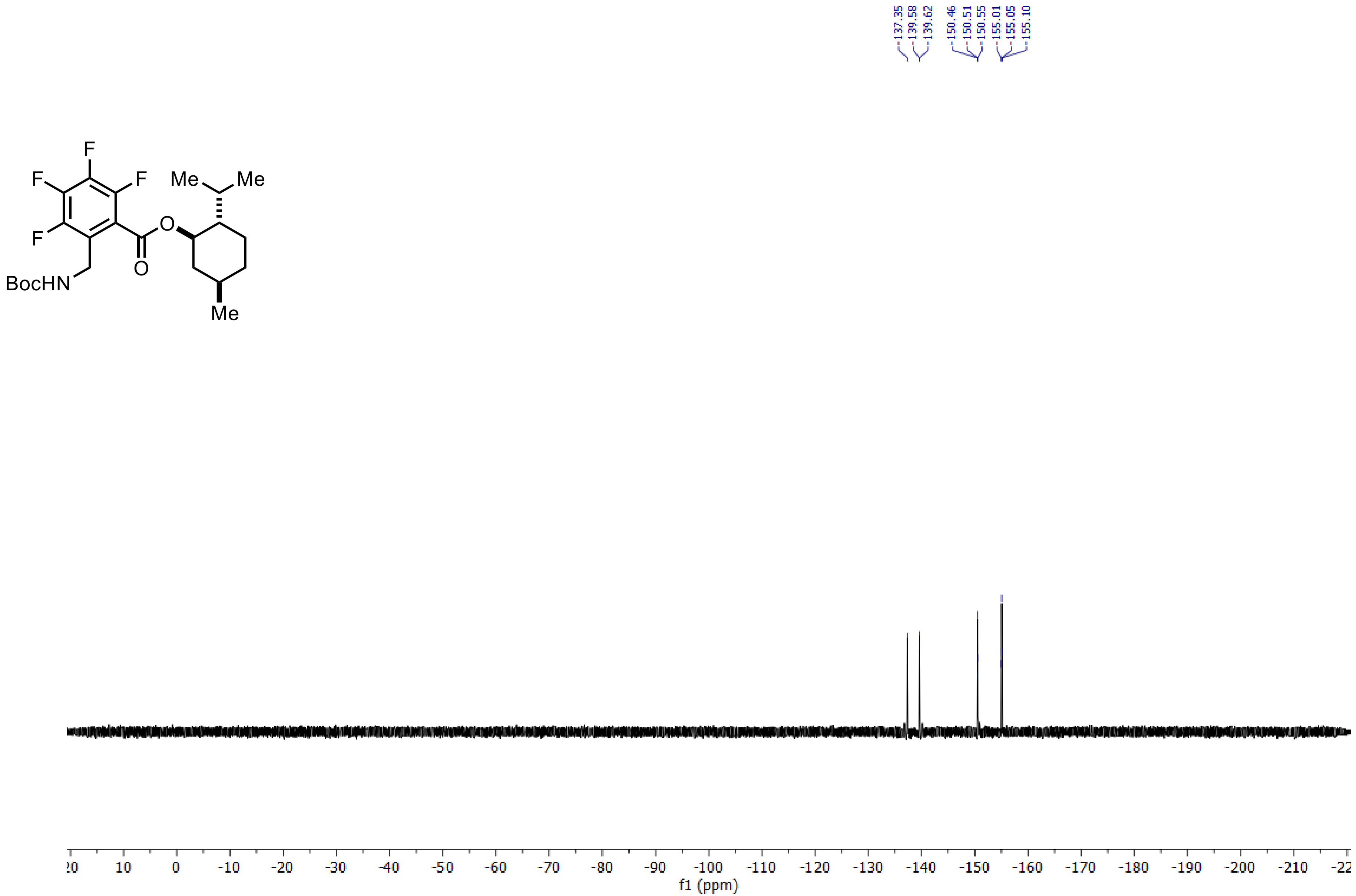

^{1}H NMR of Cyclopentylmethyl-pentafluorobenzene (34a)

$CDCl_3$, 23 ºC

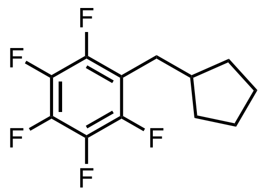

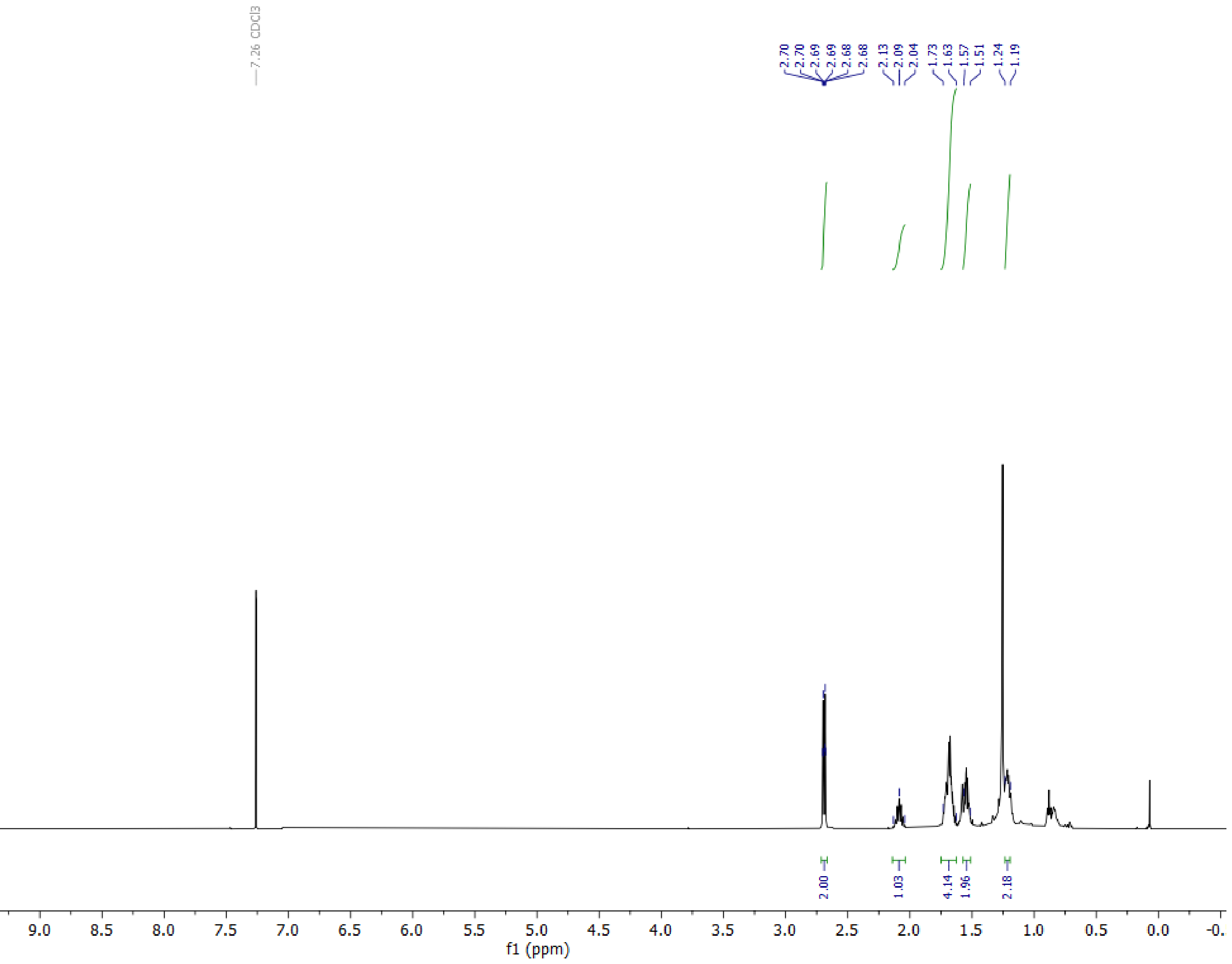

^{13}C NMR of Cyclopentylmethyl-pentafluorobenzene (34a)

$CDCl_3$, 23 ºC

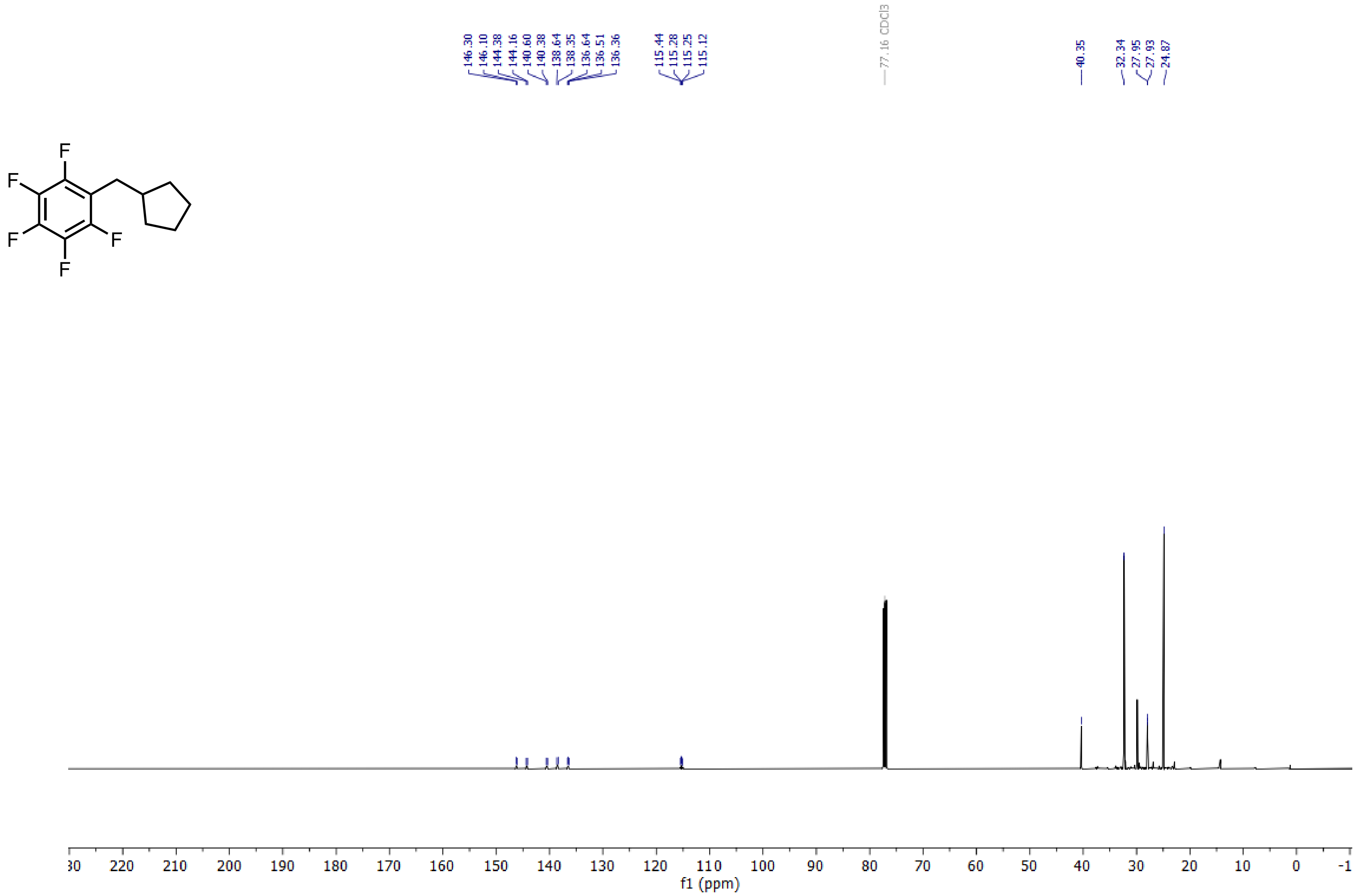

^{19}F NMR of Cyclopentylmethyl-pentafluorobenzene (34a)

$CDCl_3$, 23 ºC

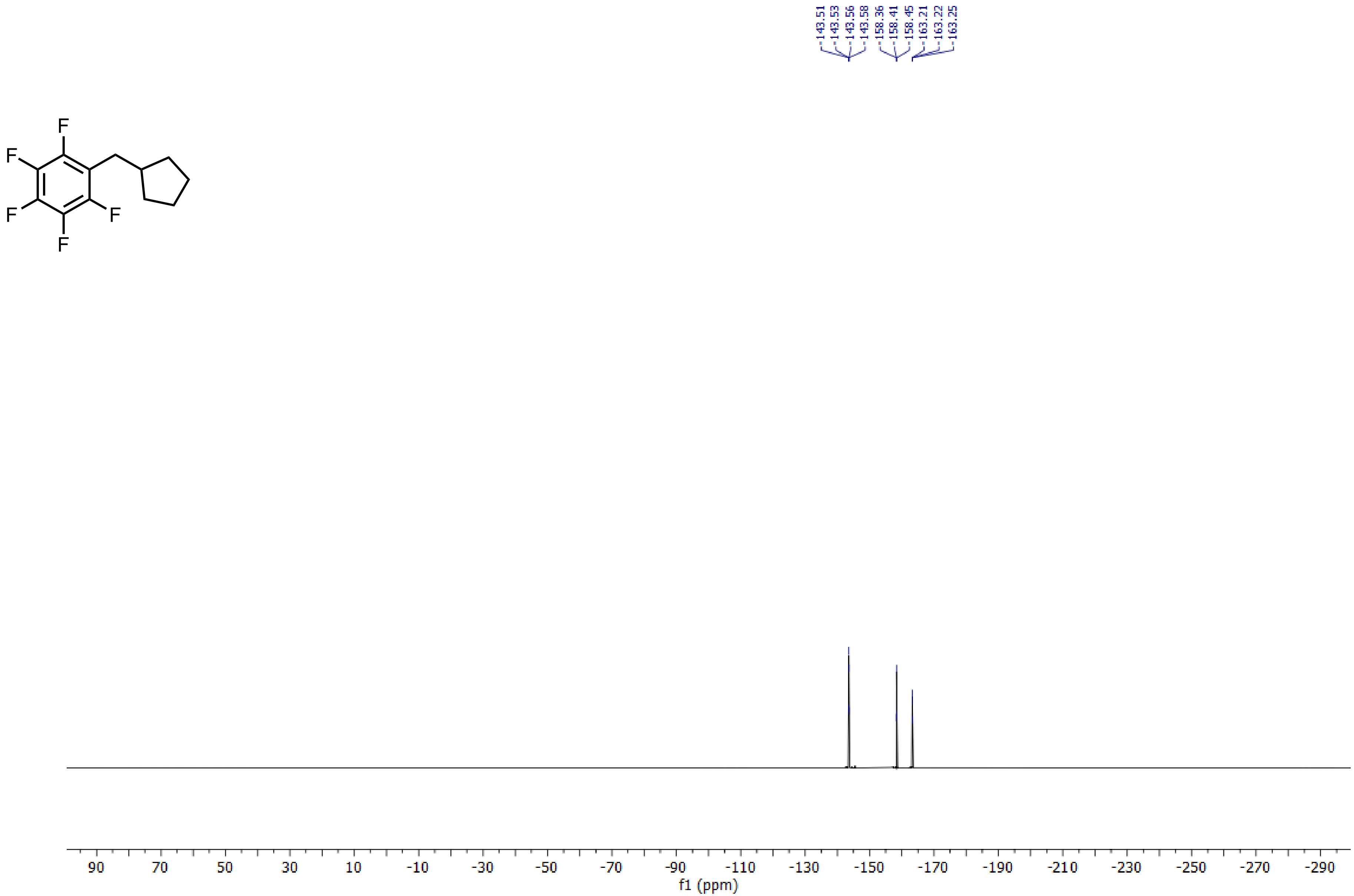

REFERENCES

1. B. Šket, M. Zupan, N. Zupančič, B. Pahor, *Tetrahedron* **1991**, *47*, 5029-5042.
2. R. Shang, Y. Fu, Y. Wang, Q. Xu, H.-Z. Yu, L. Liu, *Angew. Chem. Int. Ed.* **2009**, *48*, 9350-9354.
3. H. Jiang, W. Yang, H. Chen, J. Li, W. Wu, *Chem. Commun.* **2014**, *50*, 7202-7204.
4. H. J. Kuhn, S. E. Braslavsky, R. Schmidt, *Pure Appl. Chem.* **2004**, *76*, 2105-2146.
5. M. A. Cismesia, T. P. Yoon, *Chem. Sci.* **2015**, *6*, 5426-5434.
6. X. Sun, J. Chen, T. Ritter, *Nat. Chem.* **2018**, *10*, 1229-1233.
7. A. Lewandowski, L. Waligora, M. Galinski, *Electroanalysis*, **2009**, *21*, 2221-2227.
8. N. A. Romero, D. A. Nicewicz, *Chem. Rev.* **2016**, *116*, 10075-10166.
9. M. S. Lowry, J. I. Goldsmith, J. D. Slinker, R. Rohl, R. A. Pascal, Jr., G. G. Malliaras, S. Bernhard, *Chem. Mater.* **2005**, *17*, 5712-5719.
